# A pharynx-to-brain sensory pathway mediates swallowing-like behavior evoked by mechanical stimulation

**DOI:** 10.1038/s41467-026-78007-9

**Published:** 2026-09-28

**Authors:** Qing Pei, Lifang Huo, Jie Cao, Xingxing Lai, Zhimin Ye, Dapeng Li, Wenjie Zou, Congping Shang

**Affiliations:** 1 School of Life Sciences, Sun Yat-Sen University, Guangzhou, China; 2 School of Basic Medical Sciences, The Fifth Affiliated Hospital, Guangzhou National Laboratory, Guangzhou Medical University, Guangzhou, China; 3 Department of Neurobiology, School of Basic Medical Sciences, Beijing Key Laboratory of Neural Regeneration and Repair, Advanced Innovation Center for Human Brain Protection, Capital Medical University, Beijing, China; 4 Institute of Molecular Physiology, Shenzhen Bay Laboratory, Shenzhen, China

**Keywords:** Neural circuits, Reflexes

## Abstract

As sharing the anatomical entrance to the larynx, swallowing plays not only an important role in food and water intake, but also a major role in defensive mechanism for preventing the risk of foreign matter aspiration. Besides water/acid, the mechanical stimulation of food or foreign matter could induce swallowing, yet the relevant sensory pathways remain poorly understood. Here, using male mice as a model, we clarify that vagal sensory neurons expressing Piezo2 responsibly transmit swallowing induced mechanical stimuli from the pharynx to tyrosine hydroxylase positive (TH + ) neurons in the nucleus of the solitary tract (NTS), then to ambiguus nucleus/rostral ventrolateral medulla (Amb/RVLM complex). Specific activation of Piezo2+ vagal sensory neurons or TH + NTS neurons can induce swallowing behavior. Either inhibition of TH + NTS neurons or Piezo2 knockout in vagal sensory neurons can abolish swallowing reflex. Notably, we elucidate that the TH + NTS-Amb/RVLM pathway serves as a shared downstream pathway for both mechanical and chemical stimuli-elicited swallowing. Collectively, we describe a pharynx-to-brain sensory pathway that provides a basis for central integration of swallowing and airway defensive reflexes.

## Abstract (web summary)

This study elucidates a pharynx-to-brain sensory pathway that Piezo2+ vagal sensory neurons transmit mechanical signals to brain stem regions during mechanically evoked swallowing in mice.

## Introduction

The swallowing reflex is a vital neuromuscular process that ensures not only nutrition intake^1^, but also the safe passage in preventing the entrance of food and/or other materials into the lower respiratory tract. Following the voluntary oral phase of swallowing, the involuntary reflex phase of swallowing consists of the oropharyngeal and esophageal phases^1–4^. During these phases, several muscles are sequential activation, including the tongue, pharyngeal and laryngeal muscles, to push the chewed food bolus or liquid from the oral cavity to the esophagus^1,3,4^ while preventing aspiration into the airways^5–8^. Therefore, a thorough analysis of the neural circuit mechanisms underlying swallowing behavior holds significant scientific importance.

Swallowing can be triggered by natural chemical or physical stimulation of receptors in the pharynx and/or larynx (leading to effective swallowing in cases where paralyzing agents are involved). Chemical stimuli include water, salt, acid solutions^9–13^. Physical stimulation incorporates oropharyngeal thermal stimulation^14^ and laryngeal mucosal electrical stimulation^15^. Electrical stimulation of the superior laryngeal nerve (SLN) and the glossopharyngeal nerve (GPN) can also induce the swallowing reflex^16–19^. Additionally, the mechanical force generated by the food bolus^20^, and direct physical probing of the throat^13^ can also successfully induce the swallowing reflex. The pharynx contains a rich population of taste and mechanical sensory neurons, whose functions are being progressively elucidated^20–23^. The initiation of the pharyngeal swallowing is mediated by receptors arranged along the pharyngeal mucosa, which are innervated by branches of the vagus nerve SLN^1,24–27^.

The pharyngeal phase of swallowing is initiated by the central pattern generators (CPGs). which consists of the dorsal swallowing group (NTS-DSG) located in the nucleus tractus solitarii (NTS) and adjacent reticular formation, and the ventral swallowing group (VLM-VSG) situated in the ventrolateral medulla (VLM) near the nucleus ambiguus (NA)^1,4,5^. Peripheral sensory information is first transmitted to the NTS-DSG and then relayed to the VLM-VSG. This subsequently activates the V, VII, IX, X, and XII cranial nerves, thereby precisely regulating swallowing reflex^1^. Moreover, afferent fibers of the vagus nerve from the pharynx, as well as SLN, terminate in the NTS^1^. Pharyngeal phase swallowing is controlled not only by the medullary CPG but may also be driven by cortical autonomic regulation^4,28,29^.

NTS, as a key component of the CPG, is the primary sensory center connecting visceral organs to the brain. It receives convergent synaptic inputs from vagal afferents and ascending spinal pathways. NTS neurons respond to various internal sensory signals, including visceral sensory inputs, intestinal distension, and nutritional signals^30–32^. These sensory capabilities enable the NTS to coordinate fundamental physiological functions, including swallowing. Studies have shown that electrical stimulation of the NTS can induce swallowing behavior similar to that elicited by stimulation of the SLN. However, the specific neuronal subtypes and their circuit mechanisms regulating swallowing behavior in the NTS remain unclear. In recent years, the tyrosine hydroxylase neurons (TH) in the NTS have been identified as a feeding-related neuron^33–35^. Swallowing is an indispensable step in feeding. Consequently, these studies indicate that TH neurons in the NTS may be involved in swallowing.

The vagal ganglia play a key role in the information transmission between the brain and the peripheral (including the pharynx, larynx, etc.). The pharynx is densely innervated by the vagus nerve and the SLN, whose other ends project to the NTS^36^. The vagal afferent nerves are anatomically heterogeneous, with their peripheral axons forming characteristic sensory endings specialized for detecting chemical or mechanical stimuli^37,38^, thereby triggering protective reflexes such as swallowing and coughing. In recent years, Liberles Laboratory discovered that vagal P2RY1 neurons serve as crucial chemoreceptors in the larynx, responsible for detecting chemical stimuli such as water and acid^13^. However, the specific neuronal subpopulation that perceives mechanical stimuli in the pharynx and triggers the swallowing reflex remains unclear.

The fine structure of peripheral sensory input to central neural networks ensures the sequential execution of swallowing^39^. Any disruption at any step can lead to dysphagia. Dysphagia, or difficulty swallowing, is a major cause of aspiration pneumonia^40^, malnutrition^41^, and can ultimately result in choking and death. Furthermore, studies have reported more severe cognitive dysfunction in patients with dysphagia^42^. Therefore, the rehabilitation of dysphagia has become an important focus of clinical management. Currently, pharyngeal mechanical sensitivity assessment has been developed for evaluating patients with dysphagia^43^. Stimulating the anterior faucial pillars (AFP) and other oropharyngeal regions has also become a common therapeutic approach for dysphagia^14^.

There is no doubt that the swallowing CPG and its extensive connections within the highly complex swallowing neural network are crucial for understanding the nature of dysphagia. To date, even in experimental fields, there is limited knowledge about the synaptic neurotransmitter mechanisms of brainstem swallowing CPG, especially the NTS. Here, we established a mouse model to induce swallowing via mechanical stimulation of the pharynx. Using genetic and sequencing techniques, we identified a subset of NTS neuron subtypes—TH neurons—that regulate swallowing, along with their downstream target nuclei—Amb/RVLM. Furthermore, we elucidated that the Piezo2 ion channel serves as a critical pharyngeal mechanoreceptor, which detects pharyngeal mechanical stimuli and transmits pharyngeal sensory information to the brainstem. In summary, we identified a mechanosensory pathway that was essential for pharyngeal stimulation-evoked reflexive swallowing under anesthetized conditions. Our work provides deeper insights into the mechanisms regulating swallowing.

## Results

### NTS mediated swallowing behavior induced by mechanical stimulation

**Fig. 1:**
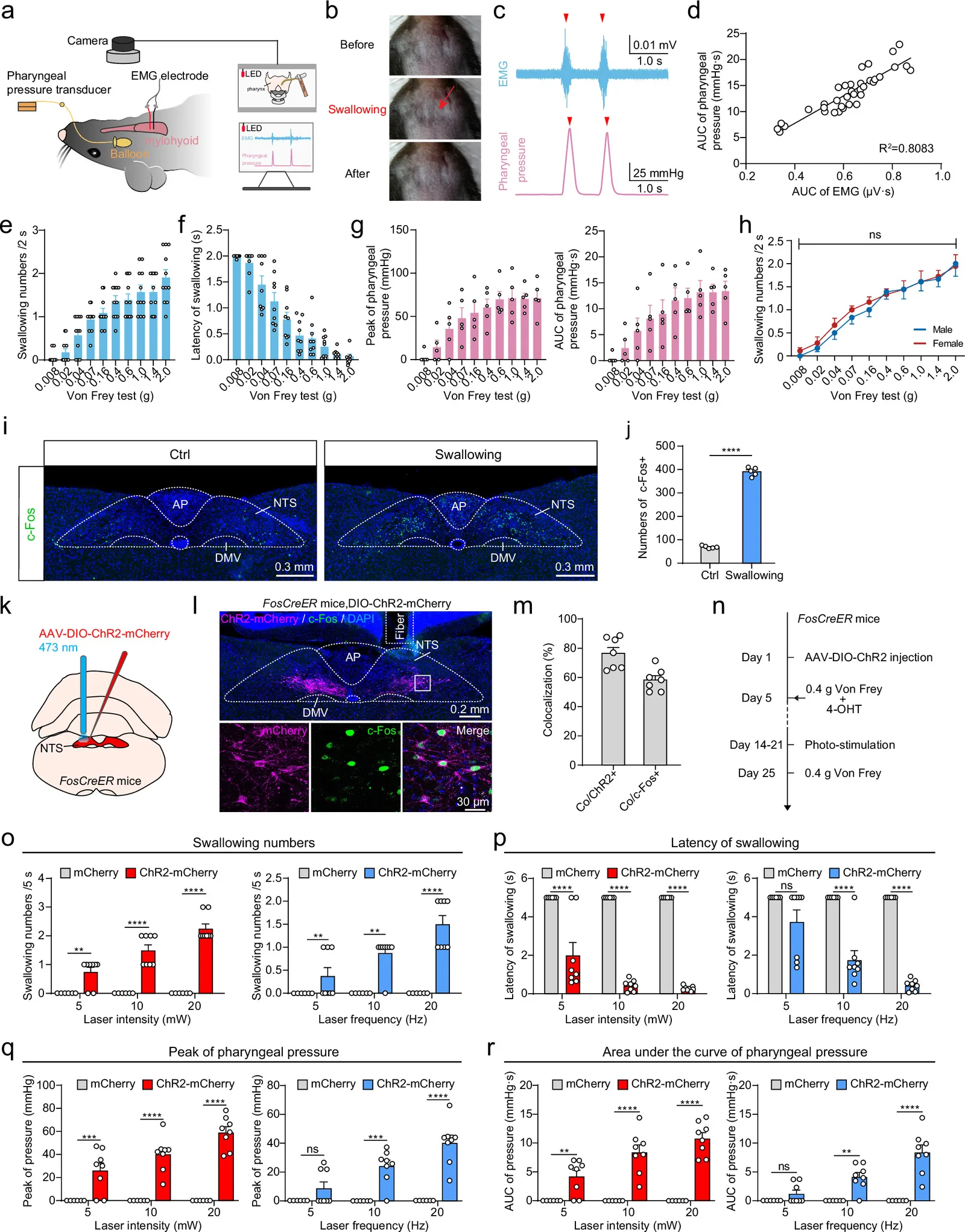
NTS mediated swallowing behavior induced by mechanical stimulation. **a** Schematic of the behavioral paradigm for monitoring swallowing induced by mechanical stimulation (Von Frey) of the pharynx in anesthetized mice. **b** Representative images of a mouse swallowing. **c** Example traces of electromyography (EMG) (blue curve) and pharyngeal pressure (pink curve) during swallowing (red triangles) via pharyngeal mechanical stimulation in *WT* mice. **d** The relationship between the AUC of EMG and pharyngeal pressure in response to swallowing induced by mechanical stimulation of the pharynx. Each point represents a swallowing (*n* = 11 mice). **e**, **f** Number (**e**) and latency (**f**) of swallowing induced by different threshold mechanical stimuli applied to the pharynx in *WT* mice (**e** *n* = 11 mice, **f** *n* = 9 mice). **g** Peak and AUC of pharyngeal pressure response to mechanical stimulation-induced swallowing (*n* = 5 mice). **h** Statistical diagram illustrating the number of swallowing induced by mechanical stimulation of the pharynx in male (*n* = 6 mice) and female mice (*n* = 5 mice). **i** Brain images immunostained for c-Fos, activated after mechanical stimulation of the pharynx with 0.4 g Von Frey for 60 min or no stimulation (See Fig. S1). **j** Number of c-Fos+ neurons in the NTS of *WT* mice activated by mechanical stimulation of the pharynx and mice not stimulated (*n* = 5 mice). **k** Schematic diagram illustrating the positions of virus injection and optical fiber implantation in the NTS of *FosCreER* mice. **l**, **m** Representative images displaying the expression of ChR2-mCherry (magenta) and c-Fos (green) in the NTS (**l**), along with statistical data on the co-localization between these neurons (*n* = 7 mice) (**m**). **n** Schematic showing the procedure for gaining genetic control over swallow-regulating neurons. **o–r** Swallowing numbers (**o**), latency (**p**), peak (**q**) and AUC of pharyngeal pressure (**r**) respond by light-evoked swallowing in *FosCreER* mice (mCherry: *n* = 6 mice, ChR2: *n* = 8 mice) (See Fig. S2). Data were shown as mean ± s.e.m. (error bars). The broken white lines in the section images represent boundaries of brain regions. Statistical analysis in **j** was performed using Student *t* tests. Statistical analysis in (**h**, **o**–**r**) were performed using Two-way ANOVA (**P < 0.01, ***P < 0.001, ****P < 0.0001, ns ≥ 0.05). Source data are provided as a Source Data file.

Although various studies have attempted to elucidate the swallowing mechanism elicited by chemical stimulation of the pharynx, there are few studies on inducing swallowing by mechanical stimulation of the pharynx in mice. In this study, point mechanical stimulation was applied to the exposed pharynx of mice using Von Frey filaments, simulating bolus activity, to induce the pharyngeal phase of swallowing. This method established a reflexive swallowing model in mice under anesthesia (Fig. 1a, b and Supplementary Fig. 1a). The mechanical strength gradually rose from 0.008 g to 2 g. The time interval between different forces was at least 1 min. Specifically, within the low-threshold stimulus intensity range (0.008 g–0.16 g), each mechanical stimulation triggered 0 or 1 swallowing. When the stimulus intensity reached 0.4 g and above, each stimulation could elicit 1 or 2 swallowing. During swallowing, the contraction of the muscle complex including the mylohyoid muscle generates pressure in the pharynx, propelling food into the esophagus^1,5,8^. To visually and quantitatively assess the swallowing in mice, we utilized parameters and indicators such as swallowing number (times of laryngeal elevation visually observed), pharyngeal pressure, and electromyography (EMG) of the mylohyoid muscle in response to swallowing (Fig. 1c). Quantitative analysis of the pharyngeal pressure and EMG area under the curve (AUC) revealed a positive correlation (Fig. 1d). What’s more, the number of swallowing increased with incremental mechanical stimulation intensity (Fig. 1e), while the latency of swallowing gradually decreased (Fig. 1f). Additionally, the amplitude and AUC of pharyngeal pressure, which reflect swallowing, also increased with increasing mechanical stimulation intensity (Fig. 1g). The sex of the mice did not influence the swallowing reflex elicited by mechanical pharyngeal stimulation (Fig. 1h). In all other experiments, male mice were used.

Subsequently, we screened for brain nuclei associated with reflexive swallowing using c-Fos immunostaining to indicate the expression of this immediate-early gene. We exposed the pharynx of wild-type (*WT*) mice, applied mechanical stimulation to the pharynx using 0.4 g Von Frey filaments, and perfused the brains for neuronal activation marker (c-Fos) immunostaining 90 min later. Control mice had their pharynx exposed but received no stimuli. The quantitative analysis of c-Fos immunostaining results indicated that the expression of c-Fos in the NTS of the swallowing group was significantly higher than that of the control group (Fig. 1i, j). This proved that NTS is involved in the swallowing behavior of mice, which is consistent with earlier studies^1,44^. Additionally, c-Fos neuronal activity was observably higher in the ambiguous nucleus (Amb) /rostral ventrolateral medulla (RVLM), paraventricular hypothalamic nucleus (PVN), lateral parabrachial nucleus (LPB), and locus coeruleus (LC) (Supplementary Fig. 1b).

The NTS is the initial relay nucleus in the medulla oblongata that receives the afferent input of swallowing receptors in the pharynx through the vagal ganglia. Then we employed the FosTRAP strategy to test whether these activated NTS neurons could initiate swallowing by mechanical stimulation of the pharynx. On day 1, AAV-DIO-ChR2-mCherry was injected into the NTS of *FosCreER* mice, and an optical fiber was embedded above the NTS. On day 5, the pharynx was mechanically stimulated using a 0.4 g Von Frey filament, and intraperitoneal injections of 4-hydroxytamoxifen were administered. In contrast, the control group mice were only exposed to the pharynx without being given mechanical stimulation (Fig. 1k, n). After 2-3 weeks, the optogenetic activation of NTS neurons in *FosCreER* mice that underwent mechanical stimulation-TRAP resulted in distinctly stronger swallowing responses compared to the control group. The number of swallowing, the latency to initiate swallowing, the amplitude and AUC of pharyngeal pressure, which characterize the swallowing, exhibited dependencies on light power and frequency. The number of swallowing increased with enhanced laser power and frequency (Fig. 1o and Supplementary Fig. 2a). The latency of swallowing shortened with an increase in laser power and frequency (Fig. 1p and Supplementary Fig. 2b). The amplitude and AUC of pharyngeal pressure enlarged with the enhancement of laser power and frequency (Fig. 1q, r and Supplementary Fig. 2c, d). To examine the specificity and efficiency of ChR2-mCherry labeling, we conducted co-immunostaining for c-Fos and mCherry in the same sections of the NTS (Fig. 1l and Supplementary Fig. 2e). Results proved that a majority of ChR2-mCherry+ cells (76.52% ± 4.69%, *n* = 7 mice) were positive for c-Fos, whereas a portion of the c-Fos+ cells (58.28% ± 2.91%, *n* = 7 mice) exhibited positivity for ChR2-mCherry (Fig. 1m). These data indicated that the FosTRAP strategy labels relevant NTS neurons with acceptable specificity and efficiency. The evidence above adequately demonstrated that the NTS played a crucial role in mediating swallowing behavior induced by mechanical stimulation.

### TH+NTS neurons were the key neuronal subtype for triggering swallowing behavior

**Fig. 2:**
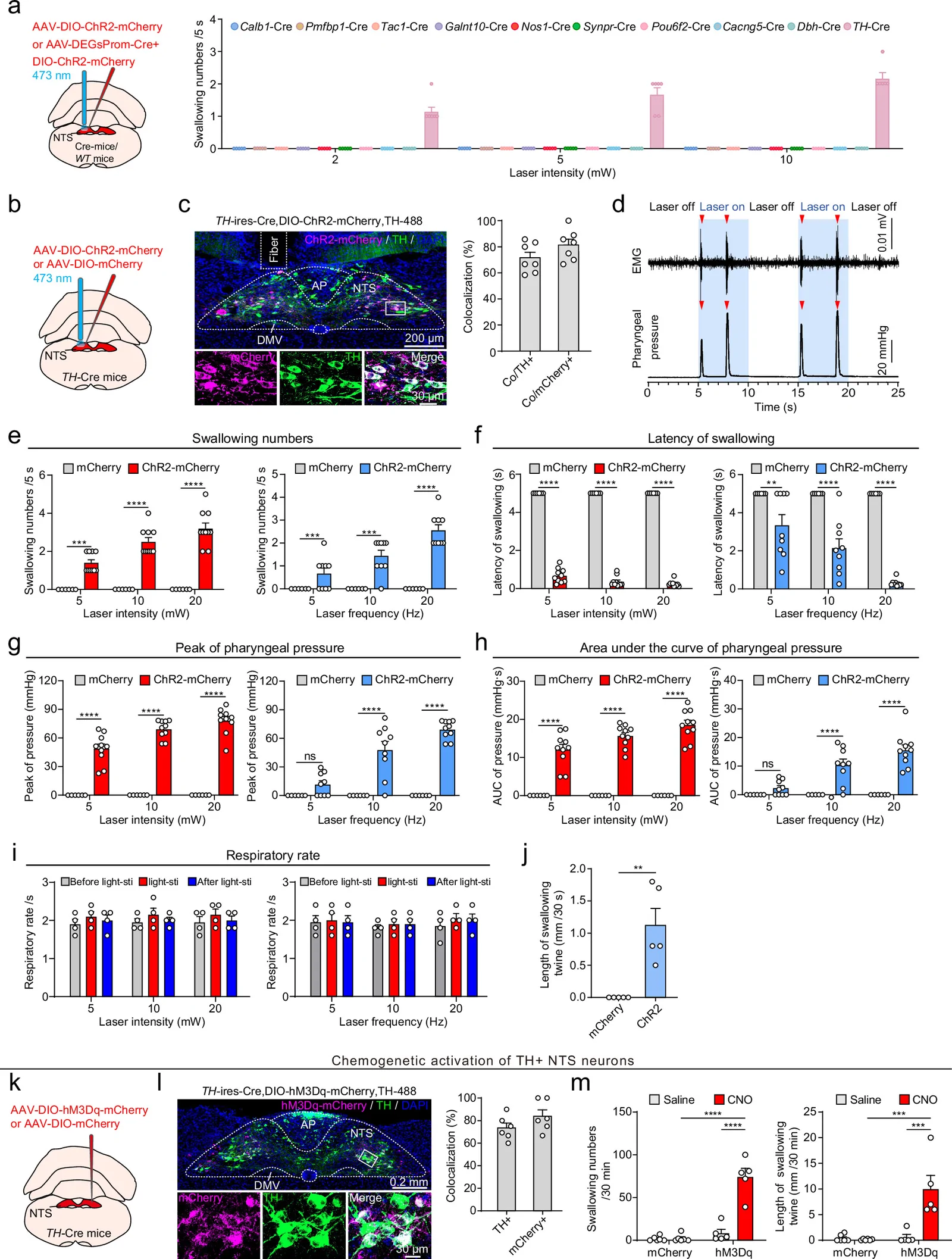
TH + NTS neurons were the key neuronal subtype for triggering swallowing behavior. **a** Schematic diagram showing position of virus injection and optical fiber implantation in intermediate subregions of the NTS (Bregma –7.48 mm) of Cre-line and *WT* mice (*n* = 5 mice). Light-evoked swallowing number was analyzed. **b** Schematic diagram illustrating the positions of virus injection and optical fiber implantation in the NTS of *TH*-ires-Cre mice. **c** Example micrographs showing ChR2-mCherry expression and the optical fiber track above ChR2-mCherry+ neurons in the NTS. Example micrographs (left) and statistical data (right) showing colocalization of ChR2-mCherry+ (magenta) with the TH+ neurons (green) from the NTS (*n* = 7 mice). **d** Example of EMG of the mylohyoid and pharyngeal pressure response to light-evoked (blue shadow) swallowing behavior (red triangles). **e**–**h** Quantitative analysis of swallowing with optogenetic activation of TH + NTS neurons (**e**–**h** left: mCherry: *n* = 6 mice, ChR2: *n* = 10 mice) and **e**–**h** right: mCherry: *n* = 6 mice, ChR2: *n* = 9 mice) (See Fig. S5). **i** Respiratory rate of *TH*-ires-Cre mice upon activation of TH + NTS neurons by light were shown (mCherry: *n* = 4 mice, ChR2: *n* = 4 mice). **j** The length of swallowing twine during light-induced NTS within 30 s in *TH*-ires-Cre mice (mCherry: *n* = 5 mice, ChR2: *n* = 5 mice). **k** Schematic diagram showing position of virus (DIO-hM3Dq or DIO-mCherry) injection in the NTS of *TH*-ires-Cre mice. **l** Example coronal brain section and magnified field displaying colocalization of hM3Dq-mCherry+ neurons (magenta) with TH+ neurons (green) in the NTS of *TH*-ires-Cre mice (left), accompanied by statistical data on the co-localization of these neurons (right) (*n* = 6 mice). **m** Swallowing numbers (left) and the length of swallowing twine (right) chemically activated the hM3Dq-expressing NTS neurons of *TH*-ires-Cre mice within 30 min, as shown (mCherry: *n* = 6 mice, hM3Dq: *n* = 5 mice). Data were shown as mean ± s.e.m. (error bars). The broken white lines in the section images represent boundaries of brain regions. Statistical analysis in (**e**–**i** and **m**) was performed using Two-way ANOVA. Statistical analysis in (**j**) was performed using Student *t* tests (**P < 0.01, ***P < 0.001, ****P < 0.0001, ns ≥ 0.05). Source data are provided as a Source Data file.

Although NTS regulation of swallowing has been widely recognized, the specific subtypes of key neurons involved in swallowing remain unknown. Our previous work, using Single-nucleus RNA sequencing, has established a cellular atlas of NTS slices from *WT* mice^45^. Given the marker genes’ expression specificity from six neuronal clusters of the NTS, we selected the top candidates to screen for key subtypes triggering swallowing (Supplementary Fig. 3). We injected AAV-DIO-ChR2-mCherry into the intermediate subregions of the NTS (Bregma –7.48 mm) of Cre-line mice. Optic fibers were implanted above the NTS (Fig. 2a left). After ~two weeks of AAV expression, NTS neurons were activated by 473 nm blue light. Optical stimulation (473 nm, 20 Hz, 10 ms, 2, 5 and 10 mW) of the NTS in *TH*-ires-Cre mice consistently triggered swallowing behavior. In contrast, the optical activation of the NTS from other Cre-lines mice did not elicit obvious swallowing (Fig. 2a right and Supplementary Fig. 4b). In addition, we conducted optogenetic activation at different locations within the NTS in various Cre mouse lines. Optogenetic activation of the rostral (Bregma –7.08 mm) and caudal NTS (Bregma –7.92 mm) evoked swallowing reflex in *TH*-ires-Cre mice but not in mice from other Cre lines (Supplementary Fig. 4a, b). This result suggested that TH + NTS neurons may be crucial for inducing swallowing behavior.

Since the NTS is a large and heterogeneous nucleus, it remains to be determined whether its activation by pharyngeal mechanical stimulation also exhibits a spatial distribution pattern along the rostro-caudal axis. We systematically analyzed the distribution of c-Fos/TH co-labeled neurons in three subregions of the NTS—caudal, intermediate, and rostral—following pharyngeal mechanical stimulation-induced swallowing. The results showed that activated TH+ neurons were not uniformly distributed following mechanical stimulation of the pharynx, but instead exhibited significant regional enrichment, primarily within the intermediate subregion of the NTS. This indicated that the TH+ neurons population in NTS exhibited spatial heterogeneity, and clarified that pharyngeal sensory input primarily activates specific neurons located within the intermediate subpopulation (Supplementary Fig 4c, d). This was consistent with the results of optogenetic activation of subregions of the NTS inducing swallowing.

To further verify this hypothesis, we meticulously investigated the effect of light stimulation on the swallowing behavior of *TH*-ires-Cre mice by using optogenetic strategies (Fig. 2b). To assess the specificity and efficiency of ChR2-mCherry labeling, immunostaining for TH was conducted on NTS sections. The results showed that the majority of TH+ cells (71.53% ± 4.27%) expressed ChR2-mCherry + , and most ChR2-mCherry-expressing cells (81.37% ± 4.40%, *n* = 7 mice) were TH positive (Fig. 2c). These data illustrated that the optogenetic strategy for labeling NTS neurons in *TH*-ires-Cre mice is both specific and efficient.

Next, we recorded the pharyngeal pressure and electromyographic responses of the mylohyoid muscle, induced by the continuous activation of TH + NTS neurons with 473 nm blue light for 5 seconds. Laser ON-OFF-ON procedure (473 nm, 20 Hz, 10 ms, 10 mW, 5 s) demonstrated a reliable increase in pharyngeal pressure and EMG (Fig. 2d). The control group injected with AAV-DIO-mCherry into the NTS exhibited no significant response. Importantly, the swallowing behavior in *TH*-ires-Cre mice was dependent on both light power and frequency of the light. Activation of TH + NTS neurons with enhanced laser power and frequency resulted in an increase in swallowing number, the amplitude and AUC of pharyngeal pressure (Fig. 2e, g, h and Supplementary Fig. 5a, c–d). The latency of swallowing tapered with increased laser power and frequency (Fig. 2f and Supplementary Fig. 5b). The oscillogram of pharyngeal pressure and EMG demonstrated manifested variations in dependency on light intensity (20 Hz, 10 ms, 5, 10 and 20 mW) and light frequency (10 mW, 10 ms, 5, 10 and 20 Hz) (Supplementary Fig. 5e–f). Furthermore, light did not markedly affect the respiratory state of anesthetized mice (Fig. 2i and Supplementary Fig. 5g). To further investigate whether optogenetic activation of TH + NTS neurons mediates the physiological function of swallowing, rather than simply eliciting nonspecific motor responses, we designed an experiment in which mice were induced to swallow a thin twine. Within 30 seconds of light activation (473 nm, 20 Hz, 10 ms, 2 mW) of the TH + NTS neurons, the mice exhibited a noticeable tendency to swallow the twine. In contrast, control mice did not display any swallowing behavior during light stimulation (Fig. 2j). A light pulse train (473 nm, 1 ms, 20 mW, 5, 10 and 20 Hz) reliably evoked phase-locked spiking activity from TH+ neurons expressing ChR2-mCherry (Supplementary Fig. 5h).

Secondly, we further investigated the sufficiency of TH + NTS neurons for swallowing function using chemogenetic methods. AAV-DIO-hM3Dq-mCherry or AAV-DIO-mCherry was injected into the NTS of *TH*-ires-Cre mice (Fig. 2k). During formal testing, saline solution was administered intraperitoneally as a control. The following day, at the same time, CNO was administered intraperitoneally while mylohyoid EMG was recorded. Compared to the control group, chemical activation of TH + NTS neurons dramatically rose the number of swallowing within 30 min and also correspondingly increased the length of swallowing twine (Fig. 2m). Immunostaining for TH in NTS sections revealed that the majority of TH+ cells (73.48% ± 4.33%, *n* = 6 mice) were positive for hM3Dq-mCherry, and the majority of hM3Dq-mCherry+ cells (83.92% ± 5.52%, *n* = 6 mice) were also positive for TH (Fig. 2l). Chemogenetic activation of TH neuron firing by CNO (10 μM) was confirmed through brain slice physiology (Supplementary Fig. 6a). These findings interpreted that the activation of TH + NTS neurons enhanced swallowing function in mice. The results above explicitly demonstrated that TH + NTS neurons were a key subtype involved in triggering swallowing behavior.

### TH+NTS neurons were necessary for swallowing behavior

**Fig. 3:**
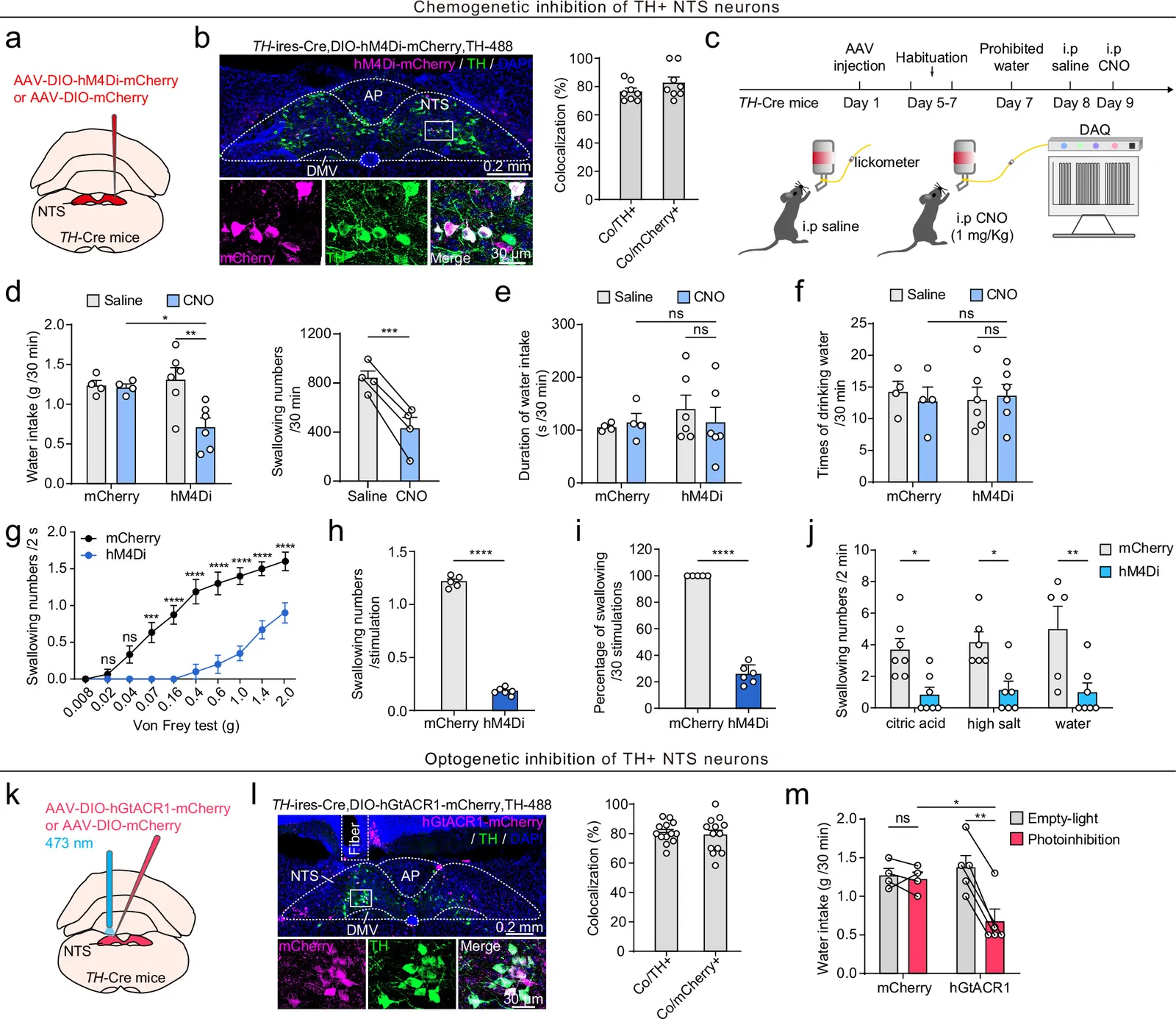
TH + NTS neurons were necessary for swallowing behavior. **a** Schematic diagram showing position of virus (DIO-hM4Di or DIO-mCherry) injection in the NTS of *TH*-ires-Cre mice. **b** Example coronal brain section displaying colocalization of hM4Di-mCherry+ neurons (magenta) with TH+ neurons (green) in the NTS (left), accompanied by statistical data on the co-localization of these neurons (right). **c** Schematic diagram illustrating the timeline (top) and procedure (bottom) for chemogenetic drinking water behavior of *TH*-ires-Cre mice. **d**–**f** Water intake (**d** left), swallowing numbers (**d** rihgt), duration of water intake (**e**) and times of drinking water (**f**) within 30 min in *TH*-ires-Cre mice following chemogenetic inhibition of TH + NTS neurons (mCherry: *n* = 4 mice, hM4Di: *n* = 6 mice). **g**–**i** Total number of swallowing (**g**) induced by mechanical stimulation of the pharynx in *TH*-ires-Cre mice with hM4Di-expressing NTS neurons (mCherry: *n* = 5 mice, hM4Di: *n* = 5 mice). Number (**h**) and percentage of swallowing behavior (**i**) induced by 0.4 g Von Frey within 30 min in *TH*-ires-Cre mice following chemogenetic inhibition of TH + NTS neurons (mCherry: *n* = 5 mice, hM4Di: *n* = 6 mice). **j** The number of swallowing caused by laryngeal perfusion with citric acid, high salt and water shortly afterwards chemogenetics inhibition of TH + NTS neurons (mCherry: citric acid: *n* = 7 mice, high salt: *n* = 6 mice, water: *n* = 5 mice; hM4Di: *n* = 7 mice) (Fig. S6 b). **k** Schematic diagram illustrating the sites of hGtACR1-mCherry injection and optical fiber implantation in the NTS of *TH*-ires-Cre mice. **l** Example micrographs showing hGtACR1-mCherry expression and the optical fiber track above these neurons in the NTS (left). Statistical data (right) showing colocalization of hGtACR1-mCherry+ (magenta) with the TH+ neurons (green) from the NTS (*n* = 5 mice). **m** Water intake of mice after optogenetic inhibition of TH + NTS neurons (mCherry: *n* = 4 mice, hGtACR1: *n* = 5 mice). Data were shown as mean ± s.e.m. (error bars). The broken white lines in the section images represent boundaries of brain regions. Statistical analysis in (**d**–**g**, **j** and **m)** were performed using Two-way ANOVA. Statistical analysis in (**d** right, **h** and **i)** were performed using Student* t*-tests (*P < 0.05, **P < 0.01, ***P < 0.001, ****P < 0.0001, ns ≥ 0.05). Source data are provided as a Source Data file.

Next, we assessed the impact of TH + NTS neurons on swallowing function in mice using a water drinking test. Swallowing can occur reflexively when substances accumulate in the pharynx, such as excess saliva, but it can also be voluntary, as during eating and drinking^46^. In *TH*-ires-Cre mice, AAV-DIO-hM4Di-mCherry or AAV-DIO-mCherry was expressed in the NTS (Fig. 3a). TH immunostaining of NTS sections revealed that the majority of TH-positive neurons (76.59% ± 2.28%, *n* = 8 mice) expressed hM4Di-mCherry, and most neurons expressing hM4Di-mCherry (82.80% ± 4.03%, *n* = 8 mice) were also positive for TH (Fig. 3b). Before the formal testing commenced, all mice were acclimated to drinking from stainless steel, pressure-bar water bottles for a period of three days (Fig. 3c). After inhibiting TH + NTS neurons, the water intake and swallowing numbers of mice was signally reduced within 30 min, suggesting a reduction in swallowing efficiency during natural drinking. However, there was no significant change in the times of drinking water or drinking duration, suggesting that inhibiting TH + NTS neurons had little effect on the mice’s motivation to drink water (Fig. 3d–f). This was consistent with the reported studies^35^. Furthermore, the chemical inhibition of TH + NTS neurons led to a substantial decrease in reflexive swallowing triggered by mechanical stimulation of the pharyngeal region (Fig. 3g–i). Previous studies revealed that chemical stimulation of the larynx can also induce reflexive swallowing response^10,11,13^. Chemical irrigation of the larynx with water, citric acid, and high-salt solution for 2 min significantly increased swallowing frequency compared to other solutions (Supplementary Fig. 6b, c). After inhibiting TH + NTS neurons, the number of reflexive swallowing induced by chemical stimulation (water, citric acid, and high-salt solution) of the laryngeal region was also visibly lessened (Fig. 3j). The chemogenetic inhibition of TH neuron firing by CNO (10 μM) was confirmed using brain slice physiology (Supplementary Fig. 6d).

Furthermore, we investigated the effect of optogenetic inhibition of TH + NTS neurons on mice’s free drinking behavior to further validate their physiological relevance. AAV-DIO-hGtACR1-mCherry or AAV-DIO-mCherry was injected into the NTS of *TH*-ires-Cre mice, and an optical fiber was implanted above it (Fig. 3k). The immunohistochemical staining of NTS sections showed that the majority of TH-positive neurons (80.80% ± 1.99%, *n* = 5 mice) expressed hGtACR1-mCherry, and a substantial number of neurons expressing hGtACR1-mCherry (78.96% ± 3.29%, *n* = 5 mice) were also positive for TH (Fig. 3l). Two to three weeks after viral expression, in a wakeful, free-drinking behavioral paradigm, blue light was delivered immediately upon initiation of drinking to inhibit TH+ neuron activity. Compared to the control period without light inhibition, optogenetic inhibition (continuous 473 nm blue light, 5 mW) of TH+ neurons significantly reduced mouse water intake in mice (Fig. 3m). These evidences precisely deciphered that TH + NTS neurons are essential for swallowing behavior in mice.

### Physiological properties of TH + NTS neurons

**Fig. 4:**
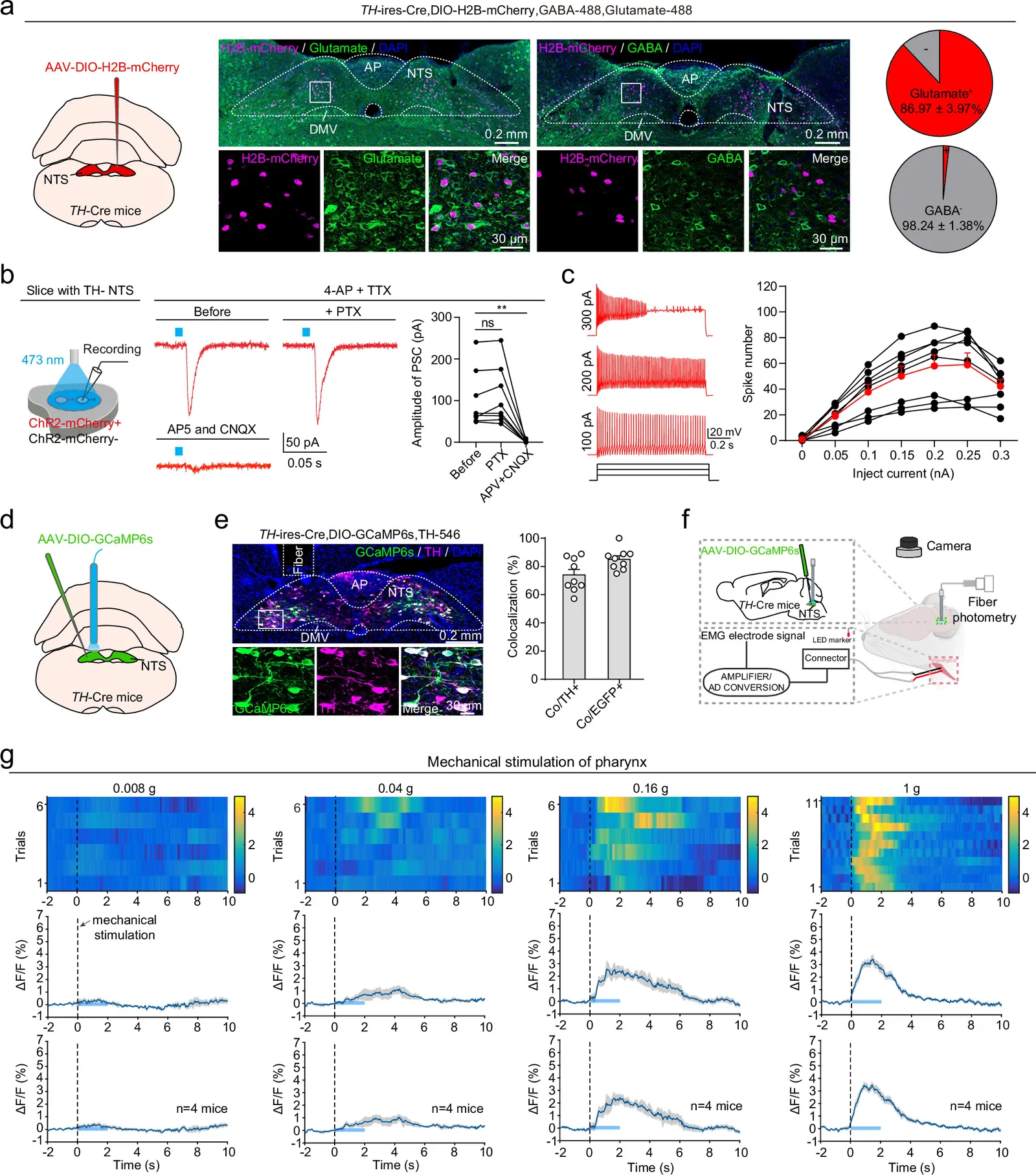
Physiological properties of TH + NTS neurons. **a** Left, schematic diagram showing the injection site of AAV-H2B-mCherry in NTS of *TH*-ires-Cre mice. Middle, micrographs showing immunostaining of TH vs. glutamate or TH vs. GABA in NTS. Right, quantitative analysis indicating overwhelming TH + NTS neurons are glutamate+ and GABA − . **b** Diagrammatic drawing showing recording of light-evoked (473 nm, 2 ms, 20 mW) PSCs from ChR2-mCherry negative neurons in acute slice with NTS; And the effects of PTX and D-AP5/CNQX on light-evoked PSCs (*n* = 8/17 cells). **c** Spike firings of TH+ neurons in the NTS to depolarizing currents (left); And quantitative analysis of spike number as a function of current intensity (right) (*n* = 8 cells). **d** Schematic diagram showing the injection site of AAV-DIO-GCaMP6s and optical fiber implantation in the NTS of *TH*-ires-Cre mice. **e** Example micrographs displaying the optical fiber track above the GCaMP+ NTS neurons (green), which were immunohistochemically verified to colocalize with TH+ neurons (magenta) (left). The fraction of colocalization of GCaMP+ and TH+ neurons was shown on the right (*n* = 9 mice). **f** Cartoon of fiber photometry and EMG recording. **g** Heatmap and average Ca2+ transients displaying 6-11 trials of normalized GCaMP fluorescence change of TH + NTS neurons. Calcium transients were elicited by mechanical stimulation of the pharynx (*n* = 4 mice). Blue thick lines indicate mean and gray shaded areas indicate s.e.m. The broken line represents the time points of the mechanical stimulation. The blue shading represents the time window for mechanical stimulation. Data were shown as mean ± s.e.m. (error bars). The broken white lines in the section images represent boundaries of brain regions. Statistical analysis in **b** was performed using One-way ANOVA (**P < 0.01, ns ≥ 0.05). Source data are provided as a Source Data file.

To examine the physiological properties of TH + NTS neurons, we first injected AAV-DIO-H2B-mCherry into the NTS of *TH*-ires-Cre mice. We then performed immunostaining by applying glutamate and GABA antibodies, respectively. The results showed that a large proportion of TH+ neurons (86.97% ± 3.97%, *n* = 8 mice) were glutamate + , while only a minority (1.76% ± 1.38%, *n* = 8 mice) were GABA+ (Fig. 4a). Subsequently, we identified the types of neurotransmitters released by the TH+ neurons. AAV-DIO-ChR2-mCherry was expressed in NTS neurons of *TH*-ires-Cre mice. In acute NTS brain slices, light pulses (473 nm, 2 ms, 20 mW) illuminating ChR2-mCherry-positive TH+ neurons elicited robust postsynaptic currents (PSCs) from ChR2-mCherry-negative NTS neurons (108 ± 24 pA, *n* = 8/17 neurons). These PSCs could be blocked with the perfusion of antagonists of glutamate receptors (APV and CNQX) but not GABAa receptors (picrotoxin, PTX) (Fig. 4b). These explorations indicated that light-evoked PSCs were mediated by glutamate receptors. Finally, after labeling TH+ neurons with AAV-DIO-EGFP in *TH*-ires-Cre mice, the EGFP-labeled TH+ neurons displayed spiking activity characterized by a slow adaptive pattern in response to depolarizing current injections (Fig. 4c).

Mechanical stimulation of the pharynx and water intake can both induce swallowing. Next, we explored how TH + NTS neurons encode mechanical sensory information during pharyngeal mechanical stimulation, as well as how they decode water signals during water-induced swallowing behavior. To monitor the activity of TH+ neurons during swallowing, we injected AAV-DIO-GCaMP6s into the NTS of *TH*-ires-Cre mice and implanted optical fibers (Fig. 4d). GCaMP-labeled neurons were predominantly distributed in the NTS, with most being TH+ (85.03% ± 2.49%, *n* = 9 mice) (Fig. 4e). First, we simultaneously recorded TH neuron dynamics, EMG signals of the mylohyoid, and pharyngeal pressure signals when the pharynx was subjected to mechanical stimulation. The intensity of Von Frey stimulation of the pharynx simulated the size of food boluses. During mechanical stimulation of the pharynx, calcium activity in TH neurons was markedly increased, particularly when 0.16–2 g stimuli were applied. The GCaMP signal of these neurons increased with the intensity of Von Frey stimulation (Fig. 4f, g and Supplementary Fig. 7a). Additionally, the calcium activity of these neurons also transiently rose when the larynx was stimulated by chemical agents (citric acid, high salt, and water) to induce swallowing (Supplementary Fig. 7c). Additionally, an apparent increase in GCaMP fluorescence was observed at the onset of water-induced swallowing, which was confirmed by synchronized EMG recordings of mylohyoid muscle activity (Supplementary Fig. 7b, movie 1). After swallowing was completed, calcium activity rapidly returned to baseline. This is consistent with the report by Wang et al. in 2024. During this period, we ensured that the mice had sufficient time to swallow the water. No obvious motion artifacts were observed during the swallowing process. In summary, TH + NTS neuron calcium activity was correlated with swallowing behavior.

### TH + NTS-Amb/RVLM pathway was the key circuit for triggering swallowing behavior

**Fig. 5:**
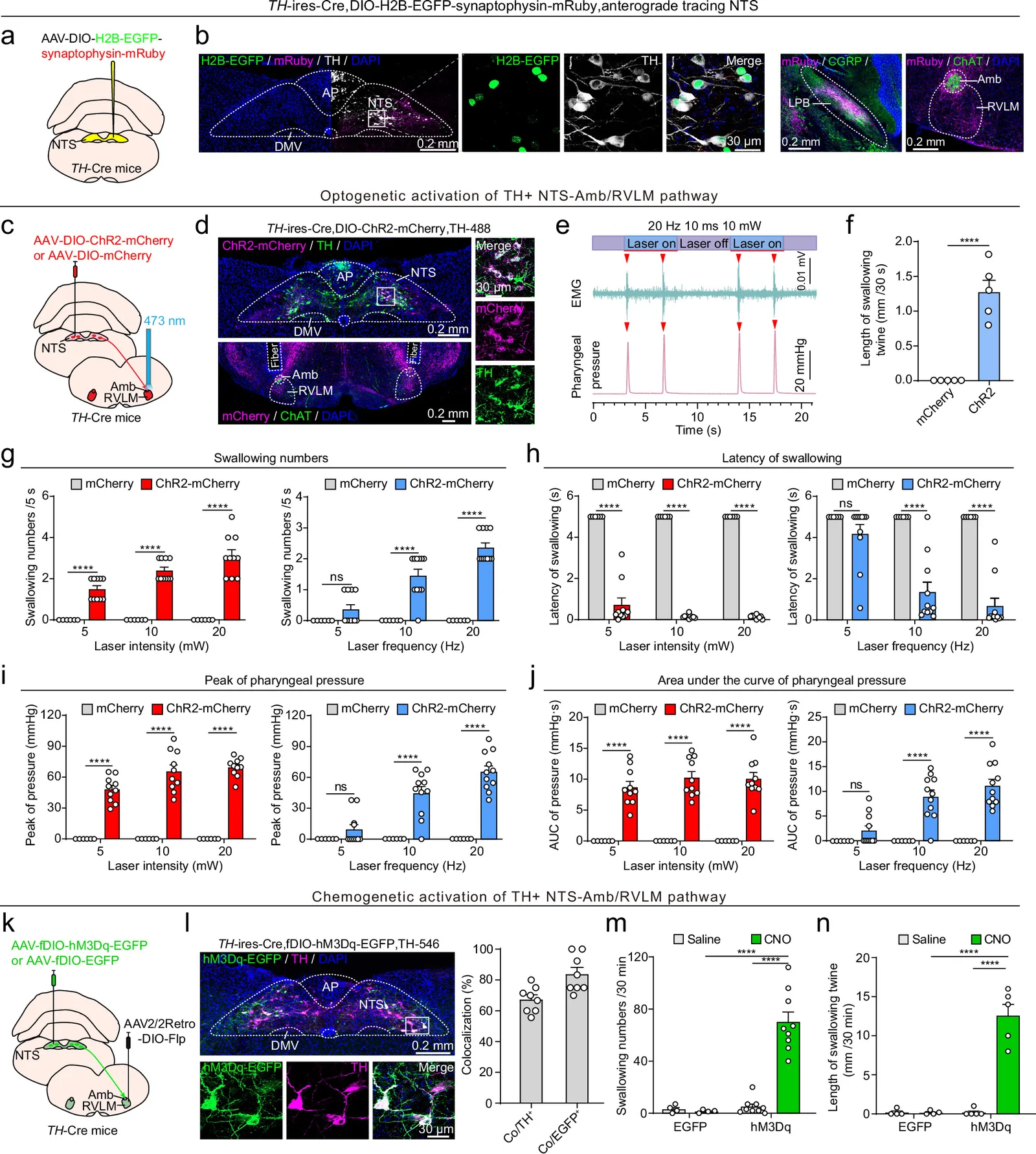
TH + NTS-Amb/RVLM pathway was the key circuit for triggering swallowing behavior. **a**,** b** Schematic diagram illustrating the injection of AAV-DIO-H2B-EGFP-synaptophysin-mRuby into the NTS of *TH*-ires-Cre mice (**a**), and example micrographs demonstrating the colocalization of H2B-EGFP+ neurons (green) with TH+ neurons (white) (**b** left), and mRuby+ axonal projections from TH + NTS neurons in the Amb/RVLM (ChAT marking) and LPB (CGRP marking) (**b** right) (see Fig. S8). **c**, **d** Schematic of optogenetic activation of the NTS-Amb/RVLM pathway (**c**). Example micrographs showing ChR2-mCherry expression in the NTS and the optical fiber track above the axon terminal in the Amb/RVLM, as well as the colocalization of ChR2-mCherry+ (magenta) with TH+ neurons (green) (**d**). **e** Representative images depicting EMG of the mylohyoid and pharyngeal pressure traces during photostimulation (blue shadow) of the NTS-Amb/RVLM pathway, inducing swallowing behavior (red triangles). **f** The length of swallowing twine during light-induced NTS-Amb/RVLM pathway within 30 s in *TH*-ires-Cre mice (mCherry: *n* = 5 mice, ChR2: *n* = 5 mice). **g**–**j** Quantitative analysis of swallowing with optogenetic activation of TH + NTS-Amb/RVLM pathway (**g**–**j** left: mCherry: *n* = 6 mice, ChR2: *n* = 10 mice; **g**–**j** right: mCherry: *n* = 6 mice, ChR2: *n* = 11 mice) (See Fig. S10). **k** Schematic of injection of AAV-fDIO-hM3Dq-EGFP into the NTS followed by AAV2/2Retro-DIO-Flp into the Amb/RVLM for chemogenetic activation of the NTS-Amb/RVLM pathway of *TH*-ires-Cre mice. **l** Example micrographs demonstrating the colocalization of Amb/RVLM-projecting hM3Dq-EGFP+ neurons (green) with TH+ neurons (magenta) in the NTS (left). Statistical data of colocalization of these neurons in the NTS was shown (right) (*n* = 8 mice). **m**, **n** Total swallowing number (**m**) and the length of swallowing twine (**n**) chemogenetically activated the NTS-Amb/RVLM pathway of *TH*-ires-Cre mice within 30 min (**m**: EGFP: *n* = 4 mice, hM3Dq: *n* = 9 mice; **n** EGFP: *n* = 4 mice, hM3Dq: *n* = 5 mice). Data were shown as mean ± s.e.m. (error bars). The broken white lines in the section images represent boundaries of brain regions. Statistical analysis in (**g**–**j** and **m**, **n**) was performed using Two-way ANOVA. Statistical analysis in **f** was performed using Student *t* tests (****P < 0.0001, ns ≥ 0.05). Source data are provided as a Source Data file.

As the key trigger of swallowing, which downstream nuclei does TH + NTS neurons project to in order to fine-tune swallowing behavior? To effectively solve this issue, AAV-DIO-H2B-EGFP-synaptophysin-mRuby was injected into the NTS of *TH*-ires-Cre mice for anterograde neural tracing (Fig. 5a). TH + NTS neurons diversely project to multiple brain regions, including the ambiguus nucleus (Amb)/ rostral ventrolateral medulla (RVLM) complex, lateral parabrachial nucleus (LPB), and arcuate hypothalamic nucleus (Arc) (Fig. 5b and Supplementary Fig. 8). LPB contains neurons that express calcitonin gene-related peptide (CGRP), which frequently act as a general alarm signal for various threats^47–49^. There are also reports in the literature that neurons within the LPB contribute to taste and feeding behavior, indicating the PBN’s involvement in swallowing^50^. The nucleus ambiguus (Amb) is a collection of motor neurons that project through the IX, X, and XI cranial nerves. The RVLM is connected to sympathetic activity and cardiovascular control. The ventral swallowing group (VSG) is located dorsal to the nucleus ambiguus or even medial to it in the reticular formation. In addition, it is evident that the Arc nucleus has direct or indirect inputs regulating the activity of hunger-sensitive neurons^51–53^, and its connection with the NTS mediates feeding pathways^34^. To evaluate the role of the TH + NTS-Arc, TH + NTS-LPB and TH + NTS-Amb/RVLM pathways in swallowing behavior, we injected AAV-DIO-ChR2-mCherry into the NTS of different *TH*-ires-Cre mice and implanted optical fibers above Arc, LPB and Amb/RVLM, respectively (Supplementary Fig. 9a). We observed that light activation (473 nm, 20 Hz, 10 ms, 2, 4 and 8 mW) of TH + NTS-Amb/RVLM pathway can reliably triggers swallowing behavior, and the number of swallowing was positively correlated with light intensity. However, under the same parameters, light stimulation of the TH + NTS-Arc and TH + NTS-LPB pathways rarely induced swallowing behavior (Supplementary Fig. 9b). This result illuminated that the TH + NTS-Amb/RVLM pathway was involved in swallowing behavior. So, are the Arc-projecting, LPB-projecting and Amb/RVLM-projecting neurons the same population of TH + NTS neurons? We applied retrograde tracing by injecting AAV2/2Retro-DIO-mCherry and AAV2/2Retro-DIO-EGFP into the Arc and Amb/RVLM of *TH*-ires-Cre mice, respectively (Supplementary Fig. 9c). Co-immunostaining of NTS brain slices for mCherry and EGFP revealed that TH + NTS neurons projecting to Arc and Amb/RVLM were largely different. Arc-projecting TH neurons were located medially in the NTS, whereas Amb/RVLM-projecting TH neurons were situated laterally in the NTS, with only 5% of these neurons being double labeled (Supplementary Fig. 9d, e). We observed that 22% of TH + NTS neurons projecting to the LPB and Amb/RVLM were double-labeled using the same strategy (Supplementary Fig. 9f–h). These results suggested that the Amb/RVLM complex was a key downstream nucleus of TH + NTS neurons involved in mediating swallowing.

To further investigate the effect of light stimulation on the TH + NTS-Amb/RVLM pathway and on swallowing behavior, AAV-DIO-ChR2-mCherry (test group) or AAV-DIO-mCherry (control group) was injected into the NTS of *TH*-ires-Cre mice, and optical fibers were embedded above the Amb/RVLM (Fig. 5c). Immunohistochemical staining of NTS sections for TH revealed that most ChR2-mCherry expressing neurons were TH-positive (Figs. 5d, 2c). Laser ON-OFF-ON paradigm (473 nm, 20 Hz, 10 ms, 10 mW, 5 s) reliably increased EMG and pharyngeal pressure (Fig. 5e and movie 2). The control mice showed no significant response. Notably, swallowing behavior induced by light activation of the TH + NTS-Amb/RVLM pathway exhibited power and frequency dependence. The number of swallowing in mice was positively correlated with laser power/frequency (Fig. 5g and Supplementary Fig. 10a). The latency of swallowing was negatively interrelated with it (Fig. 5h and Supplementary Fig. 10b). Correspondingly, the amplitude and AUC of pharyngeal pressure, which reflect swallowing strength, were also positively associated with laser intensity and frequency (Fig. 5i, j and Supplementary Fig. 10c, d). Notably, illumination of the TH + NTS-Amb/RVLM pathway triggered swallowing twine (Fig. 5f). Activation of TH + NTS-Amb/RVLM pathway did not overtly affect respiratory rate in mice (Supplementary Fig. 10e). In acute brain slices, light pulses (473 nm, 2 ms, 20 mW) illuminating ChR2-mCherry+ axon terminals of TH + NTS neurons evoked robust postsynaptic currents (PSCs) in Amb/RVLM neurons (62 ± 13 pA, *n* = 8 neurons). The light-evoked PSCs in Amb/RVLM neurons were mediated by glutamate receptors (Supplementary Fig. 10f).

Next, we utilized the Flp/fDIO system to chemogenetically activate the TH + NTS-Amb/RVLM pathway. AAV2/2Retro-DIO-Flp was injected into the Amb/RVLM, and simultaneously, either AAV-fDIO-hM3Dq-EGFP (experimental group) or AAV-fDIO-EGFP (control group) was injected into the NTS of *TH*-ires-Cre mice (Fig. 5k). The majority of hM3Dq-EGFP-expressing NTS neurons (83.83% ± 4.27%, *n* = 8 mice) were TH+ (Fig. 5l), certifying that the Flp/fDIO strategy for labeling Amb/RVLM- projecting NTS^TH^ neurons had acceptable specificity and efficiency. After treating with CNO, TH + NTS neurons projecting to the Amb/RVLM could be specifically activated. The number of swallowing and the length of swallowing twine in mice were memorably increased upon the activation of the TH + NTS-Amb/RVLM pathway (Fig. 5m, n). These findings strongly elucidated that the TH + NTS-Amb/RVLM pathway was a momentous circuit for triggering swallowing behavior.

### TH + NTS-Amb/RVLM pathway was shared downstream circuit for swallowing behaviors

**Fig. 6:**
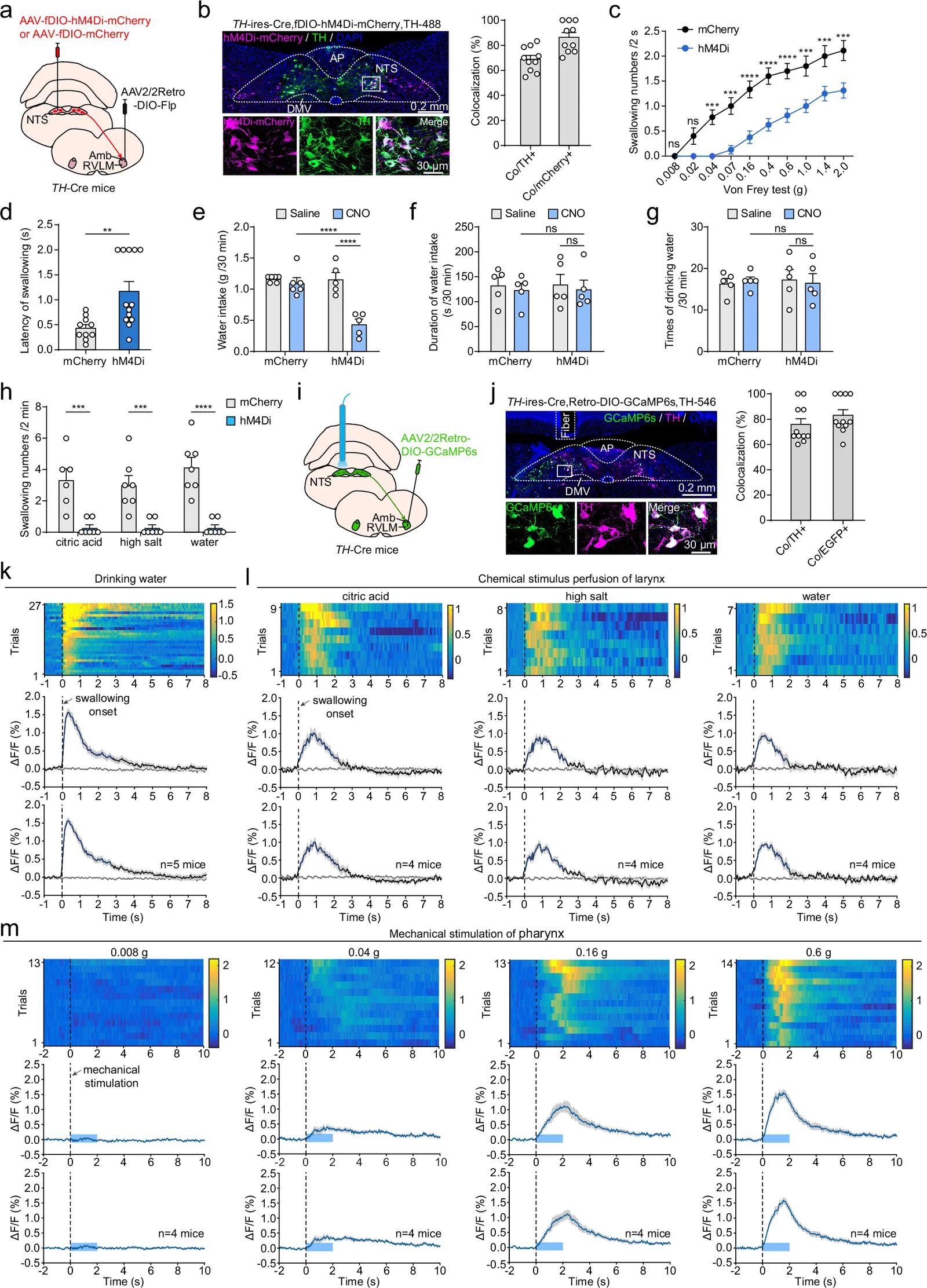
TH + NTS-Amb/RVLM pathway was shared downstream circuit for swallowing behaviors. **a** Schematic diagram of chemogenetic inhibition of the TH + NTS-Amb/RVLM pathway. **b** Micrographs showing colocalization of Amb/RVLM-projecting hM4Di-mCherry+ neurons with TH+ neurons in the NTS (left). Proportion of colocalization of these neurons was showing (right) (*n* = 10 mice). **c** Total number of swallowing induced by mechanical stimulation of the pharynx chemogenetically inhibition the TH + NTS-Amb/RVLM pathway (mCherry: *n* = 7 mice, hM4Di: *n* = 7 mice). **d** Latency of swallowing triggered by mechanical stimulation of the pharynx with 0.4 g von frey. **e**–**g** Water intake (**e**), duration of water intake (**f**) and times of drinking water (**g**) within 30 min following chemogenetic inhibition of the TH + NTS-Amb/RVLM pathway (**e**: mCherry: *n* = 6 mice, hM4Di: *n* = 5 mice; **f**, **g**: mCherry: *n* = 5 mice, hM4Di: *n* = 5 mice). **h** The number of swallowing caused by laryngeal perfusion shortly afterwards chemogenetics inhibition of TH + NTS-Amb/RVLM pathway (mCherry: citric acid: *n* = 6 mice, high salt and water: *n* = 7 mice; hM4Di: *n* = 7 mice). **i** Schematic showing the injection site of AAV2/2Retro-DIO-GCaMP6s into the Amb/RVLM and optical fiber implantation in the NTS of *TH*-ires-Cre mice. **j** Micrographs displaying the optical fiber track above the GCaMP+ NTS neurons projecting to the Amb/RVLM (green) (left). The fraction of colocalization of GCaMP+ NTS neurons with TH+ neurons (magenta) was shown on the right (*n* = 11 mice). **k** Ca2+ signals (ΔF/F) from Amb/RVLM-projecting TH + NTS neurons synchronized with water-triggered swallowing behavior (*n* = 5 mice). Thick lines indicate mean and shaded areas indicate s.e.m. Blue segments indicate Ca2+ signal exceeds above 15% from baseline to peak. The horizontal gray line represents motion interference recorded by channel 405. **l** Ca2+ signals from Amb/RVLM-projecting TH + NTS neurons that synchronizes with swallowing behavior induced by chemical stimulation of the larynx (*n* = 4 mice). The broken line represents the time points of swallowing. The horizontal gray line represents the control group. **m** Ca2+ signals from Amb/RVLM-projecting TH + NTS neurons that synchronizes with swallowing behavior elicited by mechanical stimulation of the pharynx (*n* = 4 mice). Blue thick lines indicate mean and gray shaded areas indicate s.e.m. The broken line represents the time points of the mechanical stimulation. The blue shading represents the time window for mechanical stimulation (See Fig. S12). Statistical analysis in (**c**, **e**–**h)** were performed using Two-way ANOVA. Statistical analysis in **d** was performed using Student *t* tests (**P < 0.01, ***P < 0.001, ****P < 0.0001, ns ≥ 0.05). Source data are provided as a Source Data file.

Could TH + NTS-Amb/RVLM circuit inclusively receive sensory input from mechanical and chemical stimuli to induce swallowing behaviors? Firstly, we injected AAV2/2Retro-DIO-Flp into the Amb/RVLM of *TH*-ires-Cre mice, while injecting AAV-fDIO-hM4Di-mCherry (test group) or AAV-fDIO-mCherry (control group) into the NTS (Fig. 6a). Those Amb/RVLM-projecting hM4Di-mCherry+ NTS neuron were mostly (86.40% ± 3.69%, *n* = 10 mice) TH+ (Fig. 6b). Amb/RVLM-projecting NTS^TH^ neurons were specifically inhibited with CNO. Acute chemogenetic inhibition of the TH + NTS-Amb/RVLM pathway dramatically reduced the number of reflexive swallowing induced by mechanical stimulation of the pharynx (Fig. 6c). And latency of swallowing elicited by 0.4 g Von Frey filament significantly prolonged after inhibiting this pathway (Fig. 6d). In the drinking water experiment, chemogenetic inhibition of the TH + NTS-Amb/RVLM pathway significantly decreased water intake of mice within 30 min, with no manifest changes observed in the times or the duration of drinking water (Fig. 6e–g). The number of swallowing also patently mitigated when the larynx was perfused with chemical stimulants (citric acid, high-salt solution and water) for 2 min (Fig. 6h). The data elaborated that the TH + NTS-Amb/RVLM pathway was essential for responses to both mechanically and chemically elicited swallowing behavior.

More than that, we utilized optogenetic inhibition approaches to investigate the effect of inhibiting the specific projections from the NTS to the Amb/RVLM on drinking behavior in mice. AAV-DIO-GtACR1-mCherry or AAV-DIO-mCherry was injected into the NTS, and optical fiber were implanted above bilateral Amb/RVLM region of *TH*-ires-Cre mice (Supplementary Fig. 11a, b). Two to three weeks after viral expression, in an awake, free-drinking behavioral paradigm, blue light illumination was immediately administered when mice initiated drinking. Results showed that optogenetic inhibition (continuous 473 nm blue light, 5 mW) axon terminals of TH + NTS neurons projecting to Amb/RVLM significantly reduced water intake in mice (Supplementary Fig. 11c). Although optogenetic inhibition with GtACR1 has been widely used for neuronal silencing, it has been suggested that direct illumination of axon terminals can cause off-target effects by inhibiting passing fibers. Subsequently, we further used the NpHR optogenetic silencing tool to manipulate the mouse swallowing experiment. The results similarly showed that optogenetic inhibition of TH + NTS terminals in the Amb/RVLM nucleus significantly reduced water intake (Supplementary Fig. 11d–f). These data fully demonstrated the necessity of the TH + NTS-RVLM pathway for swallowing function.

Secondly, to more definitively characterize the response of the TH + NTS-Amb/RVLM pathway to mechanical and chemical stimuli, we monitored Ca2+ signals changes in Amb/RVLM-projecting NTS^TH^ neurons under these conditions. In *TH*-ires-Cre mice, AAV2/2Retro-DIO-GCaMP6s was injected into the Amb/RVLM and fiber optics was embedded above the NTS (Fig. 6i). The neurons that were retrogradely labeled and expressed GCaMP6s through Amb/RVLM injection were predominantly (83.16% ± 4.29%, *n* = 11 mice) positive for TH. (Fig. 6j). The GCaMP signals of Amb/RVLM-projecting NTS^TH^ neurons significantly and rapidly increased at the initiation of water-induced swallowing under a fixed head condition (Fig. 6k). When the larynx was subjected to chemical stimulation with citric acid, high-salt and water solutions to induce swallowing behavior, the GCaMP signals of Amb/RVLM-projecting NTS^TH^ neurons also markedly risen (Fig. 6l), but their responses extent to all three stimuli were roughly consistent. GCaMP signaling in Amb/RVLM-projecting NTS^TH^ neurons also showed marked variation while mechanically stimulated the pharynx, and it gradually increased following the intensity of mechanical stimulation (Fig. 6m and Supplementary Fig. 12). Moreover, GCaMP signals in response to mechanical stimuli (ranging from 0.16 g to 2 g) were generally higher than those in response to chemical stimuli. The above data adequately elaborated that the TH + NTS-Amb/RVLM pathway was a shared downstream pathway for both mechanical and chemical stimuli-induced swallowing behavior.

Finally, we verified the regulation of synaptic release within the RVLM. TH is the rate-limiting enzyme in the synthesis of dopamine (DA), which is further converted into norepinephrine (NE). To monitor the release of DA or NE in the Amb/RVLM, a genetically encoded GPCR-activated GRAB-DA sensor or GRAB-NE sensor was employed to measure DA/NE levels. Specifically, AAV-DIO-C1V1-mCherry (test) or AAV-DIO-mCherry (ctrl) was injected into the NTS of *TH*-ires-Cre mice, and an optical fiber was embedded above it for optogenetic activation. Meanwhile, AAV-GRAB-DA2h or AAV-GRAB-NE2m was injected into the Amb/RVLM, and an optical fiber embedded above it to monitor neurotransmitter signals (Supplementary Fig. 13a, d and f). Two to Three weeks after viral expression, the NTS was optically activated while synaptic release within the Amb/RVLM was recorded. The results showed that optogenetic activation of the NTS (589 nm, 20 Hz, 10 ms, 2 mW, 2 s) triggered a significant increase in dopamine signals within the Amb/RVLM, rather than norepinephrine signals. In the control group, no obvious fluorescent changes were observed in Amb/RVLM neurons upon optogenetic stimulation of the NTS (Supplementary Fig. 13b and e). Furthermore, treatment with the DA antagonist haloperidol (Haldol) eliminated light-induced GRAB-DA signals (Supplementary Fig. 13c). These findings confirmed that activating the TH + NTS neurons triggered the release of DA in the Amb/RVLM.

Optogenetics and chemogenetics, combined with neuronal dynamics results, have sufficiently confirmed that the TH + NTS-Amb/RVLM pathway was a shared downstream pathway for pharyngeal mechanical stimulation and laryngeal chemical stimulation to induce swallowing reflex. This pathway was crucial for swallowing behavior and regulated dopamine release within the RVLM.

### Vagal ganglion Piezo2 sensed mechanical stimulation of the pharynx and mediated swallowing behavior

In this study, the pharynx served as the direct site for mechanical stimulation. The cell bodies of vagal sensory neurons are located in the vagal ganglia, and their axons bifurcate into two branches: one innervates visceral organs, while the other projects to the brainstem. The pharynx is densely innervated by the vagus nerve^36^. Moreover, afferent fibers of the vagus nerve from the pharynx, as well as SLN, terminate in the NTS^1,36^. To determine the anatomical connections between the vagal ganglia and the pharynx, herpes simplex virus (HSV-EGFP) was injected into the pharyngeal mucosa of *WT* mice as an anterograde transsynaptic tracer. The distribution of EGFP+ neurons was subsequently observed in the vagal ganglia and NTS (Supplementary Fig. 14a, b). Additionally, the mylohyoid muscle is regulated by CPG and is activated first during the pharyngeal phase of swallowing^1^. To trace the histological connections between the brainstem CPG and the mylohyoid muscle, pseudorabies virus (PRV-mRFP) was injected into the mylohyoid as a retrograde transsynaptic neural tracer. The virus retrogradely transports to the somata of central neurons, where active infection occurs. mRFP+ neurons originating from the mylohyoid was observed in the NTS and Amb/RVLM complex (Supplementary Fig. 14c–f). These results provided ample evidence for the anatomical connectivity of the pharyngeal-vagal ganglia-NTS-Amb/RVLM pathway.

**Fig. 7:**
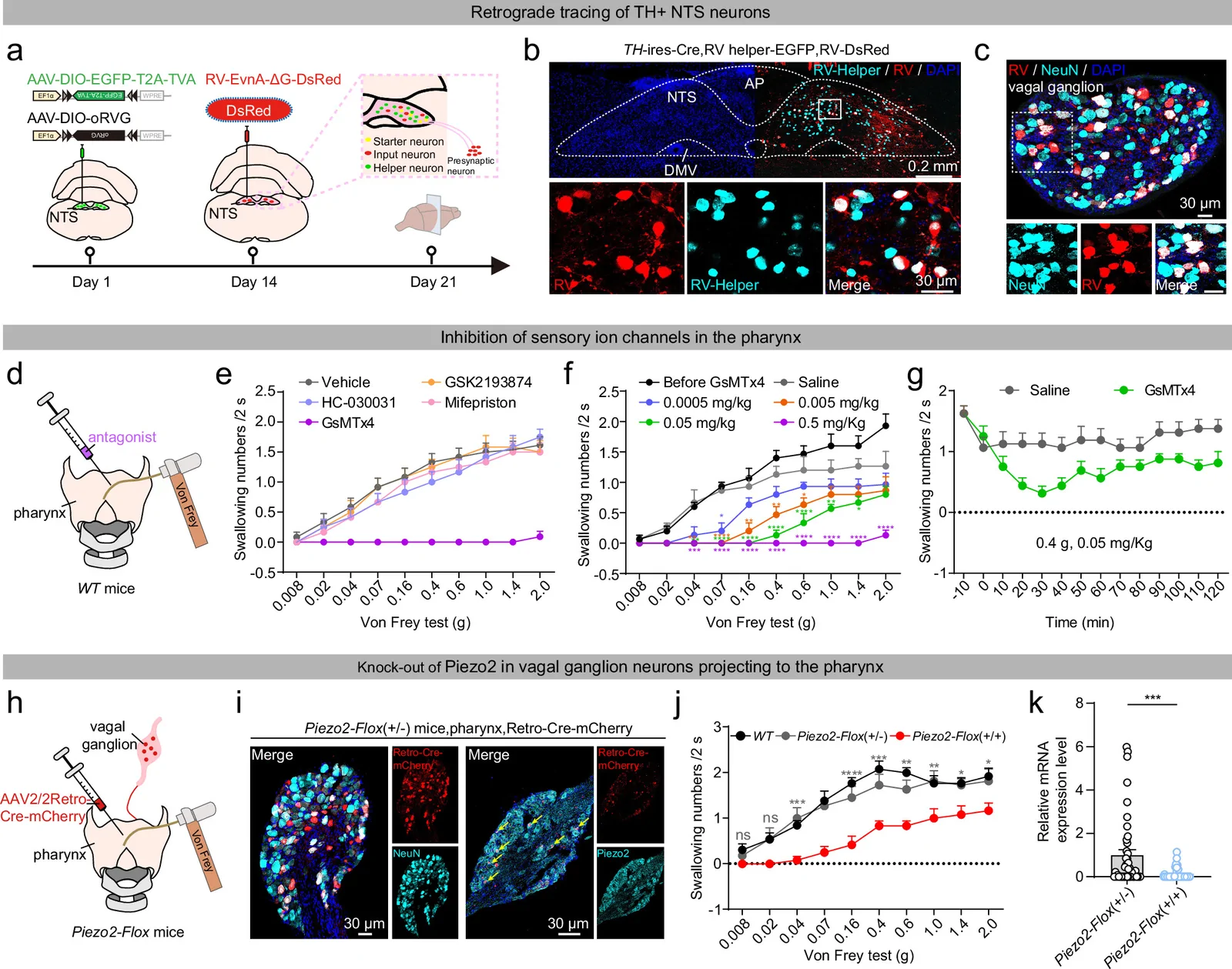
Vagal ganglion Piezo2 sensed mechanical stimulation of the pharynx and mediated swallowing behavior. **a** Schematic diagram illustrating the strategy for monosynaptic retrograde tracing of TH + NTS neurons using a combination of AAV and RV. **b** Example brain section showing the dually labeled starter cells in the NTS of *TH*-ires-Cre mice. **c** Example micrographs displaying the colocalization of DsRed+ cells with NeuN in vagal ganglion. Scale bar is 30 μm. **d** Schematic diagram of injection antagonist into pharynx in *WT* mice. **e** Total number of swallowing triggered by mechanical stimulation of the pharynx with Von Frey filaments of *WT* mice antagonizing the corresponding sensory ion channels in the pharynx (GsMTx4, 0.5 mg/Kg; GSK2193874, 1 mg/Kg; HC-030031, 0.05 mg/Kg; Mifepristone, RU-486, 1 mg/Kg; each group: *n* = 4 mice). **f**,** g** The statistical graph showing the variation of the number of swallowing induced by mechanical stimulation, as a function of dose of the Piezo antagonist GsMTx4 in mice (each dose group: *n* = 5 mice) (**f**), and the number of swallowing elicited by 0.4 g Von Frey within two h (saline: *n* = 4 mice, GsMTx4: *n* = 4 mice, 0.05 mg/Kg) (**g**). **h** Schematic diagram of injection AAV2/2Retro-Cre-mCherry into pharynx of *Piezo2-Flox* mice. **i** Representative images of colocalization of retrogradely labeled Cre-mCherry+ (red) with NeuN+ neurons (cyan) (left), and Cre-mCherry+ with Piezo2+ neurons (cyan) (right) in vagal ganglion in *Piezo2-Flox*(+/-) mice. **j** Total number of swallowing elicited by mechanical stimulation in *Piezo2-Flox* mice with knock-out of of Piezo2 in vagus ganglion neurons projecting to the pharynx (*WT*: *n* = 4 mice, *Piezo2-Flox*(+/-): *n* = 4 mice, *Piezo2-Flox*(+/+): *n* = 4 mice). **k** Relative mRNA expression level of Piezo2 in vagal ganglion neurons projecting to the pharynx by single-cell qPCR (*Piezo2-Flox*(+/-) group: *n* = 43 cells, *Piezo2-Flox*(+/+) group: *n* = 43 cells). Data were shown as mean ± s.e.m. (error bars). The broken white lines in the section images represent boundaries of brain regions. Statistical analysis in (**e**, **f**, **j)** were performed using Two-way ANOVA. Statistical analysis in **g** was performed using One-way ANOVA. Statistical analysis in **k** was performed using Student *t*-tests (*P < 0.05, **P < 0.01, ***P < 0.001, ****P < 0.0001, ns ≥ 0.05). Source data are provided as a Source Data file.

Finally, we aim to identify the mechanoreceptors in vagal sensory neurons that detect pharyngeal mechanical stimuli, and to investigate how these mechanoreceptors transmit mechanical sensory signals to TH + NTS neurons. We adopted rabies virus (RV) vector tracing to identify retrogradely labeled vagal ganglion neurons. Injection RV-helper AAVs into the NTS of *TH*-ires-Cre mice, followed by RV-DsRed injection two weeks later (Fig. 7a, b). The retro-labeled neurons were primarily located in the M1/M2, S1/S2, and vagal ganglion (Fig. 7c and Supplementary Fig. 15a, b). Most RV-labeled cells in vagal ganglion monosynaptically projecting to the TH + NTS neurons were Piezo2-positive (Supplementary fig.15c). We harvested RV-traced vagal ganglion neurons and performed SMART-seq2 sequencing (Supplementary Fig. 15c, d). We then calculated the signature score between all the 31 clusters of vagus sensory neurons and RV-traced neurons according to the signature gene expression similarity. The RV-traced neurons were similar to those in clusters 2, 4, 14, 18, 23 and 31, which contain receptors mediating swallowing induced by chemical and mechanical stimulation (Supplementary Fig. 15e–g).

Next, we used corresponding antagonists—Piezo family mechanosensitive ion channel antagonist GsMTx4, TRPA1 antagonist HC-030031, TRPV4 antagonist GSK2193874, and TRPM3 antagonist mifepristone—to respectively inhibit the pharyngeal sensory ion channels. We found that suppressing the Piezo family ion channels observably inhibited mechanostimulus-induced swallowing reflex, whereas the inhibition of other ion channels had not notably affected this behavior (Fig. 7d, e and Supplementary Fig. 16a–c). Furthermore, the latency of swallowing induced by mechanical stimulation with 0.4 g Von Frey filament was dramatically extend after antagonizing pharyngeal Piezo family ion channel, compared with the control group (Supplementary Fig. 16d). Our data also indicated that mechanical stimulation-induced swallowing reflexes exhibited a dose-dependent effect of GsMTx4 (Fig. 7f). Concurrently, the latency of swallowing triggered by 0.4 g Von Frey filament also exhibited a dose-dependent prolongation after inhibition of pharyngeal Piezo ion channel (Supplementary Fig. 16e). Local inhibition of the pharyngeal Piezo ion channel with 0.05 mg/kg GsMTx4 progressively decreased the number of swallowing triggered by mechanical stimulation with 0.4 g Von Frey, reaching a minimum at 30 min. As the drug was metabolized, the inhibitory effect on the Piezo ion channel diminished, and the swallowing elicited by mechanical stimulation gradually returned to its baseline levels (Fig. 7g). Swallowing at the 0-min mark showed a rapid decline compared to the −10-min time point, likely due to acute surgical injury. Pharmacological results demonstrated that mechanically stimulating the pharynx to induce swallowing-like behavior was mechanotransduction-dependent.

Vagal ganglion neurons primarily express the Piezo2 mechanosensitive ion channel. Piezo2 is the principal mechanotransducer in low-threshold cutaneous mechanoreceptors and skeletal-muscle-innervating proprioceptors in mice^54–57^. It can also sense tracheal stretch^58^ and blood pressure^59^. Based on the function of Piezo2 as a mechanotransducer and its abundance in various populations of sensory neurons, combined with the aforementioned SMART-seq2 sequencing results, we focused on studying Piezo2-mediated swallowing behavior. We subsequently employed the Cre-loxP strategy to conditionally knock out Piezo2 in vagal ganglion neurons that project to the pharynx by injecting AAV2/2Retro-Cre-mCherry into the pharyngeal mucosa of *Piezo2-Flox*(+/+) mice (Fig. 7h). Utilize *Piezo2-Flox*(+/−) mice and *WT* mice were used as simultaneous controls. Immunostaining of the vagal ganglia in *Piezo2-Flox*(+/-) mice demonstrated the distribution of mCherry+ neurons retrogradely labeled from the pharynx, among which 59.75% were Piezo2+ (Fig. 7i and Supplementary Fig. 16f). Our results expounded that the knockout of Piezo2 in vagal ganglion neurons that project to the pharynx dramatically reduced swallowing induced by mechanical stimulation ranging from 0.04 g to 2 g (Fig. 7j). Concurrently, after Piezo2 knockout, the latency for triggering swallowing was markedly prolonged under stimulation with a 0.4 g Von Frey filament (Supplementary Fig. 16 g).

To validate the effective knockout of Piezo2, we collected mCherry-positive vagal ganglion neurons for single-cell qPCR detection. The relative mRNA expression levels of mRNA indicated that the knock-out efficiency of Piezo2 was ~89% (Fig. 7k). The qPCR quantification results declared that Piezo2 was effectively knocked out in vagal neurons projecting to the pharynx. Although AAV of the Retro serotype is primarily taken up by axon terminals at the injection site and retrogradely transported to the cell nucleus for expression, a small amount of expression can also occur locally at the injection site. To exclude the influence of AAV2/2Retro expression in pharyngeal cells, we injected AAV-Cre-mCherry into the pharynx of *Piezo2-Flox*(+/+) mice (Supplementary Fig. 16h). After knockout of Piezo2 in pharyngeal cells, mechanical stimulation-evoked swallowing remained unaffected (Supplementary Fig. 16i). These evidences adequately clarified that Piezo2 mechanosensitive ion channel in vagal neurons was the mechanosensor mediating swallowing reflexes evoked by mechanical stimulation. It detected mechanical stimuli from the pharynx and transmitted these mechanical sensory signals to the swallowing center NTS, thereby mediating the initiation of swallowing.

### Piezo2 + SLN bundles were the key afferent fibers mediating swallowing

**Fig. 8:**
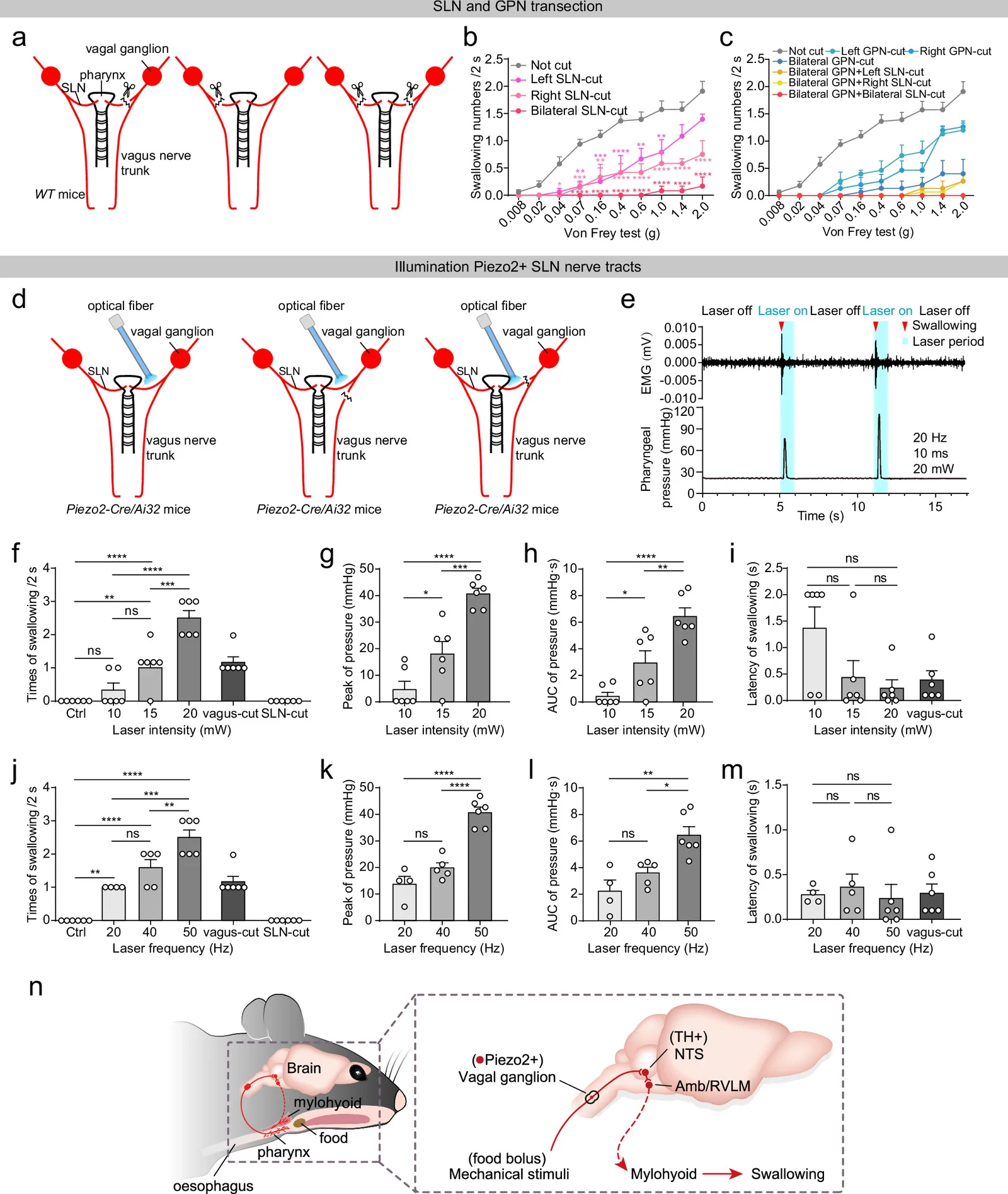
Piezo2 + SLN bundles were the key afferent fibers mediating swallowing. **a**,** b** Cartoon of the transection of the superior laryngeal nerve (SLN) in *WT* mice (**a**). Total number of swallowing behaviors induced by mechanical stimulation of cutting the left, right and bilateral SLN in *WT* mice, respectively (Not cut: *n* = 11 mice; else each group: *n* = 4 mice) (**b**). **c** Total number of swallowing induced by mechanical stimulation of cutting the left, right and bilateral glossopharyngeal nerve (GPN), bilateral GPN combined with SLN in *WT* mice, respectively (Not cut: *n* = 11 mice; else each group: *n* = 4 mice). **d** Schematic diagram of optogenetic stimulation of the SLN in *Piezo2-Cre/Ai32* mice. The middle and right diagrams illustrating the illumination of the SLN following the transection of the vagus nerve trunk and the SLN. **e** Representative images depicting EMG of the mylohyoid and pharyngeal pressure traces of swallowing (red triangles) evoked by SLN illumination (blue shadow) in *Piezo2-Cre/Ai32 mice*. **f**–**i** Swallowing numbers via illuminating SLN in *Piezo2-Cre/Ai32* mice (*n* = 6 mice) (**f**); And quantitative analysis of the amplitude (**g**), AUC (**h**) of pharyngeal pressure and latency of swallowing (**i**). **j**–**m** Swallowing numbers via activating SLN with light in *Piezo2-Cre/Ai32* mice (*n* = 4 mice) (**j**); And quantitative analysis of the amplitude (**k**) and AUC (**l**) of pharyngeal pressure rand latency of swallowing (**m**). In **f**–**m** 10 ms, 50 Hz, 20 mW was used to illuminate the SLN after cutting the SLN and the vagus nerve trunk. Among them, the control group was *Piezo2-Cre*(-/-)/*Ai32*(+/+) mice subjected to light stimulation with equivalent parameters. **n** Schematic diagram depicting brain circuits for mechanical stimuli triggered swallowing-like behavior in mice. Data were shown as mean ± s.e.m. (error bars). Statistical analysis in (**b**, **c)** were performed using Two-way ANOVA. Statistical analysis in **f**–**m** were performed using One-way ANOVA (*P < 0.05, **P < 0.01, ***P < 0.001, ****P < 0.0001, ns ≥ 0.05). Source data are provided as a Source Data file.

Neurons within the DSG receive monosynaptic or oligosynaptic inputs from sensory afferent fibers of the SLN and GPN^1,24,25,60^. Stimulation of SLN and GPN can trigger protective upper airway reflexes, with swallowing being the most common protective behavior^16,17,19^. To clarify the relative roles of SLN afferent bundles and the potential contribution of the GPN in mechanically induced swallowing, we conducted systematic nerve transection experiments. The experimental treatment group included: (1) left SLN transection; (2) right SLN transection; (3) bilateral SLN transection; (4) left GPN transection; (5) right GPN transection; (6) bilateral GPN transection; (7) bilateral GPN transection combined with left SLN transection; (8) bilateral GPN transection combined with right SLN transection; (9) bilateral transection of both the GPN and SLN. Results revealed that compared with no SLN transection, both unilateral and bilateral transection of SLN significantly impaired swallowing reflex evoked by mechanical stimulation of the pharynx, moreover, swallowing reflex was nearly abolished after bilateral SLN transection (Fig. 8a, b). Furthermore, the latency of swallowing elicited by 0.4 g Von Frey filament was obviously prolonged after SLN injury. However, no difference in swallowing latency was observed between left or right SLN transection (Supplementary Fig. 16j). Additionally, unilateral and bilateral transection of GPN both caused varying degrees of damage to mechanically stimulated reflexive swallowing. After transection of bilateral GPN combined with unilateral SLN (left or right side), reflexive swallowing could be induced by high-threshold mechanical stimulation intensity, but was otherwise almost impossible to elicit. Bilateral GPN and bilateral SLN transection completely eliminated the swallowing reflex evoked by pharyngeal mechanical stimulation. Overall, the swallowing triggered by mechanical stimulation decreased progressively after isolated GPN transection and after combined GPN and SLN transection. In other words, the degree of impairment of mechanically stimulus-induced reflexive swallowing increased gradually with unilateral (left or right) GPN transection, bilateral GPN transection, bilateral GPN combined with unilateral (left or right) SLN transection, and bilateral GPN combined with bilateral SLN transection (Fig. 8c). Notably, mice with bilateral SLN damage or combined damage to GPN and SLN were unable to survive long-term. Integrating the results of SLN and GPN transection or combined damage on mechanically stimulated swallowing manifested that SLN play critical role in pharyngeal mechanical sensory afferents.

We crossed *Ai32* mice (carrying Cre-dependent channelrhodopsin-2 allele) with *Piezo2-Cre* mice, which label mechanosensory neurons and SLN bundles. Optogenetic stimulation was achieved in offspring by focal illumination particular nerve branches. Illumination of SLN in *Piezo2-Cre/Ai32* mice (20 Hz, 10 ms, 20 mW, 1 s) can induce swallowing (Fig. 8d, e). In control mice lacking Cre, light stimulation of SLN failed to evoke swallowing-like behavior. Additionally, the number and strength of swallowing, reflected by the amplitude and AUC of pharyngeal pressure, increased with higher laser power and frequency (Fig. 8f–h, j–l). The swallowing latency was basically unaffected by light (Fig. 8i, m). Furthermore, following the implementation of the SLN transection, the illumination of the SLN did not provoke swallowing. Given the impact of light on the vagus nerve, even after severing the vagus nerve trunk, illumination of the SLN (at 20 Hz, 10 ms, 20 mW, for 1 second) could still elicit a swallowing response. These evidences strongly expounded that Piezo2+ SLN bundles were the key afferent fibers mediating swallowing.

In summary, our work confirmed that TH + NTS neurons mediate swallowing-like behavior induced by mechanical stimulation of the pharynx and chemical stimulation of the larynx, highlighting the necessity of the TH + NTS-Amb/RVLM neural circuit in driving swallowing behavior. Additionally, we identified the crucial role of Piezo2 ion channel in vagal sensory neurons in transducing swallowing signals from mechanical stimuli of food bolus. Overall, we have delineated a mechanosensory pathway that is crucial for reflexive swallowing evoked by pharyngeal stimulation under anesthesia. We summarized the main research findings and presented the neural mechanisms of this study in a comic format (Fig. 8n).

## Discussion

Swallowing plays a crucial role not only in the intake of food and water but also in defense mechanisms to prevent the inhalation of foreign objects. Understanding how sensory information is converted into functional outputs is key to deciphering the mechanisms of the sensory system. The NTS serves as the primary relay station for transmitting visceral sensory signals to the brain and is essential for initiating these responses by activating the central pattern generator (swallowing CPG) within the brainstem neural network. Although its role in feeding regulation has been explored, how mechanical sensory-related signals are processed during pharyngeal mechanical stimulation remains unclear. Our research elucidated the critical pathways of Piezo2 channels in vagal sensory neurons and of SLN nerve bundles in controlling the swallowing behavior under mechanical stimulation, and further explored the significance of the TH + NTS-Amb/RVLM circuit in the swallowing reflex driven by both mechanical and chemical stimuli, providing a certain experimental basis for improving dysphagia.

### The role of PIEZO2 mechanosensitive channels in mediating swallowing under mechanical stimulation

It is well known that reflexive swallowing is primarily caused by specific areas of the pharynx and larynx. Sensory zones eliciting reflexive swallowing were analyzed under mechanical and chemical stimuli (such as water, acid, saliva, etc.)^13^. In the pharyngeal region, mechanical stimulation effectively elicits swallowing. The current methods reported for inducing the swallowing reflex mechanically primarily involve food boluses and glass needles; however, the force and threshold of these stimuli are not quantifiable. In our work, we utilized a set of Von Frey filaments to apply standardized forces, thereby triggering the afferent fibers of Piezo2 mechanosensitive neurons. This systematic approach revealed the primary regulatory mechanisms by which mechanical stimulation of the mouse pharynx induces the swallowing reflex. This was consistent with previous findings that *C. elegans* PIEZO channel triggers rhythmical movement of the pharynx^61^, and this PIEZO channel was functionally conserved with neuronally expressed PIEZO2 in mouse, which demonstrated the evolutionary conservation of this protein in swallowing control. Piezo2 is ideally suited to mediate mechanosensation in swallowing due to its low activation threshold, which allows it to respond to innocuous pharyngeal touch, and its rapid activation kinetics, which ensure minimal delay in triggering the reflexive motor output.

### Regulation of swallowing by higher cognitive centers

Swallowing is directly controlled by brainstem circuits, such as the NTS-Amb/RVLM, whereas higher cognitive centers, including the cerebral cortex and limbic system, achieve fine control, contextual adaptation, and emotional regulation of swallowing through top-down modulation. Cortical regions, such as the sensory-motor cortex, parietal-temporal cortex, insular cortex, and cingulate cortex, primarily initiate and coordinate swallowing actions upon receiving afferent input. Meanwhile, subcortical structures, including the basal ganglia and thalamus, regulate and control the motor aspects of swallowing through the cortico-basal ganglia-thalamo-cortical circuit^4,28^. Our study identified TH neurons in the NTS as key nodes in the modulation of swallowing by higher cognitive centers. These neurons participate in the regulation of swallowing and related dysphagic disorders and hold promise as therapeutic targets.

### Investigation of the same peripheral afferent population induced by chemical and mechanical stimulation for swallowing

Our current research focus reveals that TH + NTS neurons and their downstream pathway—specifically, the TH + NTS-Amb/RVLM projection—serve as a shared pathway for both mechanically and chemically evoked swallowing. It is crucial to clarify whether chemical and mechanical stimuli converge onto TH + NTS neurons via the same upstream afferent population for establishing the existence of a unified shared circuit. Recording the responses of matched stimulated sites (e.g., applying chemical stimulation to the pharynx) and/or directly recording the reactions of upstream afferent populations will be one of the most direct experiments to verify the convergence mechanism. However, chemically stimulating the pharynx presents significant technical challenges. Due to the close anatomical proximity of the pharynx and larynx in mice, the current method of delivering chemical stimuli via laryngeal perfusion cannot reliably restrict stimulation to the pharynx without involving the larynx. We acknowledge this technical limitation and are actively exploring and refining more precise local stimulation approaches. Furthermore, the vagal ganglion primarily contains sensory neurons, which are responsible for receiving and transmitting sensory information from the periphery to the central nervous system. Therefore, it is also very important whether the upstream afferent population exhibits convergence. The convergence characteristics of upstream afferents represent an important issue that needs to be addressed in the future. We plan to make this a central focus of follow-up studies, aiming to provide a more comprehensive understanding of the complete circuit mechanisms from peripheral receptors to central integration.

### The role of Piezo2 + SLN fiber bundle in swallowing reflex

Numerous studies have demonstrated that the SLN converge on the vagal ganglia, and neurons within the DSG receive monosynaptic or oligosynaptic input from SLN sensory afferent neurons. In our study, transection of the SLN abolished reflexive swallowing triggered by pharyngeal mechanical stimulation. Importantly, optogenetic activation of Piezo2+ SLN fibers reliably induced swallowing-like behavior. This result appears to be inconsistent with the 2020 Cell paper by Prescott et al., and possible factors may lie in differences in experimental parameters (such as light stimulus intensity and duration). The optogenetic activation parameters used in the two studies are not exactly the same. Prescott et al. used the following parameters for SLN activation: 473 nm, 20 Hz, 5 ms pulses. We employed stimulation at higher frequencies and with different pulse widths (473 nm, 20, 40, 50 Hz, 10 ms pulses).

### The same neuronal population functions across multiple channels

Although the regulatory mechanisms of swallowing have been extensively studied, the neural circuits controlling swallowing may be more complex than those previously proposed. Swallowing may perform different functions in response to various stimuli and the physical properties of food, and the same neuronal subtype may play a role in different phases of swallowing or feeding. Research reports that NTS^TH^ → PVN^MC4R^ encodes feeding motivation equivalent to overnight fasting, transmitting peripheral hunger signals to the melanocortin pathway^33^. NTS^TH^ axons innervate the arcuate nucleus (Arc) and induce food intake^34^. The swallowing process is divided into three phases, including the oral phase, pharyngeal phase, and esophageal phase. Wang et al. discovered in 2024 that TH+ cNTS neurons transmit via vagal afferents to encode esophageal mechanical distension and instantaneous swallow volume, providing rapid feedback regulation of ingestive velocity^35^. In contrast, our study indicated that TH + NTS neurons mediated the pharyngeal swallowing reflex triggered by mechanical stimulation of the pharynx. TH + NTS neurons encode pharyngeal mechanical stimulus sensory information perceived and transmitted by Piezo2 ion channels in vagal ganglion neurons, and induce swallowing-like behavior in anesthetized mice through the TH + NTS-Amb/RVLM pathway. We have identified a pharynx-to-brain mechanical sensory pathway that is crucial for pharyngeal reflexive swallowing induced by pharyngeal stimulation under anesthesia. Our experimental data are consistent with the observation by Wang, 2024 that TH + NTS neurons are involved in the perception of mechanical stimulation in the pharynx.

Moreover, it is precisely during the oral phase of swallowing that, after mastication, food boluses are propelled into the pharynx by the tongue and other structures, leading to esophageal mechanical distension. This distension is perceived by TH + NTS neurons, which then regulate esophageal motility and trigger the swallowing reflex. Inactivation of these neurons impairs the perception of mechanical stimulation by food boluses entering the pharynx, even if it does not affect mastication and propulsion of oral-phase food into the pharynx. Although the bolus entering the pharynx produces mechanical stimulation to the pharynx, the pharyngeal phase of swallowing cannot proceed, leading to dysphagia. Furthermore, the intensity of Von Frey stimulation of the pharynx simulated the size of food boluses, as evidenced by the TH + NTS neurons dynamics recorded, which is consistent with the conclusion of Wang, 2024. The drinking experiment also confirmed that TH + NTS neurons are unrelated to drinking motivation, which is consistent with the phenomenon observed by Wang in 2024. In general, TH + NTS neurons encode information such as hunger motivation and mechanical sensation, exhibiting differences in sensory patterns, associated visceral organs, and ascending sensory pathways to precisely coordinate different aspects of swallowing or feeding regulation.

### The functional coordination of swallowing and breathing

Research on mechanical stimulation induced swallowing aids in elucidating the neural basis of swallowing-respiration coordination, including the functional hypothesis that the Amb/RVLM regulates swallowing and respiration inhibition. Amb is generally represented as a small cluster of large cholinergic motor neurons which participating in essential reflexes such as swallowing, the gag reflex and heart function^3,62^. While RVLM catecholaminergic neurons regulate breathing^63^. Alternatively, the axons of TH + NTS neurons are located in the brainstem respiratory control circuit (Bötzinger complex and pre-Bötzinger complex) and are used to coordinate breathing with swallowing. We hypothesize that Chat+ Amb motor neurons may be involved in swallowing movements, whereas RVLM catecholaminergic neurons play a role in transient respiration arrest during swallowing. Related validation experiments are also being carried out.

In summary, our study systematically elucidated the neural mechanisms underlying mechanical stimulation-induced swallowing behavior in mice by investigating the complete pathway of peripheral mechanical stimulation - Piezo2 sensory afference - NTS-Amb/RVLM central integration—swallowing behavioral output.

## Methods

### Animals

The *FosCreER* (*Fos-2A-iCreER*), *TH*-ires-Cre, *Calb1*−2A-Cre, *Dbh*-Cre, *Tac1*-ires-Cre, *Piezo2-Flox*, *Ai32*, *Piezo2*-ires-Cre and *C57BL/6 J* mouse lines were imported from the Jackson Laboratory (JAX Mice and Services). *Synpr*-ires-Cre mice were constructed by Shanghai Southern Model Biotechnology Co., LTD. *Piezo2-Cre/Ai32* mice were generated by crossing *Piezo2-ires-Cre* mice with *Ai32* mice in Shang Congping’s laboratory. The mouse lines used in this study are listed in Supplementary Table 1. Except for Fig. 1h, which utilized both female and male *C57BL/6 J* mice, all other animal experiments employed 8-week-old male mice. Animals were maintained under controlled environmental conditions (23 ± 1 °C, 50 ± 5% relative humidity) with a 12 h light–dark cycle and ad libitum access to food and water. They were initially group-housed (3–5 per cage) and were separated into individual cages three days prior to viral injection. Following surgery, each mouse was housed singly for a two-week recovery period before subsequent experiments. All CreER mice and control littermates received tamoxifen at least 14 days before further experiments. All animal husbandry and experimental procedures were conducted in accordance with protocols approved by the Administrative Panel on Laboratory Animal Care at School of Basic Medical Sciences, The Fifth Affiliated Hospital, Guangzhou National Laboratory, Guangzhou Medical University (Guangzhou, China).

### Swallowing-like model

Avertin (125 mg/kg) anesthetized adult mice were placed in a supine position under a stereoscopic microscope, with their body temperature maintained at 37 °C using a heating pad. The depth of anesthesia was assessed by evaluating withdrawal reflexes, blinking, and jaw tone. Prior to initiating the stimulation protocol, anesthetic depth was confirmed, and then no additional doses were administered during the experiment. The apparatus was cleaned several times with ethanol (75%) to prevent infection in the mice. A longitudinal midline incision was made in the neck, and the mice were intubated to maintain normal breathing. The pharynx was surgically opened from its ventral side, exposing the throat, epiglottis, and pharyngeal mucosa. The pharyngeal mucosa was then mechanically stimulated with Von Frey fibers for 2 s to induce the swallowing reflex. Swallowing behavior in mice was identified by pharyngeal pressure, EMG activity of the mylohyoid muscle, and digital microscope (RWD Life Science, China, DOM-1001) observations of laryngeal elevation. Swallowing is defined as pharyngeal pressure exceeding the baseline level by 10 mmHg. Both male and female mice between 8 and 12 weeks old were used for all studies, and no differences based on sex were observed.

### Physiological measurements

Pharyngeal pressure was measured by a differential pressure transducer; mylohyoid muscle contraction was characterized by electromyographic recording. The anesthetized mice had their cervical ventral hair shaved and were quickly secured in the supine position. A latex balloon (0.03 mL, VK, 73-3478) connected to PE tubing (with diameter of 1*0.5 mm) was placed near the base of the tongue and the hard palate, allowing breathing space. Pharyngeal pressure was monitored by transoral insertion of a pressure transducer (NL108T4) coupled to the bioelectric module amplifier (Digitimer, NL905) and data acquisition system (1401 Digitizer). A double-strand tungsten microelectrode (A-M Systems, 795500) with the insulating layer removed from the tip (~0.2 mm) was inserted into the right mylohyoid muscle via a syringe needle as a guide. The depth was ~5 mm. This side of the microelectrode was fixed with surgical sutures. The other side of the microelectrode passed subcutaneously and exited at the top of the scalp near the connector used for EMG recordings, and was connected to the busbar using solder. The busbar was fixed to the top of the head and connected to the recording system through a sub-busbar. Finally, the skin was sutured. The EMG signals were recorded using a Microelectrode AC Amplifier Model 1800 (A-M Systems), filtered (100–5000 Hz) and digitized at 5000 Hz using Spike2 software (Cambridge Electronic Design). Swallowing reflex was identified by mylohyoid muscle contraction, increases in pharyngeal pressure and laryngeal elevation.

For swallowing behavior induced by 1) oral water administration in awake mice (Supplementary Fig. 7b and Fig. 6k), 2) swallowing behavior induced by laryngeal chemical stimulation (Figs. 3j, 6h, l, Supplementary Fig. 6c, and Supplementary Fig. 7c), and 3) the swallowing twine experiment (Figs. 2j, m right, 5f, n), the measurement metrics were mylohyoid muscle electromyography (EMG) recordings combined with video-monitored laryngeal elevation. For all other experiments, swallowing behavior was assessed using a combined metric system consisting of mylohyoid muscle EMG recordings, pharyngeal pressure measurements, and video-monitored laryngeal elevation.

### Records of animal respiration

Anesthetized mice were placed in a supine position. Respiration was monitored using a JZ100 force transducer (Beijing Xinhang Xingye Science and Technology Trade Co., Ltd., China). Specifically, a suture was passed through the skin of the thoracic wall and attached to the elastic cantilever of the transducer, which was gently tightened to capture chest wall movements. Thoracic expansion and contraction during respiration deformed the cantilever, and the transducer converted this mechanical signal into an electrical signal, which was then recorded by a data acquisition (DAQ) system. During optogenetic experiments, the DAQ was synchronized with the output of the optical stimulator to ensure precise timing between stimulation and recording. Signals were digitized at a sampling rate of 100 Hz and acquired using Fiber Photometry software. For analysis, we manually counted the total respiratory wave peaks within three consecutive 5 s epochs: the 5 s period immediately before light stimulation, the 5 s period immediately after the onset of light stimulation, and the 5 s period immediately after the stimulation ended.

### c-Fos activation strategy

To eliminate the interference of environmental factors on c-Fos activation, the entire experiment was conducted in a quiet environment devoid of strong light. Mechanical stimulation was applied to the pharynx of anesthetized *WT* mice using 0.4 g Von Frey filament, with a total stimulation duration of 60 min. The stimulation paradigm consisted of a 2-second stimulation delivered every minute. For control mice, only the pharynx was exposed without any mechanical stimulation. At 90 min after the end of stimulation, the mice were perfused and their brains were harvested. Subsequently, coronal sections of the whole brain were prepared, and c-Fos immunofluorescence staining was performed to localize the neural nuclei associated with swallowing behavior. The expression level of c-Fos in the NTS was quantified via manual counting by an experimenter who was blinded to the experimental grouping.

### FosTRAP strategies

AAV2/9-hEF1α-DIO-hChR2(H134R)-mCherry-WPRE-pA was unilaterally injected into the NTS of *FosCreER* (*Fos-2A-iCreER*) mice on day 1. On day 5, the swallowing model was established using 0.4 g Von Frey filaments to stimulate the pharyngeal mucosa of mice. The entire process spanned one h, during which a 2-second mechanical stimulation was delivered every minute. Subsequently, 4-hydroxytamoxifen (12.5 mg/kg) was intraperitoneally injected into the *FosCreER* mice (once every 24 h for 3 consecutive days). 4-Hydroxytamoxifen was dissolved in a mixture of sunflower oil and corn oil to prepare a 20 mg/mL stock solution. The control group mice were only exposed to the pharynx without being given mechanical stimulation. On Day 14–21 (1–2 weeks after swallowing-TRAP), swallowing behavior evoked by photoactivation of the NTS was recorded, as previously described. Coronal sections were collected against immunohistochemistry for mCherry and c-Fos. Before perfusion, the pharyngeal mucosa must be stimulated once more for 1 h with 0.4 g of Von Frey filament.

### Stereotaxic viral injection

We used two AAV serotypes (AAV2/9, AAV2/2Retro) in this study. The AAVs used in this study are listed in Supplementary Table 4. The viral particles were purchased from Shanghai TaiTool Bioscience Inc. and Brain VTA Inc. The titers of viral vectors were initially in the range of 1.0 – 1.9 × 10^13^ particles/mL. The final titer used for AAV injection after dilution with PBS was 2 × 10^12^ viral particles/mL or 6 × 10^12^ viral particles/mL.

Mice were anesthetized and placed on a stereotaxic frame (RWD Life Science, China) and received standard surgery that exposed the brain surface above the NTS (bregma −7.48 mm, lateral +0.25 mm and dura −3.40 mm), Amb/RVLM (bregma −6.85 mm, lateral ±1.55 mm, dura –4.35 mm and 4° from the lateral to medial), Arc (bregma −1.46 mm, lateral +0.25 mm and dura −5.65 mm). AAVs were stereotactically infused via a glass micropipette coupled to a Nanoliter Injector 201 (World Precision Instruments) at a rate of 0.1 μL/min to minimize local tissue injury. The pipette was left in place for at least 20 min after injection before being slowly withdrawn.

For the injection into the mouse pharynx, anesthetized adult mice were positioned under a stereoscopic microscope. The pharyngeal mucosa was fully exposed, and 10 μL of AAVs (6 × 10^12^ viral particles/mL) were injected directly into it at four sites using a 2.5 μL microsyringe (Hamilton Company, Inc.) with a beveled 34-gauge needle. The needle was left at each injection site for 5 min to avoid spilling.

### Optic fiber implantation

A ceramic ferrule with an attached optical fiber was implanted 20 min after viral injection, with the fiber tip positioned above the NTS (bregma −7.48 mm, lateral +0.25 mm, and dura −3.20 mm), Amb/RVLM (bregma −6.85 mm, lateral ±1.55 mm, dura −4.15 mm, 4° from the lateral to medial), LPB (bregma −5.20 mm, lateral ±1.50 mm, dura −2.50 mm). For optogenetic recordings, fiber of 200 µm (N.A. = 0.22) diameter was used. The ferrule was secured to the skull using dental cement, the incision was closed with sutures, and topical antibiotics were applied to prevent infection. All photostimulation experiments were initiated at least two weeks after the implantation procedure.

### Optogenetics

Animals were anesthetized with Avertin (125 mg/kg) unless otherwise specified. For brain tissue optogenetic manipulations, the output of the laser was measured and adjusted to 1–20 mW before each experiment. Light pulse parameters—onset, duration, and frequency—were programmed via a pulse generator coupled to the laser system. The laser power and frequency of light pulses were adjusted to measure the dependency of the swallowing behavior in test mice expressing AAV2/9-hEF1α-DIO-hChR2(H134R)-mCherry-WPRE-pA (test group) or AAV2/9-hEF1α-DIO-mCherry-WPRE-hGH-pA (control group) on various parameters of light stimulation.

For superior laryngeal nerve (SLN) optogenetics, the left SLN of *Piezo2-Cre/Ai32* mice was surgically exposed. Fiber end attached to a manipulator was positioned for focal illumination of the SLN, and stimulus parameters (20–50 Hz, 10 msec pulses, 10–20 mW, 2 s) were controlled by a shutter system as described above. The light-evoked swallowing behavior was quantified by visual observation, measuring the number of peaks formed by pharyngeal pressure and EMG activity in mice.

### Animal drinking water detection

For chemogenetic inhibition, AAV2/9-hSyn-DIO-hM4D(Gi)-mCherry-WPRE-pA or AAV2/9-hSyn-DIO-mCherry-WPRE-pA was unilaterally injected into the NTS of *TH*-ires-Cre mice. Two weeks after AAV injection, the ability of mice to drink water was tested. Before the official test, all mice were conditioned to drink water from a bottle containing a stainless steel ball and pressure rod over the course of three consecutive days. All mice were trained and formally tested after 12 h of water deprivation with normal feeding. The mice were given 30 min of free access to water in their home cage each day. Then each test mouse received an intraperitoneal injection CNO (1 mg/kg) or saline (as a control) to allow for gradual inhibition of hM4Di-mCherry-expressing NTS neurons. After 20 min, the mouse was tested for drinking behavior and behavior was continuously recorded for 30 min. Drinking behavior was recorded using a multimodal synchronous system. Specifically, a water bottle equipped with a stainless steel ball and a pressure-sensing rod was connected to a 1401 digital-to-analog converter via a lickometer module. All licking signals were acquired in real time by Spike2 software, with simultaneous video recording (50 frames/s; Point Gray Research, Canada). Water consumption was calculated by measuring the weight difference of the water bottle before and after the experiment. During data analysis, a continuous and regular cluster of signals recorded by Spike2 was defined as one drinking event, with its duration measured from the first signal to the last signal. There should be at least a 3-second interval between two independent drinking events. The number of drinking events was counted as the total number of such independent signal clusters, and the total drinking duration was calculated as the cumulative sum of the durations of all events. The effectiveness of CNO to suppress action potential firing from hM4Di+ NTS neurons was validated with slice physiology. No such suppression was observed in control mice.

For optogenetic inhibition drinking experiment, AAV2/9-hSyn-DIO-hGtACR1-mCherry-WPRE-pA or AAV2/2-hSyn-DIO-eNpHR3.0-EYFP-WPRE-pA was injected into the NTS of *TH*-ires-Cre mice, and an optical fiber was implanted above the unilateral NTS or bilateral Amb/RVLM. 2–3 weeks after viral expression, all animals first drank water for 30 min under non-light stimulation conditions. On the next day, during the same awake, free-drinking paradigm, continuous 473 nm blue light or 589 nm yellow light was delivered upon drinking onset to inhibit TH + NTS neurons or axon terminals of TH + NTS neurons projecting to Amb/RVLM. A 3-day adaptive drinking training was conducted before the formal test. Water consumption was calculated by measuring the weight difference of the water bottle before and after the experiment.

### Detection of animal swallowing twine

We designed swallowing twine experiment in mice. Similarly, the NTS of *TH*-ires-Cre mice expressed AAV2/9-hSyn-DIO-hM3D(Gq)-mCherry-WPRE-pA as test group or AAV2/9-hSyn-DIO-mCherry-WPRE-pA as control group. Twine consists of two strands, with a diameter of ~0.3 mm, treated with professional lubrication line oil to prevent irritation. During the formal test, mice received an intraperitoneal injection of normal saline on the first day, and CNO (1 mg/kg) was administered on the second day, allowing the CNO to gradually activate the NTS neurons that express hM3Dq-mCherry. The twine, marked at 1 mm intervals, was inserted into the esophagus using a gavage needle (No. 8). The swallowing behavior was identified through visual observation using a digital microscope and EMG recording over a duration of 30 min. Finally, the effectiveness of CNO in eliciting action potential firing in NTS hM3Dq positive neurons was validated through slice physiology. The behavioral tests were performed in the same circadian period (1 PM–7 PM). All behaviors were scored by the experimenters who were blind to the animal treatments.

### Oral catheter implantation

The metal cannula (outer diameter 0.5 mm) was pre-bent at a specific angle ~one-third of its length from the tip and connected to a silicone tube (~5 cm in length, outer diameter 0.54 mm). The metal cannula was vertically fixed to the surface of the mouse skull using dental cement. A ~ 5 mm incision was made in the skin on the right side of the face. The sterilized silicone tube was passed subcutaneously into the oral cavity and secured in place with a 7-0 nylon suture on the oral side. Approximately 3 mm of the silicone tube was retained inside the oral cavity, and after trimming to an appropriate length, the skin incision was sutured closed.

### Calcium imaging

Calcium signal from genetically identified neurons was recorded using a commercialized fiber photometry system (Thinker Tech Nanjing Biotech CO., Ltd)^45,64^. Following the stereotaxic injection of AAV2/9-CAG-DIO-GCaMP6s-WPRE-hGH-pA (test virus) or AAV2/9-EF1α-DIO-EGFP-WPRE-hGH-pA (control virus) into the NTS of *TH*-ires-Cre mice, the optical fiber (Fiblaser Technology Co., Ltd) was implanted above the NTS. Excitation light (470 nm) from a laser diode was coupled into the optical path via a dichroic mirror, focused through a 10× objective (N.A. = 0.3), and directed into a rotary fiber-optic joint. A 3-m patch cord (200 µm core, N.A. = 0.37) transmitted the light between the commutator and the implanted fiber. The output power at the fiber tip was set to 0.02 mW to limit photobleaching. Emitted GCaMP6s fluorescence was separated by a bandpass filter (MF525-39, Thorlabs) and detected with a photomultiplier tube (R3896, Hamamatsu). The PMT current was converted to voltage by an amplifier (C7319, Hamamatsu) and subsequently low-pass filtered (40 Hz cutoff; Brownlee 440). The fluorescence signal, indicative of the change in strength, was transmitted back to the detection optical path via the same optical fiber, and the image was captured by CMOS camera. Analog voltage signals were digitized at 100 Hz and acquired using a Power 1401 interface and Spike2 software (CED, Cambridge, UK).

After two weeks of viral injection, a headplate was affixed to the skull, and a tube was implanted into the oral cavity. To minimize motion artifacts, mice were placed on a head-fixed roller to adapt them to the roller for 30 min daily, starting 3 days prior to the formal experiment. During recording, a long silicone tube was used to connect the fixed metal cannula to a syringe needle mounted on a microinjection pump. The entire tubing system was filled with liquid beforehand. A volume of 20 µL of distilled water was infused into the oral cavity at a constant rate of 10 µL/s. All infusions successfully elicited swallowing behavior, with a latency of less than 10 s from the start of infusion to the onset of swallowing. Each mouse received 2–3 independent infusions. During this period, the animals were given sufficient time to swallow the water. Neuronal calcium signaling, the myoelectric activity of the mylohyoid muscle and mouse behavior were monitored. As a control, water was administered to mice injected with a control virus. A 3-s light-pulse-triggered square-wave signal was recorded simultaneously with video, electromyography (EMG), and GCaMP fluorescence signals to enable temporal alignment of the multimodal data. The onset of swallowing was defined as the starting point of the characteristic burst discharge in the EMG trace. Normalized GCaMP fluorescence (ΔF/F) was utilized to measure the GCaMP signal, and the data were aligned with swallowing onset. Subsequent to the experiments, the optical fiber tip sites in each mouse were examined histologically.

### Monitoring of neurotransmitters

Neurotransmitter signals were recorded using a commercialized fiber photometry system (Thinker Tech Nanjing Biotech CO., Ltd). Specifically, AAV2/9-EF1α-DIO-C1V1-mCherry-WPRE-hGH-pA (test) or AAV2/9-DIO-mCherry (ctrl) was injected into the NTS of *TH*-ires-cre mice, and an optical fiber was implanted above it for optogenetic activation. Meanwhile, AAV2/9-hSyn-GRABeen-DA2h-WPRE-pA or AAV2/9-hSyn-GRAB-NE2m-WPRE-pA was injected into the Amb/RVLM, with an optical fiber embedded above to monitor neurotransmitter signals. 2–3 weeks after viral expression, the NTS was optically activated (589 nm, 20 Hz, 10 ms, 2 mW, 2 s) by a programmable pulse generator while synaptic release within the Amb/RVLM was recorded by fiber photometry system. The programmable pulse generator was connected to the fiber photometry system to facilitate the temporal alignment of the multimodal data. Mice were treated with haloperidol can inhibit the release of dopamine. Haloperidol (dopamine antagonist, Selleck S1920) was dissolved in 1% DMSO to prepare stock solutions at 0.8 mg/mL. The dose for intraperitoneal injection was 0.4 mg/Kg and 1.2 mg/kg.

### Cell-type-specific tracing

For cell-type-specific anterograde tracing of TH + NTS neurons, AAV2/9-EF1α-DIO-H2B-EGFP-T2A-Synaptophysin-mRuby-WPRE-pA was stereotaxically injected into the NTS of *TH*-ires-Cre mice. Animals recovered from surgery and were sacrificed 3 weeks later for histology.

For multi-level anterograde tracing from the pharyngeal mucosa, anesthetized adult mice were positioned beneath a stereoscopic microscope. The pharyngeal mucosa was fully exposed, and 10 μL of HSV-EGFP (2 × 10^9^ PFU/mL) was injected directly into it at four sites using a 2.5 μL microsyringe (Hamilton Company, Inc.) with a beveled 34-gauge needle. The needle was left at each injection site for 5 min to avoid spillage. Three days after injection, pharyngeal tissue and brain tissue were subjected to immunofluorescence staining.

For multi-level retrograde labeling from mylohyoid muscle, after adequate exposure of the mylohyoid, 3 μL of PRV-CAG-mRFP (2 × 10^9^ PFU/mL) was injected at two sites (1.5 μL for each site) across the mylohyoid using a glass capillary. The pipette was kept at each injection site for 5 min to prevent overflow. Immunofluorescence staining was conducted on the fifth day post-injection.

For cell-type-specific RV tracing, the modified RV-based three-virus system was used for retrograde tracing. All the viruses included AAV2/9-EF1α-DIO-H2B-EGFP-T2A-TVA (5 × 10^12^ viral particles/mL), AAV2/9-EF1α-DIO-oRVG (5 × 10^12^ viral particles/mL), and EnvA-pseudotyped, glycoprotein (RG)-deleted and DsRed-expressing RV (RV-EnvA-ΔG-DsRed) (5 × 10^8^ viral particles/mL). For mapping the whole-brain inputs to TH + NTS neurons, a mixture of AAV2/9-EF1α-DIO-EGFP-T2A-TVA and AAV2/9-EF1α-DIO-oRVG (1:1) was unilaterally stereotaxically injected into the NTS of *TH*-ires-Cre mice. Two weeks after AAV helper injection, RV-EnvA-ΔG-DsRed was injected into the same location in the NTS of *TH*-ires-Cre mice in a biosafety level-2 lab facility. Starter neurons were characterized by the coexpression of DsRed and EGFP, which were restricted to the NTS. Mice were used for immunofluorescence staining one week after injection. For quantifications of subregions, boundaries were based on the Allen Institute’s reference atlas. We selectively analyzed the areas densely labeled in a retrograde manner. The distribution of total cells labeled by RV was measured.

### Larynx perfusions

This procedure was adapted from the literature^13^. Following anesthesia with Avertin (125 mg/kg, i.p.), the trachea was exposed through a midline incision and opened transversely 4–5 cartilaginous rings. PE tube (1 × 0.5 mm) was placed into the distal trachea to maintain spontaneous respiration; another PE tube, covering the larynx and proximal trachea, serves as the inlet/outlet port for the perfusion solution. This inlet port and the outlet port are connected, respectively, to peristaltic pump (XMSPC). Both PE tube were secured with tissue adhesive (3 M Vetbond) to prevent leakage or movement-induced artifacts. Rinse the larynx with PBS for 10 min, followed by the administration of saline or the test solution (RT) at a constant flow rate of 0.1 mL/min; this flow rate was selected to avoid mechanically induced neural responses. PBS washes were interposed between stimuli, and air bubbles were used to separate different solutions within the tubing to prevent mixing. Test solutions comprised PBS (composition: 137 mM NaCl, 2.7 mM KCl, 8 mM Na_2_HPO_4_, and 2 mM KH_2_PO_4_, pH 7.4, 320 mOsmolar), citric acid (PBS: 25 mM, pH 2.6), high salt (5× PBS: 685 mM NaCl, 13.5 mM KCl, 40 mM Na_2_HPO_4_, and 10 mM KH_2_PO_4_, pH 7.4, 1500 mOsmolar), water, saccharin (PBS: 30 mM), sucrose (2/3× PBS: 100 mM, 320 mOsmolar), monosodium glutamate (1/3× PBS: 100 mM, 320 mOsmolar), alanine (2/3× PBS: 100 mM, 320 mOsmolar), denatonium (PBS: 1 mM), quinine (PBS: 1 mM), allyl isothiocyanate (PBS: 3 mM) or capsaicin (PBS: 16.4 μM). All solutions contained 0.0025% Fast Green FCF for visual tracking. Each animal received three stimulus trials, and the response with the greatest magnitude was retained for analysis. Trials were excluded if continuous perfusion was interrupted (e.g., due to mucus obstruction). The output tube was advanced into the pharynx a total of 10 times for mechanical stimulation, and the number of swallowing was recorded. Additionally, calcium signals that trigger swallowing were recorded for *TH*-ires-Cre mice in response to laryngeal perfusion with various fluids.

### Histological procedures

Immunohistochemical staining was performed following a published protocol^45^. Under urethane anesthesia, mice were transcardially perfused with saline followed by 4% paraformaldehyde (PFA) in PBS. Brains were harvested and cryoprotected in 30% sucrose (in PBS) until submerged. To preserve antigenicity for GABA and glutamate, post-fixation was deliberately omitted. Coronal sections (40 µm) were cut on a cryostat (Leica CM1900), and those containing the NTS, Amb/RVLM, LPB, or Arc were collected. Sections were blocked for 2 h in PBS containing 10% normal goat serum and 0.7% Triton X-100, then incubated overnight at 4 °C with primary antibodies diluted in the same blocking solution. After three rinses in PBS with 0.7% Triton X-100, sections were incubated for 2 h at room temperature with fluorophore-conjugated secondary antibodies (Cy2/Cy3/Cy5, 1:500), followed by three additional washes, DAPI counterstaining, and a final PBS rinse. Finally, cover the sections with an anti-fluorescence quenching mounting medium onto the glass slides. The coronal brain sections were imaged using a Leica Aperio Versa 8 microscope (10× objective lens), an automated slide scanning system (EVIDENT VS 200) or a Carl Zeiss LSM 980 laser scanning confocal microscope (20× and 60× oil-immersion objective lenses). Sequential excitation with 488-, 546-, and 633-nm lasers was applied to avoid bleed-through, and the acquired confocal images were analyzed using ZEN software.

The immunofluorescence staining procedure for the vagal ganglion was performed as previously described with minor modifications. In brief, cryostat sections (10 μm) were incubated for 1 h with a blocking solution (PBS containing 3% BSA and 0.3% Triton X-100) at 37 °C, after which the liquid around the specimen was wiped off, and the primary antibody (diluted in PBS containing 1% BSA and 0.1% Triton X-100) was incubated at 4 °C overnight. The following day, the sections were incubated with the secondary antibody for 2 h at 37 °C. Antibodies used for immunohistochemistry are provided in Supplementary Table 2. The Piezo2 antibody used in this study (Catalog #NBP1-78624, Novus) has been employed for staining mouse neural tissues in multiple published research papers^65–67^. All three biological replicate experiments yielded similar results.

### Slice physiology

Slice physiological recording was performed according to a published protocol^45^. Coronal brain slices (250 µm thickness) containing either the NTS or the Amb/RVLM were obtained from adult mice that had been deeply anesthetized with isoflurane prior to decapitation. Following rapid extraction, the brains were immersed in an ice-cold, oxygenated (95% O_2_ / 5% CO_2_) slicing solution composed of (in mM) 228 sucrose, 11 glucose, 26 NaHCO_3_, 1 NaH_2_PO_4_, 2.5 KCl, 7 MgSO_4_, and 0.5 CaCl_2_. Sectioning was performed on a vibratome (VT1200S, Leica Microsystems, Germany), and the resultant slices were allowed to recover for 30 min at 28 °C in oxygenated artificial cerebrospinal fluid (ACSF) containing (in mM) 119 NaCl, 2.5 KCl, 1 NaH_2_PO_4_, 1.3 MgSO_4_, 26 NaHCO_3_, 10 glucose, and 2.5 CaCl_2_. Patch electrodes were fabricated from borosilicate glass capillaries (Warner Instruments) using a PC-100 puller (Narishige, Japan). Electrode resistances ranged from 3.0 to 3.5 MΩ. The NTS slices were visualized under a ×40 water-immersion objective (Olympus, Japan) equipped with differential interference contrast (DIC) optics and a CCD camera (QImaging Rolera-XR, Canada). Thereafter, brain slices were maintained at room temperature in the same oxygenated ACSF for an additional hour before being transferred to the recording chamber, where they were continuously superfused with ACSF at a flow rate of 1 mL/min. After achieving whole-cell configuration and allowing adequate intracellular dialysis, series resistance was compensated to 10–15 MΩ; recordings with series resistance exceeding 15 MΩ were discarded. To elicit light-activated action potentials in ChR2-mCherry-positive neurons, a fiber with a diameter of 200 µm was placed above the brain slice, and a series of light pulses (wavelength: 473 nm; pulse duration: 1 ms; power: 20 mW) were applied; the timing of these pulses was controlled by the Clampex 10 software. For current-clamp recordings of action potentials, the pipette internal solution contained (in mM) 135 K-methanesulfonate, 10 HEPES, 1 EGTA, 1 Na-GTP, and 4 Mg-ATP (pH 7.4). For recording light-evoked synaptic currents from ChR2-mCherry-negative neurons, single light pulses (1 ms) were delivered in the presence of 4-AP (20 µM) and TTX (1 µM). To determine the neurotransmitter phenotype of the ChR2-expressing terminals, the following receptor antagonists were bath-applied via ACSF: D-AP5 (50 µM) plus CNQX (20 µM) for glutamate receptors, picrotoxin (PTX, 50 µM) or bicuculline (BIC, 20 µM) for GABAₐ receptors. For voltage-clamp recordings of postsynaptic currents, the internal solution consisted of (in mM) 135 CsCl_2_, 10 HEPES, 1 EGTA, 1 Na-GTP, and 4 Mg-ATP (pH 7.4). Signals were acquired using a MultiClamp700B amplifier and Clampex 10 software (Molecular Devices).

### Drug preparation and concentration

GsMTx4 (a Piezo family antagonist, GlpBio, GC38465) and GSK2193874 (a TRPV4 antagonist, Sigma, SML0942) were dissolved in dd H_2_O to prepare stock solutions at 20 mg/mL and 200 mg/mL, respectively. HC-030031 (a TRPA1 antagonist, Sigma, H4415) and Mifepristone (RU-486, a TRPM3 antagonist, Sigma, M8046) were dissolved in DMSO to prepare stock solutions at 200 mg/mL. Prior to the experiments, GsMTx4 and HC-030031 were diluted 1:200 in saline, while GSK2193874 and Mifepristone were diluted 1:20 in saline.

### Nerve transections

The superior laryngeal nerve (SLN) branches are located below the carotid artery in mice. The SLN was exposed by blunt dissection of the sternothyroid muscle. The glossopharyngeal nerve (GPN) was exposed by the removal of the digastric muscles and posterior horn of the hyoid bones. The experimental treatment groups of nerve transection included: (1) left SLN transection; (2) right SLN transection; (3) bilateral SLN transection; (4) left GPN transection; (5) right GPN transection; (6) bilateral GPN transection; (7) bilateral GPN transection combined with left SLN transection; (8) bilateral GPN transection combined with right SLN transection; (9) bilateral transection of both the GPN and SLN. In each group, during a post-operative stabilization period, swallowing was evoked by applying a series of calibrated Von Frey filament stimuli to the pharynx.

### Vagal ganglion neurons culture

Under deep anesthesia, the ganglia were rapidly excised from the mice and immediately transferred into ice-cold D-Hanks solution (Gibco). All dissections were completed within 10 min per animal. The isolated ganglia were subjected to enzymatic digestion at 37 °C for 30 min using a mixture of collagenase type 2 (2 mg/mL) and dispase II (7.5 mg/mL). Digestion was terminated by suspending the tissue in 10 mL of DMEM (Gibco) supplemented with 10% fetal bovine serum (Gibco). The ganglia were then mechanically dissociated into a single-cell suspension, and the resulting cells were plated onto 8-mm-diameter glass coverslips that had been pre-coated with 100 μg/mL poly-D-lysine (BD Biosciences). The cultures were maintained at 37 °C under a humidified atmosphere of 5% CO_2_ and 95% O_2_. Coverslips bearing adherent vagal ganglion neurons were placed in a 0.5-mL recording chamber, and the cells were bathed in an external solution containing 5 mM KCl, 2 mM CaCl_2_, 2 mM MgCl_2_, 145 mM NaCl, 10 mM HEPES, and 10 mM glucose, with the osmolarity adjusted to 320 mOsm and the pH titrated to 7.35 using NaOH. After 4 h, RV-labeled vagal ganglion neurons from *TH*-ires-cre mice were aspirated (under a microscope) into a patch pipette using a conventional patch-clamp setup with negatively pressurized pipette holder. The electrode tip was then quickly broken off into a 0.2-mL PCR tube containing 4 μL lysis buffer for subsequent SMART-seq2 library preparation.

### Vagal ganglion neuron single-cell library preparation

The vagal ganglion neurons were placed in 4 μL lysis buffer (containing oligo-dT primer, Recombinant RNase inhibitor (TaKaRa, Cat # 2313 A), and 1% Triton X-100, dNTP mix). Reverse transcription and cDNA amplification were performed following SMART-seq2 protocol^68^. After cDNA amplification, 25 μL of the sample was subjected to cDNA purification on AMPure XP beads (BeckmanM, Cat # A63881). 5 ng of each purified cDNA was used to construct the sequencing library using TruePrep® DNA Library Prep Kit V2 for Illumina (Vazyme, Cat # TD502) and TruePrep® Index Kit V2 for Illumina (Vazyme, Cat # TD202), with fragments >300 bp in length from each neuron. The SMART-seq data was analyzed using TrimGalore, HISAT2 (version 2.2.1), featureCounts and Seurat package (v4.1.0). We used our previously established vagal ganglion neuronal single-cell database (PMID: 38288368). Sequencing data was deposited in the Genome Sequence Archive (GSA) database (GSA accession number: CRA007790). For clustering analysis, we performed clustering based on 9000 highly variable feature genes with the FindVariableFeatures() function in Seurat. Feature scores between RV retrogradely traced vagal neurons and all 31 clusters of vagal sensory neurons were calculated according to the signature gene expression similarity. These are listed in Supplementary Data 1.

### Single-cell real-time quantitative PCR

Primary cultures of vagal ganglia were prepared from *Piezo2-Flox* mice using the aforementioned method. The ganglia were dissociated into individual cell suspensions; a negative-pressure electrode device equipped with a patch electrode was then used to directly introduce the cell into the patch electrode via the suction method under a fluorescence microscope; the cell was blown into a 0.2 mL PCR tube containing 4 μL of lysis buffer (SuperScript^TM^ IV CellsDirect^TM^ cDNA Synthesis Kit, Invitrogen, 11750150). Lysis buffer contained SuperScript^TM^ IV CellsDirect^TM^ Lysis Solution, Lysis Enhancer and DNase I. The sample was incubated at room temperature for 7–15 min. 3 μL of Stop solution was added, gently pipette to mix, and incubate at room temperature for 2 min. Subsequently, SuperScript^TM^ IV RT Master Mix was added to reverse transcribe into cDNA. The reaction conditions were annealing at 25 °C for 10 min, reverse transcription at 50 °C for 10 min and enzyme inactivation at 85 °C for 5 min. Finally, the cDNA samples were used for qPCR detection using Power SYBR^®^ Green PCR Master Mix (Thermo Fisher Scientific, 4367659). The reaction conditions were one cycle of initial denaturation at 95 °C for 10 min, followed by 40 cycles of 95 °C for 15 s, 60 °C for 60 s. Expression data were calculated from the cycle threshold (Ct) value, and the 2-ΔΔCT method was used to calculate relative RNA level. The reagents used in this study are listed in Supplementary Table 3.

Piezo2 forward primer: 5’-CTGCCCCTTGCCGAGATTA-3’;

reverse primer: 5’-AAGTCATAGACAAGAAACACGGGG-3’;

Actin forward primer: 5’-CCCAGCAAGGACACTGAGCAA-3’;

reverse primer: 5’-TTATGGGGGTCTGGGATGGAAA-3’.

### Cell-counting strategies

For counting cells in the vagal ganglion, we collected 10 μm coronal sections for each mouse. Five sections evenly spaced by 40 μm were sampled for immunohistochemistry to label cells positive for different markers. We acquired confocal images (ZEISS LSM900, 60× objective) within intermediate and deep layers of the vagal ganglion neurons followed by cell counting with ZEN software. We counted the numbers of NeuN+ neurons and calculated the average in the neuronal population retrogradely labeled by RV-DsRed.

For counting cells in the NTS, we collected coronal sections (40 μm) from bregma –7.32 to –7.52 for each brain. Three sections spaced by 200 μm were sampled for immunohistochemistry to label cells positive for different markers. We calculated the average in NTS neurons labeled by retrograde tracers injected into different target regions (AAV2/2Retro-EF1α-DIO-EGFP-WPRE-hGH-pA) in Amb/RVLM; AAV2/2Retro-EF1α-DIO-mCherry-WPRE-hGH-pA in Arc or LPB). For counting cells in the whole brain, we collected coronal sections (40 μm) for each mouse. One section every 200 μm was taken for immunohistochemistry to label positive cells. We acquired micrographs (10× objective) of these nucleus and calculated the average of RV-DsRed+ or PRV-RFP+ neurons. Summary of cell counting strategy is provided in Supplementary Table 5.

### Quantification and statistical analysis

Data were presented as the Mean ± SEM and analyzed using GraphPad Prism 9.1. Statistical significance was evaluated using either a Student’s *t* test or one-way analysis of variance (ANOVA) or two-way ANOVA. *P* value < 0.05 was considered to indicate a statistically significant difference. Summary of all experimental designs and Statistical analysis in this study are listed in Supplementary Data 2 and Supplementary Data 3.

### Reporting summary

Further information on research design is available in the Nature Portfolio Reporting Summary linked to this article.

## Supplementary information

Supplementary Information

Description of Additional Supplementary Files

Supplementary Data 1

Supplementary Data 2

Supplementary Data 3

Supplementary Movie 1

Supplementary Movie 2

Reporting Summary

Transparent Peer Review file

## Source data

Source Data

## Data Availability

Sequencing data generated in this study have been deposited in the Genome Sequence Archive (GSA) database under accession code (CRA047891 [https://ngdc.cncb.ac.cn/gsa/browse/CRA047891] and CRA047906). This study utilizes the single-cell database of vagal ganglion neurons previously established by our research group^45^ and its corresponding sequencing data (GSA accession number: CRA007790). All other data are available in the main text or supplementary materials. Source data are provided with this paper.

## Code availability

Code has been deposited in the public repository *Zenodo* (https://doi.org/10.5281/zenodo.17486163) and (https://doi.org/10.5281/zenodo.17486084).
