# Supplementary material for: A pharynx-to-brain sensory pathway mediates swallowing-like behavior evoked by mechanical stimulation
Source: Nat Commun. 2026 Sep 28;17:11180. doi: 10.1038/s41467-026-78007-9 (PMC13714286; doi:10.1038/s41467-026-78007-9)
Supplement: Supplementary file 1 [file 41467_2026_78007_MOESM1_ESM.pdf]

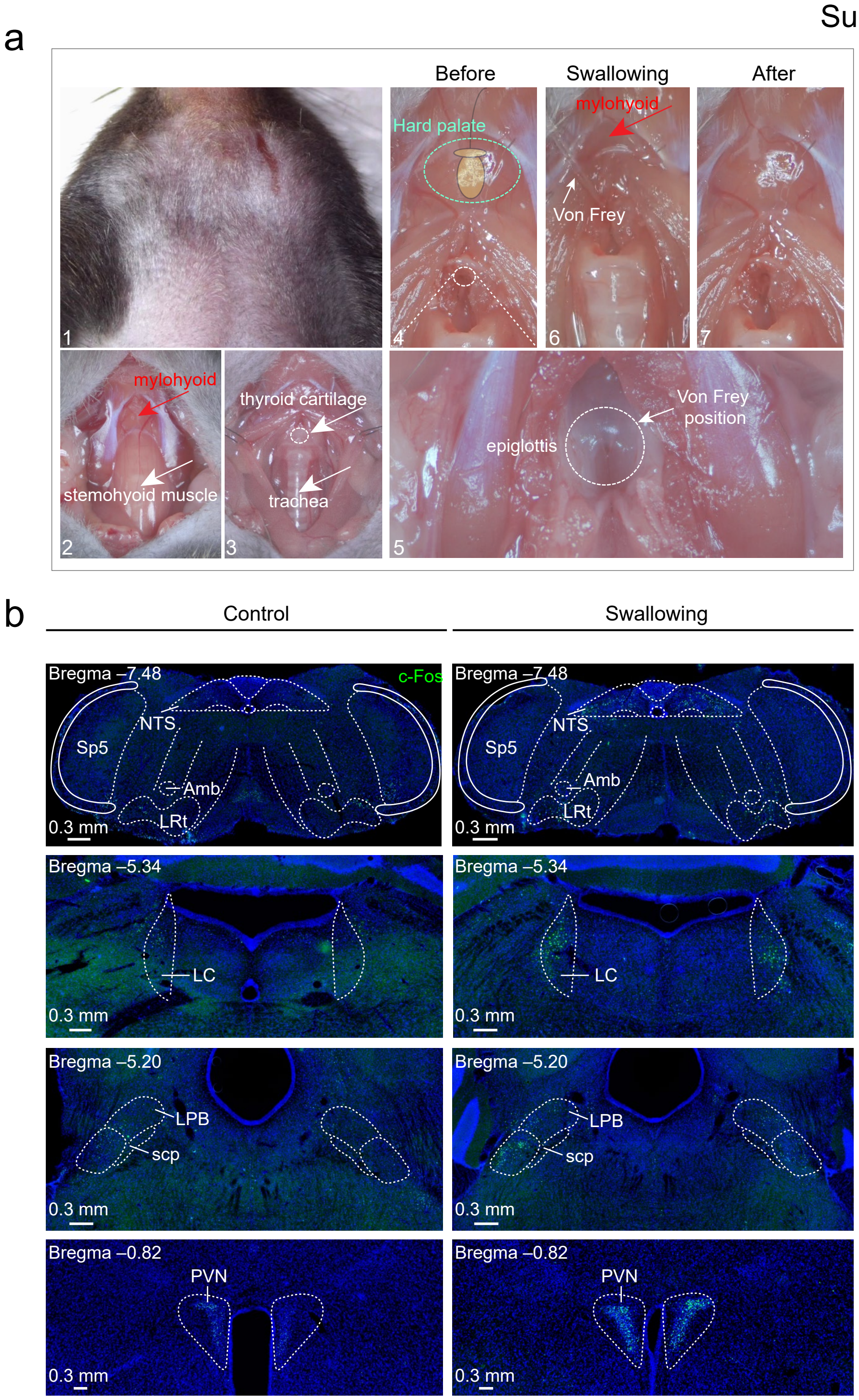

Supplementary information, Fig.1 Whole-brain c-Fos mapping activated by swallowing-like behavior.

a Surgical procedure for inducing swallowing in mice through mechanical stimulation of the pharynx. 1. Anesthetized mice were placed in a supine position. 2. Incise the skin of the neck to expose the mylohyoid muscle and sternohyoid muscle. 3. Make a longitudinal incision in the sternohyoid muscle and fix it to both sides with surgical sutures. 4. Incise the thyroid cartilage to expose the pharynx, and place the sensing latex balloon orally at the base of the tongue and the hard palate. 5. The stimulation site of the Von Frey filaments. 6. Stimulation of the pharynx with Von Frey filaments induced contraction of the mylohyoid muscle and elevation of the larynx during swallowing. 7. The swallowing reflex was completed.

b Whole-brain images immunostained for a neuronal activation marker (c-Fos) associated with swallowing, these neurons were activated after mechanical stimulation of the pharynx in *WT* mice with 0.4 g Von Frey for 90 minutes. These brain regions were primarily concerned with nucleus tractus solitarius (NTS), ambiguus nucleus (Amb), rostroventrolateral reticular nucleus (RVLM), paraventricular hypothalamic nucleus (PVN), lateral parabrachial nucleus (LPB) and locus coeruleus (LC).

Supplementary Figure 2

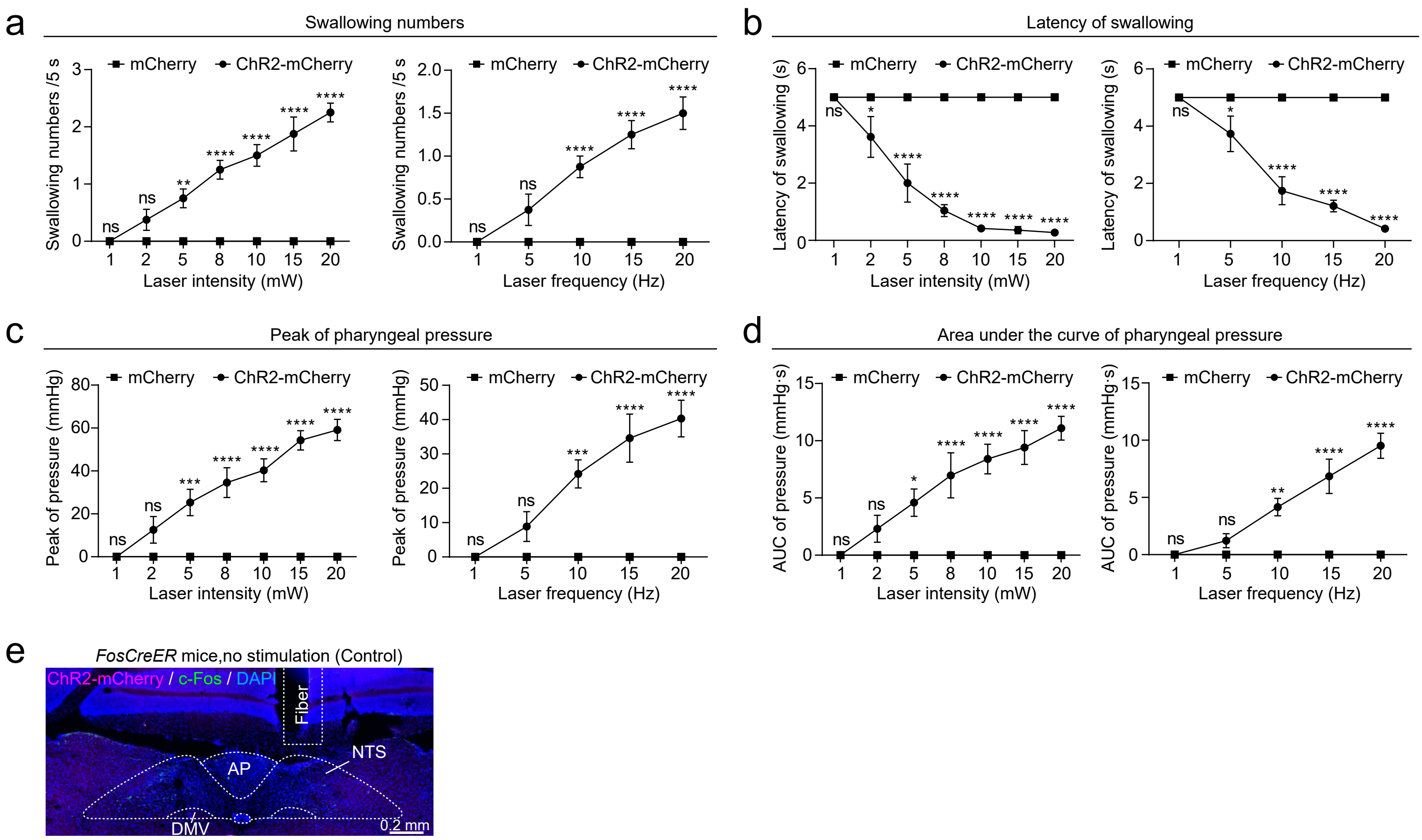

Supplementary information, Fig.2 Swallowing triggered by the light-activation of NTS neurons trapped by mechanical stimulation was laser power and laser frequency-dependent.

a–d Total number of swallowing (**a**), latency time (**b**), peak (**c**) and AUC of pharyngeal pressure (**d**) in response to swallowing evoked by light-activating NTS neurons (left: 473 nm, 10 ms, 20 Hz, 1, 2, 5, 8, 10, 15 and 20 mW, right: 473 nm, 10 ms, 10 mW, 1, 5, 10, 15 and 20 Hz) in *FosCreER* mice. In **a–d**, mCherry: n=6 mice, ChR2: n=8 mice.

e Brain images immunostained for the control group mice (no mechanical stimulation) in the FosTRAP experiment.

Data were shown as mean ± s.e.m. (error bars). Statistical analysis in **a–d** were performed using Two-way ANOVA (*P < 0.05, **P < 0.01, ***P < 0.001, ****P < 0.0001, ns ≥ 0.05). Source data are provided as a Source Data file.

Supplementary Figure 3

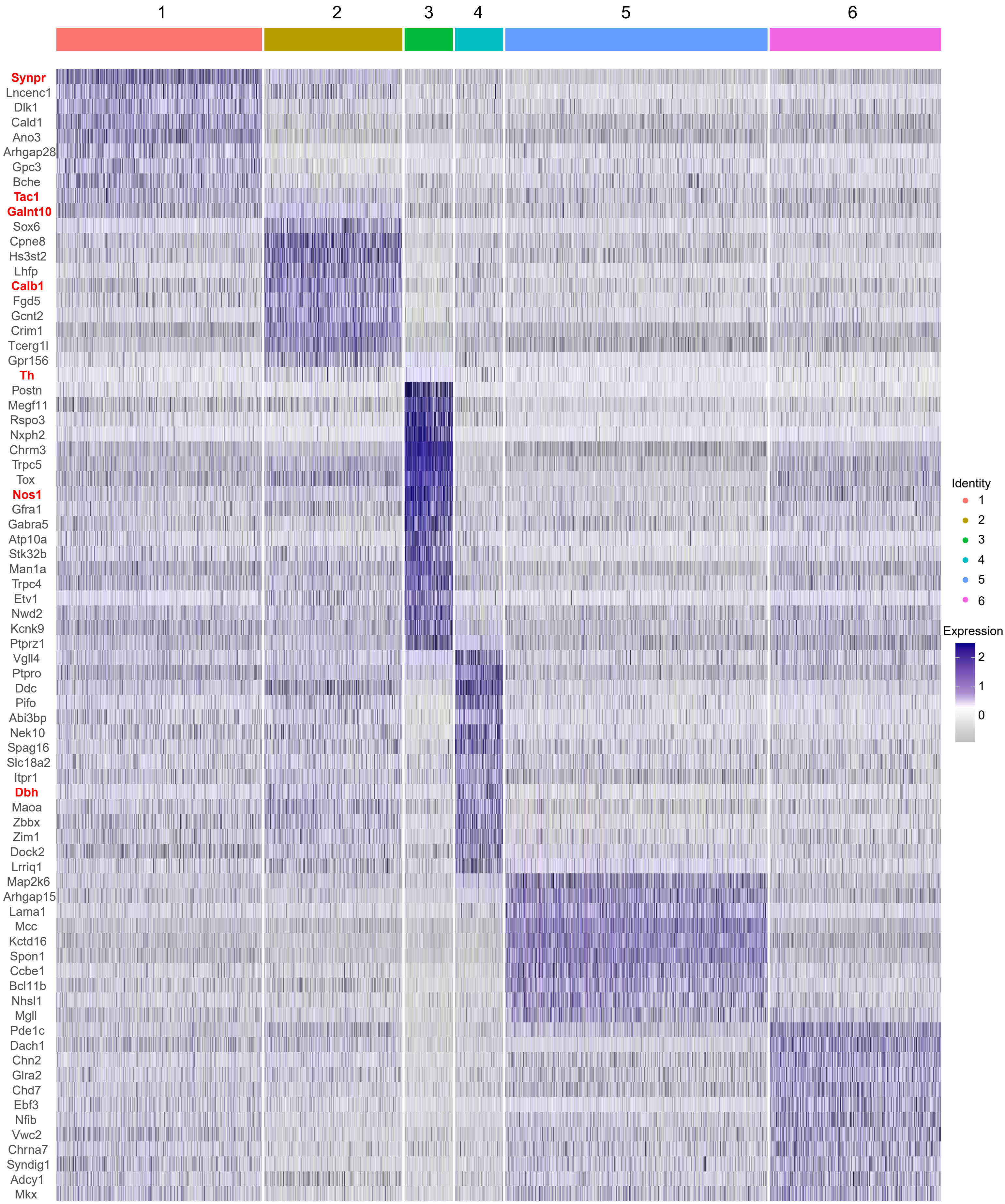

Supplementary information, Fig.3 Heatmap of top 11 marker genes (y axis) for every sub-cluster (x axis) in NTS neurons of mice.

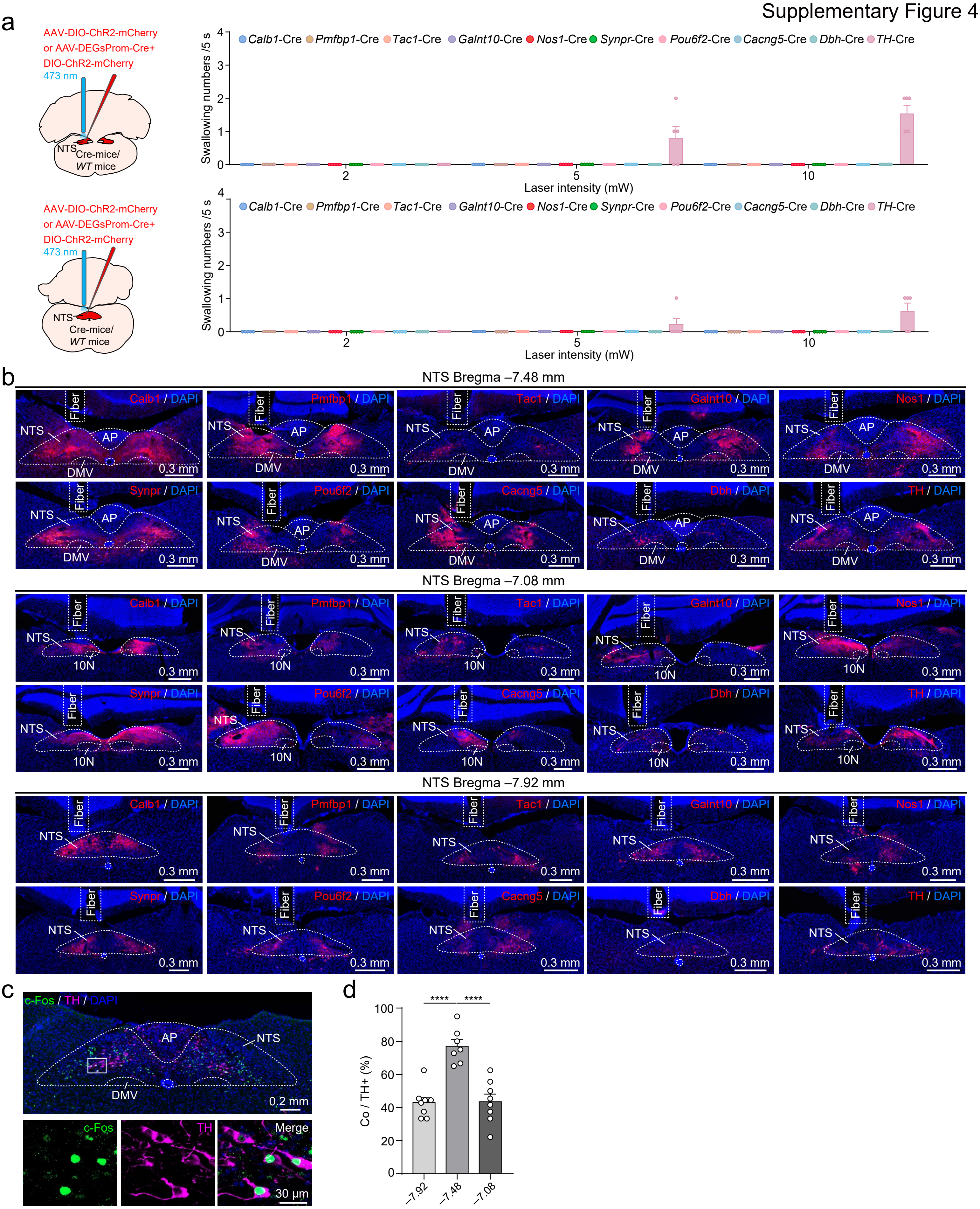

Supplementary information, Fig.4 Genetic screening for regulating swallowing.

a Schematic illustrating ChR2-mCherry injection and fiber implantation sites in the rostral NTS (Bregma –7.08 mm) and caudal NTS (Bregma –7.92 mm) of Cre-line and *WT* mice; statistical graphs showing swallowing number evoked by light (473 nm, 10 ms, 20 Hz, 2, 5 and 10 mW) of medial-lateral or anterior-posterior axes of the NTS (n=5 mice).

b Representative images of ChR2-mCherry expression and optic fiber placements of all AAV-DEGsProm-Cre virus or Cre-line mice.

c Representative images showing c-Fos neurons in the NTS activated by mechanical stimulation of the pharynx in *WT* mice.

d The proportion of activated neurons (c-Fos+/TH+) in the caudal, intermediate, and rostral subregions of the NTS following swallowing evoked by mechanical stimulation of the pharynx. The broken white lines in the section images represent boundaries of brain regions. Statistical analysis in **d** was performed using One-way ANOVA (**** P < 0.0001). Source data are provided as a Source Data file.

Supplementary Figure 5

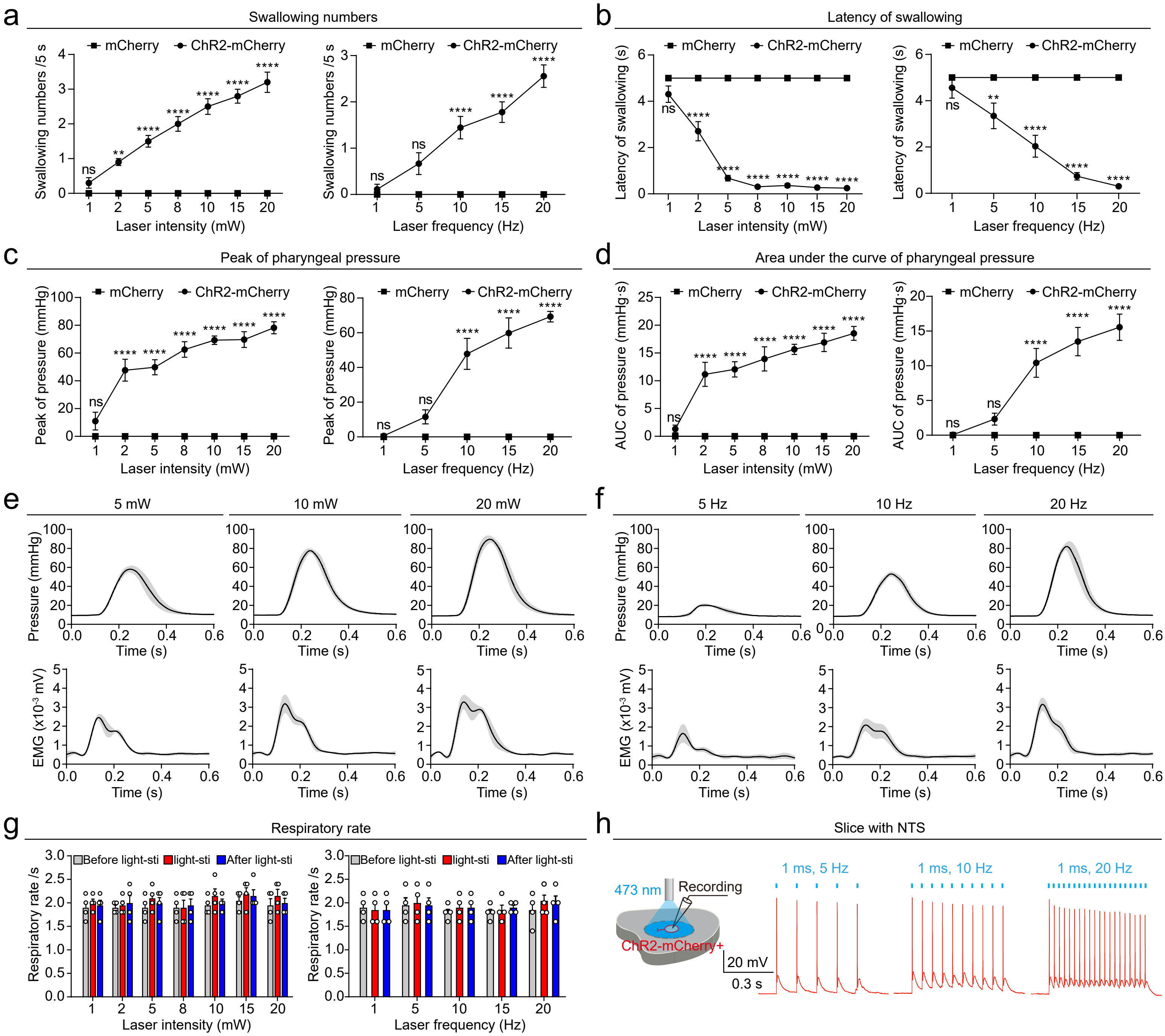

Supplementary information, Fig.5 Swallowing triggered by the light-activation of TH+ NTS neurons was laser power and laser frequency-dependent.

a–d Total number of swallowing (**a**), latency time (**b**), peak (**c**) and AUC of pharyngeal pressure (**d**) induced by activating NTS neurons with light (left: 473 nm, 10 ms, 20 Hz, 1, 2, 5, 8, 10, 15 and 20 mW, and right: 473 nm, 10 ms, 10 mW, 1, 5, 10, 15 and 20 Hz) in *TH*-ires-Cre mice. In **a–d**, left: mCherry: n=6 mice, ChR2: n=10 mice, right: mCherry: n=6 mice, ChR2: n=9 mice.

e–f Oscillogram of pharyngeal pressure and EMG of mylohyoid muscle activity during swallowing (n=4 mice).

g Respiratory rate of *TH*-ires-Cre mice upon activation of NTS neurons by light (left: 473 nm, 10 ms, 20 Hz, 1, 2, 5, 8, 10, 15 and 20 mW, and right: 473 nm, 10 ms, 10 mW, 1, 5, 10, 15 and 20 Hz) (mCherry: n=4 mice, ChR2: n=4 mice).

h Light-pulse trains (1 ms, 20 mW, 5, 10 and 20 Hz) reliably evoked phase-locked spiking in ChR2-expressing NTS neurons in acute brain slices of *TH*-ires-Cre mice (n=12 cells).

Data were shown as mean ± s.e.m. (error bars). Statistical analysis in **a–d** and **g** were performed using Two-way ANOVA (**P < 0.01, ****P < 0.0001, ns ≥ 0.05). Source data are provided as a Source Data file.

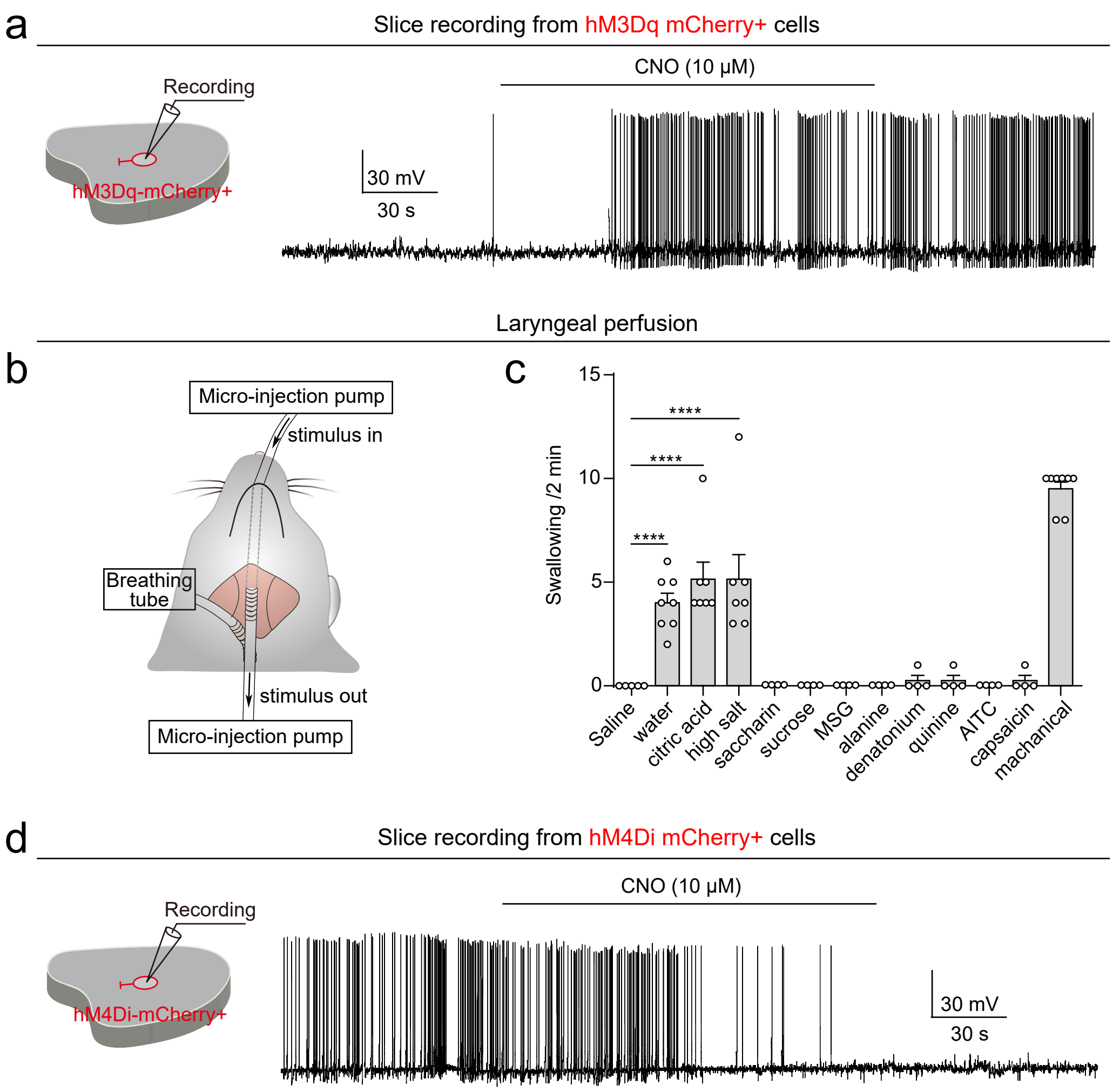

Supplementary information, Fig.6 Whole-cell recording of hM3Dq-mCherry+ and hM4Di-mCherry+ neurons in the NTS.

a Schematic diagram showing whole-cell recording of hM3Dq-mCherry+ neurons in the NTS within acute brain slices (left). Additionally, the example trace of action potential firing demonstrating the effectiveness of CNO to chemogenetically activate hM3Dq-expressing NTS neurons in acute brain slices (n=9 cells) (right). CNO was dissolved in artificial cerebrospinal fluid (ACSF) (10 μM) and subsequently perfused over the brain slices.

b Schematic for perfusion of laryngeal stimuli.

c The total number of swallowing induced by laryngeal perfusion with various chemical stimuli in *WT* mice. Each data point represents a single mouse (See methods).

d Schematic diagram showing whole-cell recording of hM4Di-mCherry+ neurons in the NTS within acute brain slices (left). Example trace of action potential firing demonstrating the efficacy of CNO in chemogenetically silencing hM4Di-expressing NTS neurons in acute brain slices (n=10 cells) (right).

Data were shown as mean ± s.e.m. (error bars). Statistical analysis in **c** was performed using One-way ANOVA (***P < 0.001, ****P < 0.0001). Source data are provided as a Source Data file.

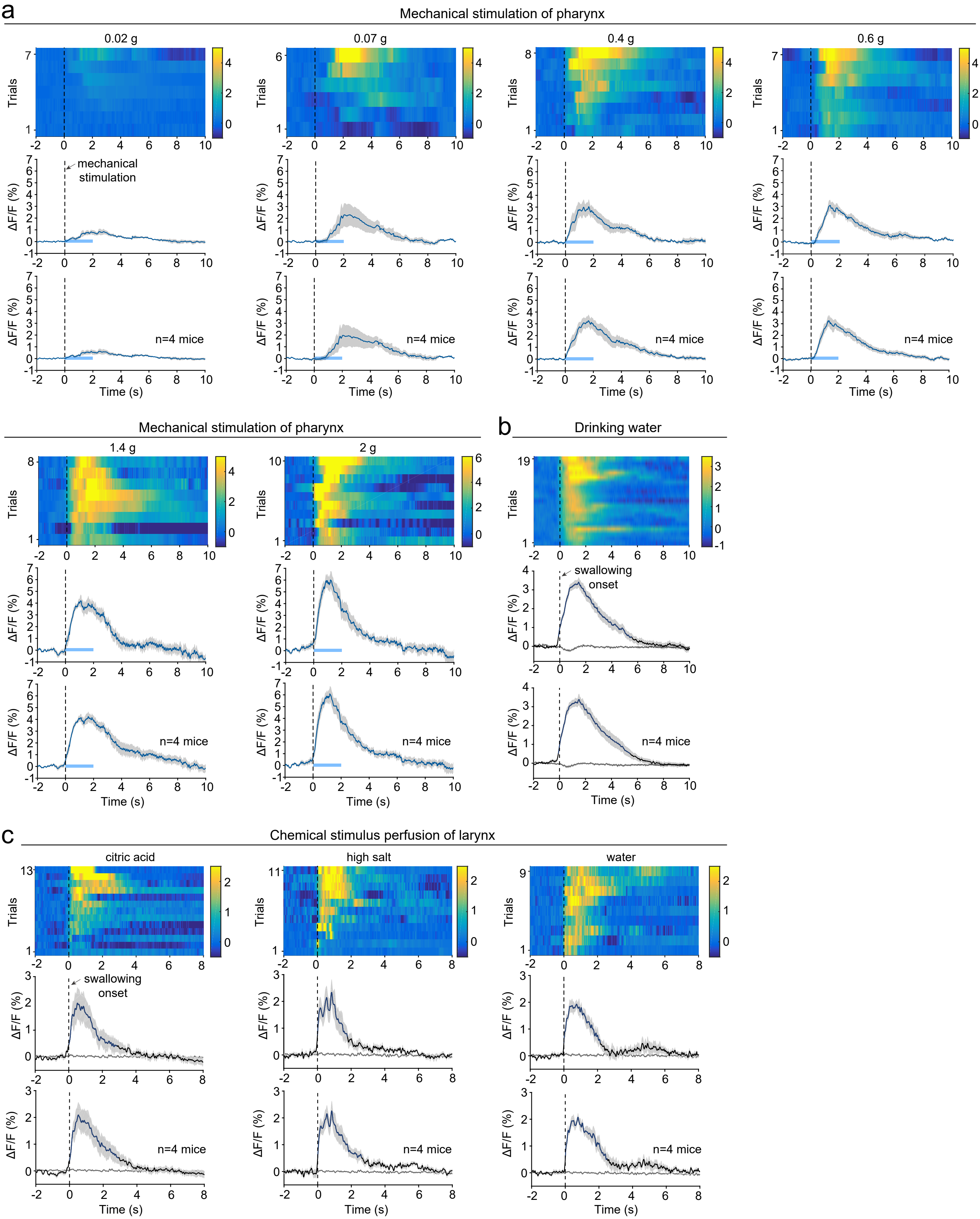

Supplementary information, Fig.7 Calcium activity of TH+ NTS neurons associated with mechanical and chemical stimulation.

Continuation of legend on next page

a Heatmap and average Ca2+ transients displaying 6-10 trials of normalized GCaMP fluorescence change of TH+ NTS neurons. Calcium transients were elicited by mechanical stimulation of the pharynx (n=4 mice). Blue thick lines indicate mean and gray shaded areas indicate s.e.m.. The broken line represents the time points of the mechanical stimulation. The blue shading represents the time window for mechanical stimulation.

b Ca2+ signals associated with trial of swallowing behavioral session. Heatmap displaying 19 trials of normalized GCaMP fluorescence changes (ΔF/F) synchronized with swallowing behavior induced by water (top); Normalized GCaMP signal changes in TH+ NTS neurons when mice were induced to exhibit swallowing behavior with water; Average GCaMP transients of TH+ NTS neurons associated with swallowing behavior induced by water (middle and bottom) (n=4 mice). Thick lines indicate mean and shaded areas indicate s.e.m.. Blue segments indicate Ca2+ signal exceeds above 15% from baseline to peak. The horizontal gray line represents motion interference recorded by channel 405.

c Heatmap and average Ca2+ transients displaying 9-13 trials of normalized GCaMP fluorescence changes of TH+ NTS neurons. Calcium transients were synchronized with swallowing behavior evoked by chemical stimulation of the larynx (n=4 mice). GCaMP signals showed a detectable increase before the swallowing event, and remained elevated during subsequent swallowing. Thick lines indicate mean and shaded areas indicate s.e.m.. Blue segments indicate Ca2+ signal exceeds above 18% from baseline to peak. The broken line represents the time points of swallowing. The horizontal gray line represents GCaMP signal changes when saline was perfused through the mouse larynx. Source data are provided as a Source Data file.

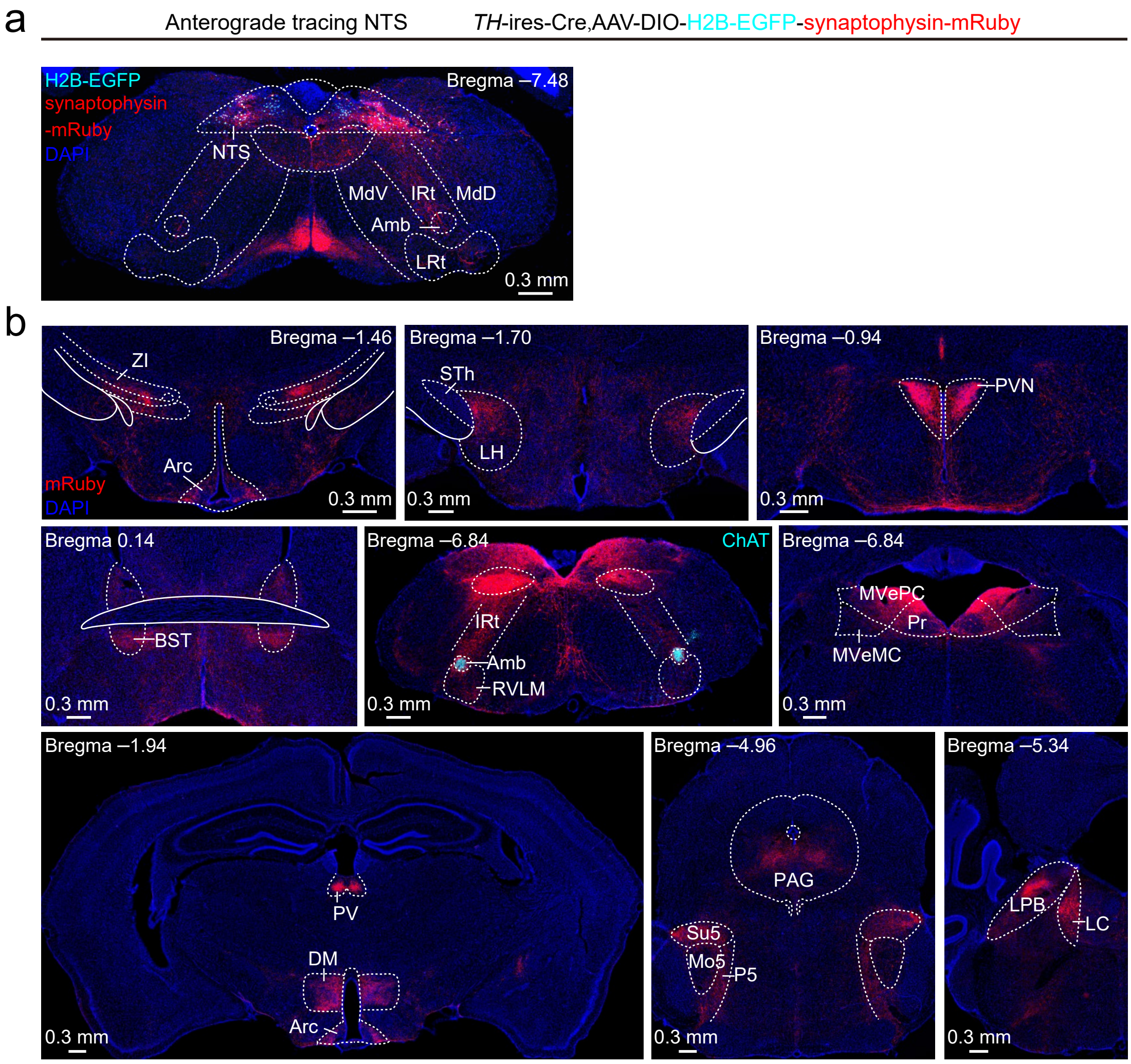

Supplementary information, Fig.8 Projections of TH+ NTS neurons.

a–b Representative brain section images showing mRuby+ axonal projections from TH+ NTS neurons in the Amb/RVLM, zona incerta (ZI), arcuate hypothalamic nucleus (Arc), lateral hypothalamic area (LH), paraventricular hypothalamic nucleus (PVN), bed nucleus of the stria terminalis (BST), medial vestibular nucleus (MVN), paraventricular thalamic nucleus (PV), dorsomedial hypothalamic nucleus (DM), periaqueductal gray (PAG), supratrigeminal nucleus (Su5), peritrigeminal zone (P5), lateral parabrachial nucleus (LPB) and locus coeruleus (LC).

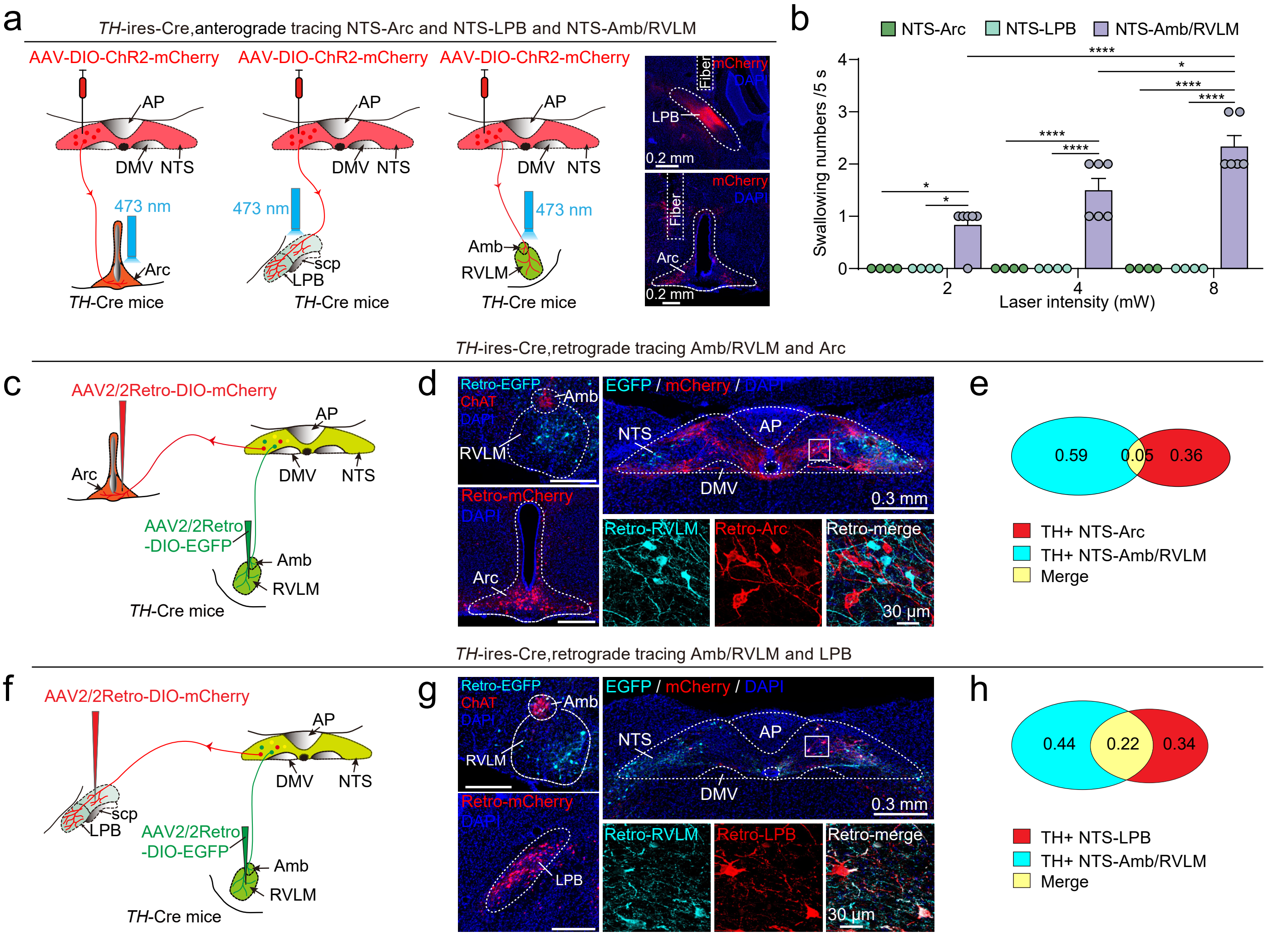

Supplementary information, Fig.9 Comparison of the TH+ NTS-Amb/RVLM with TH+ NTS-Arc or TH+ NTS-LPB pathway.

a Schematic diagram of optogenetic strategy of activating the axonal terminals of NTS projecting to Arc or LPB or Amb/RVLM in different *TH*-ires-Cre mice (left). Example micrographs displaying the optical fiber track above the axon terminal in the LPB and Arc (right).

b Quantitative analysis of swallowing numbers of *TH*-ires-Cre mice with optogenetic activation (473 nm, 10 ms, 20 Hz, 2, 4 and 8 mW) of the NTS-Arc (n=4) or NTS-LPB (n=4) or NTS-Amb/RVLM (n=6) pathway severally. For NTS-Amb/RVLM pathway, the frequency of swallowing during light-evoked is a function of laser intensity (See Fig. 5 and Fig. S10).

c–e Schematic diagram of viral injection strategy to Arc-projecting and Amb/RVLM-projecting TH+ NTS neurons in the same mouse (**c**). Example coronal brain section and the magnified fields showing the distribution of Amb/RVLM-projecting (cyan) and Arc-projecting (red) TH+ neurons in the NTS (**d**). The scale is 0.3 mm in **d** left. The fraction of EGFP-labeled, mCherry-labeled and dually labeled neurons in the NTS (**e**).

f–h Schematic diagram of viral injection strategy to LPB-projecting and Amb/RVLM-projecting TH+ NTS neurons in the same mouse (**f**). Example coronal brain section and the magnified fields showing the distribution of Amb/RVLM-projecting (cyan) and LPB-projecting (red) TH+ neurons in the NTS (**g**). The scale is 0.3 mm in **g** left. The fraction of EGFP-labeled, mCherry-labeled and dually labeled neurons in the NTS (**h**).

Data were shown as mean ± s.e.m. (error bars). The broken white lines in the section images represent boundaries of brain regions. Statistical analysis in **b** was performed using Two-way ANOVA (*P < 0.05, ****P < 0.0001). Source data are provided as a Source Data file.

Supplementary Figure 10

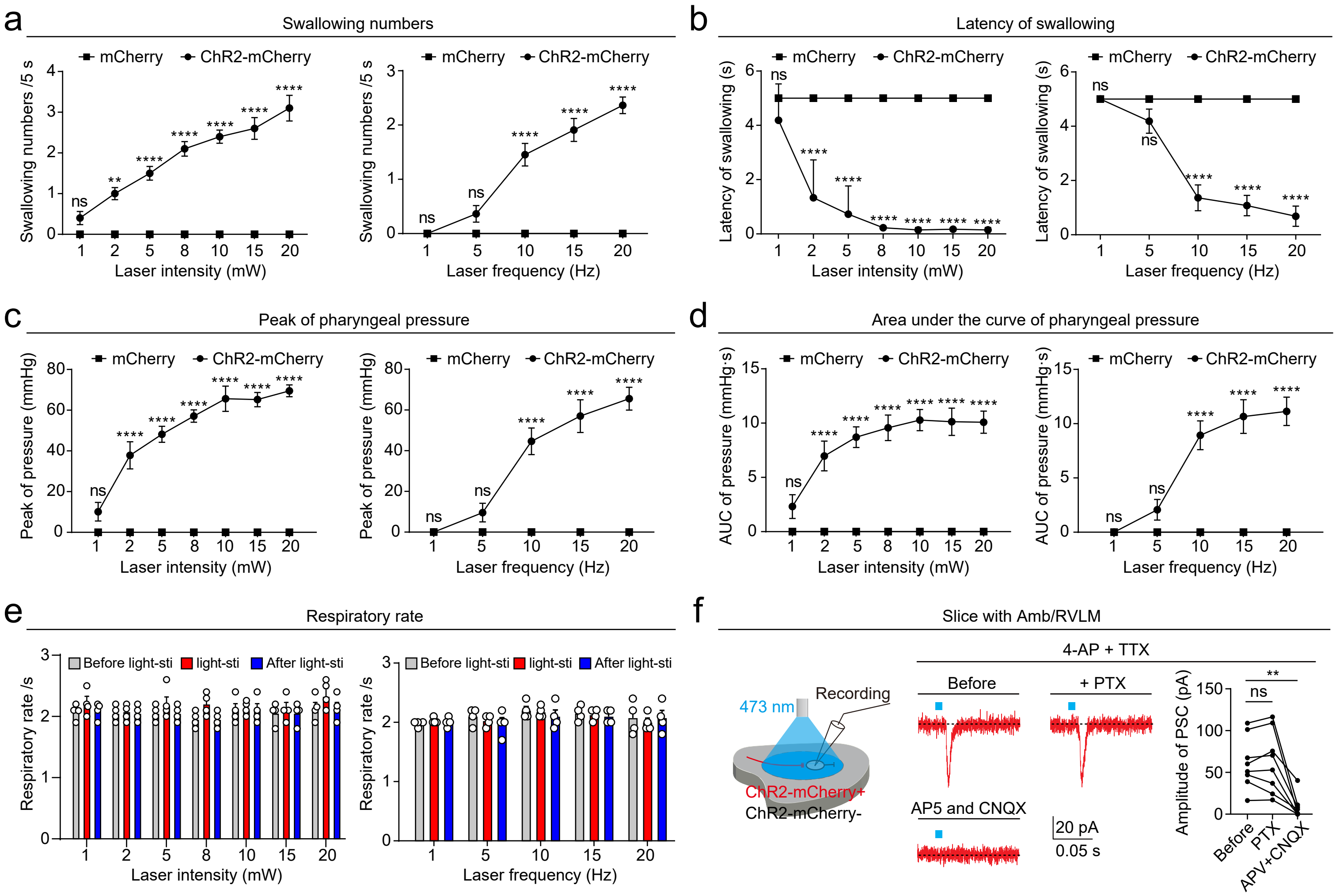

Supplementary information, Fig.10 Swallowing triggered by the light-activation of TH+ NTS-Amb/RVLM pathway was laser power and laser frequency-dependent.

a–d Total number of swallowing (**a**), latency time (**b**), peak (**c**) and AUC of pharyngeal pressure (**d**) in response to swallowing evoked by light-activating NTS-Amb/RVLM pathway (left: 473 nm, 10 ms, 20 Hz, 1, 2, 5, 8, 10, 15 and 20 mW, right: 473 nm, 10 ms, 10 mW, 1, 5, 10, 15 and 20 Hz) in *TH*-ires-Cre mice. In **a–d**, left: mCherry: n=6 mice, ChR2: n=10 mice; right: mCherry: n=6 mice, ChR2: n=11 mice.

e Respiratory rate of *TH*-ires-Cre mice upon activation of NTS-Amb/RVLM pathway by light (left: 473 nm, 10 ms, 20 Hz, 1, 2, 5, 8, 10, 15 and 20 mW, and right: 473 nm, 10 ms, 10 mW, 1, 5, 10, 15 and 20 Hz) (mCherry: n=4 mice, ChR2: n=4 mice).

f Schematic diagram showing recording of light-evoked (473 nm, 2 ms, 20 mW) PSCs from ChR2-mCherry-negative neurons with Amb/RVLM in acute slice (left). And effects of PTX and D-AP5/CNQX on light-evoked PSCs recorded from ChR2-mCherry-negative neurons in the Amb/RVLM (n=8 cells) (right).

Data were shown as mean ± s.e.m. (error bars). Statistical analysis in **a–e** were performed using Two-way ANOVA. Statistical analysis in **f** was performed using One-way ANOVA (**P < 0.01, ****P < 0.0001, ns ≥ 0.05). Source data are provided as a Source Data file.

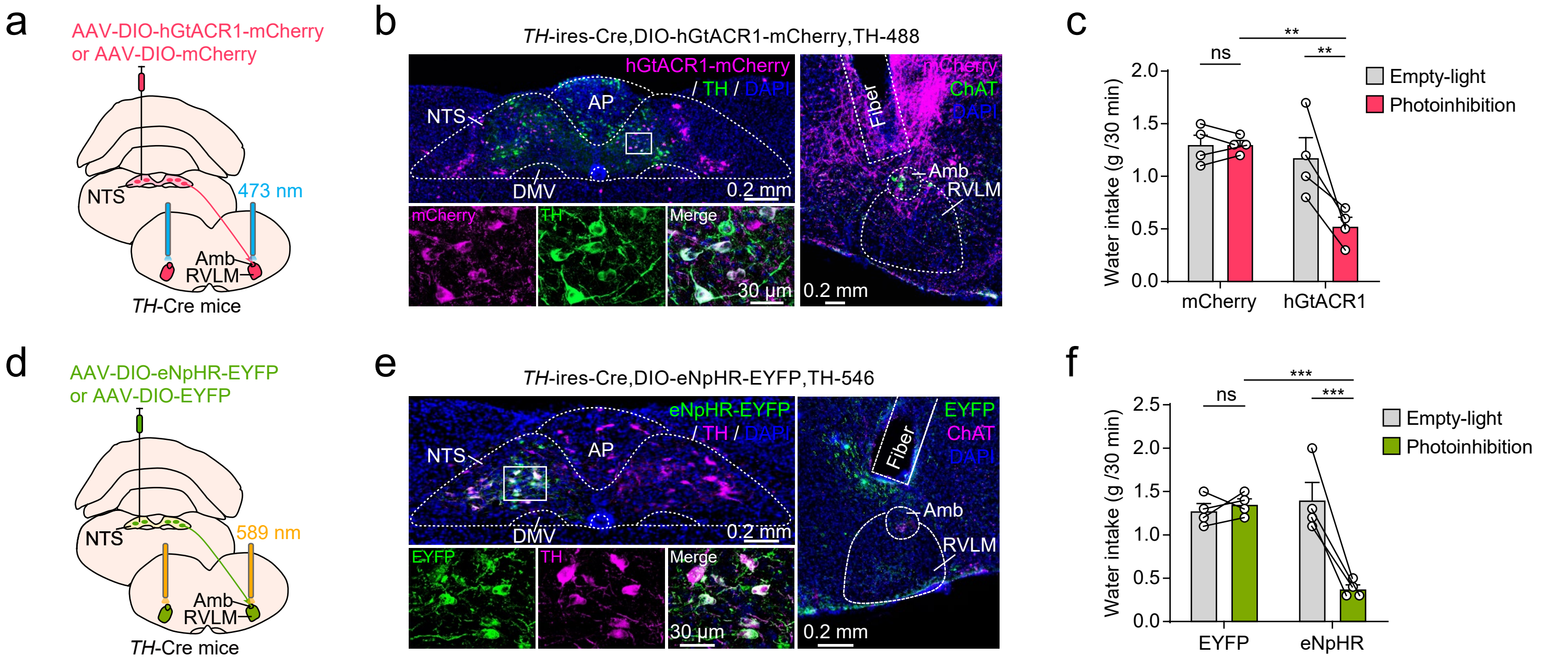

Supplementary information, Fig.11 Optogenetic inhibition axon terminals of TH+ NTS neurons projecting to Amb/RVLM significantly reduced water intake in mice.

a Schematic diagram showing injection sites of AAV-DIO-hGtACR1-mCherry (test) or AAV-DIO-mCherry (ctrl) in the NTS and optical fiber implantation in the Amb/RVLM of *TH*-ires-Cre mice.

b Example micrographs showing hGtACR1-mCherry expression in the NTS and the optical fiber track above the axon terminals in the Amb/RVLM, as well as the colocalization of hGtACR1-mCherry+ (magenta) with TH+ neurons (green).

c Water intake of mice after optogenetic inhibition of TH+ NTS-Amb/RVLM projections (n=4 mice).

d Schematic diagram showing injection sites of AAV-DIO-eNpHR-EYFP (test) or AAV-DIO-EYFP (ctrl) in the NTS and optical fiber implantation in the Amb/RVLM of *TH*-ires-Cre mice.

e Example micrographs showing eNpHR-EYFP expression in the NTS and the optical fiber track above the axon terminals in the Amb/RVLM, as well as the colocalization of eNpHR-EYFP+ (green) with TH+ neurons (magenta).

f Water intake of mice after optogenetic inhibition of TH+ NTS-Amb/RVLM projections (n=4 mice).

Data were shown as mean ± s.e.m. (error bars). The broken white lines in the section images represent boundaries of brain regions. Statistical analysis in **c** and **f** were performed using Two-way ANOVA (**P < 0.01, ***P < 0.001, ns ≥ 0.05). Source data are provided as a Source Data file.

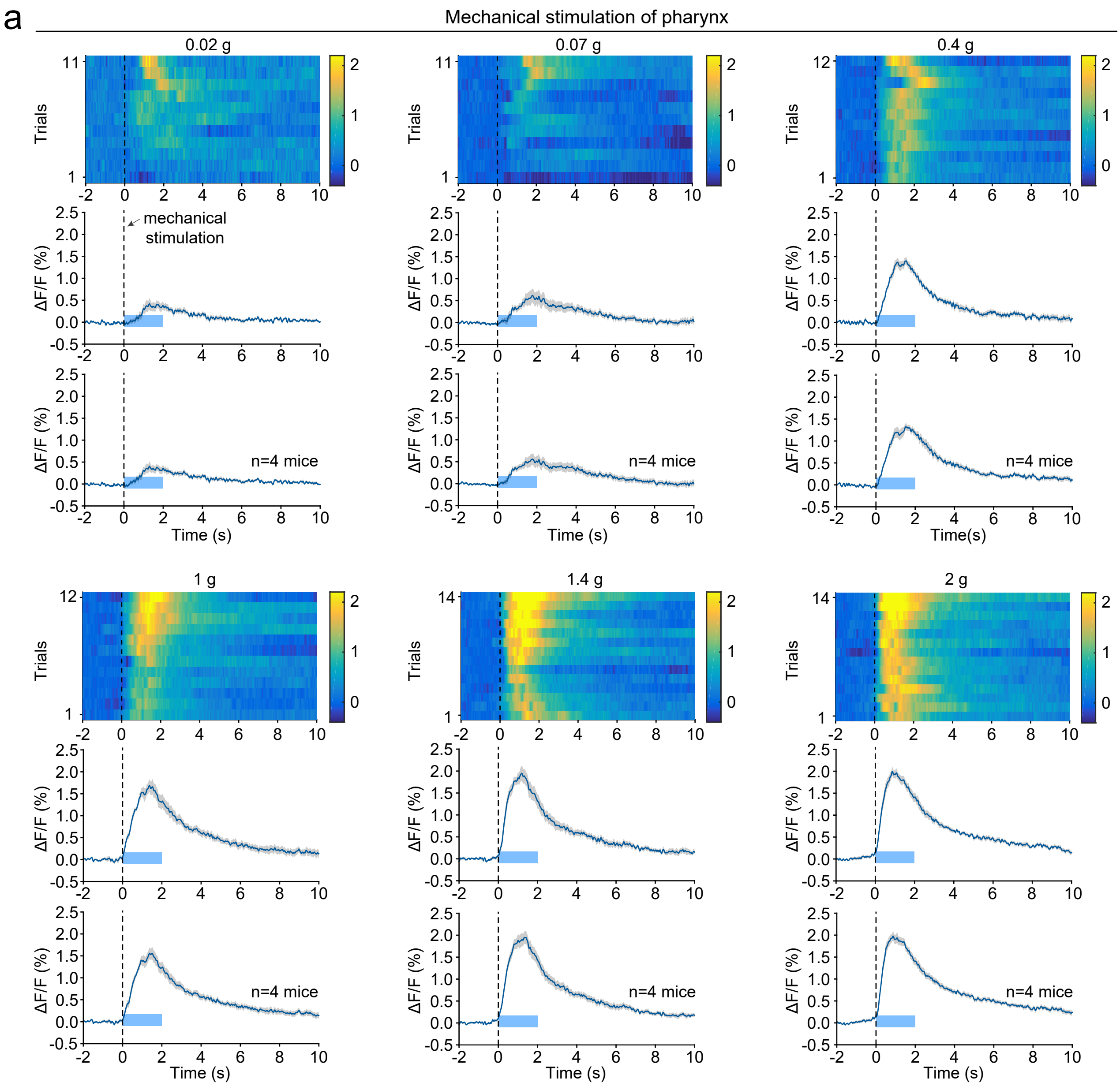

Supplementary information, Fig.12 Calcium activity of Amb/RVLM-projecting TH+ NTS neurons associated with mechanical stimulation of the pharynx.

a Heatmap and average Ca2+ transients displaying 11-14 trials of normalized GCaMP fluorescence change of Amb/RVLM-projecting TH+ NTS neurons. Calcium transients were elicited by mechanical stimulation of the pharynx (n=4 mice). Blue thick lines indicate mean and gray shaded areas indicate s.e.m.. The broken line represents the time points of the mechanical stimulation. The blue shading represents the time window for mechanical stimulation. Source data are provided as a Source Data file.

Supplementary Figure 13

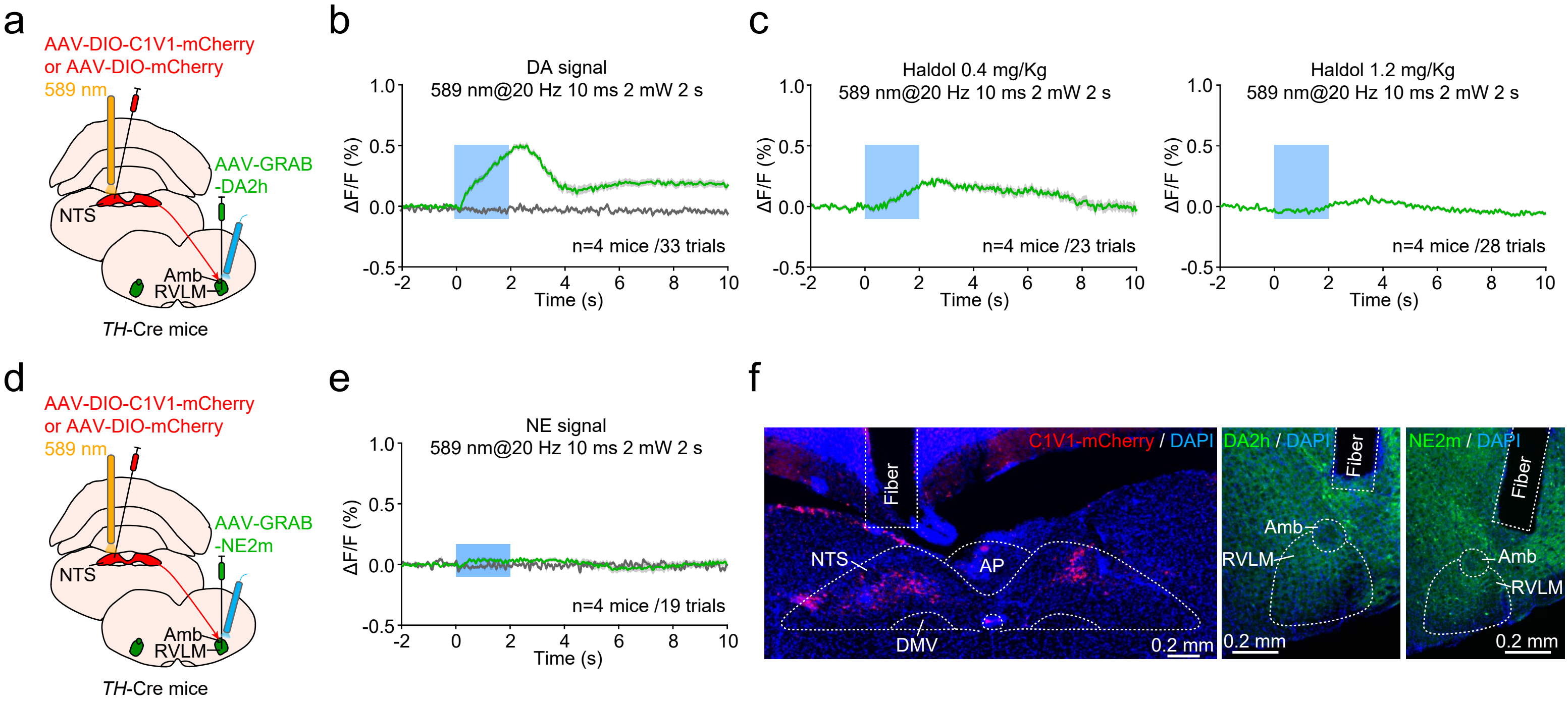

Supplementary information, Fig.13 Light-activated NTS regulated the release of dopamine in the Amb/RVLM.

a Schematic diagram showing injection sites of AAV-DIO-C1V1-mCherry (test) or AAV-DIO-mCherry (ctrl) into the NTS and AAV-GRAB-DA2h into the Amb/RVLM of *TH*-ires-cre mice, followed by optical fiber implantation above the NTS and Amb/RVLM to apply optogenetic activation and fiber photometry, respectively.

b Dopamine signal (ΔF/F) released from Amb/RVLM in 33 trials of optogenetic activation of NTS.

c DA signals (ΔF/F) released in the Amb/RVLM during light activation of NTS in 23-28 trials after treatment with the dopamine antagonist Haldol.

d Schematic diagram showing optogenetic activation of NTS and recording of NE signals in the Amb/RVLM.

e Norepinephrine signals (ΔF/F) released from Amb/RVLM in 19 trials of optogenetic activation of NTS.

f Example micrographs showing C1V1-mCherry expression and optical fiber track in the NTS (left). And GRAB-DA sensor or GRAB-NE sensor expression and optical fiber track in the Amb/RVLM (middle and right). Source data are provided as a Source Data file.

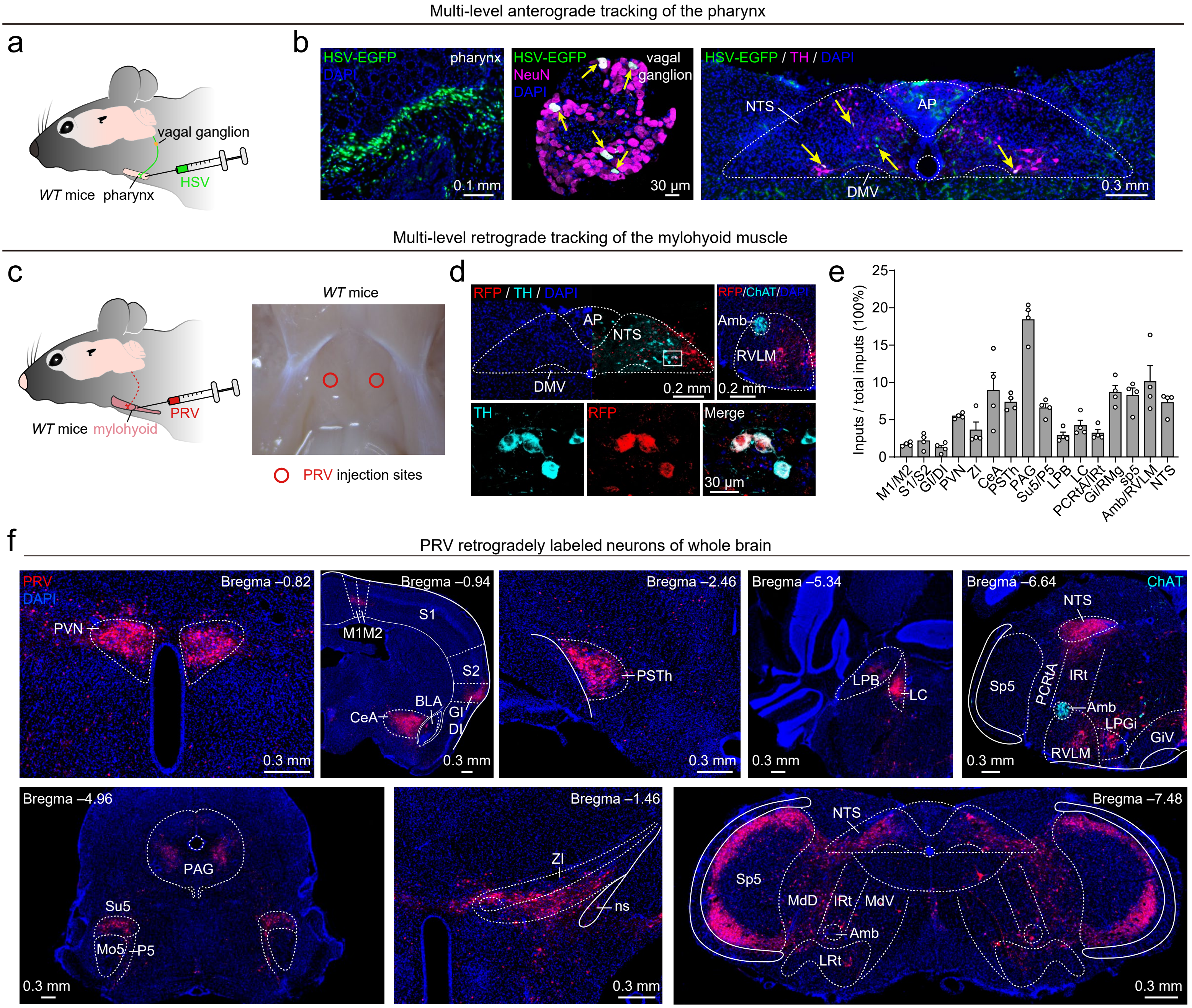

Supplementary information, Fig.14 Multi-level anterograde tracking of the pharynx and multi-level retrograde tracking of the mylohyoid muscle.

a Schematic diagram of injection HSV into pharynx in *WT* mice.

b Organ sections showing the expression of HSV-EGFP in the pharynx (left), as well as the distribution of anterograde tracing neurons (yellow arrows) in the vagal ganglia (middle), and NTS (right).

c Schematic diagram of PRV viral injection site to mylohyoid muscle in *WT* mice.

d Example coronal brain section and the magnified fields displaying the distribution of retrogradely labeled neurons of NTS and RVLM. Colocalization of retrogradely labeled neurons (red) with TH+ neurons (cyan) in the NTS (left), and Amb/RVLM was marked by ChAT+ neurons (cyan) (right).

e Diagram illustrating the proportion of cells in all retrogradely labeled nuclei (n=4 mice).

f Representative image showing the distribution of PRV retrogradely labeled neurons of whole brain in *WT* mice. These brain regions were primarily concerned with paraventricular hypothalamic nucleus (PVN), primary motor cortex (M1), secondary motor cortex (M2), dysgranular insular cortex (DI), granular insular cortex (GI), central amygdaloid nucleus (CeA), parasubthalamic nucleus (PSTh), lateral parabrachial nucleus (LPB), locus coeruleus (LC), RVLM, gigantocellular reticular nucleus (Gi), periaqueductal gray (PAG), supratrigeminal nucleus (Su5), zona incerta (ZI), NTS and spinal trigeminal nucleus (sp5). Source data are provided as a Source Data file.

Supplementary Figure 15

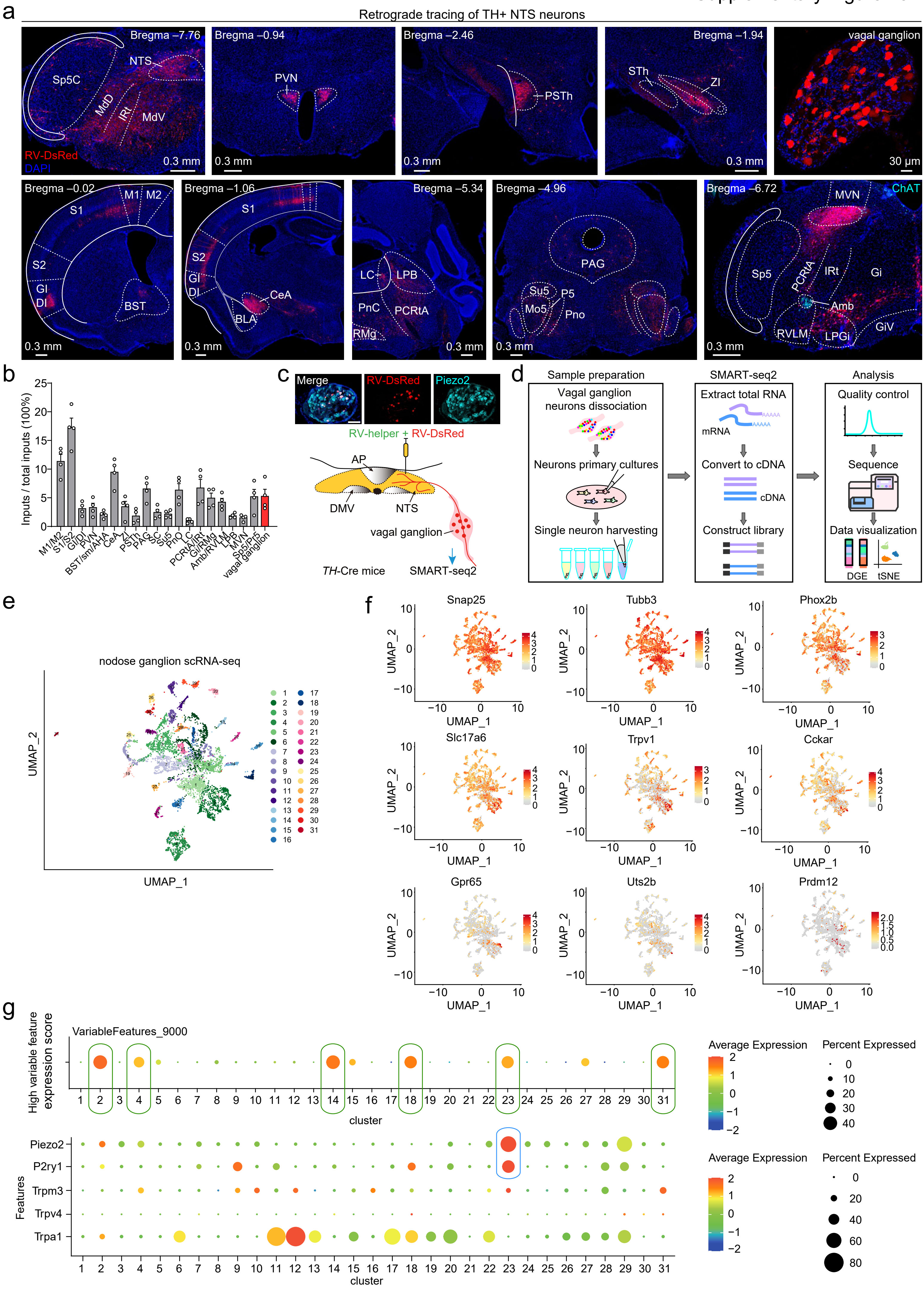

Supplementary information, Fig.15 RV monosynaptic retrograde tracing of TH+ NTS neurons.

a Representative image showing the distribution of RV retrogradely labeled neurons of whole brain in *TH*-ires-Cre mice. These brain regions were primarily concerned with spinal trigeminal nucleus (sp5), paraventricular hypothalamic nucleus (PVN), parasubthalamic nucleus (PSTh), zona incerta (ZI), primary motor cortex (M1), secondary motor cortex (M2), dysgranular insular cortex (DI), granular insular cortex (GI), primary somatosensory cortex (S1), secondary somatosensory cortex (S2), central amygdaloid nucleus (CeA), lateral parabrachial nucleus (LPB), locus coeruleus (LC), periaqueductal gray (PAG), supratrigeminal nucleus (Su5), motor trigeminal nucleus (Mo5), peritrigeminal zone (P5), rostroventrolateral reticular nucleus (RVLM), gigantocellular reticular nucleus (Gi) and vagal ganglion.

b Proportion of total DsRed+ cells in different brain regions that monosynaptically project to the TH+ NTS neurons (n=4 mice). Note that data in **b** was normalized by dividing the total number of DsRed+ cells in these brain regions.

c Example micrographs displaying the colocalization of RV-labeled neurons with Piezo2 in the vagal ganglion. Scale bar is 30 μm (top). Schematic diagrams illustrating RV-labeled cells in vagal ganglion monosynaptically projecting to the TH+ NTS neurons (bottom).

d Schematic illustration of the methodology of SMART-seq2 used in this study.

e UMAP plot showing the clustering pattern of nodose ganglion neurons.

f UMAP plot displaying the expression profiles of characteristic genes from nodose ganglion neurons.

g Transcriptome similarity analysis of DsRed labeled nodose ganglion neurons with nodose ganglion neural clusters, and dotplot of these signature scores.

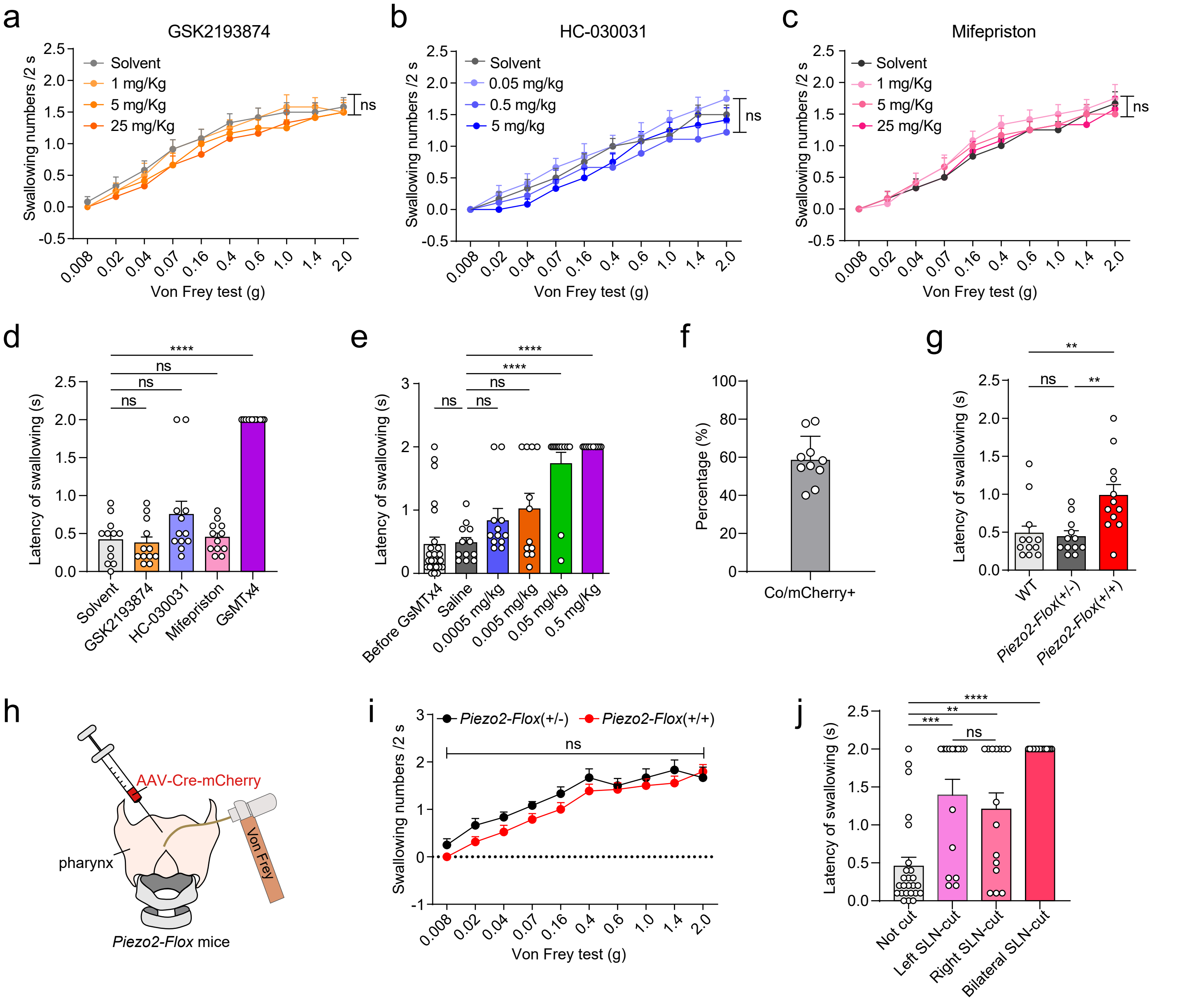

Supplementary information, Fig.16 Inhibition of pharyngeal ion channels and knockout of pharyngeal cells expressing Piezo2 had no significant impact on mechanical stimulation elicited swallowing reflex.

a–c The number of swallowings induced by mechanical stimulation after inhibition of pharyngeal TRPV4, TRPA1, and TRPM3 ion channels.

d Latency of swallowing triggered by mechanical stimulation with 0.4 g Von Frey after antagonizing the sensory ion channels in the pharynx (Piezo antagonist GsMTx4, 0.5 mg/Kg; TRPV4 antagonist GSK2193874, 1 mg/Kg; TRPA1 antagonist HC-030031, 0.05 mg/Kg; TRPM3 antagonist Mifepristone, RU-486, 1 mg/Kg; each group: n=4 mice).

e Latency of swallowing induced by mechanical stimulation with 0.4 g Von Frey after inhibiting the pharyngeal Piezo ion channel with different doses of GsMTx4 (each dose group: n=5 mice).

f Proportion of mCherry and Piezo2 co-labeled neurons among retrogradely labeled mCherry+ neurons in *Piezo2-Flox*(+/-) mice.

g Latency of swallowing elicited by mechanical stimulation of the pharynx with 0.4 g Von Frey after knocking-out of Piezo2 in vagus ganglion innervating the pharynx (n=4 mice).

h–i Schematic diagram of injection AAV-Cre-mCherry into pharynx of *Piezo2-Flox* mice (**h**). Total number of swallowing evoked by mechanical stimulation within 2 s in *Piezo2-Flox* mice with Knock-out Piezo2 expressed in pharyngeal cells (*Piezo2-Flox*(+/-) group: n=4 mice, *Piezo2-Flox*(+/+) group: n=6 mice) (**i**).

j Latency of swallowing elicited by 0.4 g Von Frey filament after SLN transection.

Data were shown as mean ± s.e.m. (error bars). Statistical analysis in **a–c** and **i** were performed using Two-way ANOVA. Statistical analysis in **d–f** and **j** were performed using One-way ANOVA (**P < 0.01, ***P < 0.001, ****P < 0.0001, ns ≥ 0.05). Source data are provided as a Source Data file.

Supplementary Table 1 Information of mouse lines

Mouse Lines		
FosCreER mice	JAX Mice	Stock No: 021882
Tac1-ires-Cre	JAX Mice	Stock No: 021877
Calb1-2A-Cre	JAX Mice	Stock No: 028532
TH-ires-Cre	JAX Mice	Stock No: 008601
Dbh-Cre	JAX Mice	Stock No: 033951
C57BL/6J	JAX Mice	Stock No: 000664
Synpr-ires-Cre	Shanghai Southern Model Biotechnology Co., LTD	NM-KI-234501
Piezo2-Flox	JAX Mice	Stock No: 027720
Ai32	JAX Mice	Stock No: 012569
Piezo2-ires-Cre	JAX Mice	Stock No: 027719
Piezo2-Cre/Ai32	Shang Congping's Laboratory	N/A

Supplementary Table 2 Information of antibodies

Antibodies		
Rabbit monoclonal Anti-Fos	Cell Signaling Technology	2250S
Chicken polyclonal anti-Tyrosine Hydroxylase	Abcam	ab76442
Rabbit polyclonal anti-Tyrosine Hydroxylase	Abcam	ab137869
Rabbit polyclonal anti-EGFP	Abcam	ab290
Chicken polyclonal anti-EGFP	Abcam	ab13970
Chicken polyclonal anti-mCherry	Abcam	ab205402
Rabbit polyclonal anti-Glutamate	Sigma	G6642
Rabbit polyclonal anti-GABA	Sigma	A2052
Rabbit polyclonal Piezo2 antibody	Novus	NBP1-78624
Rabbit polyclonal anti-RFP	Abcam	ab62341
Rabbit polyclonal anti-NeuN	Abcam	ab177487
Goat polyclonal anti-Choline Acetyltransferase	Merck-Millipore	ab144p
Goat anti-Rabbit IgG (H+L) Alexa 488	Abcam	ab150077
Goat anti-Rabbit IgG (H+L) Alexa 546	Invitrogen	A11010
Goat anti-Rabbit IgG (H+L) Alexa 633	Invitrogen	A21071
Goat anti-Chicken IgY (H+L) Alexa 488	Abcam	ab150169
Goat anti-Chicken IgY (H+L) Alexa 555	Invitrogen	A32932
Goat anti-Chicken IgY (H+L) Alexa 546	Invitrogen	A11040
Donkey anti-Goat IgG (H+L) Alexa 488	Jackson	711545152
Donkey anti-Goat IgG (H+L) Alexa 555	Abcam	ab150130
Donkey anti-Rabbit IgG (H+L) Alexa 488	Abcam	ab150073
Donkey anti-Rabbit IgG (H+L) Alexa 555	Abcam	ab150074

Supplementary Table 3 Information of reagents

Chemicals, peptides, and recombinant proteins		
4-Hydroxytamoxifen	Sigma	H6278
Clozapine N-oxide	ENZO	BML-NS105
DAPI	Solarbio	C0065
BSA, Fraction V	Beyotime	ST023
Collagenase type 2	Worthington-biochem	LS004176
Dispase® II	Roche	04942078001
Vetbond tissue adhesive	3M	1469SB
Citric acid	Sigma	C2404
Capsaicin	MedChemExpress	V900116
Sodium L-glutamate, monohydrate	Macklin	L817833
Denatonium benzoate	MedChemExpress	HY-B1146
Fast green FCF	Sigma	F7252
GsMTx4	GlpBio	GC38465
GSK2193874	Sigma	SML0942
HC-030031	Sigma	H4415
Mifepristone	Sigma	M8046
Haloperidol	Selleck	S1920
D-AP5	Tocris	0106
CNQX	Tocris	0190
Picrotoxin	Tocris	1128
TTX	Tocris	1078
4-AP	Tocris	0940
dNTP mix (10 mM each)	Thermo Fisher Scientific	R0192
KAPA HiFi HotStart ReadyMix (2×)	KAPA Biosystems	KK2602
SuperScript™ II reverse transcriptase (200 U/μL)	Invitrogen	18064014
SuperScript™ IV CellsDirect™ cDNA Synthesis Kit	Invitrogen	11750150
Power SYBR® Green PCR Master Mix	Thermo Fisher Scientific	4367659

Supplementary Table 4 Information of virus strains

Virus strains		
AAV2/9-hEF1α-DIO-mCherry-WPRE-hGH-pA	BrainVTA	PT-0013
AAV2/9-hEF1α-DIO-hChR2(H134R)-mCherry-WPRE-pA	TaiTool	S0170
AAV2/9-hSyn-DIO-hM3D(Gq)-mCherry-WPRE-pA	TaiTool	S0192-9
AAV2/9-hSyn-DIO-hM4D(Gi)-mCherry-WPRE-pA	TaiTool	S0193-9
AAV2/9-hSyn-DIO-hGtACR1-mCherry-WPRE-pA	TaiTool	S0693
AAV2/2-EF1α-DIO-eNpHR3.0-EYFP-WPRE-hGH-pA	BrainVTA	PT-0006
AAV2/9-CAG-DIO-GCaMP6s-WPRE-hGH-pA	BrainVTA	PT-0196
AAV2/9-EF1α-DIO-H2B-mCherry-WPRE-pA	BrainVTA	PT-6656
AAV2/9-DIO-H2B-EGFP-T2A-Synaptophysin-mRuby	BrainVTA	PT-9073
AAV2/2Retro-EF1α-DIO-Flp-WPRE-hGH-pA	BrainVTA	PT-0075
AAV2/9-EF1α-fDIO-EGFP-WPRE-hGH-pA	BrainVTA	PT-1047
AAV2/9-nEF1α-fDIO-hM3D(Gq)-EGFP-WPRE-pA	BrainVTA	PT-0160
AAV2/9-EF1α-fDIO-mCherry-WPRE-hGH-pA	BrainVTA	PT-0170
AAV2/9-hEF1α-fDIO-hM4D(Gi)-mCherry-WPRE-pA	TaiTool	S0336-9
AAV2/2Retro-EF1α-DIO-EGFP-WPRE-hGH-pA	BrainVTA	PT-0795
AAV2/2Retro-EF1α-DIO-GCaMP6s-WPRE-hGH-pA	BrainVTA	PT-0071
AAV2/9-EF1α-DIO-C1V1-mCherry-WPRE-hGH-pA	BrainVTA	PT-6001
AAV2/9-hSyn-GRABeen-DA2h-WPRE-pA	TaiTool	S0839
AAV2/9-hSyn-GRAB-NE2m-WPRE-pA	BrainVTA	PT-2393
AAV2/9-EF1α-DIO-H2B-EGFP-T2A-TVA	BrainVTA	PT-0021
AAV2/9-EF1α-DIO-oRVG-WPRE-hGH-pA	BrainVTA	PT-0023
RV-EnvA-ΔG-DsRed	BrainVTA	R01002
PRV-CAG-mRFP	BrainVTA	P03002
HSV-EGFP	BrainVTA	H01001
AAV2/2Retro-Plus-hSyn-Cre-mCherry-WPRE-pA	TaiTool	S0702-2RP
AAV2/9-hSyn-Cre-mCherry-WPRE-pA	TaiTool	S0702-9-H20

Supplementary Table 5 Summary of cell counting strategy

**Brain region**	**Vagal ganglion**	**NTS**	**The whole brain**
Section type	Coronal section (10 μm)	Coronal section (40 μm)	Coronal section (40 μm)
Section Range	The whole vagal ganglion	Bregma (–7.32 to –7.52)	The whole brain
Total collection	Approximately 30 sections	Approximately 5 sections	Approximately 300 sections
Sampling	To sample 1 section every 3 sections to get 6 sections evenly spaced by the whole vagal ganglion	To sample 1 section every 5 sections to get 4 sections evenly spaced by 200 μm	To sample 1 section every 5 sections to get 50 sections evenly spaced by the whole brain
