# Supplementary material for: A pharynx-to-brain sensory pathway mediates swallowing-like behavior evoked by mechanical stimulation
Source: Nat Commun. 2026 Sep 28;17:11180. doi: 10.1038/s41467-026-78007-9 (PMC13714286; doi:10.1038/s41467-026-78007-9)
Supplement: Supplementary file 2 [file 41467_2026_78007_MOESM2_ESM.pdf]

Description of Additional Supplementary Files

Supplementary Movie 1. Recording of GCaMP signal of TH+ NTS neurons and EMG signal during swallowing in mice, related to Supplementary Figure 7.

Movie S1 shows the GCaMP signal in TH+ NTS neurons (top) and the EMG signal from the mylohyoid muscle (bottom) during water-induced swallowing in TH-Cre mice under natural conditions.

Supplementary Movie 2. Optogenetic activation of TH+ NTS-Amb/RVLM pathway, related to Figure 5.

Movie S2 shows the EMG from the mylohyoid (top) and pharyngeal pressure (bottom) during optogenetic activation (473 nm, 20 Hz, 10 ms, 10 mW) of the TH+ NTS-Amb/RVLM pathway, which induces swallowing-like behavior in TH-Cre mice.

Supplementary Data 1 shows ranking of differentially expressed genes across 31 vagal ganglion neuron clusters, along with RV-tracked cluster-specific genes.

Supplementary Data 2 shows a summary of all experimental designs.

Supplementary Data 3 shows a summary of statistical analyses of all the data.
