# Supplementary material for: A pharynx-to-brain sensory pathway mediates swallowing-like behavior evoked by mechanical stimulation
Source: Nat Commun. 2026 Sep 28;17:11180. doi: 10.1038/s41467-026-78007-9 (PMC13714286; doi:10.1038/s41467-026-78007-9)
Supplement: Supplementary file 3 [file 41467_2026_78007_MOESM8_ESM.pdf]

nature portfolio

Corresponding author(s): Congping Shang

Last updated by author(s): Aug 14, 2026

Reporting Summary

Nature Portfolio wishes to improve the reproducibility of the work that we publish. This form provides structure for consistency and transparency in reporting. For further information on Nature Portfolio policies, see our Editorial Policies and the Editorial Policy Checklist.

Statistics

For all statistical analyses, confirm that the following items are present in the figure legend, table legend, main text, or Methods section.

n/a	Confirmed	
☐	☒	The exact sample size (*n*) for each experimental group/condition, given as a discrete number and unit of measurement
☐	☒	A statement on whether measurements were taken from distinct samples or whether the same sample was measured repeatedly
☒	☐	The statistical test(s) used AND whether they are one- or two-sided<br>*Only common tests should be described solely by name; describe more complex techniques in the Methods section.*
☒	☐	A description of all covariates tested
☐	☒	A description of any assumptions or corrections, such as tests of normality and adjustment for multiple comparisons
☐	☒	A full description of the statistical parameters including central tendency (e.g. means) or other basic estimates (e.g. regression coefficient) AND variation (e.g. standard deviation) or associated estimates of uncertainty (e.g. confidence intervals)
☐	☒	For null hypothesis testing, the test statistic (e.g. *F*, *t*, *r*) with confidence intervals, effect sizes, degrees of freedom and *P* value noted<br>*Give P values as exact values whenever suitable.*
☒	☐	For Bayesian analysis, information on the choice of priors and Markov chain Monte Carlo settings
☒	☐	For hierarchical and complex designs, identification of the appropriate level for tests and full reporting of outcomes
☒	☐	Estimates of effect sizes (e.g. Cohen's *d*, Pearson's *r*), indicating how they were calculated

Our web collection on statistics for biologists contains articles on many of the points above.

Software and code

Policy information about availability of computer code

Data collection	Behavioral videos were recorded using digital microscope (RWD Life Science, China, DOM-1001) and a camera (Point Grey Research, Canada).<br>Pharyngeal pressure was monitored by a pressure transducer (NL108T4) coupled to the bioelectric module amplifier (Digitimer, NL905) and 1401 Digitizer data acquisition system.<br>The EMG signals were recorded using a Microelectrode AC Amplifier (A-M Systems, Model 1800) and Spike2 software (Cambridge Electronic Design).<br>Respiration was monitored using a JZ100 force transducer (Beijing Xinhang Xingye Science and Technology Trade Co., Ltd., China).<br>Drinking water-related data was recorded using a multimodal synchronous system. A water bottle equipped with a stainless steel ball and a pressure-sensing rod was connected to a 1401 digital-to-analog converter via a lickometer module. All licking signals were acquired in real time by Spike2 software, with simultaneous video recording (50 frames/s; Point Grey Research, Canada).<br>Calcium signal were recorded using a commercialized Three colors Multi-channel optical fiber recording system (Thinker Tech Nanjing Biotech CO., Ltd).<br>For slice physiological recording, the current and voltage signals were recorded with MultiClamp 700B and Clampex 10 data acquisition software (Molecular Devices, USA).<br>The smart-seq data was provided by Illumina Inc. (USA).<br>The brain sections were imaged using a Leica Aperio Versa 8 microscope, a fully automatic slide scanning system (EVIDENT VS 200) and a laser scanning confocal microscope (Carl Zeiss LSM 980).<br>Real-time PCR data was obtained by a Fluorescence quantitative PCR instrument (Gentier 96R).
Data analysis	Behavior videos were analyzed using Corel VideoStudio Pro X8.<br>Physiological measurements (including pharyngeal pressure and EMG) were analyzed using spike2 software (Cambridge Electronic Design).

Raw data of EMG was digitized at 5000 Hz using Spike2 software.
For fiber photometry data analysis, raw data was analyzed using Spike2 (Version 10) and MATLAB R2017b. MATLAB code is presented in Zenodo at https://doi.org/10.5281/zenodo.17486163.
Microscope imaging data were analyzed using ImageScope x64, OlyVIA and ZEN2012 black/blue edition.
For slice physiological recording, the data was analyzed using Clampfit 10.4 software.
The smart-seq data was analyzed using TrimGalore, HISAT2 (vesion 2.2.1), featureCounts and Seurat package (v4.1.0).
Real-time PCR raw data was analyzed using Gentier software.
All data were replotted and statistical analyzed using GraphPad Prism 9.1 software.

For manuscripts utilizing custom algorithms or software that are central to the research but not yet described in published literature, software must be made available to editors and reviewers. We strongly encourage code deposition in a community repository (e.g. GitHub). See the Nature Portfolio guidelines for submitting code & software for further information.

Data

Policy information about availability of data

All manuscripts must include a data availability statement. This statement should provide the following information, where applicable:

- Accession codes, unique identifiers, or web links for publicly available datasets
- A description of any restrictions on data availability
- For clinical datasets or third party data, please ensure that the statement adheres to our policy

Sequencing data generated in this study have been deposited in the Genome Sequence Archive (GSA) database under accession code (CRA047891 and CRA047906). This study utilizes the single-cell database of vagal ganglion neurons previously established by our research group (PMID: 38288368) and its corresponding sequencing data (GSA accession number: CRA007790). All other data are available in the main text or supplementary materials. Source data are provided with this paper.

Research involving human participants, their data, or biological material

Policy information about studies with human participants or human data. See also policy information about sex, gender (identity/presentation), and sexual orientation and race, ethnicity and racism.

Reporting on sex and gender	NA
Reporting on race, ethnicity, or other socially relevant groupings	NA
Population characteristics	NA
Recruitment	NA
Ethics oversight	NA

Note that full information on the approval of the study protocol must also be provided in the manuscript.

Field-specific reporting

Please select the one below that is the best fit for your research. If you are not sure, read the appropriate sections before making your selection.

[x] Life sciences   [ ] Behavioural & social sciences   [ ] Ecological, evolutionary & environmental sciences

For a reference copy of the document with all sections, see nature.com/documents/nr-reporting-summary-flat.pdf

Life sciences study design

All studies must disclose on these points even when the disclosure is negative.

Sample size	No statistical method was used to predetermine the sample size.
Data exclusions	For behavioral analyses, the mice with missed viral injections/optical fiber and unhealthy conditions were excluded for experimental analyses. For whole-cell recordings, the neurons with Ra larger than 15 MΩ were excluded from further analyses, because Ra larger than 15 MΩ indicates poor patch clamp.
Replication	Behavioral experiments were replicated multiples times with independent mice, and two people independently analyzed the behavioral data. All the experimental recordings were reliably reproduced. Numbers of replicates (n) are indicated in the figure legends.
Randomization	All mice were randomly assigned to different groups.
Blinding	All investigators were blinded to group allocation during data collection and analysis.

Reporting for specific materials, systems and methods

We require information from authors about some types of materials, experimental systems and methods used in many studies. Here, indicate whether each material, system or method listed is relevant to your study. If you are not sure if a list item applies to your research, read the appropriate section before selecting a response.

Materials & experimental systems

n/a	Involved in the study
☐	☒ Antibodies
☒	☐ Eukaryotic cell lines
☒	☐ Palaeontology and archaeology
☐	☒ Animals and other organisms
☒	☐ Clinical data
☒	☐ Dual use research of concern
☒	☐ Plants

Methods

n/a	Involved in the study
☒	☐ ChIP-seq
☒	☐ Flow cytometry
☒	☐ MRI-based neuroimaging

Antibodies

Antibodies used

Primary antibodies:
Rabbit monoclonal Anti-Fos (Cell Signaling Technology, Cat# 2250S, dilution 1:500); Chicken polyclonal anti-Tyrosine Hydroxylase (Abcam, Cat# ab76442, dilution 1:1000); Rabbit polyclonal anti-Tyrosine Hydroxylase (Abcam, Cat# ab137869, dilution 1:1000); Rabbit polyclonal anti-EGFP (Abcam, Cat# ab290, dilution 1:2000); Chicken polyclonal anti-EGFP (Abcam, Cat# ab13970, dilution 1:2000); Chicken polyclonal anti-mCherry (Abcam, Cat# ab205402, dilution 1:2000); Rabbit polyclonal anti-Glutamate (Sigma, Cat# G6642, dilution 1:500); Rabbit polyclonal anti-GABA (Sigma, Cat #A2052, dilution 1:500); Rabbit polyclonal Piezo2 antibody (Novus, Cat# NBP1-78624, dilution 1:1000); Rabbit polyclonal anti-RFP (Abcam, Cat# ab62341, dilution 1:5000); Rabbit polyclonal anti-NeuN (Abcam, Cat# ab177487, dilution 1:1000); Goat polyclonal anti-Choline Acetyltransferase (Merck-Millipore, Cat# ab144p, dilution 1:250).

Secondary antibodies:
Goat anti-Rabbit IgG (H+L) Alexa 488 (Abcam, Cat# ab150077, dilution 1:500); Goat anti-Rabbit IgG (H+L) Alexa 546 (Invitrogen, Cat# A11010, dilution 1:500); Goat anti-Rabbit IgG (H+L) Alexa 633 (Invitrogen, Cat# A21071, dilution 1:500); Goat anti-Chicken IgY (H+L) Alexa 488 (Abcam, Cat# ab150169, dilution 1:500); Goat anti-Chicken IgY (H+L) Alexa 555 (Invitrogen, Cat# A32932, dilution 1:500); Goat anti-Chicken IgY (H+L) Alexa 546 (Invitrogen, Cat# A11040, dilution 1:500); Donkey anti-Goat IgG (H+L) Alexa 488 (Jackson, Cat# 711545152, dilution 1:500); Donkey anti-Goat IgG (H+L) Alexa 555 (Abcam, Cat# ab150130, dilution 1:500); Donkey anti-Rabbit IgG (H+L) Alexa 488 (Abcam, Cat# ab150073, dilution 1:500); Donkey anti-Rabbit IgG (H+L) Alexa 555 (Abcam, Cat# ab150074, dilution 1:500).

Validation

All antibodies are commonly used in the field and have been validated by the manufacturer. All antibodies listed below were validated for immunohistochemical use in rodent in previously published work: PMID: 38288368 and PMID: 26929410.

Animals and other research organisms

Policy information about studies involving animals; ARRIVE guidelines recommended for reporting animal research, and Sex and Gender in Research

Laboratory animals

The animals used in the study were described in Methods (Animals) section and Supplementary Table 1. The FosCreER (Fos-2A-iCreER) (Stock No: 021882), TH-ires-Cre (Stock No: 008601), Calb1-2A-Cre (Stock No: 028532), Dbh-Cre (Stock No: 033951), Tac1-ires-Cre (Stock No: 021877), Piezo2-Flox (Stock No: 027720), Ai32 (Stock No: 012569), Piezo2-ires-Cre (Stock No: 027719) and C57BL/6J (Stock No: 000664) mouse lines were imported from the Jackson Laboratory (JAX Mice and Services). Synpr-ires-Cre mice (NM-KI-234501) were constructed by Shanghai Southern Model Biotechnology Co., LTD. Piezo2-Cre/Ai32 mice were generated by crossing Piezo2-ires-Cre mice with Ai32 mice in Shang Congping's laboratory. Mice were housed at room temperature (23 ± 1°C) with a stable humidity (50 ± 5%) on a 12-h light/12-h dark cycle. Mice were fed with ad libitum standard chow and water, unless food-deprived overnight for 12-h for fasted experiments. Mice were at least eight weeks old at the time of virus injection.

Wild animals

No wild animals were used in this study.

Reporting on sex

Except for the initial experimental verification of the effect of gender on behavior, male mice were used for all the experiments.

Field-collected samples

No field-collected samples were used in this study.

Ethics oversight

All experimental procedures were carried out in strict compliance with protocols approved by the Administrative Panel on Laboratory Animal Care at the School of Basic Medical Sciences, The Fifth Affiliated Hospital, Guangzhou National Laboratory, Guangzhou Medical University (Guangzhou, China).

Note that full information on the approval of the study protocol must also be provided in the manuscript.

Plants

Seed stocks	*Report on the source of all seed stocks or other plant material used. If applicable, state the seed stock centre and catalogue number. If plant specimens were collected from the field, describe the collection location, date and sampling procedures.*
Novel plant genotypes	*Describe the methods by which all novel plant genotypes were produced. This includes those generated by transgenic approaches, gene editing, chemical/radiation-based mutagenesis and hybridization. For transgenic lines, describe the transformation method, the number of independent lines analyzed and the generation upon which experiments were performed. For gene-edited lines, describe the editor used, the endogenous sequence targeted for editing, the targeting guide RNA sequence (if applicable) and how the editor was applied.*
Authentication	*Describe any authentication procedures for each seed stock used or novel genotype generated. Describe any experiments used to assess the effect of a mutation and, where applicable, how potential secondary effects (e.g. second site T-DNA insertions, mosiacism, off-target gene editing) were examined.*
