# Supplementary material for: A pharynx-to-brain sensory pathway mediates swallowing-like behavior evoked by mechanical stimulation
Source: Nat Commun. 2026 Sep 28;17:11180. doi: 10.1038/s41467-026-78007-9 (PMC13714286; doi:10.1038/s41467-026-78007-9)
Supplement: Supplementary file 4 [file 41467_2026_78007_MOESM9_ESM.pdf]

nature portfolio

Peer Review File

A Pharynx-to-brain Sensory Pathway Mediates Swallowing-like Behavior Evoked by Mechanical Stimulation

Corresponding Author: Dr Congping Shang

This file contains all reviewer reports in order by version, followed by all author rebuttals in order by version.

Version 0:

Reviewer comments:

Reviewer #1

(Remarks to the Author)
Review of A pharynx-to-brain sensory pathway mediates swallowing-like behavior evoked by mechanical stimulation (Pei et al)

The objective of this manuscript is to characterize the sensory/motor neural circuit for reflexive swallows. In particular, they describe how mechanical stimulation of the pharynx evokes activity in tyrosine hydroxylase expressing neurons within the nucleus of the solitary tract (NTS) which are necessary for these reflexive swallows and sufficient to elicit swallows. The authors demonstrate that these TH+ neurons can be further defined by a projection to the ventrolateral medulla. In general, the claims made by the authors appear to be supported by the data provided and there is importance to understanding the neural circuit for these mechanically evoked reflexive swallows, but a significant lack of detail in the methods and analysis are required to accurately assess this. Furthermore, the authors do not provide data to demonstrate that the pathway they have characterized is required for 1) consumptive swallowing and 2) that the motor pathway is mediated by the TH+ NTS -> ventrolateral medulla connection. Also, the authors do not sufficiently incorporate their finding into the existing literature for the role of TH+ neurons in feeding and esophageal control nor the literature on laryngeal reflexive swallows. Without these elements, despite the immense amount of experiments / data, the significance of the manuscript for a general readership is lacking.

Major Concerns:
1) No data demonstrates that the motor reflexes evoked are related to consumptive swallowing – The presumption of the paper is that mechanical stimuli in the pharynx are important for consumptive swallowing, and that by probing a mechanosensory swallowing reflex, the authors have uncovered the neural circuit for swallowing. A central component of this circuit they have described are the TH+ NTS neurons. However, there is no data in the paper that shows consumptive swallowing is connected to the described circuit. For example, chemogenetic inhibition experiments do not measure swallowing (Fig. 3) and instead show the mice consume less water. Importantly, the mice DO consume water still and the decrease could be a result of the TH+ NTS neurons being involved in other aspects of feeding like esophageal motility as proposed in Wang, 2024. This data demonstrates that, despite inhibition, the mice can swallow. Another example is that the TH+ fiberphotometry signals do not precede swallows but instead follow the swallow (Fig. 4), inconsistent with a model in which they trigger a swallow. The authors either need to show the importance of this circuit for consumptive swallowing or re-frame the paper for a modulatory circuit for mechanosensory reflexive swallows in anesthetized animals. This is a major issue.

2) The conclusion that the pathway is Piezo2 vagal fibers -> TH+ NTS ->RVLM (Fig. 8k) – This conclusion is a model that stems from the data and not a conclusion that can be made. For example, characterizing that the TH+ NTS neurons that project to the RVLM in the swallowing behavior (Fig. 5 / 6) does not demonstrate that this projection is the motor pathway to induce a swallow. None of the experiments conducted are modulating the synaptic release exclusively in the RVLM, and, furthermore, the mylohyoid muscle used to measure swallowing has motor neurons within the pontine CN5. While the authors can conclude that the TH+ NTS neurons with the RVLM projections are specialized for the swallow behavior (compared to those that project to the PBC), they cannot conclude that this is the motor pathway.

3) The authors fail to cite and integrate their findings with existing literature – The authors are studying TH+ NTS neurons which have recently been described to be involved in esophageal pressure sensing and motility (Wang, 2024) and assays

that are used to confirm swallowing (the twine assay) could be a consequence of inducing esophageal motility. Also, activation of the SLN in Piezo2-Cre; Ai32 mice has been done in Prescott, 2020 and it did not evoke swallowing (contrast to Fig. 7). TH+ neurons are also Dbh+ (one example in Wang, 2024), so why does Dbh-Cre not label these swallowing neurons and why does the sequencing data provided not show that TH+ neurons are not Dbh+ (Supplemental Fig. 3). It is important for the authors to properly integrate their study with others.

4) Overall, there is a poor description of the primary data and the methodology. This makes it challenging to understand what the manipulations actually are / look like. Examples of this and they are noted below.

• Von Frey experiments - Figure 1b shows the external throat movements that were monitored to quantify a swallow, however the methods state that the pharynx had been surgically opened from the ventral side, EMG electrodes implanted in the mylohyoid muscle and VF filaments applied to the pharyngeal mucosa. These two things are incompatible. If external movements were used as a measure of swallow for some experiments and EMG +/- pharyngeal pressure for others, then this needs to be clearly stated for each experiment. Additionally, a similar photo series showing the placement of the VF filaments in the pharynx, the pressure transducer etc would be very informative to the reader on how the experiment was performed. Given that these experiments are central to much of the paper, this clarification is essential. The stimulation site could be further explained by referencing anatomical landmarks in the pharynx e.g. proximity to epiglottis, hard palate.

• Were any numerical or analytical thresholds used to determine swallowing? From the representative traces presented (Figure 1C) it is clear where the events are but since the swallowing magnitude varies with stimulus weight (Figure 1G), it is important to know how a swallow was defined, particularly at lower stimulus weights. Additionally, transient apnea is mentioned as a defining feature of a swallow but it is unclear from the main text and methods how breathing was being recorded in this experiment. It is also unclear how 'tracheal peristalsis' is related to swallowing (or what it is), how it was quantified and where this quantification is in the paper.

• When conducting the Von Frey experiments, how many times is the pharynx probed at each pressure step and are there responses at each step. For example, in Fig. 3K, it is reported that 1-1.5 swallows occur per 30 probe trials. Does this mean that only 1 out of every 30 trials has a swallow? Or one swallow occurs each trial? The overall reproducibility of the behavior within a single animal is not shown and instead the panels in each figure are displayed as a mean of the mean for each animal.

• The parameters of stimulation to evoke cFOS are not adequately described in the text or methods. What was the duration of the stimulation? Was it continuous or intermittent? These are described for the FosTRAP experiment and so I presume they are the same but this needs to be stated. The arytenoid cartilage exposes the larynx and not the pharynx (line 138).

• The nucleus ambiguus is not positively identified with a marker like ChAT.

• No control cFOS expression data is shown in the supplemental figure and the proper TRAP control should be TRAP-ChR2 animals with implants that are not mechanically probed. The mCherry control is only accounting for light induced effects but not the specificity of Fos expression.

• If the swallow is changing breathing, then it is hard to understand how the respiratory rate is not impacted by light in the optogenetic experiments throughout the paper. How breathing is annotated and quantified is not described.

• In the experiment summarized in Figure 2a the authors demonstrate that optogenetic activation of NTS neurons in TH-cre but not lines for other transcriptomically distinct cell types. Figure Supp. 4 a shows the placement of fibers for this experiment which seems to vary across the medial-lateral axis. The authors should describe any exclusion criteria that were applied for misplaced fibers. Secondly, of the included mice, it would be informative for the reader to see a plot of fiber placements medial-lateral, rostral-caudal to see that fiber placement was equivalent between cre lines and these clusters can be correctly excluded as candidate swallow-evoking neuron populations. One reason why this is confusing is that Calb1 is expressed by all TH+ neurons (Fig. Supp. 4) yet it does not induce swallowing. And, Dbh has been reported to be expressed by TH+ neurons but does not induce swallowing. These results could simply be technical and not biological.

• In Figure 3f-i, water-restricted mice had drinking behavior quantified after chemogenetic inhibition of TH+ NTS neurons. CNO injected mice drank less water in 30 minutes while spending an equivalent amount of time at the spout than when injected with saline and non-DREADD expressing controls. It is not clear from the methods, figure legend or text how many of the parameters reported in Figure 3 were calculated. For the drinking experiment the methods merely state that behavior was observed from a camera. What criteria were used to determine duration of water intake (Figure 3h). I don't know what 'Times of drinking water' means or how it was calculated (Figure 3i). Given the importance of this experiment to the overall conclusions of the paper, this must be better explained.

• Figure 3m and 6l, it is possible that the impact of citric acid and high salt is due to adding fluid or pressure onto the pharynx, just like water. It seems that this result could simply be explained by mechanosensation.

• The fiber photometery experiment in figure 4 d-g is not adequately described. The methods need to include details like the creation of the oral infusion catheter and the performance of the infusion. At the moment, the reader must guess what the authors have done which makes the interpretation of the data dubious. I assume the authors infused a small volume of water into the mouth (how much? what solution?), recorded the mouse swallowing and aligned the photometry signal to the swallow event however the methods are far too vague. Further clarification is needed on what the infused volume was? what

was the latency from infusion to swallow? did all infusions result in a swallow? how many infusions were considered per mouse? which EMG features were used to define swallow onset?

• There is no histology to show the injection site of HSV in the pharynx and most HSV expression in the brainstem is outside of the NTS and many of the neurons within the NTS are TH negative.

• More detail and histology is required about the injection of pharmacological agents into the pharynx. Were they injected into the same 4 sites as the virus detailed in the methods? How were effective concentrations for the drugs calculated and confirmed? How were the injection sites validated?

• Interpretation of the nerve transection results in Figure 8 are once again complicated by unclear reporting in the methods, main text and figure. In figure 8a it appears that the SLN is being illuminated proximal to the transection site. Given the result I find it surprising that illuminating a transected nerve proximal to the cut site would show any effect of cutting the nerve. Was this the case or was the light delivered distal to the transection site?

• Supplemental Figure 12 shows very little data, like what genes are used to define the vagal clusters?

• Figure 7 does not convincingly show a knockout of Piezo2. In fact, panel M still shows immunostaining for Piezo2 in the neurons that express Cre.

Reviewer #2

(Remarks to the Author)
The manuscript proposes a vagal, Piezo2-dependent sensory pathway from the pharynx to TH+ neurons in the nucleus of the solitary tract (NTS) that, via projections to the Ambiguus/RVLM complex, triggers swallowing-like behavior. The authors combine Von Frey–evoked swallowing under anesthesia, EMG and pharyngeal pressure readouts, c-Fos mapping, opto/chemo-genetics, fiber photometry, trans-synaptic tracing, SLN transections, pharmacology (GsMTx4), and a retro-AAV Piezo2 knockout strategy. Overall, the study addresses an important and understudied aspect of swallowing neurobiology (mechanosensory triggering). However, there are some shortcomings that need to be addressed.
1. GsMTx4 is a broad blocker of stretch-activated channels and not selective for Piezo2. The observed suppression after GsMTx4 supports a role for mechanosensitive channels but does not isolate Piezo2. Conversely, the other antagonists used (e.g., mifepristone for TRPM3) have known off-targets. The pharmacology thus supports “mechanotransduction dependence” more than “Piezo2 specificity.” The genetic KO strengthens the case but is partial (~50% mRNA knockdown) and leaves residual responses.
2. While SLN transection severely impairs evoked swallowing, the literature and introduction note roles for glossopharyngeal (GPN) afferents in pharyngeal swallowing. The study does not test GPN transection or combined lesions; thus, the exclusive primacy of SLN Piezo2 afferents in pharyngeal mechanosensation remains suggestive rather than definitive.
3. Many mechanosensory tests and opto-evoked outputs are under anesthesia. Although the authors include head-fixed water-evoked swallowing and projection-specific photometry, the key causal tests (e.g., terminal opto at Amb/RVLM driving swallows) are anesthetized. Demonstrating necessity/sufficiency in awake animals would further validate physiological relevance. A suggestion: test opto-inhibition of TH+ NTS or TH+ NTS→Amb/RVLM during awake voluntary drinking to show necessity under natural conditions (e.g., stGtACR2 or ArchT).
4. Chemical stimuli are applied to the larynx, whereas mechanical stimuli are applied to the pharynx; both converge on TH+ NTS→Amb/RVLM. The conclusion of a unified “shared circuit” is attractive but could be strengthened by matching stimulus sites (e.g., pharyngeal chemical stimuli) and/or by directly recording upstream afferent population responses.
5. How do you know that evoked events meet clinical swallowing criteria rather than generalized upper-airway reflexes?
Minor comments
1. The abstract states that “Either ablation of TH+ NTS neurons or Piezo2 knockout in vagal sensory neurons both could abolish swallowing reflex” but the Results show chemogenetic inhibition (hM4Di) and not true ablation. If ablation data exist, they are not presented; if not, the abstract should be revised to “inhibition” to avoid overstatement.
2. The causality language is too strong. Please tone down.

Reviewer #3

(Remarks to the Author)
In this manuscript by Dr. Pei and colleagues, the authors first stimulated the pharynx using von Frey filaments to trigger swallowing, which induced c-Fos expression in the NTS. Optogenetic or chemogenetic activation of TRAPed NTS neurons also elicited swallowing. The study then identifies these neurons as TH-positive NTS neurons. Inhibition of TH-positive NTS neurons impaired both mechanically and chemically induced swallowing. The authors further demonstrate that TH-positive neurons are glutamatergic and excitatory, and that they are activated during water-induced swallowing. They next examined downstream projections of TH-positive neurons and show that the Amb/RVLM pathway mediates swallowing. The nodose–NTS circuit is mapped, revealing that TH-positive neurons receive input from Piezo2-positive nodose neurons. Lastly, the authors show that Piezo2 deletion reduces mechanically induced swallowing, and optogenetic stimulation of Piezo2-positive fibers triggers swallowing.
An impressive amount of data is presented in this manuscript. Much of the work appears technically solid, with appropriate controls and quantification, and the experiments are generally designed with care.
My principal concern relates to conceptual novelty. Many of the major findings concerning TH-positive NTS neurons in

swallowing have already been reported, notably in Wang et al., 2024, from Dr. Ling Bai's group. A substantial portion of the current study reproduces previously published observations. While it is reassuring to see these results replicated with rigorous experimental design, the conceptual advance is limited. The demonstration that stimulation of TH-positive neurons triggers swallowing and that their inhibition abolishes swallowing is new, but largely expected. The TH→Amb projection is indeed novel. The role of Piezo2 in mechanical swallowing is new as well but appears inconsistent with earlier reports and requires further validation. Overall, the authors should more clearly acknowledge what is already known and highlight what is genuinely new in their work. Additional major concerns are listed below.

1. It is difficult to reconcile the claim that mechanical stimulation of the pharynx activates most TH-positive neurons, given that this is a large and heterogeneous NTS population. Are there spatial patterns along the rostral–caudal axis?
2. The results described in Fig. 3g–i are unclear. The number of swallowing events should be explicitly monitored and quantified.
3. The data in Fig. 4b are confusing. Were the ChR2-mCherry-negative NTS neurons patched at random? What proportion of mCherry-negative neurons receive direct input from mCherry-positive TH-positive neurons? Furthermore, while these electrophysiological experiments are elegant, the neurochemical identity of TH-positive NTS neurons has already been well characterized in previous profiling studies, such as in Want et al 2024, Su et al 2024, Ludwig et al 2021.
4. The findings described in Fig. 4e–f have been previously reported in Bai and Knight.
5. The identification of 31 clusters of vagal sensory neurons requires clarification. It is unclear which dataset was used for this analysis and what gene signatures are expressed by the RV-traced neurons. The meaning of "expression level of the DsRed-labeled neurons" should be explicitly defined.
6. I have significant concerns regarding the data in Fig. 7. Given the limited efficiency of AAV-retro targeting, and the fact that Piezo2-positive nodose neurons innervate multiple organs, it is not plausible to observe a 50 percent reduction in Piezo2 RNA expression at the whole-ganglion level. The specificity of the Piezo2 antibody also requires demonstration. Moreover, as shown both in earlier studies and in this manuscript, nodose neurons responding to chemical stimuli (water, acid) also project to the pharynx and can trigger swallowing. It is therefore difficult to reconcile the finding that all retrogradely labeled nodose neurons are Piezo2-positive.
7. The data in Fig. 8 are inconsistent with Prescott's 2020 Cell paper. High-intensity light alone can activate neurons. A no-Cre control group is required.
8. The overall manuscript flow would benefit from revision. Several sections appear to have been added later and are not fully integrated into the earlier narrative.

Version 1:

Reviewer comments:

Reviewer #1

(Remarks to the Author)
Regarding nomenclature:

• Comments regarding the ventral swallowing group being the Amb/RVLM are inaccurate. An example of this is below in quotations. The nucleus ambiguus (Amb) is a collection of motor neurons that project through the IX, X, and XI cranial nerves. The RVLM is connected to sympathetic activity and cardiovascular control. The ventral swallowing group (VSG) is located dorsal to the nucleus ambiguus or even medial to it in the reticular formation. This is also how the VSG is described in the first citation of this manuscript.

While the functional data, like ChR2 stimulation of axons innervating the nucleus ambiguus in Fig. 5d shows the NTS Th+ neurons innervating this anatomical location are sufficient to induce a swallow, the role of the RVLM is unclear. One possibility is that these axons are in the brainstem breathing control circuit (the Bötzinger and preBötzinger Complex) and serve to coordinate breathing with swallow.

"The Amb/RVLM complex, located in the VSG of the brainstem, is responsible for transmitting sensory information from the DSG to the next level of motor neurons, thereby coordinating the initiation of swallowing."

Regarding swallowing during natural consumption:

• The concerns regarding the role of the Th+ neurons in consumptive swallowing remains. The data provided in the rebuttal is a simple bar graph with methodology described. Is there a reason that more data was not presented or included in the manuscript? At this time, the conclusion that consumptive swallowing is impacted is that the drinking bout size and duration are unchanged, but even the raw data to make these quantifications like in Fig. 3h and i cannot be found within the manuscript. It is challenging to evaluate the validity of this conclusion: "(page 12, line 271) which to some extent reflectd a dysfunction in swallowing".

The most definitive conclusion from this experiment is that despite inhibition of the NTS Th+ neurons, the mice still CAN swallow during natural consumption.

• "Furthermore, a apparent increase in GCaMP fluorescence at the onset of swallowing was observed when fixed-head mice initiated swallowing induced by slow manual water delivery (Supplementary Fig. 7b, movie 1)."

How was this concluded to be a swallow? Was the animal even consuming fluid?

Regarding Fiberphotometry conclusions:

• Supplemental Fig. 7 shows that the Th+ neurons have a Fiberphotometry signal during a swallow that is induced by slow perfusion of citric acid, salt, or water into the pharyx / larynx. Unlike the model of Piezo2 fibers exciting the Th neurons when the pharynx is mechanically stimulated, this data indicates that the Th+ neurons are activity that is recruited by the action of a swallow. While this does not mean that cannot be important for swallowing, it is challenging to understand how they become activated by CA, high salt, and water as proposed.

Once again, the Fiberphotometry signals always occur after the dashed line (swallow onset) as opposed to preceding them.

Regarding Piezo2 channel Rhodopsin:

• The additional discussion section is incorrect regarding the transgenes used in Prescott 2020. The methods of the paper clearly state that the transgenic lines are Piezo2-EGfp-ires-Cre (027719) and loxP-ChR2 (012569). This loxP-ChR2 line is Ai32. I do not understand the explanation provided in the discussion. This should be removed.

Regarding GtACR1 terminal inhibition:

• This channel has been shown in multiple papers to elicit synaptic release when light is provided to the axon terminals. Other optogenetic tools such as NpHR or OPN3 or PPO are the accepted methodologies for terminal inhibition. While the data provided is consistent with the hm4di results, the lack of validation of this method for terminal inhibition weakens the meaning of this data. It is hard to imagine that this ventral brainstem region is the premotor source of swallowing.

Reviewer #2

(Remarks to the Author)
The authors have addressed my comments.

Reviewer #3

(Remarks to the Author)
The authors performed a large number of new experiments and reasonably addressed most of my concerns. Although there is still a novelty concern that the current manuscript seems a combination of Want et al 2024 and Prescott et al 2020, the data provided is though and solid, and could complement the field. I just have a few minor comments left.
First, in line 88, This is not from Prescott team - Prescott's work in the Liberles lab.
Second, the first paragraph in line 246, this is not necessity. It should be moved to the last section.
Third, in line 308, it should be mentioned that this is 8/17 recorded neurons.
Fourth, in the paragraph starts from line 342, functional divergence was tested between Th-LPB and Th-NA pathways. However, it is not mentioned whether they are the same neurons or different neurons. Vice versa, the authors mentioned that Th-NA and Th-Arc are different neurons, yet whether the Th-Arc pathway also contributes to swallowing has not been tested.
Fifth, the newly added experiment described in line 411 should be moved to the next section, as it describes the necessity.
Sixth, in line 524, it is still not clear what this single cell data shows. The authors should show the integrated UMAP plot of these neurons and all NTS sequenced, and show representative gene expressions including Piezo2, Trpv1, and etc to guide the readers about the characteristics of the relevant nodose neuron types.

Version 2:

Reviewer comments:

Reviewer #1

(Remarks to the Author)
I have no further comments to address.

Reviewer #3

(Remarks to the Author)
The authors have additional experiments and have satisfactorily addressed most of my previous concerns. Although I continue to believe that the conceptual novelty is primarily in the integration of previously established findings rather than the discovery of an entirely new principle, the study now provides a comprehensive and technically rigorous characterization of a neural circuit mediating mechanically evoked swallowing, which will be a valuable contribution to the field.

I have one remaining comment mainly concern the interpretation of the single-cell analysis results. In the TSNE plots shown in Supplemental Figure 15, there are numerous very small clusters. Very likely, many of them are not neuronal populations. However, it is difficult to know which clusters really represent nodose neurons or jugular neurons. The authors should show Phox2b expression (nodose), Prdm12 (jugular marker), Slc17a6 (pan), Snap25, Tubb3, Trpv1, Cckar, Gpr65, Uts2b, and other markers used in Kupari, Prescott, Bai, Zhao, and other papers to guide the readers about vagal sensory neuron subtypes. I strongly recommend to replace TSNE plot with UMAP plot, which is the current standard way for single-cell data visualization. I also suggest to subset only neuronal populations for better visualization. Currently, it is difficult to identify cluster 2, 4, 14, 18, and 23, which are enriched clusters from labeled cells, from panel f. The whole idea of this figure is to identify the molecular signatures of retrogradely labeled vagal neurons and current figures cannot serve the role.

Open Access This Peer Review File is licensed under a Creative Commons Attribution 4.0 International License, which permits use, sharing, adaptation, distribution and reproduction in any medium or format, as long as you give appropriate credit to the original author(s) and the source, provide a link to the Creative Commons license, and indicate if changes were made.
In cases where reviewers are anonymous, credit should be given to 'Anonymous Referee' and the source.
The images or other third party material in this Peer Review File are included in the article's Creative Commons license, unless indicated otherwise in a credit line to the material. If material is not included in the article's Creative Commons license and your intended use is not permitted by statutory regulation or exceeds the permitted use, you will need to obtain permission directly from the copyright holder.
To view a copy of this license, visit https://creativecommons.org/licenses/by/4.0/

Response to Reviews

Manuscript ID: NCOMMS-25-80988-T

Title: A Pharynx-to-brain Sensory Pathway Mediates Swallowing-like Behavior Evoked by Mechanical Stimulation

We sincerely appreciate the positive and helpful evaluations provided by the reviewers. Guided by these thoughtful comments, we have completed an extensive set of additional experiments to address specific concerns. We also made extensive revisions to the manuscript and incorporated content related to the comments of each reviewer. A revised version of the manuscript with tracked changes (highlighted in red) has been uploaded as a separate file. The detailed changes and our point-by-point responses to each of the Reviewers' questions and comments are presented below.

Contents:

Response to Reviewer #1: Page 02-36

Response to Reviewer #2: Page 37-45

Response to Reviewer #3: Page 46-57

Reviewer 1

Review of A pharynx-to-brain sensory pathway mediates swallowing-like behavior evoked by mechanical stimulation (Pei et al). The objective of this manuscript is to characterize the sensory/motor neural circuit for reflexive swallows. In particular, they describe how mechanical stimulation of the pharynx evokes activity in tyrosine hydroxylase expressing neurons within the nucleus of the solitary tract (NTS) which are necessary for these reflexive swallows and sufficient to elicit swallows. The authors demonstrate that these TH+ neurons can be further defined by a projection to the ventrolateral medulla. In general, the claims made by the authors appear to be supported by the data provided and there is importance to understanding the neural circuit for these mechanically evoked reflexive swallows, but a significant lack of detail in the methods and analysis are required to accurately assess this. Furthermore, the authors do not provide data to demonstrate that the pathway they have characterized is required for 1) consumptive swallowing and 2) that the motor pathway is mediated by the TH+ NTS-ventrolateral medulla connection. Also, the authors do not sufficiently incorporate their finding into the existing literature for the role of TH+ neurons in feeding and esophageal control nor the literature on laryngeal reflexive swallows. Without these elements, despite the immense amount of experiments/data, the significance of the manuscript for a general readership is lacking.

We appreciate your thoughtful assessment and valuable suggestions, which have helped us identify key areas for strengthening the manuscript. We appreciate the recognition that "the claims made by the authors appear to be supported by the data provided" and will address the noted concerns regarding methodological detail, integration with existing literature, and additional experimental evidence to enhance the significance of our study.

Major Concerns:

1) No data demonstrates that the motor reflexes evoked are related to consumptive swallowing – The presumption of the paper is that mechanical stimuli in the pharynx are important for consumptive swallowing, and that by probing a mechanosensory swallowing reflex, the authors have uncovered the neural circuit for swallowing. A central component of

this circuit they have described are the TH+ NTS neurons. However, there is no data in the paper that shows consumptive swallowing is connected to the described circuit. For example, chemogenetic inhibition experiments do not measure swallowing (Fig. 3) and instead show the mice consume less water. Importantly, the mice DO consume water still and the decrease could be a result of the TH+ NTS neurons being involved in other aspects of feeding like esophageal motility as proposed in Wang, 2024. This data demonstrates that, despite inhibition, the mice can swallow. Another example is that the TH+ fiber photometry signals do not precede swallows but instead follow the swallow (Fig. 4), inconsistent with a model in which they trigger a swallow. The authors either need to show the importance of this circuit for consumptive swallowing or re-frame the paper for a modulatory circuit for mechanosensory reflexive swallows in anesthetized animals. This is a major issue.

We sincerely thank you for raising this critical and insightful point, which correctly identifies the core issue regarding the physiological relevance of our findings. In response to this concern, we have implemented the following modifications:

Question 1：There is no data in the paper that shows consumptive swallowing is connected to the described circuit. For example, chemogenetic inhibition experiments do not measure swallowing (Fig. 3) and instead show the mice consume less water. Importantly, the mice DO consume water still and the decrease could be a result of the TH+ NTS neurons being involved in other aspects of feeding like esophageal motility as proposed in Wang, 2024. This data demonstrates that, despite inhibition, the mice can swallow.

Response:

(1) The swallowing process is divided into three phases, including the oral phase, pharyngeal phase, and esophageal phase. The scientific hypothesis we propose is that TH+ NTS neurons mediate the pharyngeal swallowing reflex triggered by mechanical stimulation of the pharynx. Our further experimental data was consistent with and did not conflict with the observation by Wang, 2024 that TH+ NTS neurons were involved in esophageal motility. In fact, Wang, 2024 did not mention esophageal motility, instead, it placed greater emphasis on esophageal mechanical distension. It is precisely during the oral phase of swallowing that, after

mastication, food boluses are propelled into the pharynx by the tongue and other structures, leading to esophageal mechanical distension. This distension is perceived by TH+ NTS neurons, which then regulate esophageal motility and trigger the swallowing reflex. Inactivation of these neurons impairs the perception of mechanical stimulation by food boluses entering the pharynx, even if it does not affect mastication and propulsion of oral-phase food into the pharynx. Although the bolus entering the pharynx produces mechanical stimulation to the pharynx, the pharyngeal phase of swallowing cannot proceed, leading to dysphagia. Furthermore, the intensity of Von Frey stimulation of the pharynx simulated the size of food boluses, as evidenced by the TH+ NTS neurons dynamics recorded (Fig. 4g and Supplementary Fig. 7a), which was consistent with the conclusion of Wang, 2024. The drinking experiment also confirmed that TH+ NTS neurons were unrelated to drinking motivation, which was consistent with the phenomenon observed by Wang, 2024.

This point is thoroughly discussed in the revised manuscript (Lines 755-797).

(2) In the chemogenetic inhibition experiments, we measured swallowing behavior in anesthetized mice (Fig. 3j). The results indicated that after chemogenetic inhibition of TH+ NTS neurons, the swallowing reflex was impaired, manifesting as a requirement stronger mechanical stimulation to evoke swallowing or showing a significant reduction in the number of swallowings elicited under the same stimulus intensity.

(3) In the drinking behavior assays following chemogenetic inhibition, we quantified total water intake, number of drinking bouts, and total drinking duration. The data revealed that while total water consumption decreased, neither the number of drinking bouts nor the total drinking time were affected. We acknowledge that using reduced water intake alone as a proxy for impaired swallowing is not rigorous. To directly measure swallowing behavior, we have performed new experiments in awake, freely behaving mice. We directly measured swallowing kinematics via submental electromyography (EMG) of the mylohyoid muscle during spontaneous drinking, both before and after chemogenetic inhibition of TH+ NTS neurons. Chemogenetic inhibition of TH+ NTS neurons reduced water intake in mice, as well as the number of swallowing during free drinking periods (Response Document Figure 1).

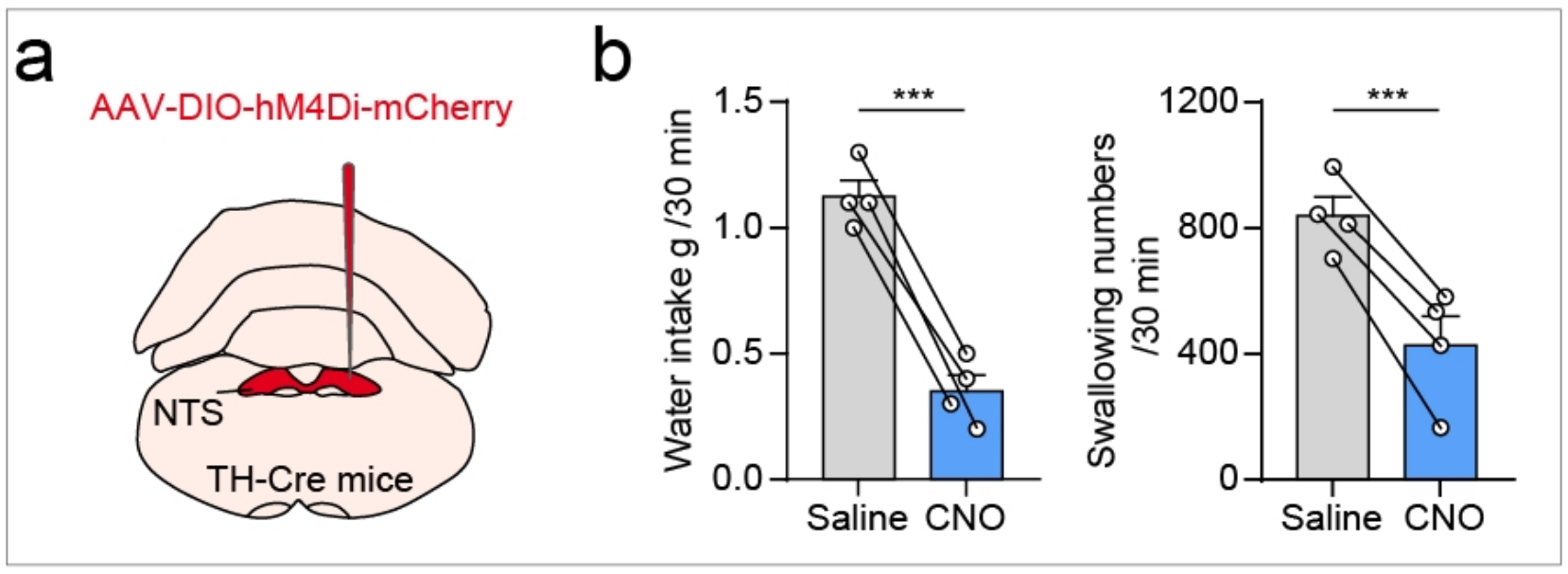

Response Document Figure 1 | Chemogenetic inhibition of TH+ NTS neurons reduced swallowing frequency during free drinking. **a** Schematic diagram showing injection site of AAV-DIO-hM4Di-mCherry in the NTS of *TH*-ires-Cre mice. **b** Water intake and swallowing frequency within 30min in mice following chemogenetic inhibition of TH+ NTS neurons (n=4 mice).

(4) We propose that TH+ NTS neurons represent a key, but not exclusive, neuronal population involved in the swallowing reflex. Therefore, the inhibition resulted in an attenuation, rather than a complete abolition, of swallowing behavior. Meanwhile, since the oral phase was not affected, water or food could enter the oral cavity normally. However, accumulation in the oral cavity would naturally overflow from it, which may also be one of the reasons for continued water consumption observed after the deactivation of TH+ NTS neurons.

Question 2: Another example is that the TH+ fiber photometry signals do not precede swallows but instead follow the swallow (Fig. 4), inconsistent with a model in which they trigger a swallow.

Response:

In Fig. 4g (new Supplementary Fig. 7b), the dashed line marks the onset of the swallowing behavior, which occurred after the onset of the GCaMP signal—specifically when the signal reached 15% of its peak.

Question 3: The authors either need to show the importance of this circuit for consumptive swallowing or re-frame the paper for a modulatory circuit for mechanosensory reflexive

swallows in anesthetized animals.

Response:

We have substantially revised the manuscript's framework and Discussion sections (Line 111-113, 641-643 and 767-774) to more precisely state that we identified a mechanosensory pathway that was essential for pharyngeal stimulation-evoked reflexive swallowing under anesthetized conditions.

2) The conclusion that the pathway is Piezo2 vagal fibers -TH+ NTS -RVLM (Fig. 8k). This conclusion is a model that stems from the data and not a conclusion that can be made. For example, characterizing that the TH+ NTS neurons that project to the RVLM in the swallowing behavior (Fig. 5 / 6) does not demonstrate that this projection is the motor pathway to induce a swallow. None of the experiments conducted are modulating the synaptic release exclusively in the RVLM, and, furthermore, the mylohyoid muscle used to measure swallowing has motor neurons within the pontine CN5. While the authors can conclude that the TH+ NTS neurons with the RVLM projections are specialized for the swallow behavior (compared to those that project to the PBC), they cannot conclude that this is the motor pathway.

We sincerely thank you for raising this critical point. We fully agree that the existing data do not suffice to define the "TH+ NTS-RVLM" connection as the direct motor output pathway that triggers swallowing, and our original description was indeed overly definitive and exaggerated.

Response:

(1) Previous research has established the general neural circuit involved in swallowing: peripheral sensory information is first relayed to the Nucleus Tractus Solitarius – Dorsal Swallowing Group (NTS-DSG). It is then transmitted to the Ventrolateral Medulla – Ventral Swallowing Group (VLM-VSG). Subsequently, neural signals are conveyed via the nucleus ambiguus (swallowing motoneurons) and pontine swallowing neurons, ultimately activating

bilateral cranial nerves V, VII, IX, X, XII, and the C1-3 spinal motor centers to precisely coordinate the timing and rhythm of swallowing movements (references: *Brain stem control of swallowing: neuronal network and cellular mechanisms*; *The Cortical and Subcortical Neural Control of Swallowing: A narrative review*; *Neurophysiology of swallowing*).

The innovative findings of our study are: (1) identifying that the mechanoreceptors for peripheral sensory mechanical stimulation are Piezo2 ion channels in primary sensory neurons of the vagus ganglion; and (2) demonstrating that the subpopulation of TH+ NTS neurons projecting to the RVLM constitutes a key neuronal type for the swallowing reflex. Therefore, the primary objective of this study is to reveal the specific relay pathway within the brainstem for pharyngeal mechanosensory information (i.e., TH+ NTS neurons and their specific projection to the RVLM) and to prove its necessity for reflexive swallowing, rather than to delineate the entire sensorimotor pathway from sensation to muscle activation.

In response to this conclusion, we have rephrased the text and made revisions throughout the manuscript (in the Abstract, Results, Discussion, and the description of the schematic in Fig. 8n). We have removed the potentially misleading term "motor pathway" and instead focus on emphasizing the conclusion that "TH+ NTS neurons projecting to the RVLM are involved in swallowing".

(2) Our previous data also confirmed the existence of a monosynaptic connection between TH+ NTS neurons and the RVLM (Supplementary Fig. 10f). We have further employed optogenetic inhibition approaches in mice's swallowing experiments. To this end, AAV-DIO-hGtACR1-mCherry (test) or AAV-DIO-mCherry (ctrl) was injected into the NTS of *TH*-ires-cre mice, and optical fibers were implanted above the bilateral Amb/RVLM. A 3-day adaptive drinking training was conducted before the formal test. All animals first drank water for 30 minutes under non-light stimulation conditions. On the next day, continuous 473 nm blue light was used to inhibit the TH+ NTS-Amb/RVLM pathway. Optogenetic inhibition test demonstrated that inhibiting the terminals of TH+ NTS neurons within the RVLM nucleus suppressed swallowing behavior (Response Document Figure 2, and also see new Figure 5o-q). This result provided additional evidence that the TH+ NTS-RVLM projection was involved in swallowing.

We have updated these results with our new data in the revised manuscript (Line 411-419) and Methods (Line 189-198).

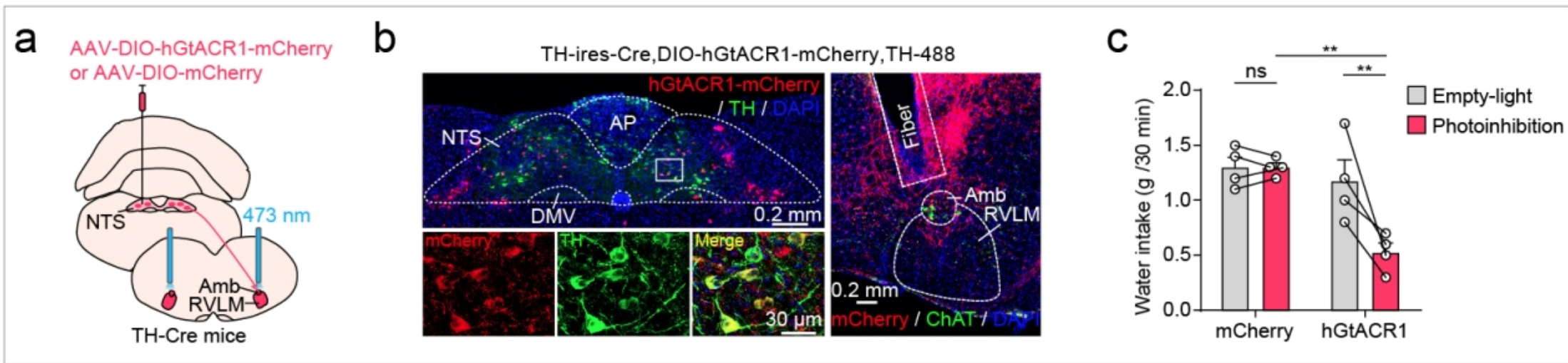

Response Document Figure 2 | Optogenetic inhibition of the TH+ NTS-Amb/RVLM pathway reduced water intake in mice. **a** Schematic diagram showing injection sites of AAV-DIO-hGtACR1-mCherry (test) or AAV-DIO-mCherry (ctrl) in the NTS and optical fiber implantation in the Amb/RVLM of *TH*-ires-Cre mice. **b** Example micrographs showing hGtACR1-mCherry expression in the NTS and the optical fiber track above the axon terminals in the Amb/RVLM, as well as the colocalization of hGtACR1-mCherry+ (red) with TH+ neurons (green). **c** Water intake of mice after optogenetic inhibition of TH+ NTS-Amb/RVLM projections (n=4 mice).

(3) We supplemented experimental verification of the regulation of synaptic release within the RVLM. TH is the rate-limiting enzyme in the synthesis of dopamine (DA), which is further converted into norepinephrine (NE). To monitor the release of DA or NE in the Amb/RVLM, a genetically encoded GPCR-activated GRAB-DA sensor or GRAB-NE sensor was employed to track DA/NE levels. Specifically, AAV-DIO-C1V1-mCherry (test) or AAV-DIO-mCherry (ctrl) was injected into the NTS of *TH*-ires-cre mice and an optical fiber was embedded above it for optogenetic activation. Meanwhile, AAV-GRAB-DA2h or AAV-GRAB-NE2m was injected into the Amb/RVLM, with an optical fiber embedded above it to monitor neurotransmitter signals. 2-3 weeks after viral expression, the NTS was optically activated while synaptic release within the Amb/RVLM was recorded. The results showed that optogenetic activation of the NTS (589 nm, 20 Hz, 10 ms, 2 mW, 2 s) triggered a significant increase in dopamine signals within the Amb/RVLM, rather than norepinephrine. In the control group, no obvious fluorescent changes were observed in Amb/RVLM neurons upon optogenetic stimulation of the NTS. Furthermore, treatment with the DA antagonist

haloperidol (Haldol) eliminated light-induced GRAB-DA signals (Response Document Figure 3, and also see new Supplementary Figure 12). These findings confirmed that activating the TH+ NTS neurons triggered the release of DA in the Amb/RVLM.

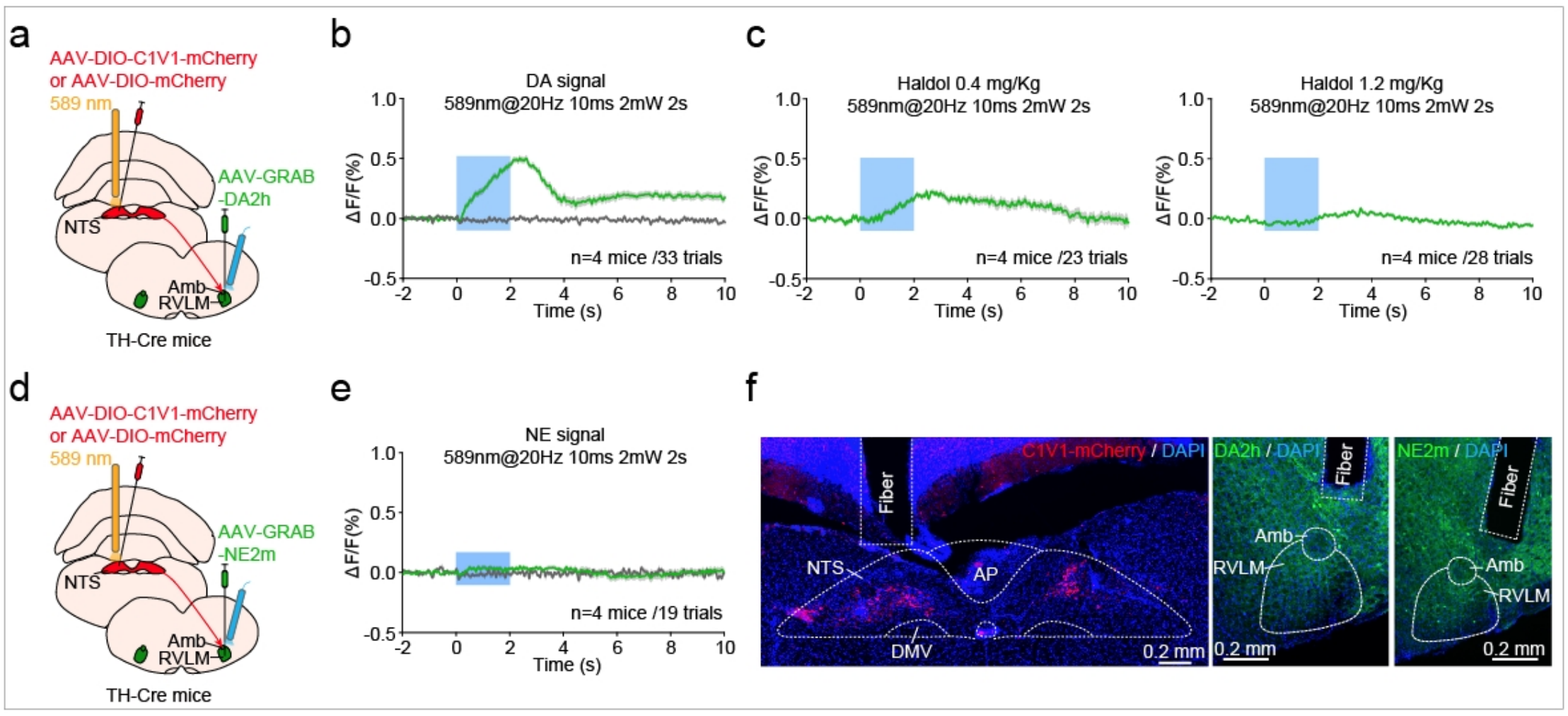

Response Document Figure 3 | Light-activated NTS regulated the release of dopamine in the Amb/RVLM. a Schematic diagram showing injection sites of AAV-DIO-C1V1-mCherry (test) or AAV-DIO-mCherry (ctrl) into the NTS and AAV-GRAB-DA2h into the Amb/RVLM of *TH*-ires-cre mice, followed by optical fiber implantation above the NTS and Amb/RVLM to apply optogenetic activation and fiber photometry, respectively. **b** Dopamine signal (ΔF/F) released from Amb/RVLM in 33 trials of optogenetic activation of NTS. **c** DA signals (ΔF/F) released in the Amb/RVLM during light activation of NTS in 23-28 trials after treatment with the dopamine antagonist Haldol. **d** Schematic diagram showing optogenetic activation of NTS and recording of NE signals in the Amb/RVLM. **e** Norepinephrine signals (ΔF/F) released from Amb/RVLM in 19 trials of optogenetic activation of NTS. **f** Example micrographs showing C1V1-mCherry expression and optical fiber track in the NTS (left). And GRAB-DA sensor or GRAB-NE sensor expression and optical fiber track in the Amb/RVLM (middle and right).

We have updated these results with our new data in the revised manuscript (Line 464-487) and Methods section (Line 267-282).

Once again, thank you for helping us enhance the rigor of our study and the precision of our presentation.

3) The authors fail to cite and integrate their findings with existing literature – The authors are studying TH+ NTS neurons which have recently been described to be involved in esophageal pressure sensing and motility (Wang, 2024) and assays that are used to confirm swallowing (the twine assay) could be a consequence of inducing esophageal motility. Also, activation of the SLN in Piezo2-Cre; Ai32 mice has been done in Prescott, 2020 and it did not evoke swallowing (contrast to Fig. 7). TH+ neurons are also Dbh+ (one example in Wang, 2024), so why does Dbh-Cre not label these swallowing neurons and why does the sequencing data provided not show that TH+ neurons are not Dbh+ (Supplemental Fig. 3). It is important for the authors to properly integrate their study with others.

Thank you for this critical points regarding the integration of our findings with the existing literature. These comments are essential for placing our study in the proper context and for clarifying the specificity of our conclusions. We address each point below.

Question 1: The authors fail to cite and integrate their findings with existing literature – The authors are studying TH+ NTS neurons which have recently been described to be involved in esophageal pressure sensing and motility (Wang, 2024) and assays that are used to confirm swallowing (the twine assay) could be a consequence of inducing esophageal motility.

Response:

(1) We appreciate your important point. We fully agree that contextualizing our findings within a broader literature is essential for accurately interpreting their significance. Accordingly, we have revised the manuscript (Introduction and Discussion sections, Line 70-81 and 755-797) to address this.

(2) The swallowing process is divided into three phases, including the oral phase, pharyngeal phase, and esophageal phase. The scientific hypothesis we propose is that TH+ NTS neurons mediate the pharyngeal swallowing reflex triggered by mechanical stimulation of the pharynx. Our further experimental data was consistent with and does not conflict with the observation by Wang, 2024 that TH+ NTS neurons were involved in esophageal motility. In fact, Wang, 2024 did not mention esophageal motility, instead, it placed greater emphasis on esophageal

mechanical distension. It is precisely during the oral phase of swallowing that, after mastication, food boluses are propelled into the pharynx by the tongue and other structures, leading to esophageal mechanical distension. This distension is perceived by TH+ NTS neurons, which then regulate esophageal motility and trigger the swallowing reflex. Inactivation of these neurons impairs the perception of mechanical stimulation by food boluses entering the pharynx, even if it does not affect mastication and propulsion of oral-phase food into the pharynx. Although the bolus entering the pharynx produces mechanical stimulation to the pharynx, the pharyngeal phase of swallowing cannot proceed, leading to dysphagia. Furthermore, the intensity of Von Frey stimulation of the pharynx simulated the size of food boluses, as evidenced by the TH+ NTS neurons dynamics recorded (Fig. 4g and Supplementary Fig. 7a), which was consistent with the conclusion of Wang, 2024. The drinking experiment also confirmed that TH+ NTS neurons were unrelated to drinking motivation, which was consistent with the phenomenon observed by Wang, 2024.

(3) The related twine experiments were conducted by combining video recording and mylohyoid electromyography (EMG) to determine swallowing behavior; the rope swallowing length was used to reflect the physiological function of swallowing.

Question 2: Activation of the SLN in Piezo2-Cre;Ai32 mice has been done in Prescott, 2020 and it did not evoke swallowing (contrast to Fig. 7).

Response:

Regarding the discrepancies between the result of the present study and those of Prescott, 2020: factors including the genetic background of the mice used, experimental parameters (light stimulation intensity, duration), as well as anesthesia protocols and depth of anesthesia, can all affect the induction of swallowing behavior.

A detailed analysis of the differences between the two studies is as follows:

(1) The experimental animals used were not entirely identical. Prescott *et al*. utilized *Piezo2-ires-Cre;loxP-ChR2* mice, whereas our study adopted *Piezo2-Cre;Ai32* mice.

Although both *Piezo2-Cre;loxP-ChR2* and *Piezo2-Cre;Ai32* mice are functionally similar in that Cre-mediated recombination drives ChR2 expression specifically in Piezo2-expressing cells, they differ in their specific genetic constructs, ChR2 expression levels, promoter regulation, and/or other genetic background details.

Specifically, *Piezo2-Cre;loxP-ChR2* mice are generated by flanking the ChR2 gene with loxP sites via genetic engineering. Cre recombinase recognizes these loxP sites, inserting the ChR2 gene into the genome of Piezo2-expressing cells through a LoxP-STOP-LoxP cassette. In contrast, the *Ai32* mouse is a specific, standardized transgenic strain harboring a loxP-ChR2 construct, generated by The Jackson Laboratory (JAX Stock No. 012569). It is specifically designed for conditional ChR2 expression. Crossing *Ai32* mice with *Piezo2-Cre* mice enables optogenetic control in specific cell types.

The *loxP-ChR2* mice category may encompass various lines with differences in details such as the insertion site, ChR2 variant, or fluorescent tag. Their key components typically include a LoxP-STOP-LoxP cassette, the ChR2 (H134R) variant, and a reporter gene (e.g., EYFP). The *Ai32* strain, however, is highly standardized with a well-defined genetic background (*C57BL/6J*). Its key features include: precise insertion of the loxP-ChR2 construct into the Rosa26 locus, a strong CAG promoter driving high-level ChR2 expression post-Cre activation, a LoxP-STOP-LoxP cassette, and a ChR2 (H134R)-EYFP fusion protein. JAX provides comprehensive genotyping protocols, usage guidelines, and published data, ensuring high reliability.

In summary, the *Piezo2-Cre;Ai32* strain may be optimized for ChR2 expression efficiency, stability, or compatibility with other genetic modifications. Therefore, these should not be considered identical mouse strains.

(2) The optical activation parameters were not identical. Prescott *et al*. used the following parameters for SLN activation: 473 nm, 20 Hz, 5 ms pulses. We employed stimulation at higher frequencies and with different pulse widths (473 nm, 20, 40, 50 Hz, 10 ms pulses). Additionally, illumination of the SLN in *Piezo2-Cre/Ai32* mice with 473 nm, 20 Hz, 5 ms pulses failed to successfully induce swallowing behavior.

In summary, the differences between the results of this study and those of Prescott, 2020 may be attributed to factors such as the genetic background of the mice used, experimental parameters (light stimulus intensity and duration), as well as anesthetic protocols and anesthetic depth. An analysis of these discrepancies has been incorporated into the Discussion section (Line 715-754) of the present study.

Question 3: TH+ neurons are also Dbh+ (one example in Wang, 2024), so why does Dbh-Cre not label these swallowing neurons and why does the sequencing data provided not show that TH+ neurons are not Dbh+ (Supplemental Fig. 3). It is important for the authors to properly integrate their study with others.

Response:

Dbh uses dopamine synthesized by TH as its substrate, thus theoretically Dbh+ neurons are TH+. Wang, 2024 core research focused on TH+ neurons in the caudal nucleus tractus solitarii (cNTS). The paper does not appear to explicitly declaring or prove that these Dbh+ neurons are TH+.

(1) Tyrosine hydroxylase (TH) is the rate-limiting enzyme for the first step of all catecholamine synthesis, whereas dopamine β-hydroxylase (Dbh) is the enzyme that converts dopamine to norepinephrine. Therefore, there exists a classic subset of dopaminergic neurons that express TH but not Dbh (or express Dbh at extremely low levels). To this end, we co-stained TH and Dbh antibodies in the NTS of *WT* mice. The results showed that there is indeed a class of TH+/Dbh- neurons, which may specifically mediate the swallowing reflex. In addition, Dbh+ neurons accounted for approximately 18.94% of TH+ neurons (Response Document Figure 4), and may be insufficient to induce the swallowing reflex due to their smaller number. Our co-immunohistochemical staining of TH and Dbh antibodies is consistent with the results observed by multiple in situ hybridization (mRNA FISH) technology in the literature (Fig. 5), clearly demonstrating the co-localization of TH and Dbh transcripts within the same cell (references: *Reciprocal activity of AgRP and POMC neurons governs coordinated control of feeding and metabolism*).

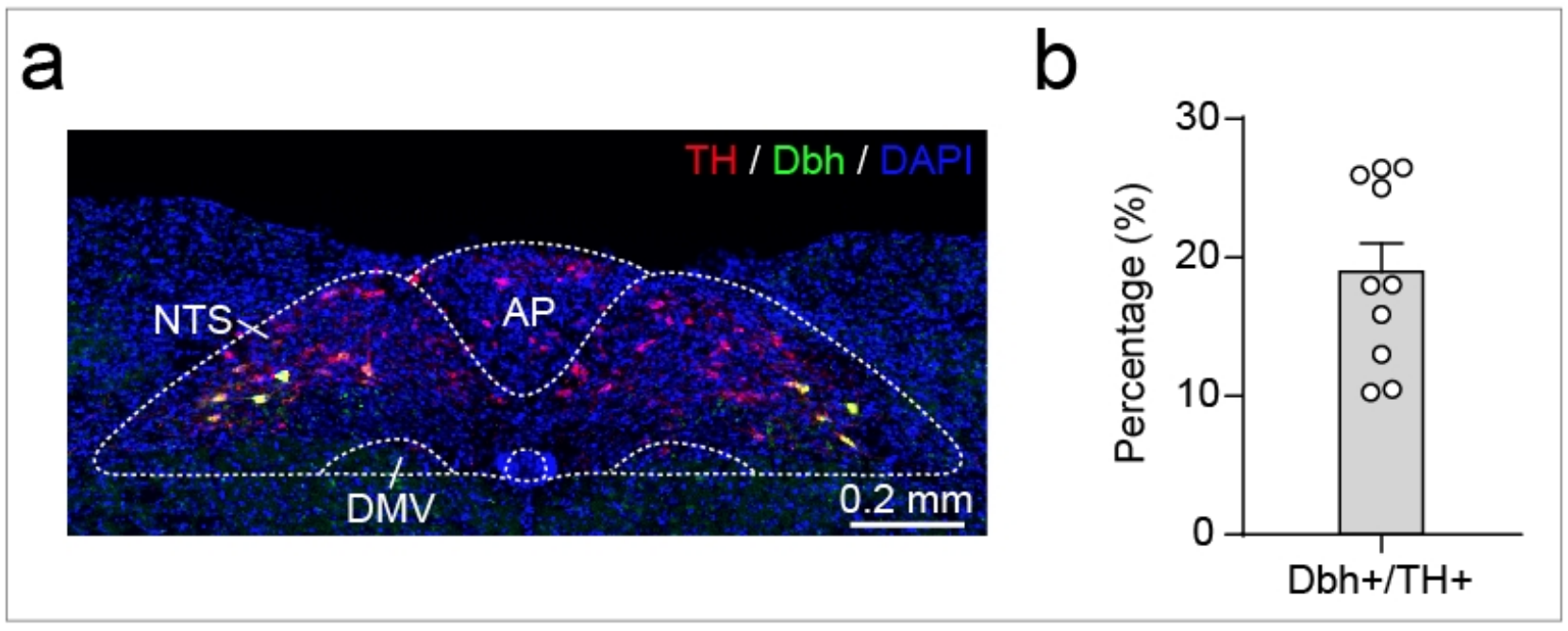

Response Document Figure 4 | Distribution of TH+ and Dbh+ neurons in the NTS. a Representative images of TH and Dbh neuron distribution in the NTS. **b** The ratio of Dbh+ neurons to TH+ neurons (n=4 mice).

(2) The supplementary results of regulation of synaptic release within the RVLM (Response Document Figure 3, and also see new Supplementary Figure 12) showed that optogenetic activation of the NTS triggered a significant increase in dopamine signals within the Amb/RVLM, rather than norepinephrine. Furthermore, treatment with the DA antagonist haloperidol (Haldol) could eliminate light-induced GRAB-DA signals. These findings confirmed that activating the TH+ NTS neurons triggered the release of DA in the Amb/RVLM.

(3) In addition, related research reports indicate that Dbh+ neurons are more closely associated with airway hyperresponsiveness. (references: *Brainstem Dbh+ neurons control allergen-induced airway hyperreactivity*).

Thus, Dbh+ neurons cannot mark swallowing, which may be due to two scenarios: (1) The limited number of Dbh+ neurons is insufficient to trigger swallowing; (2) There exists a molecularly unique subgroup of TH+ / Dbh-low/negative NTS neurons that specifically mediate the swallowing reflex. This provides a novel perspective for the fine-grained analysis of functional subtypes in the NTS.

(4) Sequencing data for TH and Dbh:

1) Heterogeneity of NTS: The NTS is a complex nucleus. Our sequencing data analysis covers the global data of the entire NTS (including the rostral, middle, and caudal subregions), whereas Wang, 2024 focused on the caudal nucleus of the NTS (cNTS) region. The classical

catecholamineergic cell population is primarily located in the middle and caudal parts of the NTS.

2) We reanalyzed the sequencing data of TH and Dbh. Dot plots showed that TH+ neurons also express Dbh, with good colocalization between the two. Due to dropout defects in sequencing, the expression of TH and Dbh may be inconsistent in a few cells, but their distributions are consistent in UMAP analysis (Response Document Figure 5).

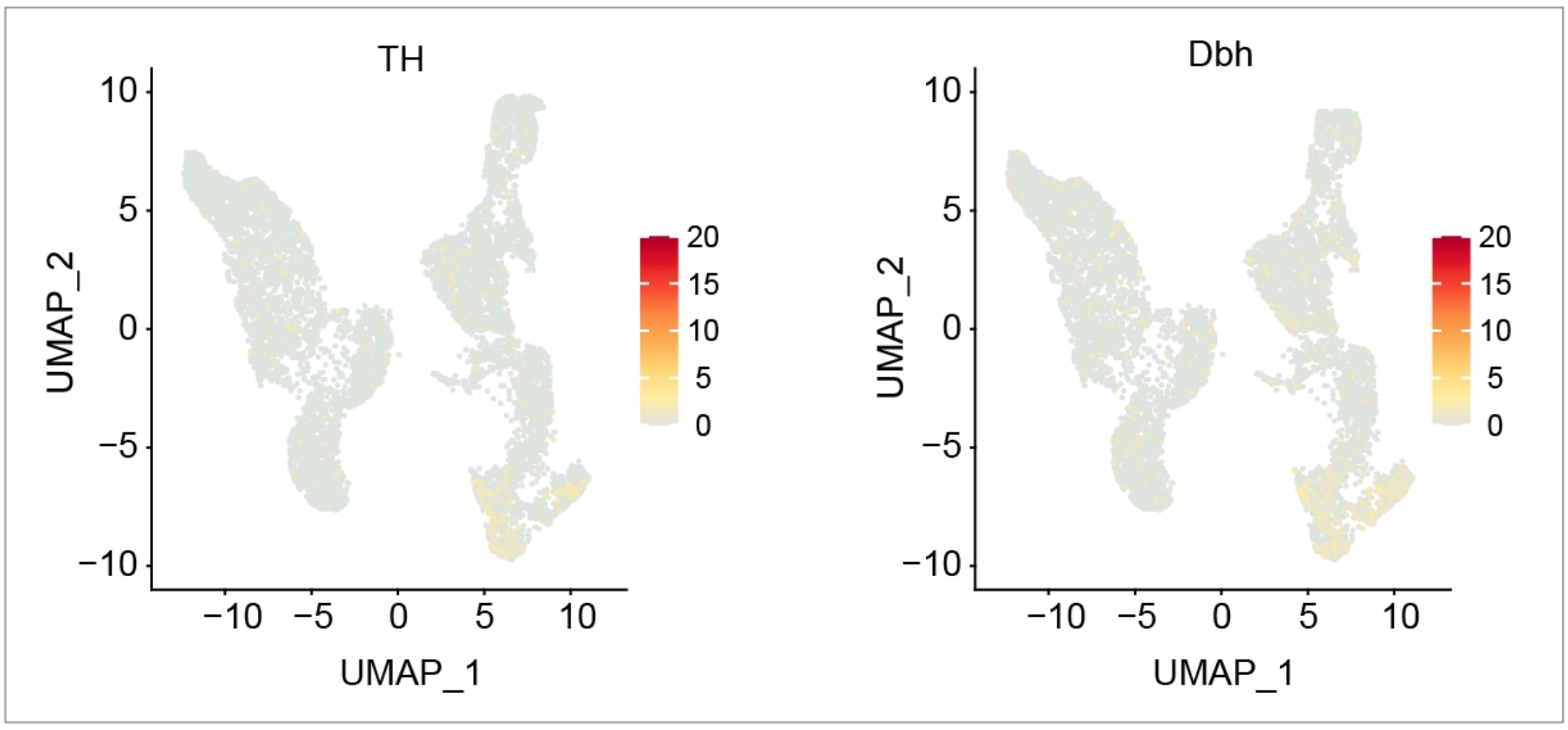

Response Document Figure 5 | UMAP plot of TH and Dbh gene expression across individual cells.

In general, in this study, we utilized Dbh-Cre line to fail in labeling NTS neurons involved in swallowing regulation. This result is highly consistent with the latest findings by Wang, 2024 in Nature Neuroscience. Their study, through meticulous functional screening and molecular identification, demonstrated that TH+ cNTS neurons, which encode mechanical expansion perception of the esophagus, function independently of the classical catecholaminergic (Dbh+) system. Our findings further support the model of parallel pathways with distinct functional and molecular identities in the NTS. Future research should integrate multiple in situ hybridization techniques to directly identify the complete neurochemical phenotypes of swallowing-related NTS neurons and elucidate their spatial distribution and functional interactions with catecholaminergic cell populations.

4) Overall, there is a poor description of the primary data and the methodology. This makes it challenging to understand what the manipulations actually are / look like. Examples of this and they are noted below.

Thank you for your exceptionally thorough examination and valuable suggestions regarding our primary data and methodology. We fully acknowledge that these details are crucial for reproducibility and interpretation, and we will provide comprehensive clarifications and additional data to address each point raised.

Question 1: Von Frey experiments - Figure 1b shows the external throat movements that were monitored to quantify a swallow, however the methods state that the pharynx had been surgically opened from the ventral side, EMG electrodes implanted in the mylohyoid muscle and VF filaments applied to the pharyngeal mucosa. These two things are incompatible. If external movements were used as a measure of swallow for some experiments and EMG-pharyngeal pressure for others, then this needs to be clearly stated for each experiment. Additionally, a similar photo series showing the placement of the VF filaments in the pharynx, the pressure transducer etc would be very informative to the reader on how the experiment was performed. Given that these experiments are central to much of the paper, this clarification is essential. The stimulation site could be further explained by referencing anatomical landmarks in the pharynx e.g. proximity to epiglottis, hard palate.

Response:

(1) We apologize for the ambiguity in describing the experimental methodology. We hereby provide a detailed clarification regarding the recording protocols for physiological indicators in different experimental groups as follows: For swallowing behavior induced by oral water administration in awake mice (Supplementary Fig. 7b, and Fig. 6k) and swallowing behavior induced by laryngeal chemical stimulation (Fig. 3m, Fig. 6h, Fig. 6l, Supplementary Fig. 6c, Supplementary Fig. 7c), and the swallowing twine experiment (Fig. 2j, Fig. 3c right, Fig. 5f and Fig. 5n), the measurement metrics were mylohyoid muscle electromyography (EMG) recordings combined with video-monitored laryngeal elevation. For all other experiments, swallowing behavior was assessed using a combined metric system consisting of mylohyoid

muscle EMG recordings, pharyngeal pressure measurements, and video-monitored laryngeal elevation.

To address this issue, we have made detailed revisions and supplements to the Methods section (Line 59-67), clearly specifying the exact measurement metrics corresponding to different experimental scenarios, thus ensuring the clarity and reproducibility of the experimental methodology.

(2) Following your suggestions, we have added supplementary photographs depicting the experimental procedures. These images clearly illustrated the surgical incision site in the pharyngolaryngeal region, the stimulation site of Von Frey filaments in this region (proximal to the epiglottis), as well as the precise placement positions of the pressure-sensitive latex balloons on the base of the tongue and hard palate (Response Document Figure 6, and also see new Supplementary Figure 1a), thereby clarifying the key details of the experimental operations.

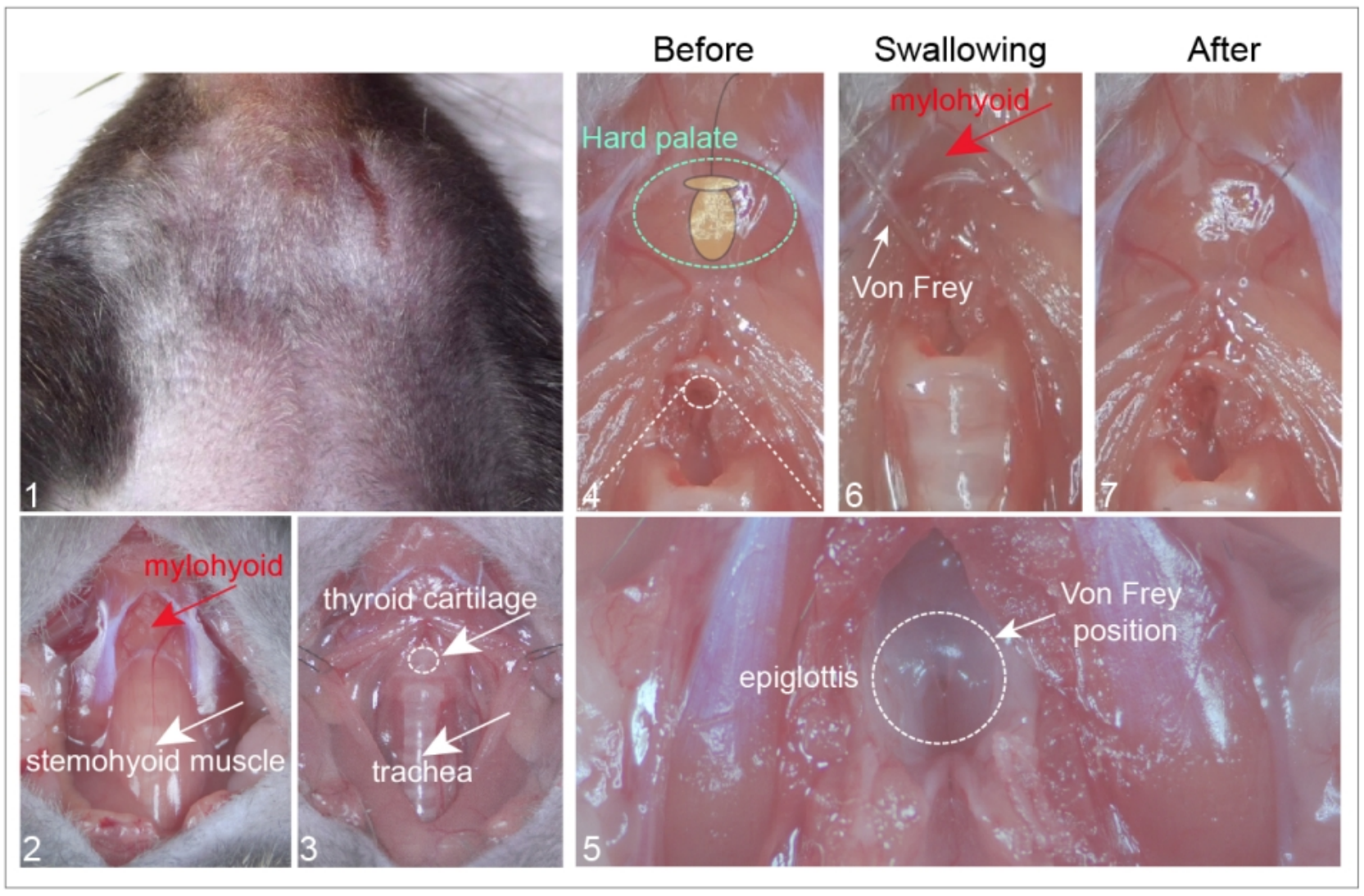

Response Document Figure 6 | Surgical procedure for inducing swallowing in mice through mechanical stimulation of the pharynx. 1 Anesthetized mice were placed in a supine position. **2** Incise the skin of the neck to expose the mylohyoid muscle and sternohyoid muscle. **3** Make a longitudinal incision in the sternohyoid muscle and fix it to both sides with surgical sutures. **4** Incise the thyroid cartilage to expose the pharynx, and place the sensing latex balloon orally at the base of the tongue and the hard palate. **5** The stimulation site of the

Von Frey filaments. **6** Stimulation of the pharynx with Von Frey filaments induced contraction of the mylohyoid muscle and elevation of the larynx during swallowing. **7** The swallowing reflex was completed.

Question 2: Were any numerical or analytical thresholds used to determine swallowing? From the representative traces presented (Figure 1C) it is clear where the events are but since the swallowing magnitude varies with stimulus weight (Figure 1G), it is important to know how a swallow was defined, particularly at lower stimulus weights. Additionally, transient apnea is mentioned as a defining feature of a swallow but it is unclear from the main text and methods how breathing was being recorded in this experiment. It is also unclear how 'tracheal peristalsis' is related to swallowing (or what it is), how it was quantified and where this quantification is in the paper.

Response:

(1) We defined swallowing events by integrating three complementary assessments: video-monitored laryngeal elevation, mylohyoid muscle electromyography (EMG) recordings, and pharyngeal pressure measurements. Specifically, a pharyngeal pressure amplitude exceeding 10 mmHg was set as the quantitative threshold for identifying a valid swallowing.

(2) Respiratory activity in mice was recorded using a tension transducer, with the detailed procedure as follows: Anesthetized mice were placed in a supine position. Respiration was monitored using a JZ100 force transducer (Beijing Xinhang Xingye Science and Technology Trade Co., Ltd., China). Specifically, a suture was passed through the skin of the thoracic wall and attached to the elastic cantilever of the transducer, which was gently tightened to capture chest wall movements. Thoracic expansion and contraction during respiration deformed the cantilever, and the transducer converted this mechanical signal into an electrical signal, which was then recorded by a data acquisition (DAQ) system. During optogenetic experiments, the DAQ was synchronized with the output of the optical stimulator to ensure precise timing between stimulation and recording. Signals were digitized at a sampling rate of 100 Hz and acquired using Fiber Photometry software. For analysis, we manually counted the total

respiratory wave peaks within three consecutive 5-s epochs: the 5 s immediately after the onset of light stimulation, and the 5 s periods immediately before and after the stimulation. It should be noted that the data presented in the figures represent breaths per second. Relevant methodological details have been supplemented in the Methods section (Line 69-83) of the revised manuscript.

(3) We sincerely apologize for any confusion caused by the inappropriate terminology in the original manuscript. The term "tracheal peristalsis" previously used actually refers to laryngeal elevation, and this erroneous expression has been corrected at the corresponding position in the revised manuscript (Line 132).

Question 3: When conducting the Von Frey experiments, how many times is the pharynx probed at each pressure step and are there responses at each step. For example, in Fig. 3K, it is reported that 1-1.5 swallows occur per 30 probe trials. Does this mean that only 1 out of every 30 trials has a swallow? Or one swallow occurs each trial? The overall reproducibility of the behavior within a single animal is not shown and instead the panels in each figure are displayed as a mean of the mean for each animal.

Response:

(1) During the Von Frey experiments, 3 stimulations were delivered to the pharynx of each mouse at every stimulus intensity, with each stimulation lasting 2 seconds. The mean value of the swallowing counts induced by these 3 stimulations was defined as the swallowing response level of the mouse at the corresponding stimulus intensity. Specifically, within the low-threshold stimulus intensity range (0.008 g - 0.16 g), not every stimulation could successfully induce a swallowing response, with either 0 or 1 swallowing triggered per mechanical stimulation. When the stimulus intensity reached 0.4 g and above, each stimulation could elicit 1 or 2 swallowing. In addition, we have supplemented the manuscript with statistical graphs illustrating the number of swallowing induced by different intensities for individual mice (Response Document Figure 7, and also see new Figure 1e-g). Detailed descriptions were added to the revised manuscript manuscript (Lines 124-127).

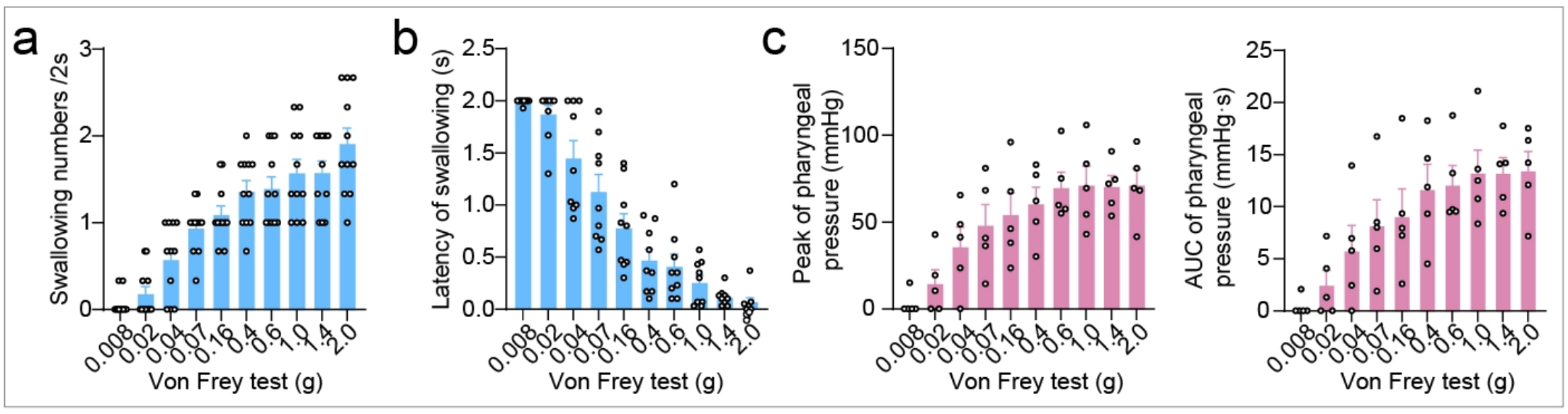

Response Document Figure 7 | Mechanical stimulation of the pharynx induced swallowing in mice. a-b Number (**a**) and latency (**b**) of swallowing induced by mechanical stimulation of varying intensities in individual mice. **c** The amplitude and area under the curve (AUC) of pharyngeal pressure during swallowing induced by mechanical stimulation of varying intensities in individual mice.

(2) The incorrect ordinate labeling of Fig. 3k caused confusion among you. As correctly noted by you, one swallowing was induced per stimulation in this experiment. We have now revised the ordinate labeling of this figure accordingly (Response Document Figure 8, and also see new Figure 3k).

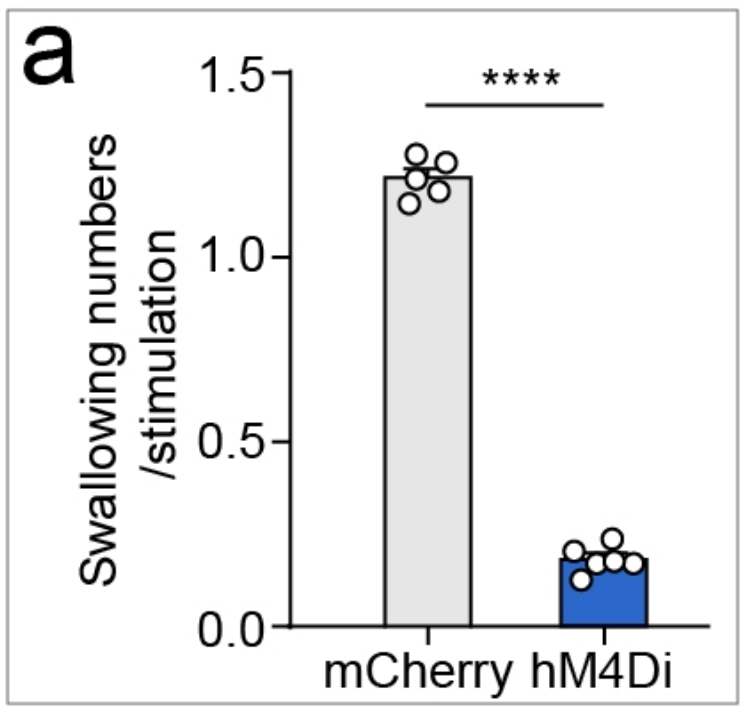

Response Document Figure 8 | Chemical inhibition of TH+ NTS neurons reduced swallowing elicited by mechanical stimulation. a Swallowing numbers induced by per 0.4 g Von Frey stimulation in *TH*-ires-Cre mice following chemogenetic inhibition of TH+ NTS neurons (mCherry: n=5 mice, hM4Di: n=6 mice).

Question 4: The parameters of stimulation to evoke c-FOS are not adequately described in the text or methods. What was the duration of the stimulation? Was it continuous or intermittent? These are described for the FosTRAP experiment and so I presume they are the same but this

needs to be stated. The “arytenoid cartilage” exposes the larynx and not the pharynx (line 138).

Response:

(1) The stimulation parameters for c-Fos activation induced by pharyngeal mechanical stimulation-evoked swallowing in mice were mentioned in the legend of Fig. 1i, but the description was insufficiently detailed. In response to your suggestion, we provide a detailed clarification here: To eliminate the interference of environmental factors on c-Fos activation, the entire experiment was conducted in a quiet environment devoid of strong light. Mechanical stimulation was applied to the pharynx of anesthetized *WT* mice using 0.4 g Von Frey filaments, with a total stimulation duration of 60 minutes. The stimulation paradigm consisted of a 2-second stimulation delivered every 1 minute. For control mice, only the pharynx was exposed without any mechanical stimulation. 90 minutes after the end of stimulation, the mice were perfused and their brains were harvested. Subsequently, coronal sections of the whole brain were prepared and c-Fos immunofluorescence staining was performed to localize the neural nuclei associated with swallowing behavior. The expression level of c-Fos in the NTS was quantified via manual counting by an experimenter who was blinded to the experimental grouping. Relevant operational details have been supplemented in the Methods section (Line 85-96) of the manuscript accordingly.

(2) In addition, we have deleted the incorrect description of “arytenoid cartilage” in Line 138 of the original manuscript.

Question 5: The nucleus ambiguus is not positively identified with a marker like ChAT.

Response:

Choline acetyltransferase (ChAT) is a key enzyme that catalyzes the synthesis of acetylcholine, and is widely recognized as a specific molecular marker for cholinergic neurons. The nucleus ambiguus (Amb) is a motor nucleus responsible for regulating critical functions such as swallowing and vocalization, and is enriched with a large number of

cholinergic neurons. Therefore, ChAT-based immunostaining is a classic and reliable approach for the precise identification of the Amb in neuroanatomical localization. (references: *Ultrastructural localization of choline acetyltransferase in the rat rostral ventrolateral medulla: evidence for major synaptic relations with non-catecholaminergic neurons; Innervation of the rat trachea by bilateral cholinergic projections from the nucleus ambiguus and direct motor fibers from the cervical spinal cord: a retrograde and anterograde tracer study; Dense TRPV2 immunoreactivity defines a subset of motoneurons in the dorsal lateral nucleus of the spinal cord, the nucleus ambiguus and the trigeminal motor nucleus in rat; Expression of glial cell-derived neurotrophic factor receptors within nucleus Ambiguus during rat development; Anatomical evidence of non-parasympathetic cardiac nitrergic nerve fibres in rat*).

We attach great importance to the suggestion regarding the lack of using ChAT—a specific molecular marker—to precisely localize and verify that the brain region under study or stimulation is the nucleus ambiguus. In this regard, we provide the following clarification: In Fig. 5b and 5d of the original manuscript, the nucleus ambiguus was specifically labeled via ChAT immunostaining. However, in Fig. S8b, S9d, S13f and S14a, the nucleus ambiguus (Amb) was identified solely based on anatomical coordinates, without providing ChAT labeling for co-localization verification.

To address this limitation and ensure the accuracy of localization, we have performed supplementary experiments, in which ChAT immunofluorescence staining was conducted on the relevant samples (Response Document Figure 9, and also see new Supplementary Figure 8b, 9d, 13f and 14a). These experiments further verified that the target brain region is indeed the ChAT-positive nucleus ambiguus area.

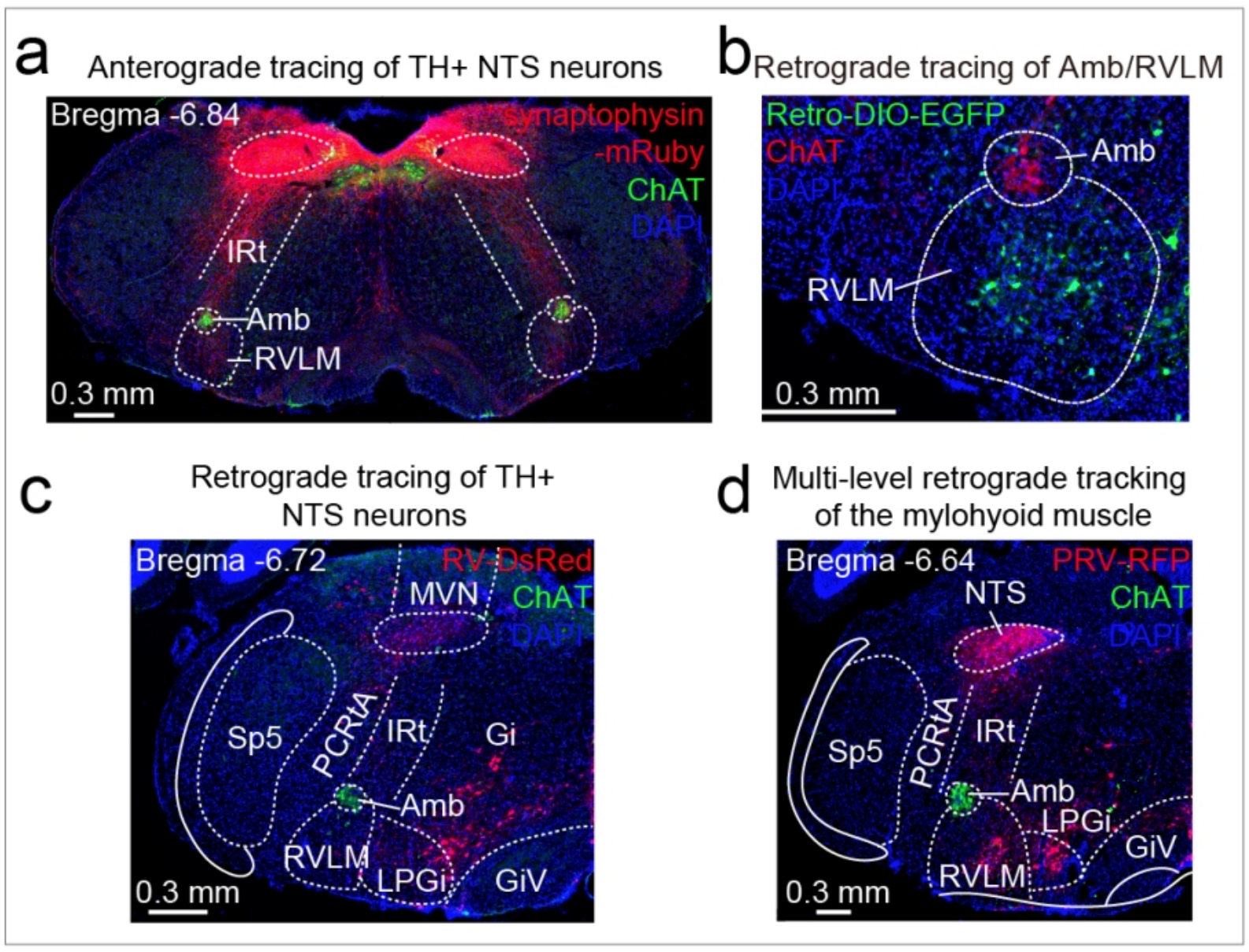

Response Document Figure 9 | Mark the nucleus ambiguus with ChAT neurons. a Anterograde tracing of TH+ NTS neurons. **b** Retrograde tracing of Amb/RVLM. **c** Retrograde tracing of TH+ NTS neurons. **d** Retrograde tracing of the mylohyoid muscle.

Question 6: No control c-FOS expression data is shown in the supplemental figure and the proper TRAP control should be TRAP-ChR2 animals with implants that are not mechanically probed. The mCherry control is only accounting for light induced effects but not the specificity of Fos expression.

Response:

Thank you for your valuable suggestions. We agree that the previous control group design was insufficiently rigorous and have now supplemented the study with an additional control group of TRAP-ChR2 mice that underwent fiber implantation but did not receive mechanical stimulation, as recommended.

The revised experimental protocol is as follows: On day 1, AAV-DIO-ChR2-mCherry was injected into the NTS of *Fos-CreER* mice, and an optical fiber was implanted above the NTS. On day 5, mice in the experimental group received mechanical stimulation of the pharynx using 0.4 g Von Frey filaments and were intraperitoneally administered 4-hydroxytamoxifen, whereas mice in the control group only underwent pharyngeal exposure without mechanical

stimulation. Two to three weeks later, swallowing behavior were tested upon optical activation, followed by perfusion and brain collection for immunohistochemical staining of c-Fos and mCherry. The experimental results are shown in the following figure (Response Document Figure 10, and also see new Supplementary Figure 2e):

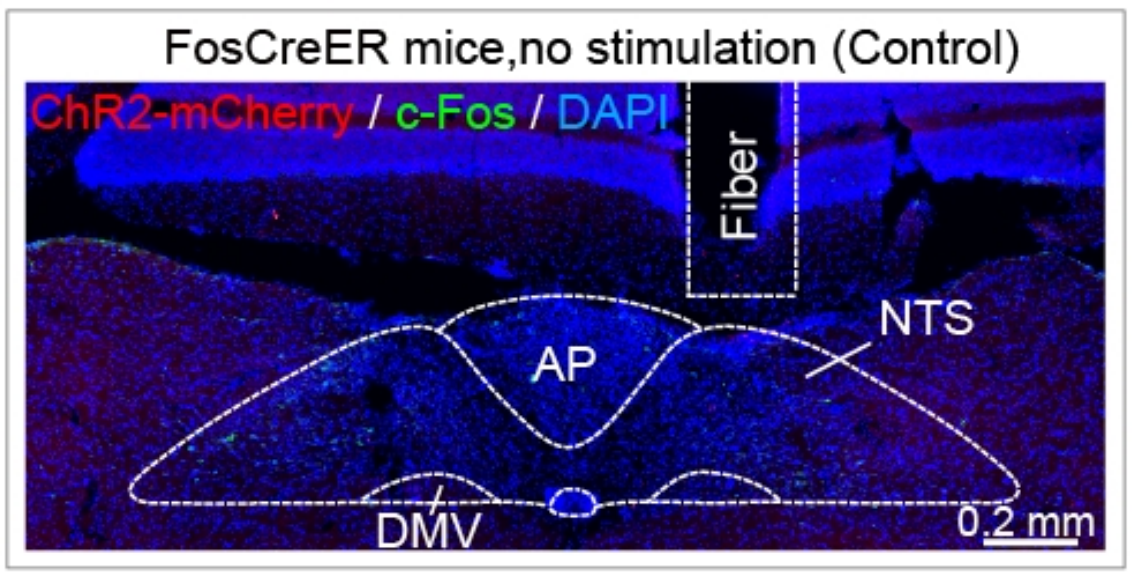

Response Document Figure 10 | Brain images immunostained for the control group mice (no mechanical stimulation) in the FosTRAP experiment.

The inclusion of this additional control group further supports our conclusions. The revised manuscript (Line 160-161) has been updated accordingly.

We sincerely appreciate your careful reading and constructive comments, which have significantly enhanced the rigor of the experimental design in this study.

Question 7: If the swallow is changing breathing, then it is hard to understand how the respiratory rate is not impacted by light in the optogenetic experiments throughout the paper. How breathing is annotated and quantified is not described.

Response:

(1) Regarding the respiratory pause during swallowing, it should be clarified that this is a brief physiological event lasting only 0.2-0.3 s. It occurs as a normal protective reflex—in which the epiglottis covers the trachea—when swallowing is elicited by optogenetic activation of TH+ NTS neurons. In our statistical analysis, respiratory frequency was evaluated over a 5 s window; such brief apneas contribute minimally to the total frequency, and thus do not significantly affect the statistical results of the overall respiratory rate. Moreover, a compensatory respiratory response typically follows the swallowing act. Overall,

there was no statistically significant difference in the mean respiratory frequency during the optical stimulation period compared to the periods before and after stimulation.

(2) Concerning the respiratory recording method, respiration in anesthetized mice was monitored using a JZ100 force transducer (Beijing Xinhang Xingye Science and Technology Trade Co., Ltd., China) with the animal placed in the supine position. Specifically, a suture was passed through the skin of the thoracic wall and attached to the elastic cantilever of the transducer, which was gently tightened to capture chest wall movements. Thoracic expansion and contraction during respiration deformed the cantilever, and the transducer converted this mechanical signal into an electrical signal, which was then recorded by a data acquisition (DAQ) system. During optogenetic experiments, the DAQ was synchronized with the output of the optical stimulator to ensure precise timing between stimulation and recording. Signals were digitized at a sampling rate of 100 Hz and acquired using Fiber Photometry software. For analysis, we manually counted the total respiratory wave peaks within three consecutive 5-s epochs: the 5 s immediately after the onset of light stimulation, and the 5 s periods immediately before and after the stimulation. This section has been added to the Methods section (Lines 69-83).

Question 8: In the experiment summarized in Figure 2a the authors demonstrate that optogenetic activation of NTS neurons in TH-cre but not lines for other transcriptomically distinct cell types. Figure Supp. 4 a shows the placement of fibers for this experiment which seems to vary across the medial-lateral axis. The authors should describe any exclusion criteria that were applied for misplaced fibers. Secondly, of the included mice, it would be informative for the reader to see a plot of fiber placements medial-lateral, rostral-caudal to see that fiber placement was equivalent between cre lines and these clusters can be correctly excluded as candidate swallow-evoking neuron populations. One reason why this is confusing is that Calb1 is expressed by all TH+ neurons (Fig. Supp. 4) yet it does not induce swallowing. And, Dbh has been reported to be expressed by TH+ neurons but does not induce swallowing. These results could simply be technical and not biological.

Response:

Thank you for raising these important questions and for close attention to the experimental details, which are crucial for improving our study. We fully understand your concerns regarding the accuracy of fiber placement and the specificity of the results across different neuronal subtypes. Below are our detailed responses to these two points:

(1) Exclusion criteria and validation of fiber placement

In the optogenetic experiments, we indeed established strict screening criteria for fiber implantation sites. All experimental animals underwent histological verification through brain sectioning after perfusion. Only mice whose fiber tips were located within the target NTS region were included in the final statistical analysis. To rule out potential result variations due to positional deviations of the fiber tips along the mediolateral (ML) or anteroposterior (AP) axes, we conducted optogenetic activation at different locations within the NTS across various Cre mouse lines. Following your suggestion, we have supplemented the revised manuscript with a distribution plot showing the positions of the fiber tips along the ML and AP axes for all included Cre mice (Response Document Figure 11, and also see new Supplementary Figure 4a-b). This plot demonstrates that the fiber implantation sites across different Cre lines were highly overlapping within the target NTS region, with no systematic bias. Therefore, the observed differences between lines are unlikely to stem from inconsistencies in fiber placement. We have added this result to the revised manuscript (Lines 195-198).

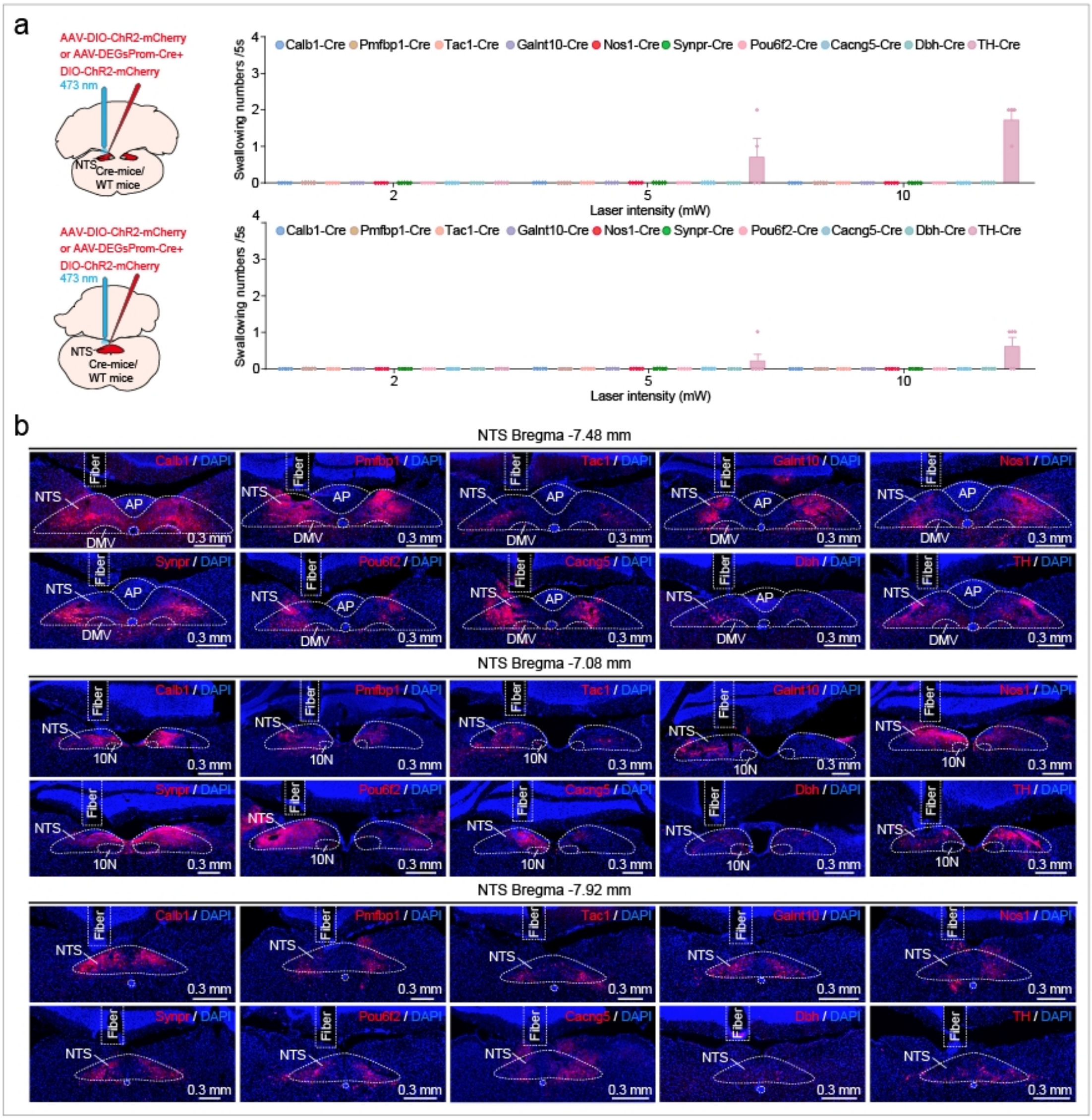

Response Document Figure 11 | Genetic screening for regulating swallowing. a Schematic illustrating ChR2-mCherry injection and fiber implantation sites in the rostral NTS (Bregma -7.08 mm) and caudal NTS (Bregma -7.92 mm) of Cre-line and *WT* mice; statistical graphs showing swallowing number evoked by light (473 nm, 10 ms, 20 Hz, 2, 5 and 10 mW) of medial-lateral or anterior-posterior axes of the NTS (n=4-5 mice). **b** Representative images of ChR2-mCherry expression and optic fiber placements of all AAV-DEGsProm-Cre virus or Cre-line mice.

(2) Functional differences among TH+, Calb1+, and Dbh+ neurons in swallowing elicitation.

As replied in Question 3 (Response Document page 13), the reasons for the functional differences between TH+ neurons and Dbh+ neurons in swallowing-induced processes may

include, 1) there may exist a class of TH+/Dbh- neurons that specifically mediate the swallowing reflex; 2) Dbh+ neurons constitute a smaller proportion of TH+ neurons and are insufficient to induce the swallowing reflex; and 3) Dbh neurons are more closely associated with airway hyperreactivity. Our study may indicat that a molecularly distinct subpopulation of TH+/Dbh-low/negative neurons in the NTS specifically mediates the swallowing reflex. This may provide a new perspective for finely dissecting functional subtypes within the NTS.

Additionally, the extended exposure time may have resulted in a more extensive mCherry distribution in the Calb1-Cre NTS sections of the original Supplementary Fig. 4. The statement "Calb1 is expressed by all TH+ neurons" was not described in our manuscript. Although studies have reported that 58.2% of TH neurons in the VTA region co-express Calb1 (reference: *Calbindin content and differential vulnerability of midbrain efferent dopaminergic neurons in macaques*), this phenomenon remains unclear in the NTS. Calb1+ neurons constitute a functionally highly heterogeneous population involved in diverse roles. Our previous research revealed that Calb1+ NTS neurons mediate retching-like behavior (reference: *Brain circuits for retching-like behavior*).

Question 9: In Figure 3f-i, water-restricted mice had drinking behavior quantified after chemogenetic inhibition of TH+ NTS neurons. CNO injected mice drank less water in 30 minutes while spending an equivalent amount of time at the spout than when injected with saline and non-DREADD expressing controls. It is not clear from the methods, figure legend or text how many of the parameters reported in Figure 3 were calculated. For the drinking experiment the methods merely state that behavior was observed from a camera. What criteria were used to determine duration of water intake (Figure 3h). I don't know what 'Times of drinking water' means or how it was calculated (Figure 3i). Given the importance of this experiment to the overall conclusions of the paper, this must be better explained.

Response:

We appreciate your important comments regarding our experimental methods and the details of data presentation. We fully agree that the completeness of methodological description is

crucial for ensuring the reproducibility of experiments and the reliability of conclusions. Regarding the methodological details of drinking behavior analysis in Fig. 3f-i, we provide the following explanation, which has been supplemented accordingly in the Methods section (Lines 175-186) and figure captions of the manuscript:

Drinking behavior was recorded using a multimodal synchronous system. Specifically, a water bottle equipped with a stainless steel ball and a pressure-sensing rod was connected to a 1401 digital-to-analog converter via a lickometer module. All licking signals were acquired in real time by Spike2 software, with simultaneous video recording. Water consumption was calculated by measuring the weight difference of the water bottle before and after the experiment.

During data analysis, a continuous and regular cluster of signals recorded by Spike2 was defined as one drinking event, with its duration measured from the first signal to the last signal. The number of drinking events was counted as the total number of such independent signal clusters, and the total drinking duration was calculated as the cumulative sum of the durations of all events.

To exclude nonspecific signals, irregular signals generated by accidental contact of the bottle during the mouse's free movement were discarded. All recordings were cross-verified with synchronized video to ensure they corresponded to actual licking behavior. In addition, it was confirmed that no spontaneous leakage occurred from the water bottle throughout the experiment.

Question 10: Figure 3m and 6l, it is possible that the impact of citric acid and high salt is due to adding fluid or pressure onto the pharynx, just like water. It seems that this result could simply be explained by mechanosensation.

Response:

Thank you for raising this important point. We understand your concern that the citric acid and high salt solution used in the experiment may produce effects similar to water due to

mechanical stimulation. In this regard, we would like to clarify that all chemical solutions—including citric acid and high salt solution—were perfused into the pharyngeal region at a constant, low flow rate (0.1 mL/min). This flow rate is sufficiently slow to avoid significant mechanical pressure changes induced by fluid movement, thereby minimizing nonspecific activation of pharyngeal mechanoreceptors (as referenced in the Methods section of Prescott, 2020). We have also supplemented the explanation in the Methods section (Line 325-328).

Question 11: The fiber photometry experiment in figure 4 d-g is not adequately described. The methods need to include details like the creation of the oral infusion catheter and the performance of the infusion. At the moment, the reader must guess what the authors have done which makes the interpretation of the data dubious. I assume the authors infused a small volume of water into the mouth (how much? what solution?), recorded the mouse swallowing and aligned the photometry signal to the swallow event however the methods are far too vague. Further clarification is needed on what the infused volume was? what was the latency from infusion to swallow? did all infusions result in a swallow? how many infusions were considered per mouse? which EMG features were used to define swallow onset?

Response:

We appreciate your valuable comments. We fully agree that a comprehensive description of the methodology is essential for the reproducibility of the experiments. In response to the concerns regarding the fiber photometry recording experiments in Fig. 4d-g (new Fig. 4d-f and Supplementary Fig. 7b), we have now provided the following detailed additions to the Methods section (Lines 217-226, 250-256 and 259-263):

(1) The metal cannula (outer diameter 0.5 mm) was pre-bent at a specific angle approximately one- third of its length from the tip and connected to a silicone tube (~5 cm in length, outer diameter 0.54 mm). The metal cannula was vertically fixed to the surface of the mouse skull using dental cement. A ~5 mm incision was made in the skin on the right side of the face. The sterilized silicone tube was passed subcutaneously into the oral cavity and secured in place

with a 7-0 nylon suture on the oral side. Approximately 3 mm of the silicone tube was retained inside the oral cavity, and after trimming to an appropriate length, the skin incision was sutured closed.

(2) During recording, a long silicone tube was used to connect the fixed metal cannula to a syringe needle mounted on a microinjection pump. The entire tubing system was filled with liquid beforehand. A volume of 20 μL of distilled water was infused into the oral cavity at a constant rate of 10 μL/s. All infusions successfully elicited swallowing behavior, with a latency of less than 10 s from the start of infusion to the onset of swallowing. Each mouse received 2-3 independent infusions.

(3) A 3-s light-pulse-triggered square-wave signal was recorded simultaneously with video, electromyography (EMG), and GCaMP fluorescence signals to enable temporal alignment of the multimodal data. The onset of swallowing was defined as the starting point of the characteristic burst discharge in the EMG trace.

We believe that these clarifications will provide readers with a clear and reproducible experimental protocol. Thank you once again for your careful and thorough review.

Question 12: There is no histology to show the injection site of HSV in the pharynx and most HSV expression in the brainstem is outside of the NTS and many of the neurons within the NTS are TH negative.

Response:

Thank you for pointing out the deficiency in histological validation. Following your suggestion, we have supplemented the results verifying the HSV injection sites in the pharynx, as shown in the figure below (Response Document Figure 12, and also see new Supplementary Figure 13a-b). In addition, HSV-positive signals are located within the NTS, and most of them are TH-positive neurons (indicated by yellow arrows). The signals outside the NTS in the original image are not neurons. For this reason, we present the original image as follows.

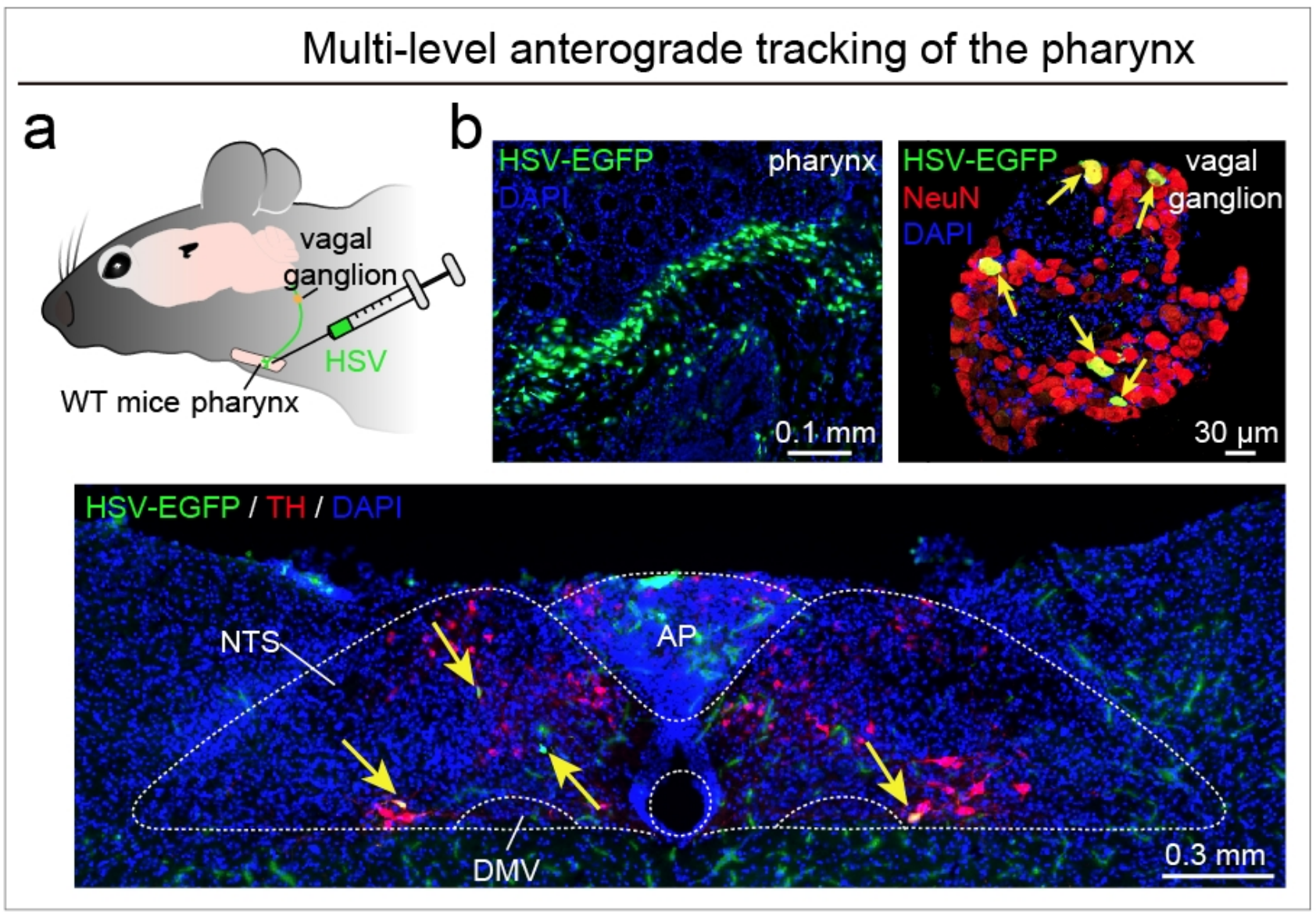

Response Document Figure 12 | Multi-level anterograde tracking of the pharynx. a Schematic diagram of injection HSV into pharynx in *WT* mice. **b** Organ sections showing the expression of HSV-EGFP in the pharynx (left), as well as the distribution of anterograde tracing neurons (yellow arrows) in the vagus ganglion (right), and NTS (bottom).

Question 13: More detail and histology is required about the injection of pharmacological agents into the pharynx. Were they injected into the same 4 sites as the virus detailed in the methods? How were effective concentrations for the drugs calculated and confirmed? How were the injection sites validated?

Response:

Thank you for your meticulous and professional review. In accordance with your feedback, we have provided the following clarifications and additions concerning the specific procedures and validation methods for the pharyngeal drug injection experiments:

(1) **Injection Sites**: The drug injection sites were identical to the viral injection sites described in the Methods section, specifically targeting four predefined locations in the pharynx.

(2) **Dose Selection**: The administered doses of each antagonist (GsMTx4: 0.5 mg/kg; GSK2193874: 1 mg/kg; HC-030031: 0.5 mg/kg; Mifepristone: 1 mg/kg) were primarily based

on the effective doses reported in the product instructions and relevant literature using animal models (e.g., oral gavage, intrathecal, or intraperitoneal injection). Based on this, we conducted gradient experiments and performed Von Frey tests. The results showed that high concentrations inhibiting TRPV4, TRPA1, and TRPM3 were all ineffective in suppressing mechanically stimulated swallowing reflexes, thereby selecting the dosage used in our study.

(3) **Site Verification**: To confirm the accuracy of the injection sites, 1% CTB555 was added to all drug solutions. This tracer allows histological detection of the injection site post-administration (Response Document Figure 13, and also see new Supplementary Figure 15a-c).

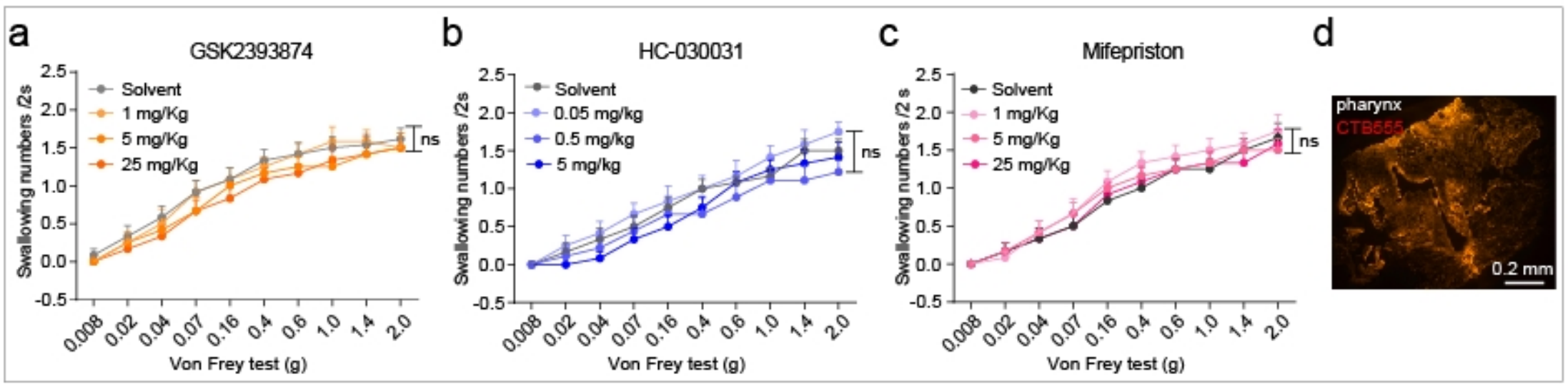

Response Document Figure 13 | Inhibiting the pharyngeal TRPV4, TRPA1, and TRPM3 sensory ion channels had no significant effect on mechanically stimulated swallowing reflex. a-c The number of swallowings induced by mechanical stimulation after inhibition of pharyngeal TRPV4, TRPA1, and TRPM3 ion channels. **d** Pharyngeal tracing of the antagonist injection.

The above supplementary information has been incorporated into the Methods section (Line 416-423). We believe these details better demonstrate the rigor of our experimental approach.

Question 14: Interpretation of the nerve transection results in Figure 8 are once again complicated by unclear reporting in the methods, main text and figure. In figure 8a it appears that the SLN is being illuminated proximal to the transection site. Given the result I find it surprising that illuminating a transected nerve proximal to the cut site would show any effect of cutting the nerve. Was this the case or was the light delivered distal to the transection site?

Response:

Thank you for your thorough review and valuable comments. We apologize for the misunderstanding caused by the insufficient clarity of the schematic diagram, which may have led to unnecessary confusion during your review.

In the experiment shown in Fig. 8a (new Fig. 8d), the light stimulation was indeed applied to the distal end of the transected site. Specifically, we transected the superior laryngeal nerve (SLN) at its brainstem end and subsequently delivered light stimulation to the distal segment (i.e., the peripheral segment) of the transected site. The experimental results showed that under these conditions, light stimulation failed to evoke swallowing behavior.

This finding further supports our conclusion that Piezo2-expressing SLN afferent fibers are essential for initiating the pharyngeal phase of swallowing. As your suggestions, we have redrawn and updated the schematic diagram (Response Document Figure 14, and also see new Figure 8d) to ensure that it accurately and clearly reflects the experimental design.

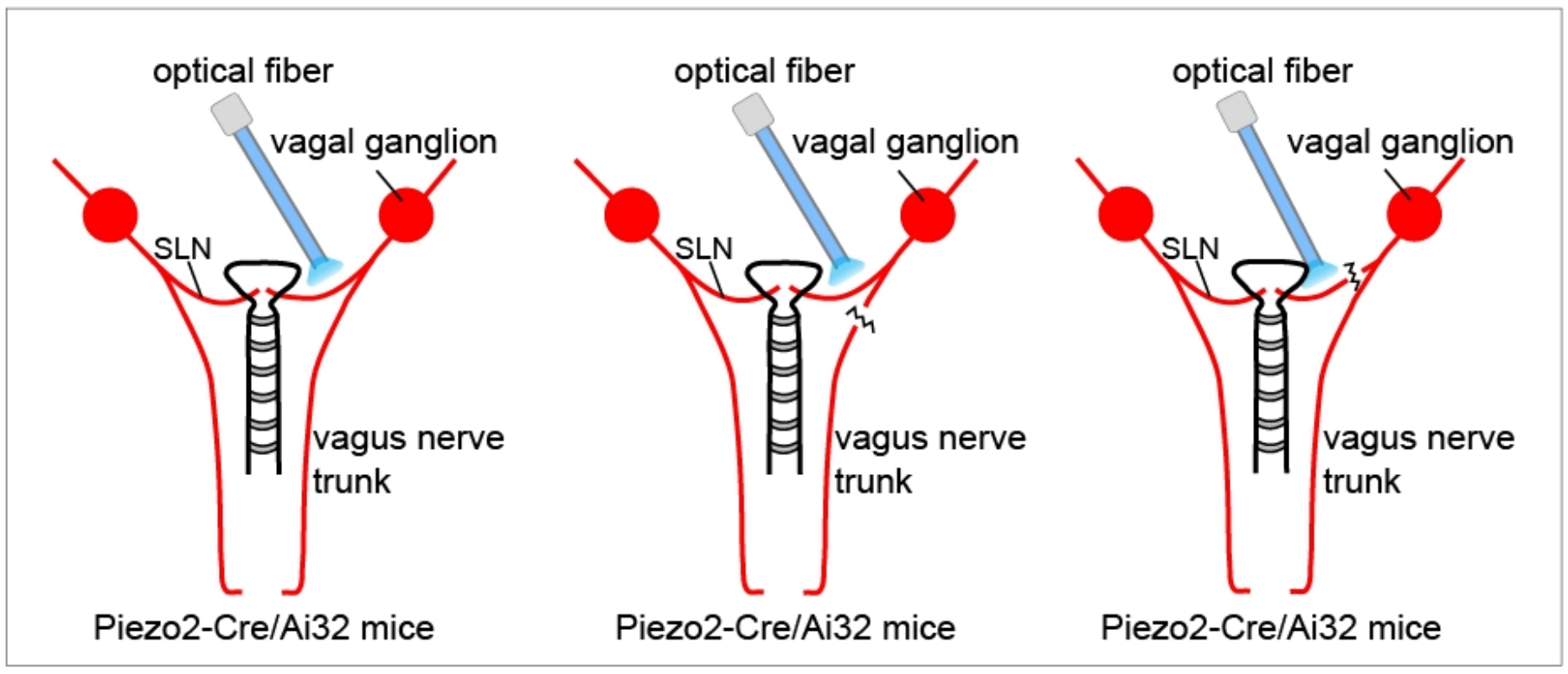

Response Document Figure 14 | Schematic diagram of optogenetic stimulation of the SLN in *Piezo2-Cre/Ai32* mice.

Question 15: Supplemental Figure 12 shows very little data, like what genes are used to define the vagal clusters?

Response:

We sincerely appreciate your constructive feedback regarding the insufficiency of the Smart-seq data in Supplementary Fig. 12 (see new Supplementary Fig. 14), which is of great

significance for improving our manuscript. We used our previously established vagal ganglion neuronal single-cell database (PMID: 38288368). Sequencing data was deposited in the Genome Sequence Archive (GSA) database (GSA accession number: CRA007790). For clustering analysis, instead of using marker genes, we performed clustering based on 9,000 highly variable feature genes with the FindVariableFeatures() function in Seurat. The attachment shows the ranking of differentially expressed genes for each of the 31 clusters, as well as the cluster-specific genes of the RV traced clusters.

Question 16: Figure 7 does not convincingly show a knockout of Piezo2. In fact, panel M still shows immunostaining for Piezo2 in the neurons that express Cre.

Response:

Thank you for your valuable comments. We are deeply sorry for the unclear details in the figure. The representative images presented in our original manuscript were based on immunohistochemical staining of the nodose ganglion from *Piezo2-Flox*(+/-) mice that received pharyngeal injections of Retro-Cre-mCherry. These images were intended to accurately reflect the number of retrogradely labeled neurons from the pharynx (new Fig. 7i).

Furthermore, the Piezo2 knockout analysis presented in the original manuscript was based on whole vagal ganglia levels. To more precisely demonstrate the knockout of Piezo2 in neurons, we isolated the retrogradely labeled neurons and conducted single-cell qPCR analysis (Response Document Figure 15, and also see new Figure 7k). This result provides molecular-level evidence further confirming the successful knockout of Piezo2.

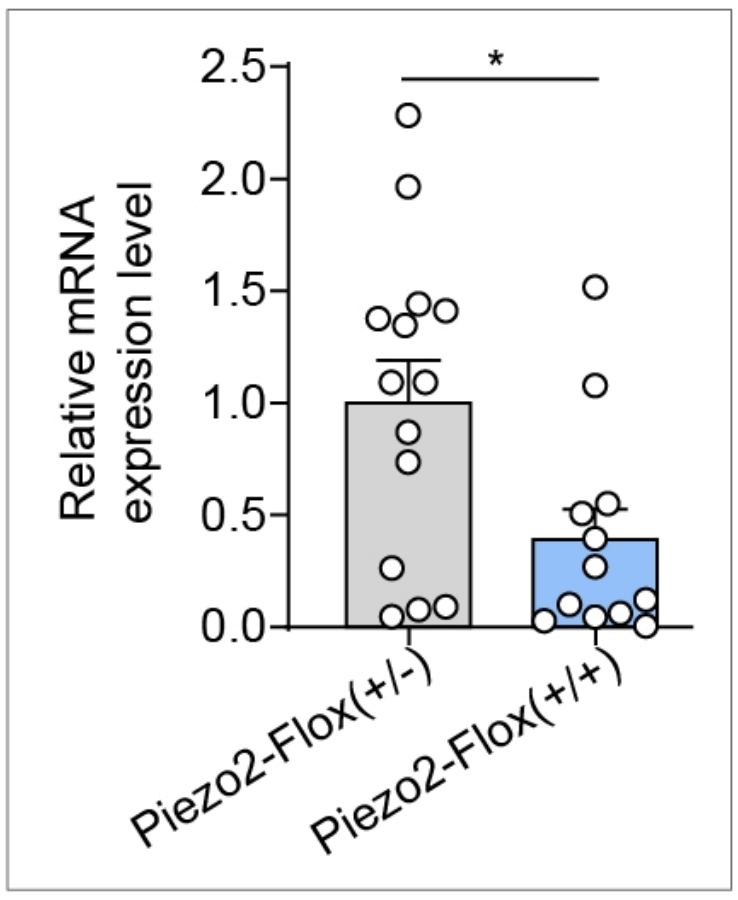

Response Document Figure 15 | Knockout efficiency of Piezo2 in neurons of the vagal ganglia that innervate the pharynx. a Relative mRNA expression level of Piezo2 in vagal ganglion neurons projecting to the pharynx by single-cell qPCR (*Piezo2-Flox*(+/-) group: n=14 cells, *Piezo2-Flox*(+/+) group: n=12 cells).

The above results have been updated to the revised manuscript (Lines 568-573) and the Methods section (Lines 468-489).

Together, these experimental data quantitatively verify the knockout efficiency at the protein and molecular levels, thereby making the conclusions more robust and reliable.

We would like to express our sincere gratitude for your valuable feedback and insightful suggestions, which have significantly enhanced the quality of our manuscript, improved the rigor of the research, and the accuracy of the report.

Reviewer #2 (Remarks to the Author)

The manuscript proposes a vagal, Piezo2-dependent sensory pathway from the pharynx to TH+ neurons in the nucleus of the solitary tract (NTS) that, via projections to the Ambiguus/RVLM complex, triggers swallowing-like behavior. The authors combine Von Frey–evoked swallowing under anesthesia, EMG and pharyngeal pressure readouts, c-Fos mapping, opto/chemo-genetics, fiber photometry, trans-synaptic tracing, SLN transections, pharmacology (GsMTx4), and a retro-AAV Piezo2 knockout strategy. Overall, the study addresses an important and understudied aspect of swallowing neurobiology (mechanosensory triggering). However, there are some shortcomings that need to be addressed.

1. GsMTx4 is a broad blocker of stretch-activated channels and not selective for Piezo2. The observed suppression after GsMTx4 supports a role for mechanosensitive channels but does not isolate Piezo2. Conversely, the other antagonists used (e.g., mifepristone for TRPM3) have known off-targets. The pharmacology thus supports “mechanotransduction dependence” more than “Piezo2 specificity.” The genetic KO strengthens the case but is partial (~50% mRNA knockdown) and leaves residual responses.

Response:

We appreciate your valuable suggestions. Regarding the specific question on pharmacological specificity, we have clearly stated in revised manuscript (Lines 526-548). The pharmacological effects of inhibiting pharyngeal sensory ion channels by Piezo family mechanosensitive ion channel antagonist GsMTx4, TRPA1 antagonist HC-030031, TRPV4 antagonist GSK2193874, and TRPM3 antagonist mifepristone collectively demonstrated that mechanically stimulating the pharynx to induce swallowing-like behavior was mechanotransduction-dependent. Vagal ganglion neurons primarily express the Piezo2 mechanosensitive ion channel. To further identify the key channel type involved, we adopted a genetic approach by specifically knocking out Piezo2 in vagal ganglion neurons that innervate the pharynx.

The detailed experimental strategy and validation are as follows: We used *Piezo2-Flox*(+/+)

mice and delivered AAV2/2Retro-Cre-mCherry virus via local injection into the pharynx. This allowed us to both label pharynx-innervating neurons and conditionally knockout Piezo2 in these cells. *Piezo2-Flox*(+/-) mice served as controls. Two-three weeks after viral expression, we performed single cell qPCR on the labeled (mCherry-positive) neurons to assess Piezo2 mRNA levels. The results showed that Piezo2 mRNA expression in the experimental group was reduced by approximately 61% compared to the control group (Response Document Figure 15, and also see new Figure 7k). This directly supports the effectiveness of our strategy, demonstrating a specific and highly efficient knockdown of Piezo2 in the functionally relevant neuronal population.

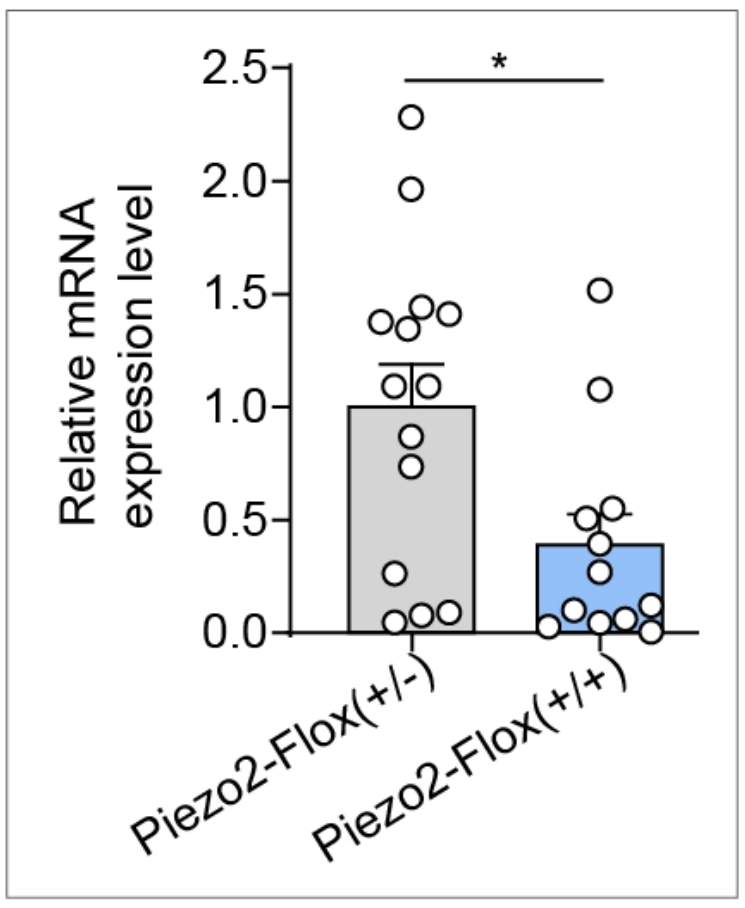

Response Document Figure 15 | Knockout efficiency of Piezo2 in neurons of the vagal ganglia that innervate the pharynx. a Relative mRNA expression level of Piezo2 in vagal ganglion neurons projecting to the pharynx by single-cell qPCR (*Piezo2-Flox*(+/-) group: n=14 cells, *Piezo2-Flox*(+/+) group: n=12 cells).

The Piezo2 knockout analysis has been updated from whole-vagus ganglion level in the original manuscript to single-cell level in order to more precisely demonstrate the successful knockout of Piezo2. The above results have been updated to the revised manuscript (Lines 568-573) and Methods section (Lines 468-489).

2. While SLN transection severely impairs evoked swallowing, the literature and introduction note roles for glossopharyngeal (GPN) afferents in pharyngeal swallowing. The study does not test GPN transection or combined lesions; thus, the exclusive primacy of SLN Piezo2

afferents in pharyngeal mechanosensation remains suggestive rather than definitive.

Response:

Thank you for your valuable suggestions. To clarify the relative role of superior laryngeal nerve (SLN) afferent nerve bundle in mechanically evoked swallowing and to assess the potential contribution of the glossopharyngeal nerve (GPN), we systematically supplemented the original SLN transection experiments with additional unilateral/bilateral GPN transections and combined lesions of the GPN and SLN.

The detailed experimental protocol is as follows: In addition to the unilateral and bilateral SLN transections described in the manuscript, the following treatment groups were newly added: (1) left GPN transection; (2) right GPN transection; (3) bilateral GPN transection; (4) bilateral GPN transection combined with left SLN transection; (5) bilateral GPN transection combined with right SLN transection; (6) bilateral transection of both the GPN and SLN. In each group, during a post-operative stabilization period, swallowing was evoked by applying a series of Von Frey filament stimuli with calibrated forces to the pharynx, and the number of swallowing was recorded for each stimulus intensity (Response Document Figure 16, and also see new Figure 8c).

Preliminary experimental results indicated that: Unilateral and bilateral transection of GPN both caused varying degrees of damage to mechanically stimulated reflexive swallowing. After transection of bilateral GPN combined with unilateral SLN (left or right side), reflexive swallowing could be induced by high-threshold mechanical stimulation intensity, but was otherwise almost impossible to elicit. Bilateral GPN and bilateral SLN transection completely eliminated the swallowing reflex evoked by pharyngeal mechanical stimulation. Overall, the swallowing triggered by mechanical stimulation decreased progressively after isolated GPN transection and after combined GPN and SLN transection. In other words, the degree of impairment of mechanically stimulus-induced reflexive swallowing increased gradually with unilateral (left or right) GPN transection, bilateral GPN transection, bilateral GPN combined with unilateral (left or right) SLN transection, and bilateral GPN combined with bilateral SLN transection (Line 590-597).

The details of the above experiment have been added to the Methods section (Lines 427-436).

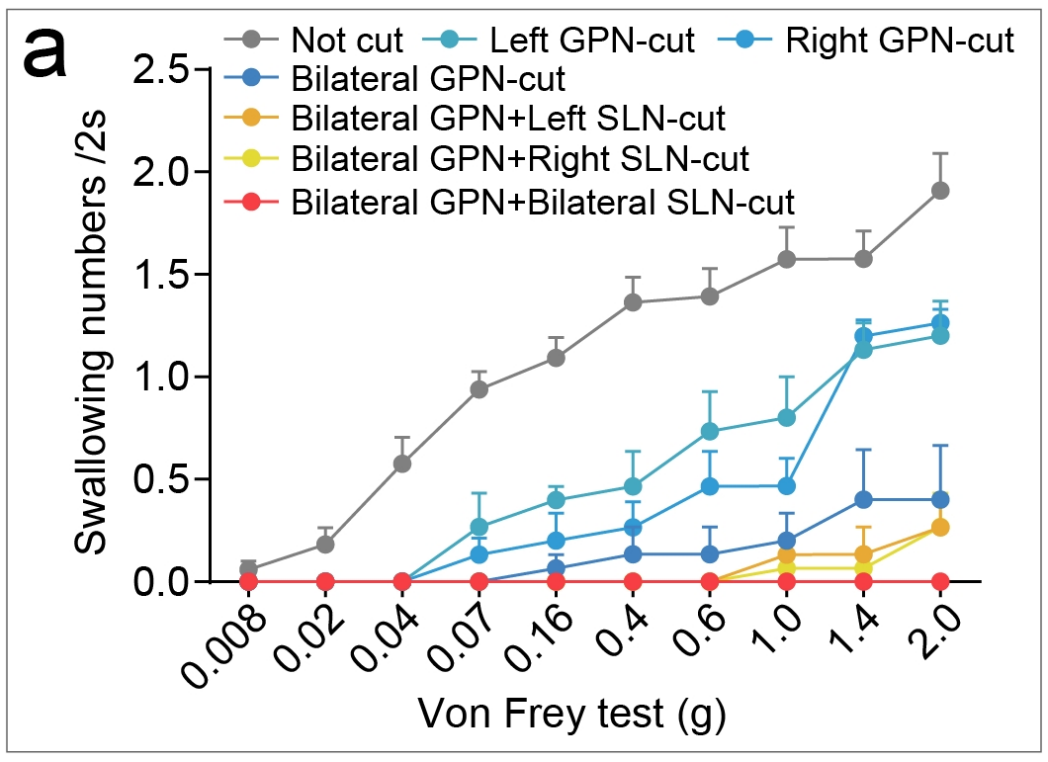

Response Document Figure 16 | Mechanical stimulation-induced swallowing reflex damaged by transection of GPN and transection of GPN combined with SLN. a Total number of swallowing behaviors induced by mechanical stimulation within 2 s of cutting the left, right and bilateral GPN, bilateral GPN combined SLN lesions in *WT* mice, respectively (Not cut: n=11 mice; else each group: n=4 mice).

3. Many mechanosensory tests and opto-evoked outputs are under anesthesia. Although the authors include head-fixed water-evoked swallowing and projection-specific photometry, the key causal tests (e.g., terminal opto at Amb/RVLM driving swallows) are anesthetized. Demonstrating necessity/sufficiency in awake animals would further validate physiological relevance. A suggestion: test opto-inhibition of TH+ NTS or TH+ NTS→Amb/RVLM during awake voluntary drinking to show necessity under natural conditions (e.g., stGtACR2 or ArchT).

Response:

Thank you for your valuable suggestions. We fully agree that verifying neural circuits in awake, natural behavioral states is essential for establishing their physiological relevance.

Following your recommendation, we systematically conducted two additional optogenetic inhibition experiments during voluntary drinking behavior in awake animals:

(1) Inhibition of TH+ NTS neurons: AAV-DIO-hGtACR1-mCherry was injected into the NTS

of *TH*-ires-Cre mice, and an optical fiber was implanted above the injection site. 2-3 weeks after viral expression, in an awake, free-drinking behavioral paradigm, blue light was delivered immediately upon the initiation of drinking to inhibit TH+ neuronal activity. Compared with control periods without light inhibition, optogenetic suppression of TH+ neurons significantly reduced the amount of water consumed (Response Document Figure 17, and also see new Figure 3n-p).

(2) Inhibition of TH+ NTS→Amb/RVLM projections: Again using *TH*-ires-Cre mice, AAV-DIO-GtACR1-mCherry was injected into the NTS, and an optical fiber was implanted above the Amb/RVLM. 2-3 weeks after viral expression, during the same awake, free-drinking paradigm, blue light was delivered upon drinking onset to inhibit axon terminals of TH+ NTS neurons projecting to Amb/RVLM. Similarly, compared with no-light conditions, inhibiting this specific projection significantly reduced water intake (Response Document Figure 17, and also see new Figure 5o-q).

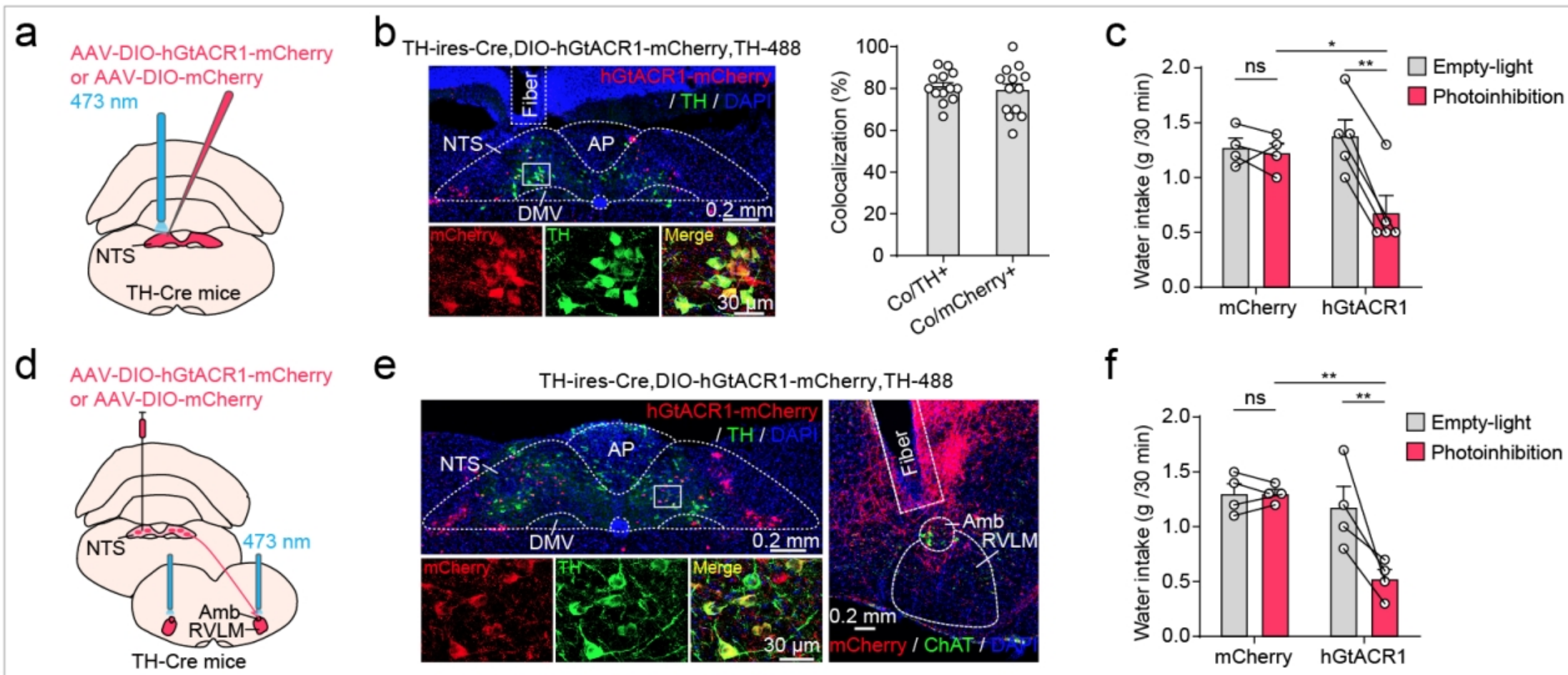

Response Document Figure 17 | Optogenetic inhibition of TH+ NTS neurons and axon terminals of TH+ NTS neurons projecting to Amb/RVLM significantly reduced mouse water intake. a Schematic diagram illustrating the sites of hGtACR1-mCherry injection and optical fiber implantation in the NTS of *TH*-ires-Cre mice. **b** Example micrographs showing hGtACR1-mCherry expression and the optical fiber track above hGtACR1-mCherry+ neurons in the NTS (left, top). Example micrographs (left, down) and statistical data (right) showing colocalization of hGtACR1-mCherry+ (red) with the TH+ neurons (green) from the NTS. **c** Water intake of mice after optogenetic inhibition of TH+ NTS neurons (n=4-5 mice).

d Schematic diagram illustrating the sites of hGtACR1-mCherry injection in the NTS and optical fiber implantation in the Amb/RVLM of *TH*-ires-Cre mice. **e** Example micrographs showing hGtACR1-mCherry expression in the NTS and the optical fiber track above the terminals of hGtACR1-mCherry+ neurons projecting to the Amb/RVLM. **f** Water intake of mice after optogenetic inhibition of TH+ NTS-Amb/RVLM projections (n=4 mice).

These supplementary experimental results indicated that during natural drinking behavior in awake animals, TH+ NTS neurons and their projections to the Amb/RVLM are necessary for swallowing initiation. The results have been updated to the revised manuscript (Line 284-295 and 411-419). Relevant experimental details have been synchronously supplemented in the Methods section (Lines 189-198).

4. Chemical stimuli are applied to the larynx, whereas mechanical stimuli are applied to the pharynx; both converge on TH+ NTS→Amb/RVLM. The conclusion of a unified "shared circuit" is attractive but could be strengthened by matching stimulus sites (e.g., pharyngeal chemical stimuli) and/or by directly recording upstream afferent population responses.

Response:

Thank you for tyour insightful and constructive comments. The primary focus of our current study is to demonstrate that the TH+ NTS neurons and their downstream pathway in the NTS—specifically, the TH+ NTS→Amb/RVLM projection—serves as a shared downstream pathway for both mechanical and chemical stimuli. This finding itself carries significant scientific value. We fully agree that clarifying whether chemical and mechanical stimuli converge onto TH+ NTS neurons via the same upstream afferent population is crucial for establishing the existence of a "unified shared circuit". We respond to the two recommendations as follows:

First, regarding the technical challenge of matching the stimulation site (e.g., applying chemical stimuli to the pharynx): We share your view that this would be one of the most direct experiments to verify convergence. However, given the close anatomical proximity between the pharynx and larynx in mice, the current method of delivering chemical stimuli

via laryngeal perfusion cannot reliably restrict stimulation to the pharynx without involving the larynx. We acknowledge this technical limitation and are actively exploring and refining more precise local stimulation approaches.

Second, regarding direct recording of responses in the upstream afferent fiber population: As the reviewer rightly points out, whether convergence also occurs at the level of upstream afferent population is indeed important; however, we have not yet investigated this aspect. We highly value your perspective and have accordingly addressed this limitation in the Discussion section (Line 692-714) of the manuscript. We explicitly state that the convergence characteristics of upstream afferent inputs remain an important open question for future research. We plan to make this a central focus of follow-up studies, aiming to provide a more comprehensive understanding of the complete circuit mechanisms from peripheral receptors to central integration.

Once again, we sincerely thank you for helping enhance the depth and rigor of this study.

5. How do you know that evoked events meet clinical swallowing criteria rather than generalized upper-airway reflexes?

Response:

Thank you for raising this critical point. We fully agree that rigorously distinguishing swallowing from simple upper airway reflexes in animal models is essential. Below we provide clarifications and justifications based on clinical standards and our experimental design:

Clinical standards for detecting swallowing include: Fiberoptic Endoscopic Evaluation of Swallowing (FEES), which assesses pharyngeal and laryngeal structures; Videofluoroscopic Swallowing Study (VFSS), which measures pharyngeal transit time, esophageal transit time, and bolus size; Standardized Swallowing Assessment (SSA), which evaluates consciousness, postural control, breathing, lip closure, laryngeal function, pharyngeal reflex, and spontaneous cough; Kubota Water Swallowing Test (WST), which assesses the presence of post-swallow

coughing or a "wet voice"; and Pharyngeal Surface Electromyography (PsEMG), which quantifies features of extrinsic musculature via root mean square, integrated EMG, and average EMG.

However, these techniques are not yet widely adopted in rodent experiments. Recently, Lever's laboratory at the University of Missouri School of Medicine has developed equipment for transoral endoscopy (FEES) and videofluoroscopic swallowing studies (VFSS) in rodents, but these have not become routine experimental approaches.

In our study, we employed pharyngeal pressure monitoring and mylohyoid electromyography (EMG) as physiological indicators to detect swallowing. Although these provide indirect evidence, when combined with the twine swallowing test and voluntary drinking experiments, they support the conclusion that the observed behavior is not merely an upper airway reflex.

Minor comments

1. The abstract states that "Either ablation of TH+ NTS neurons or Piezo2 knockout in vagal sensory neurons both could abolish swallowing reflex" but the Results show chemogenetic inhibition (hM4Di) and not true ablation. If ablation data exist, they are not presented; if not, the abstract should be revised to "inhibition" to avoid overstatement.

2. The causality language is too strong. Please tone down.

Response:

Thank you for your valuable correction regarding the precision of the manuscript's wording. We fully agree with your comments and have implemented the following revisions in the manuscript:

(1) **Correction of the term "ablation":** We confirm that the study employed chemogenetic inhibition (hM4Di) and did not involve neuronal ablation experiments. Accordingly, we have replaced all instances of "ablation" in the abstract of the manuscript with "inhibition" (Line 26).

(2) **Toning down causal language:** We have thoroughly reviewed and revised overly absolute statements of causality throughout the manuscript.

1) Integrating the results of GPN sectioning or combined SLN damage on mechanically stimulated swallowing indicates that SLN afferent fibers play an important role in pharyngeal mechanical sensation (Line 618-620).

2) Pharmacological results demonstrated that mechanically stimulating the pharynx to induce swallowing-like behavior was mechanotransduction-dependent (Lines 546-548).

Once again, we sincerely appreciate your constructive feedback, which has helped enhance the accuracy of our paper.

Reviewer #3

In this manuscript by Dr. Pei and colleagues, the authors first stimulated the pharynx using von Frey filaments to trigger swallowing, which induced c-Fos expression in the NTS. Optogenetic or chemogenetic activation of TRAPed NTS neurons also elicited swallowing. The study then identifies these neurons as TH-positive NTS neurons. Inhibition of TH-positive NTS neurons impaired both mechanically and chemically induced swallowing. The authors further demonstrate that TH-positive neurons are glutamatergic and excitatory, and that they are activated during water-induced swallowing. They next examined downstream projections of TH-positive neurons and show that the Amb/RVLM pathway mediates swallowing. The nodose–NTS circuit is mapped, revealing that TH-positive neurons receive input from Piezo2-positive nodose neurons. Lastly, the authors show that Piezo2 deletion reduces mechanically induced swallowing, and optogenetic stimulation of Piezo2-positive fibers triggers swallowing.

An impressive amount of data is presented in this manuscript. Much of the work appears technically solid, with appropriate controls and quantification, and the experiments are generally designed with care.

My principal concern relates to conceptual novelty. Many of the major findings concerning TH-positive NTS neurons in swallowing have already been reported, notably in Wang et al., 2024, from Dr. Ling Bai's group. A substantial portion of the current study reproduces previously published observations. While it is reassuring to see these results replicated with rigorous experimental design, the conceptual advance is limited. The demonstration that stimulation of TH-positive neurons triggers swallowing and that their inhibition abolishes swallowing is new, but largely expected. The TH→Amb projection is indeed novel. The role of Piezo2 in mechanical swallowing is new as well but appears inconsistent with earlier reports and requires further validation. Overall, the authors should more clearly acknowledge what is already known and highlight what is genuinely new in their work. Additional major concerns are listed below.

We appreciate your careful reading of our manuscript and insightful and constructive

comments. We fully agree with your point regarding the need to more clearly define the novelty of our study. In the revised manuscript （Line 755-797）, we have discussed the work by Wang (2024) and other relevant prior studies more comprehensively, and have placed greater emphasis on highlighting the unique contributions of our research.

Wang's (2024) study indeed found that TH cNTS neurons encode esophageal mechanical distension and bolus volume, thereby enabling rapid feedback regulation of feeding rate. This represents a physiological function of TH cNTS neurons.

The swallowing process is divided into three phases, including the oral phase, pharyngeal phase, and esophageal phase. The scientific hypothesis we propose is that TH+ NTS neurons mediate the pharyngeal swallowing reflex triggered by mechanical stimulation of the pharynx. Our further TH+ NTS neuronal dynamics results and drinking experiment data are consistent with the observation by Wang (2024) that TH+ NTS neurons encode instantaneous swallowing volume. It is precisely during the oral phase of the swallowing process that the masticated food bolus is propelled into the pharynx, where it is detected by TH+ NTS neurons, thereby triggering the swallowing reflex.

The innovative findings of this study are as follows: 1) We identified that the Piezo2 mechanosensitive channel, expressed in primary sensory neurons of the vagal ganglia, is critically important as the key mechanoreceptor for detecting pharyngeal stimuli and triggering swallowing; 2) We clarified that the TH+ NTS neuron subpopulation projecting to the RVLM is a key neuronal subtype for the swallowing reflex; 3) We discovered that the TH+ NTS-Amb/RVLM pathway is a shared downstream pathway for mechanically and chemically induced reflexive swallowing. Therefore, the main contribution of this study is to reveal that the Piezo2 mechanosensitive channel, expressed in primary sensory neurons of the the vagal ganglia, and SLN nerve bundles perceive pharyngeal mechanical stimuli, as well as the specific relay pathways of pharyngeal mechanical sensory information in the brainstem (i.e., TH+ NTS neurons and their specific projections to the RVLM), and to demonstrate their necessity for reflexive swallowing.

1. It is difficult to reconcile the claim that mechanical stimulation of the pharynx activates

most TH-positive neurons, given that this is a large and heterogeneous NTS population. Are there spatial patterns along the rostral–caudal axis?

Response:

Thank you very much for valuable comments. We fully agree that clarifying the spatial distribution pattern of the activated TH+ neurons within the NTS is crucial for understanding their functional role in the swallowing reflex.

In response to your point that the claim "pharyngeal mechanical stimulation activates the majority of TH-positive neurons" may be questionable, and following the specific suggestion to analyze the spatial distribution pattern, we have redesigned and completed supplementary experiments. We systematically analyzed the distribution of c-Fos and TH co-labeled neurons in the caudal, intermediate, and rostral subregions of the NTS following swallowing evoked by mechanical stimulation of the pharynx. Specifically, we divided the NTS into these three continuous regions according to the brain atlas and acquired high-resolution images from equally spaced sections within each corresponding region for every mouse. By counting the total number of TH+ neurons and the number of c-Fos+/TH+ co-labeled neurons in each subregion, we calculated the proportion of activated neurons (c-Fos+/TH+) within each subdivision (Response Document Figure 18, and also see new Supplementary Figure 4d).

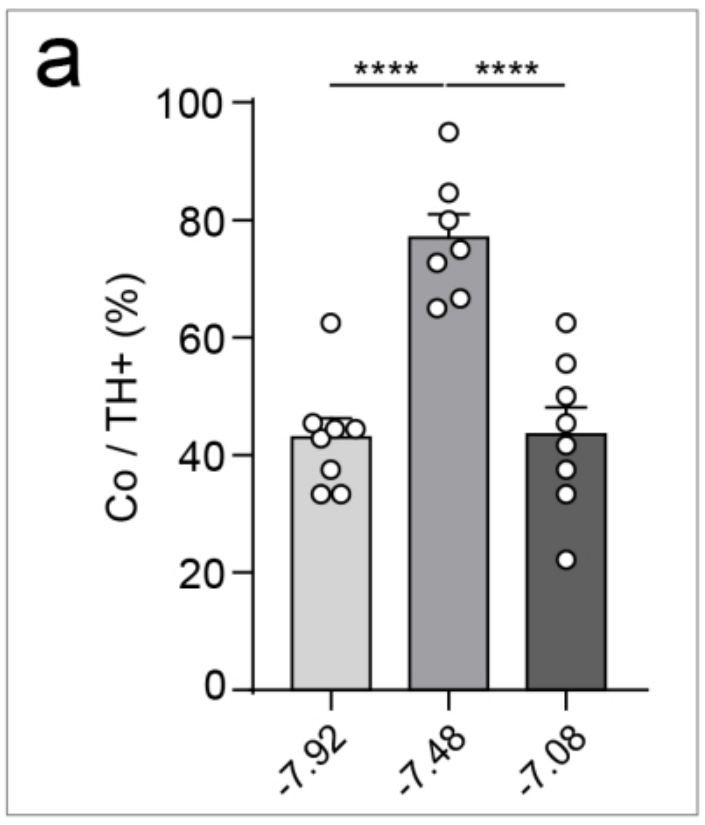

Response Document Figure 18 | The proportion of activated neurons (c-Fos+/TH+) in the caudal, intermediate, and rostral subregions of the NTS following swallowing evoked by mechanical stimulation of the pharynx.

The results show that after pharyngeal mechanical stimulation, the activated TH+ neurons

were not uniformly distributed but exhibited significant regional enrichment, primarily within the intermediate subregion of the NTS. This finding supports your perspective that the population of TH+ neurons in the NTS is spatially heterogeneous and clarifies that pharyngeal sensory input mainly activates a specific neuronal subset located in the intermediate subregion.

We have incorporated these new spatial analysis data into the revised manuscript (Line 200-211) and modified the relevant textual descriptions to more accurately reflect the activation pattern of TH+ neurons. We sincerely thank you for this insightful suggestion, which has greatly enhanced the rigor and completeness of our conclusions.

2. The results described in Fig. 3g–i are unclear. The number of swallowing events should be explicitly monitored and quantified.

Response:

Thank you for your important comment. We fully agree that directly monitoring and quantifying swallowing events is crucial for accurately evaluating the function of TH+ NTS neurons.

In the original Fig. 3g-i, we inferred swallowing function using three indirect behavioral metrics: water intake, duration of water intake, and times of drinking water. Specifically: water intake was measured by weighing; drinking behavior was recorded using a contact sensor. In the Spike2 system, continuous and regular clusters of electrical signals were defined as a single drinking event, with its duration measured from signal onset to offset. Times of drinking water was the total count of such events, and duration of water intake was the sum of all event durations. Non-specific interference signals (e.g., accidental contact) were excluded during analysis (Method Section Line 175-186). Based on these data, we found that inhibiting TH+ NTS neurons did not affect motivation to drink (as the number and duration of drinking bouts remained unchanged) but impaired swallowing efficiency (reflected by reduced water intake).

As you have pointed out, these metrics provide indirect reflections of swallowing. To enable direct and objective assessment of swallowing function, we have followed your suggestion and performed a key supplementary experiment: during the free-drinking paradigm, we simultaneously recorded electromyographic (EMG) activity from the mylohyoid muscle in mice. This EMG signal serves as a direct electrophysiological marker of swallowing movements, allowing precise quantification of the number of swallowing events (Response Document Figure 19).

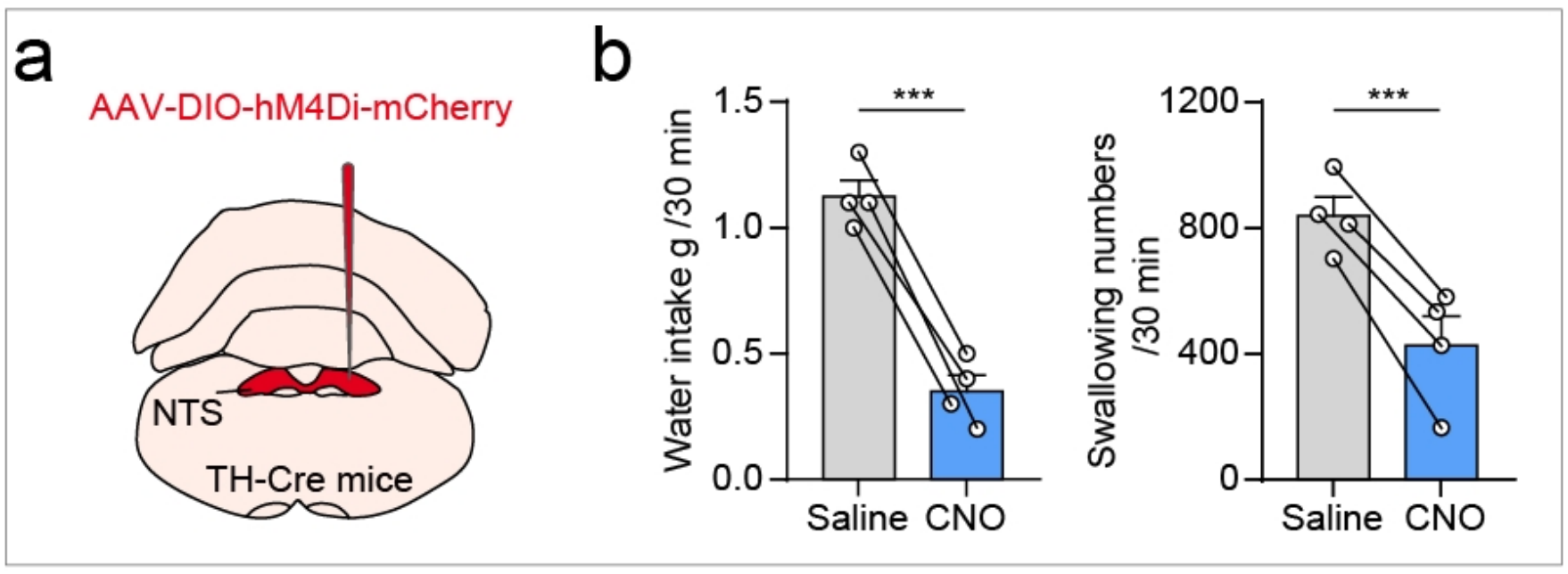

Response Document Figure 19 | Chemogenetic inhibition of TH+ NTS neurons reduced swallowing frequency during free drinking. **a** Schematic diagram showing injection site of AAV-DIO-hM4Di-mCherry in the NTS of *TH*-ires-Cre mice. **b** Water intake and swallowing frequency within 30 min in mice following chemogenetic inhibition of TH+ NTS neurons (n=4 mice).

3. The data in Fig. 4b are confusing. Were the ChR2-mCherry-negative NTS neurons patched at random? What proportion of mCherry-negative neurons receive direct input from mCherry-positive TH-positive neurons? Furthermore, while these electrophysiological experiments are elegant, the neurochemical identity of TH-positive NTS neurons has already been well characterized in previous profiling studies, such as in Want et al 2024, Su et al 2024, Ludwig et al 2021.

Response:

Thank you for your comment regarding Fig. 4b. We would like to clarify the recording strategy and data as follows: In NTS brain slices from *TH*-Cre mice, we randomly selected

ChR2-mCherry-negative neurons for patch-clamp recording. Out of the total 17 mCherry-negative neurons recorded, excitatory postsynaptic currents were detected in 8 neurons upon optogenetic stimulation of TH-positive axon terminals. This indicates that approximately 47.1% of the randomly recorded ChR2-mCherry-negative neurons receive monosynaptic excitatory input from ChR2-expressing TH-positive neurons.

We agree with you that the transcriptomic profiles of TH-positive NTS neurons have been previously studied (e.g., Wang *et al*., 2024; Su *et al*., 2024; Ludwig *et al.*, 2021). The primary goal of our electrophysiological experiment is not to re-identify their neurotransmitter phenotype, but rather to functionally confirm the excitatory synaptic transmission properties of this neuronal population within a specific swallowing-related circuit. This provides cellular physiological evidence supporting our subsequent finding that their activation is sufficient to drive downstream neuronal activity and behavior.

4. The findings described in Fig. 4e–f have been previously reported in Bai and Knight.

Response:

Thank you for this important point, which have helped us to more prominently highlight the focus of our research. The dynamics of TH+ NTS neurons during water-induced swallowing in the original manuscript's Fig. 4e-f are consistent with the results of Bai *et al*. Nevertheless, more meaningfully, we identified a mechanosensory pathway that is crucial for the reflexive pharyngeal swallowing induced by mechanical stimulation of the pharynx under anesthesia. The intensity of Von Frey stimulation of the pharynx that can be precisely controlled simulated the size of food boluses, as reflected in the dynamics of TH+ NTS neurons recorded (Fig. 4g and Supplementary Fig. 7a). Two independent teams of we and Bai consistently demonstrated that TH+ NTS neurons were responsible for perceiving mechanical stimulation in the pharynx through distinct mechanical stimulation modalities. We have adjusted Fig. 4f from the original manuscript to Supplementary Fig. 7b to more clearly illustrate the important conclusion that TH+ NTS neurons mediate pharyngeal mechanical stimulation-triggered swallowing reflex. The corresponding results statement has been

synchronously updated to the revised manuscript (Line 315-338).

5. The identification of 31 clusters of vagal sensory neurons requires clarification. It is unclear which dataset was used for this analysis and what gene signatures are expressed by the RV-traced neurons. The meaning of "expression level of the DsRed-labeled neurons" should be explicitly defined.

Response:

We sincerely appreciate your suggestions regarding the supplementary detailed description of Smart-seq, which is of constructive significance for improving our manuscript. We used our previously established vagal ganglion neuronal single-cell database (PMID: 38288368). Sequencing data was deposited in the Genome Sequence Archive (GSA) database (GSA accession number: CRA007790). For clustering analysis, instead of using marker genes, we performed clustering based on 9,000 highly variable feature genes with the FindVariableFeatures() function in Seurat. The attachment shows the ranking of differentially expressed genes for each of the 31 clusters, as well as the cluster-specific genes of the RV traced clusters.

Furthermore, we apologize for the inappropriate wording in the figure captions. We have revised the caption to: Transcriptome similarity analysis of DsRed labeled vagal ganglion neurons from TH+ NTS neurons with vagal ganglion neural clusters, and dotplot of these signature scores.

6. I have significant concerns regarding the data in Fig. 7. Given the limited efficiency of AAV-retro targeting, and the fact that Piezo2-positive nodose neurons innervate multiple organs, it is not plausible to observe a 50 percent reduction in Piezo2 RNA expression at the whole-ganglion level. The specificity of the Piezo2 antibody also requires demonstration. Moreover, as shown both in earlier studies and in this manuscript, nodose neurons responding to chemical stimuli (water, acid) also project to the pharynx and can trigger swallowing. It is

therefore difficult to reconcile the finding that all retrogradely labeled nodose neurons are Piezo2-positive.

Response:

Thank you for your insightful and important concerns regarding the data in Fig. 7, which prompted us to perform more rigorous supplementary validation of the related analyses.

(1) Regarding the query about the significant decrease in Piezo2 RNA expression observed at the whole nodose ganglion level: We understand that since AAV-retro transduction is not 100% efficient, and Piezo2-positive neurons in the nodose ganglion widely innervate multiple organs, the magnitude of change at the whole-tissue RNA level requires reasonable interpretation. In our experiments, this significant decrease (~50%) likely reflects the combined effects of the following factors: 1) The AAV-retro-Cre we injected locally into pharyngeal muscles achieved relatively high infection efficiency, thereby specifically and effectively targeting the neuronal population innervating this region; 2) Neurons innervating the pharynx may constitute a considerable proportion of sensory neurons in the vagal ganglion; 3) qPCR detection itself is highly sensitive. More importantly, we have supplemented single-cell-level qPCR data: by performing micromanipulation to aspirate individual retrogradely labeled (mCherry-positive) neurons for detection, we directly confirmed effective knockdown of Piezo2 mRNA in Cre-expressing neurons at the molecular level. This supplementary data provided more direct evidence for the specificity and efficacy of the gene knockout (Response Document Figure 20, and also see new Figure 7k).

(2) Regarding the specificity of the Piezo2 antibody: The Piezo2 antibody used in this study (Catalog #NBP1-78624, Novus) has been employed for staining mouse neural tissues in multiple published research papers (e.g., Jia *et al.*, 2016; Narayanan *et al.*, 2016; Nencini and Ivanusic, 2017; Narayanan *et al.*, 2018; Lu *et al.*, 2020), and its applicability has been validated. We have added detailed information about this antibody and relevant citations in the Methods section (Line 377-379).

(3) Regarding the statement that "all retrogradely labeled neurons were Piezo2-positive" and its potential contradiction with chemosensitive neurons: We thank the reviewer for pointing

out the inaccuracy of this statement. In the original manuscript, the high positivity rate of Piezo2 was only observed in a very limited number of vagal ganglion sections, and no quantitative statistical analysis was performed. This was our oversight and hasty conclusion. To determine the positivity rate of Piezo2 in vagal ganglion neurons marked for retrograde labeling, we have re-performed quantitative statistical analysis (calculating the proportion of mCherry and Piezo2 co-labeled neurons among retrogradely labeled mCherry neurons). The results showed that approximately 59.75% of retrogradely labeled vagal ganglion neurons were Piezo2-immunopositive, not 100% (Response Document Figure 20, and also see new Figure 7i right and Supplementary Figure 15g).

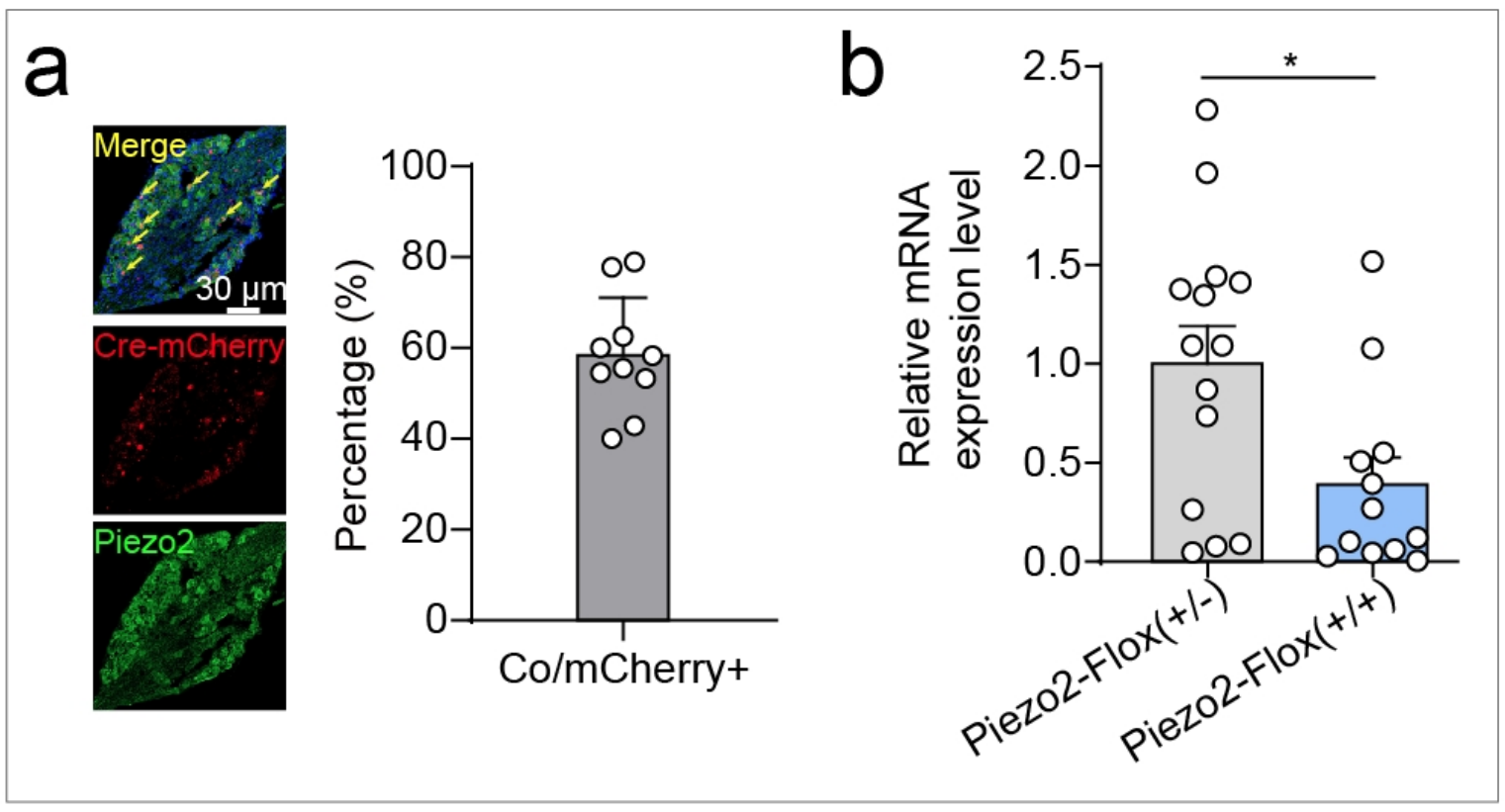

Response Document Figure 20 | Knockout efficiency of Piezo2 in neurons of the vagal ganglia that innervate the pharynx. a Proportion of mCherry and Piezo2 co-labeled neurons among retrogradely labeled mCherry+ neurons in *Piezo2-Flox*(+/-) mice. **b** Relative mRNA expression level of Piezo2 in vagal ganglion neurons projecting to the pharynx by single-cell qPCR (*Piezo2-Flox*(+/-) group: n=14 cells, *Piezo2-Flox*(+/+) group: n=12 cells).

We have revised the manuscript (Line 559-561 and 568-573), Methods section (Lines 468-489) and relevant figure legends accordingly based on the above supplementary analyses and clarifications.

7. The data in Fig. 8 are inconsistent with Prescott's 2020 Cell paper. High-intensity light alone can activate neurons. A no-Cre control group is required.

Response:

Regarding the discrepancies between the result of the present study and those of Prescott (2020): factors including the genetic background of the mice used, experimental parameters (light stimulation intensity, duration), as well as anesthesia protocols and depth of anesthesia, can all affect the induction of swallowing behavior.

A detailed analysis of the differences between the two studies is as follows:

(1) The experimental animals used are not entirely identical. Prescott (2020) utilized *Piezo2-ires-Cre;loxP-ChR2* mice, whereas our study adopted *Piezo2-Cre;Ai32* mice. Although both *Piezo2-Cre;loxP-ChR2* and *Piezo2-Cre;Ai32* mice are functionally similar in that Cre-mediated recombination drives ChR2 expression specifically in Piezo2-expressing cells, they differ in their specific genetic constructs, ChR2 expression levels, promoter regulation, and/or other genetic background details.

Specifically, *Piezo2-Cre;loxP-ChR2* mice are generated by flanking the ChR2 gene with loxP sites via genetic engineering. Cre recombinase recognizes these loxP sites, inserting the ChR2 gene into the genome of Piezo2-expressing cells through a LoxP-STOP-LoxP cassette. In contrast, the *Ai32* mouse is a specific, standardized transgenic strain harboring a loxP-ChR2 construct, generated by The Jackson Laboratory (JAX Stock No. 012569). It is specifically designed for conditional ChR2 expression. Crossing *Ai32* mice with *Piezo2-Cre* mice enables optogenetic control in specific cell types.

The "*LoxP-ChR2* mice" category may encompass various lines with differences in details such as the insertion site, ChR2 variant, or fluorescent tag. Their key components typically include a LoxP-STOP-LoxP cassette, the ChR2 (H134R) variant, and a reporter gene (e.g., EYFP). The *Ai32* strain, however, is highly standardized with a well-defined genetic background (*C57BL/6J*). Its key features include: precise insertion of the loxP-ChR2 construct into the Rosa26 locus, a strong CAG promoter driving high-level ChR2 expression post-Cre activation, a LoxP-STOP-LoxP cassette, and a ChR2 (H134R)-EYFP fusion protein. JAX provides comprehensive genotyping protocols, usage guidelines, and published data, ensuring high reliability.

In summary, the *Piezo2-Cre;Ai32* strain may be optimized for ChR2 expression efficiency,

stability, or compatibility with other genetic modifications. Therefore, these should not be considered identical mouse strains.

(2) The optical activation parameters are not identical. Prescott (2020) used the following parameters for SLN activation: 473 nm, 20 Hz, 5 ms pulses. We employed stimulation at higher frequencies and with different pulse widths (473 nm, 20, 40, 50 Hz, 10 ms pulses). Additionally, illumination of the SLN in *Piezo2-Cre/Ai32* mice with 473 nm, 20 Hz, 5 ms pulses failed to successfully induce swallowing behavior.

An analysis of these discrepancies has been incorporated into the revised manuscript (Line 715-754) of the present study.

We fully agree that rigorous optogenetic control experiments must rule out potential nonspecific effects caused by high-intensity light stimulation itself. Following your suggestion, we have supplemented a crucial "no-Cre" control experiment.

Specifically, we performed identical experiments on mice with the *Piezo2-Cre*(−/−); *Ai32*(+/+) genotype: the superior laryngeal nerve (SLN) was exposed and stimulated with the same optical parameters (473 nm, 50 Hz, 10 ms pulses, 20 mW). The results showed that optogenetic stimulation failed to evoke swallowing behavior in this control group. This negative result has been included in the revised manuscript (Response Document Figure 21, and also see new Figure 8f and 8j) (Line 625-626), demonstrating that the swallowing behavior observed in our original figures is mediated by ChR2 expression specific to Piezo2-Cre neurons and not by nonspecific effects of the light stimulus itself.

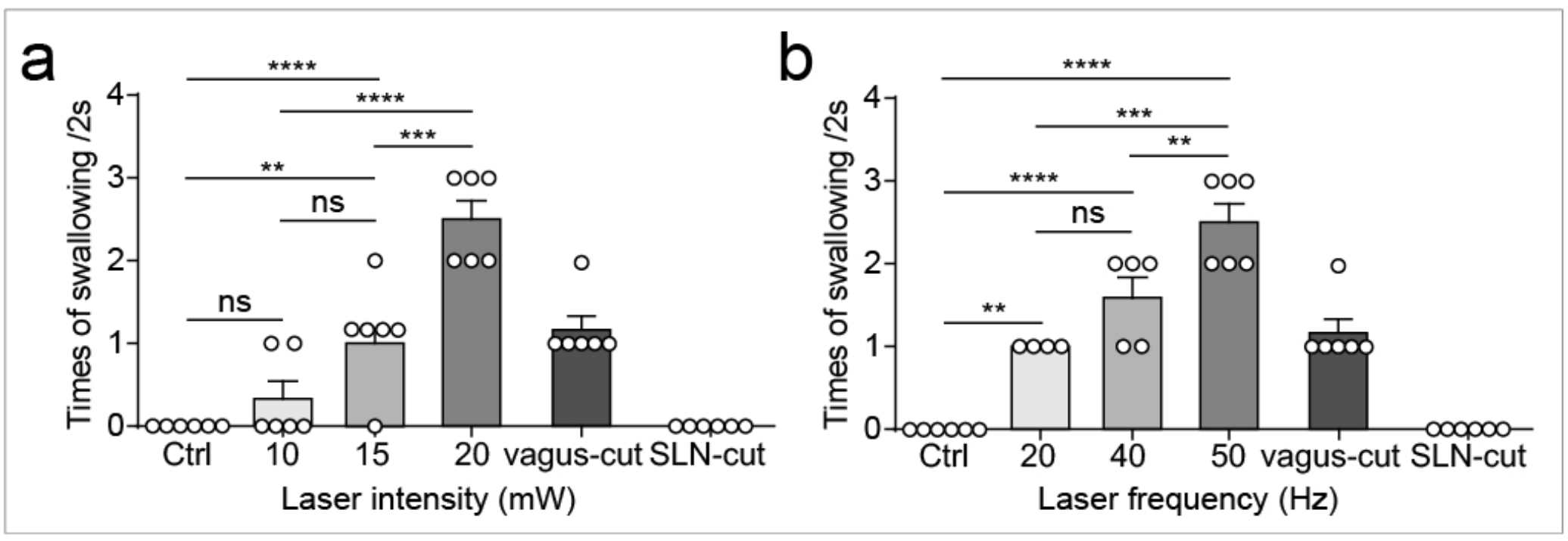

Response Document Figure 21 | Piezo2+ nerve tracts were the pathways involved in swallowing. a-b Swallowing numbers via illuminating SLN (**a**: 473 nm, 10 ms, 50 Hz, 10, 15

and 20 mW; **b** 473 nm, 10 ms, 20 mW, 20, 40 and 50 Hz) within 2 s in *Piezo2-Cre/Ai32* mice (n=6 mice). Among them, the control group was *Piezo2-Cre(-/-)/Ai32(+/+)* mice subjected to light stimulation with equivalent parameters.

8. The overall manuscript flow would benefit from revision. Several sections appear to have been added later and are not fully integrated into the earlier narrative.

Response:

Thank you for your valuable feedback regarding the overall structure and logical coherence of the manuscript. We fully agree with the points you raised and have undertaken a systematic reorganization and refinement of the entire text in the revised version. Specifically, we have:

(1) Reordered the presentation of certain results to better align with the natural scientific narrative (primarily Fig. 7 and Fig. 8, and Supplementary Fig. 13-15)—progressing from "phenomenon observation" to "mechanism verification" and then to "analysis of upstream inputs";

(2) Added transitional sentences between sections to clearly articulate the purpose of each experimental design and the specific scientific questions it addresses;

(3) More carefully integrated the subsequently supplemented experimental data, ensuring it connects organically with the earlier parts of the study.

Once again, we appreciate for your highly insightful comments, which have strengthened the rigor of the manuscript.

Response to Reviews

Manuscript ID: NCOMMS-25-80988-T

Title: A Pharynx-to-brain Sensory Pathway Mediates Swallowing-like Behavior Evoked by Mechanical Stimulation

We sincerely appreciate the positive and helpful evaluations provided by the reviewers. Guided by these thoughtful comments, we have completed supplementary experiments and analyses to address specific concerns. We have also made detailed targeted revisions to the manuscript and incorporated content related to each reviewer's comments. A revised version of the manuscript with tracked changes (highlighted in red) has been uploaded as a separate file. The detailed changes and our point-by-point responses to each of the Reviewers' questions and comments are presented below.

Contents:

Response to Reviewer #1: Page 02-10

Response to Reviewer #2: Page 11

Response to Reviewer #3: Page 11-15

Reviewer #1:

Regarding nomenclature:

• Comments regarding the ventral swallowing group being the Amb/RVLM are inaccurate. An example of this is below in quotations. The nucleus ambiguus (Amb) is a collection of motor neurons that project through the IX, X, and XI cranial nerves. The RVLM is connected to sympathetic activity and cardiovascular control. The ventral swallowing group (VSG) is located dorsal to the nucleus ambiguus or even medial to it in the reticular formation. This is also how the VSG is described in the first citation of this manuscript.

While the functional data, like ChR2 stimulation of axons innervating the nucleus ambiguus in Fig. 5d shows the NTS Th+ neurons innervating this anatomical location are sufficient to induce a swallow, the role of the RVLM is unclear. One possibility is that these axons are in the brainstem breathing control circuit (the Bötzinger and preBötzinger Complex) and serve to coordinate breathing with swallow.

"The Amb/RVLM complex, located in the VSG of the brainstem, is responsible for transmitting sensory information from the DSG to the next level of motor neurons, thereby coordinating the initiation of swallowing."

Response:

We would like to thank you for pointing out the issue regarding terminology accuracy. Your correction is entirely correct. We fully agree with the reviewer's perspective and have made the corresponding revisions.

(1) Clarification of the classic anatomical localization of the VSG: Classic literature such as Jean (1984) and Jean (2001) clearly defines the ventral group of the brainstem swallowing network (VSG) as located within the dorsal reticular formation of the nucleus ambiguus (Amb) —that is, in the dorsal and more medial region of the brainstem reticular formation area of the Amb. Describing the VSG directly as the "Amb/RVLM complex" is indeed inaccurate. The RVLM, by classic definition, is primarily responsible for cardiovascular sympathetic regulation and does not have complete anatomical overlap with the swallowing center. We

have corrected this in the revised manuscript (Lines 354-358).

(2) The true anatomical localization of the optogenetic stimulation target in this study: Our experimental evidence (Fig. 5d) confirms that the axon terminals of TH+ NTS neurons project clearly to the Amb region, which is the final common pathway for swallowing output. In this study, the optical fiber indeed targeted this ventrolateral area, located at the intersection of the VSG core (dorsal reticular formation of the Amb) and the region innervated by Amb motor neurons. For simplicity, we used "Amb/RVLM" as an abbreviation for this ventrolateral key swallowing region.

(3) Potential physiological significance of RVLM in this pathway: As the reviewer noted, the core function of RVLM is cardiovascular sympathetic regulation. A large body of research also indicates that interneurons within the RVLM region modulate the coordination of swallowing and respiration (e.g., Bötzinger complex and pre-Bötzinger complex). Functionally, activation of the TH+ NTS-Amb/RVLM pathway alters swallowing frequency without significantly affecting respiratory frequency, which may suggest that the primary role of this pathway is to "drive swallowing itself". Given that our study focuses on the drive circuits for swallowing behavior rather than the mechanisms of respiratory-swallowing coordination, the functional significance of the RVLM region in this pathway—specifically its use for coordinating respiration and swallowing—as you pointed out, requires further investigation. We have added this content to the Discussion section (Lines 781-792).

"The functional coordination of swallowing and breathing

Research on mechanical stimulation induced swallowing aids in elucidating the neural basis of swallowing-respiration coordination, including the functional hypothesis that the Amb/RVLM regulates swallowing and respiration inhibition. Amb is generally represented as a small cluster of large cholinergic motor neurons which participating in essential reflexes such as swallowing, the gag reflex and heart function[3,62]*. While RVLM catecholaminergic neurons regulate breathing*[63]*. Alternatively, the axons of TH+ NTS neurons are located in the brainstem respiratory control circuit (Bötzinger complex and pre-Bötzinger complex) and are used to coordinate breathing with swallowing. We hypothesize that Chat+ Amb motor neurons*

may involve in swallowing movements, whereas RVLM catecholaminergic neurons play a role in transient respiration arrest during swallowing. Further verification will be conducted in the future."

Regarding swallowing during natural consumption:

• The concerns regarding the role of the Th+ neurons in consumptive swallowing remains. The data provided in the rebuttal is a simple bar graph with methodology described. Is there a reason that more data was not presented or included in the manuscript? At this time, the conclusion that consumptive swallowing is impacted is that the drinking bout size and duration are unchanged, but even the raw data to make these quantifications like in Fig. 3h and i cannot be found within the manuscript. It is challenging to evaluate the validity of this conclusion: "(page 12, line 271) which to some extent reflectd a dysfunction in swallowing".

The most definitive conclusion from this experiment is that despite inhibition of the NTS Th+ neurons, the mice still CAN swallow during natural consumption.

• "Furthermore, a apparent increase in GCaMP fluorescence at the onset of swallowing was observed when fixed-head mice initiated swallowing induced by slow manual water delivery (Supplementary Fig. 7b, movie 1)."

How was this concluded to be a swallow? Was the animal even consuming fluid?

Response:

Thank you very much for raising this key question, which helps us further improve our manuscript.

(1) Throughout our entire study, electromyography (EMG) recordings of the mylohyoid muscle were used as an indicator to detect swallowing behavior. This is because, in most species, the onset of the pharyngeal phase of swallowing begins with the contraction of the mylohyoid muscle, which can be considered the first muscle to become active during swallowing (Jean, 2001). A substantial body of literature indicates that mylohyoid EMG recordings represent a non-invasive and convenient assessment method for evaluating

swallowing. An EMG burst sequence is defined as a single swallowing event, and the number of swallows is determined by the count of EMG bursts (references: *Lulu Yao et al, Electroacupuncture improves swallowing function in a post-stroke dysphagia mouse model by activating the motor cortex inputs to the nucleus tractus solitarii through the parabrachial nuclei, 2023; Murakawa et al, Modulation of the swallowing reflex by stimulation of the gigantocellular reticular nucleus in the rat, 2023; Jean, Brain stem control of swallowing: Neuronal network and cellular mechanisms, 2001; Kitagawa et al, Pharyngeal branch of the glossopharyngeal nerve plays a major role in reflex swallowing from the pharynx, 2002*).

In chemogenetic inhibition experiments of TH+ NTS neurons during drinking behavior, to directly measure swallowing behavior, we monitored swallowing metrics (mylohyoid electromyography) before and after chemogenetic inhibition of TH+ NTS neurons while mice drank freely. Although presented as a single bar graph, this indicated that chemogenetic inhibition of TH+ NTS neurons reduced swallowing frequency during free drinking. Inactivation of these neurons impaired the perception of water entering the pharynx, even if it did not affect the oral phase of water propulsion into the pharynx, thereby preventing the pharyngeal phase of swallowing and leading to swallowing abnormalities.

Furthermore, our study proposes that TH+ NTS neurons are a key neuronal population involved in the swallowing reflex, but not the sole neuronal type. As you noted, despite the inhibition of TH+ NTS neurons, mice are still capable of swallowing during natural feeding. Additionally, since the oral phase was unaffected, water or food can enter the oral cavity normally; however, accumulation in the oral cavity would naturally spill out, which may also be one reason for the continued water consumption observed after TH+ NTS neuron inactivation. To more comprehensively demonstrate the role of TH+ NTS neurons in drinking-swallowing function, we have updated the mouse swallowing frequency (EMG counts) before and after chemogenetic inhibition of TH+ NTS neurons in the manuscript (see new Figure 3d right, Line 270). To strengthen the rigor of the conclusion, we have revised “this to some extent reflects swallowing dysfunction” to “suggesting a reduction in swallowing efficiency during natural drinking” (Line 271).

For the quantitative analysis of the data in Fig. 3h and 3i (see new Figure 3e and 3f), detailed

explanations are provided in the Methods file (Lines 175-187) and the Response to Reviewers on page 28, Question 9. To facilitate reading by reviewers and readers, we have updated the quantitative analysis of Fig. 3h and 3i (see new Figure 3e and 3f) in the corresponding figure legends as follows:

"Drinking behavior was recorded using a multimodal synchronous system. All licking signals were acquired in real time by Spike2 software, with simultaneous video recording (50 frames/s; Point Grey Research, Canada). During data analysis for 3, a continuous and regular cluster of signals recorded by Spike2 was defined as one drinking event, with its duration measured from the first signal to the last signal. There should be at least a 3-second interval between two independent drinking events. The number of drinking events was counted as the total number of such independent signal clusters, and the total drinking duration was calculated as the cumulative sum of the durations of all events."

(2) Regarding the assessment of water-induced swallowing in mice with fixed heads: The following is our explanation based on clinical standards and the experimental design: Clinical methods for detecting swallowing include Fiberoptic Endoscopic Evaluation of Swallowing (FEES), Videofluoroscopic Swallowing Study (VFSS), Standardized Swallowing Assessment (SSA), and Water Swallow Test (WST). However, these techniques have not been widely applied in rodent experiments. Recently, Lever's laboratory at the University of Missouri School of Medicine has developed equipment for fiberoptic endoscopy (FEES) and videofluoroscopic swallowing studies (for rodents, VFSS), but it has not yet become a routine experimental method.

In our study, we still employed swallowing physiological indicators—electromyography (EMG) of the mylohyoid muscle—to detect swallowing behavior. As shown in Movie 1, when water was administered orally, we simultaneously recorded typical EMG waveforms, using the EMG burst characteristics as the initiation of swallowing. This provides electrophysiological corroboration for behavioral observations. Additionally, we conducted control experiments: no water administration or only lip touching. The results showed that EMG bursts occurred only when actual water was administered. To enhance clarity, we revised the sentence "Additionally, a significant increase in GCaMP fluorescence was

observed at the onset of swallowing induced by slow manual water delivery in head-fixed mice (Supplementary Figure 7b, Movie 1)" to: "Additionally, a significant increase in GCaMP fluorescence was observed at the onset of water-induced swallowing, which was confirmed by the electromyogram of synchronized mylohyoid muscle activity (Supplementary Figure 7b, Movie 1)" (Lines 332-335).

Thank you again for your careful review.

Regarding Fiberphotometry conclusions:

• Supplemental Fig. 7 shows that the Th+ neurons have a Fiberphotometry signal during a swallow that is induced by slow perfusion of citric acid, salt, or water into the pharyx / larynx. Unlike the model of Piezo2 fibers exciting the Th neurons when the pharynx is mechanically stimulated, this data indicates that the Th+ neurons are activity that is recruited by the action of a swallow. While this does not mean that cannot be important for swallowing, it is challenging to understand how they become activated by CA, high salt, and water as proposed.

Once again, the Fiberphotometry signals always occur after the dashed line (swallow onset) as opposed to preceding them.

Response:

Thank you for raising this critical temporal question. We fully understand your concern: if the GCaMP signals of TH+ NTS neurons activated by citric acid, high salt, and water consistently occur after the onset of swallowing (EMG burst initiation) rather than before, then the activity of these neurons is more likely a consequence of swallowing rather than its cause. When analyzing the calcium activity of TH+ NTS neurons activated by stimulation of the pharynx with citric acid, high salt, and water, we initially used visually observed pharyngeal elevation as the start of swallowing, which introduced some error. We re-analyzed the data using EMG burst characteristics as the marker for swallowing onset, performing frame-by-frame analysis. The results showed that swallowing initiation occurs when the fiber photometry signal

reaches 18% of the GCaMP peak, suggesting that TH+ NTS neuron activity precedes swallowing (consistent with relevant reference indicating that neural activity precedes behavior: *Shinya Fuse et al, Spatio-temporal segregation between sensory relay and swallowing pre-motor population activities by optical imaging in the rat nucleus of the solitary tract, 2025*). Furthermore, the entire process from activation of TH+ NTS neurons by chemical stimulation to the end of swallowing was extremely brief, approximately 0.4 seconds. Therefore, the fiber photometry signal indicating activation of TH+ NTS neurons and the swallowing onset signal are highly temporally proximal. We have added an accurate description of the signal timing in the legend of Supplementary Figure 7c: "GCaMP signals showed a detectable increase before the swallowing event and remained elevated during subsequent swallowing." (see legend of Supplementary Figure 7). For supplementary Figure 7a, the dashed line indicates the time point of mechanical stimulation of the pharyngeal mucosa by Von Frey filaments, not the onset of swallowing. There is a latency period from the onset of mechanical stimulation to the initiation of the swallowing reflex (similar to optogenetic activation of TH+ NTS neurons inducing swallowing). This is also clearly stated in the figure legend. To make it clearer and more convenient for reading, we have also marked it in the figure.

Once again, thank the reviewers for their insightful comments, which have significantly enhanced the rigor of the analysis and the conservatism of the conclusions.

Regarding Piezo2 channel Rhodopsin:

• The additional discussion section is incorrect regarding the transgenes used in Prescott 2020. The methods of the paper clearly state that the transgenic lines are Piezo2-EGfp-ires-Cre (027719) and loxP-ChR2 (012569). This loxP-ChR2 line is Ai32. I do not understand the explanation provided in the discussion. This should be removed.

Response:

Thank you for pointing out the incorrect description in our Discussion section. We have carefully verified and confirmed this issue. We fully agree with the reviewer's judgment. After

a thorough review of the original literature, we confirm that the transgenic mouse strain used by Prescott 2020 in their Cell paper is *loxP-ChR2* (JAX Stock No. 012569), which is identical to the *Ai32* (JAX Stock No. 012569) that we employed. We acknowledge that referring to loxP-ChR2 as an independent strain rather than Ai32 in the original Discussion could be misleading.

Following your suggestion, we have deleted the paragraph containing the lengthy explanation of strain differences related to the Prescott 2020 study from the Discussion section (original manuscript lines 724-748).

Regarding GtACR1 terminal inhibition:

• This channel has been shown in multiple papers to elicit synaptic release when light is provided to the axon terminals. Other optogenetic tools such as NpHR or OPN3 or PPO are the accepted methodologies for terminal inhibition. While the data provided is consistent with the hm4di results, the lack of validation of this method for terminal inhibition weakens the meaning of this data. It is hard to imagine that this ventral brainstem region is the premotor source of swallowing.

Response:

Sincerely appreciate your professional and significant technical suggestions, which are crucial for verifying the necessity of the TH+ NTS-Amb/RVLM pathway for swallowing function.

(1) Regarding the validation of optogenetic terminal inhibition methods, you are entirely correct. While GtACR1-mediated optogenetic silencing has been widely employed for neuronal inactivation, studies have indicated that direct illumination of axon terminals may induce off-target effects by inhibiting passing fibers, thereby limiting the interpretation of specific projection functions. Consequently, there is insufficient fine-grained spatially specific inhibition validation of the TH+ NTS to Amb/RVLM projection using GtACR1-based optogenetic tools, which weakens the data strength supporting the necessity of this projection at the circuit level.

(2) In accordance with your suggestion, we further utilized the NpHR optogenetic silencing tool to manipulate swallowing experiments in mice. To achieve this, AAV-DIO-eNpHR-EYFP (test) or AAV-DIO-EYFP (ctrl) was injected into the NTS of *TH*-ires-cre mice, and optical fibers were implanted above the bilateral Amb/RVLM. Three days of adaptive drinking training was conducted prior to formal testing. All animals first drank water for 30 minutes under non-light stimulation conditions. On the following day, 589 nm blue light was delivered continuously to silence the TH+ NTS-Amb/RVLM pathway when mice initiated drinking. The optogenetic silencing experiment demonstrated that optogenetic inhibition of TH+ NTS terminals within the Amb/RVLM nucleus significantly reduced water intake (Response Document Figure 1, and also see new Supplementary Figure 11 d-f). This result further confirms the necessity of the TH+ NTS-RVLM pathway for swallowing function.

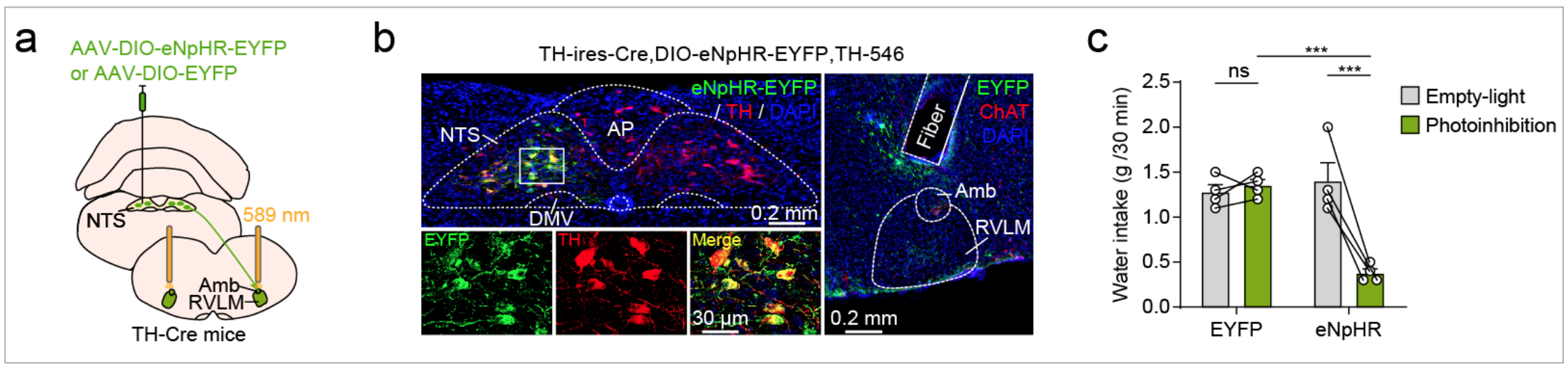

Response Document Figure 1 | Optogenetic inhibition of the TH+ NTS-Amb/RVLM pathway reduced water intake in mice. a Schematic illustrating the injection sites of AAV-DIO-eNpHR-EYFP (experimental group) or AAV-DIO-EYFP (control group) in the NTS of *TH*-ires-Cre mice, and the fiber implantation sites in the Amb/RVLM region. b Representative microphotographs showing eNpHR-EYFP expression in the NTS, the fiber tract above the Amb/RVLM axon terminals, and colocalization of eNpHR-EYFP-positive (green) and TH-positive neurons (red). **c** Water intake in mice following optogenetic inhibition of TH+ NTS neuron terminals in the RVLM (n = 4 mice). Data are presented as mean ± standard error of the mean (error bars). The dashed white lines in the brain slice images represent brain region boundaries. Statistical analysis for c was performed using two-way ANOVA (***P < 0.001, ns > 0.05).

We have supplemented these new data in the Methods section (Line 191-196) and the revised manuscript (Lines 449-456).

Reviewer #2:

The authors have addressed my comments.

We are pleased that our revisions have addressed your concerns and that the manuscript is now clearer. Your insightful comments and constructive suggestions throughout the review process have been extremely helpful in strengthening our study. We thank you again for your time and expertise in reviewing our paper.

Reviewer #3:

The authors performed a large number of new experiments and reasonably addressed most of my concerns. Although there is still a novelty concern that the current manuscript seems a combination of Want et al 2024 and Prescott et al 2020, the data provided is though and solid, and could complement the field. I just have a few minor comments left.

We sincerely appreciate the positive evaluation and recognition of our research work. The advisory comments you provided during the first round of review significantly contributed to improving the manuscript. Below, I will respond point by point.

First, in line 88, This is not from Prescott team - Prescott's work in the Liberles lab.

Response:

We have changed “Prescott team” in the original manuscript to “Liberles Laboratory” (Line 88).

Second, the first paragraph in line 246, this is not necessity. It should be moved to the last section.

Response:

The first paragraph of line 246, which describes chemogenetic activation of TH+ NTS neurons immediately following the results of optogenetic activation of TH+ NTS neurons to further validate their sufficiency for swallowing, has been moved to the last paragraph of Section 2 in the Results section to enhance logical coherence and tightness (see new Figure 2 k-m, Lines 243-256).

Third, in line 308, it should be mentioned that this is 8/17 recorded neurons.

Response:

We have clearly indicated “n = 8/17 neurons” in the revised manuscript (Line 309).

Fourth, in the paragraph starts from line 342, functional divergence was tested between Th-LPB and Th-NA pathways. However, it is not mentioned whether they are the same neurons or different neurons. Vice versa, the authors mentioned that Th-NA and Th-Arc are different neurons, yet whether the Th-Arc pathway also contributes to swallowing has not been tested.

Response:

Thank you for your suggestions to improve the TH+ NTS neuron downstream pathway. Based on your recommendations, we have supplemented the corresponding validation experiments.

(1) To test whether the TH+ NTS-Arc pathway is involved in swallowing, we performed optogenetic experiments. AAV-DIO-ChR2-mCherry was injected into the NTS of *TH*-ires-Cre mice, and an optical fiber was implanted above Arc. We observed that optogenetic activation of TH+ NTS-Arc (473 nm, 20 Hz, 10 ms, 2, 4, and 8 mW) failed to induce swallowing behavior (Response Document Figure 2, and also see new Supplementary Figure 9 a–b).

(2) To mark the distribution of TH+ neurons projecting to the LPB and Amb/RVLM within the NTS, we employed a specific retrograde tracing strategy. AAV2/2Retro-DIO-mCherry and AAV2/2Retro-DIO-EGFP were respectively injected into the LPB and Amb/RVLM of

TH-ires-Cre mice. After 3 weeks of AAV expression, co-immunostaining and quantitative analysis of mCherry and EGFP in NTS brain slices revealed that 22% of TH+ NTS neurons projecting to the LPB and Amb/RVLM were double-labeled (Response Document Figure 2; also see new Supplementary Figure 9f–h).

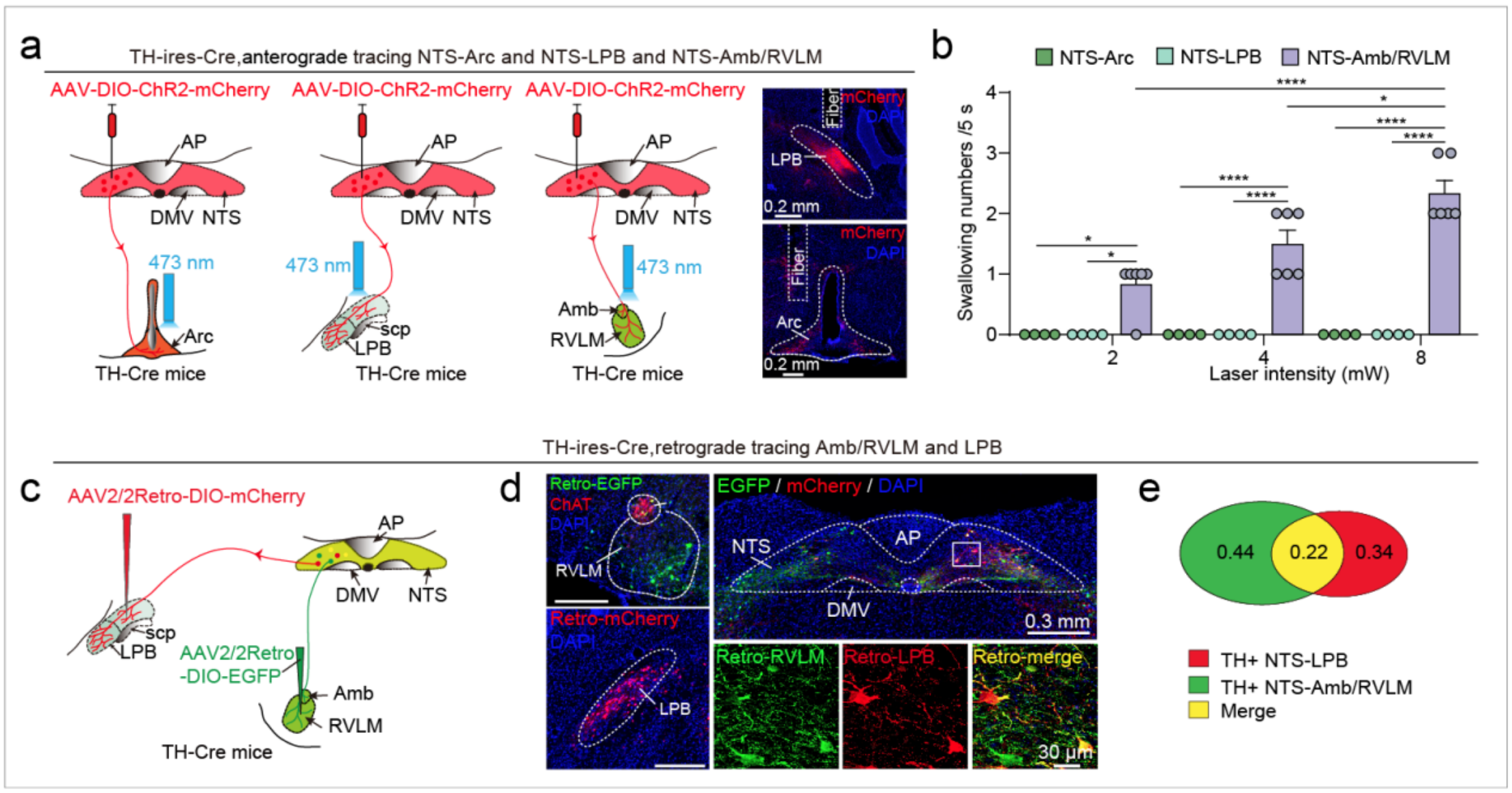

Response Document Figure 2 | Comparison of the TH+ NTS-Amb/RVLM pathway with the TH+ NTS-Arc pathway or the TH+ NTS-LPB pathway. a Schematic diagram of optogenetic strategy of activating the axonal terminals of NTS projecting to Arc or LPB or Amb/RVLM in different *TH*-ires-Cre mice (left). Example micrographs displaying the optical fiber track above the axon terminal in the LPB and Arc (right). **b** Quantitative analysis of swallowing numbers of *TH*-ires-Cre mice with optogenetic activation (473 nm, 10 ms, 20 Hz, 2, 4 and 8 mW) of the NTS-Arc (n=4) or NTS-LPB (n=4) or NTS-Amb/RVLM (n=6) pathway severally. **c–e** Schematic diagram of viral injection strategy to LPB-projecting and Amb/RVLM-projecting TH+ NTS neurons in the same mouse (c). Example coronal brain section and the magnified fields showing the distribution of Amb/RVLM-projecting (EGFP+) and LPB-projecting (mCherry+) TH+ neurons in the NTS (d). The scale is 0.3 mm in g left. The fraction of EGFP-labeled (green), mCherry-labeled (red) and dually labeled (yellow) neurons in the NTS (e). Data were shown as mean ± s.e.m. (error bars). The broken white lines in the section images represent boundaries of brain regions. Statistical analyses in b was performed using Two-way ANOVA (*P < 0.05, ****P < 0.0001).

Fifth, the newly added experiment described in line 411 should be moved to the next section, as it describes the necessity.

Response:

As per your suggestion, we have moved the optogenetic silencing experiment to verify the necessity of the TH+ NTS-RVLM pathway to the next section (see new Supplementary Figure 11) (Lines 441-456).

Sixth, in line 524, it is still not clear what this single cell data shows. The authors should show the integrated UMAP plot of these neurons and all NTS sequenced, and show representative gene expressions including Piezo2, Trpv1, and etc to guide the readers about the characteristics of the relevant nodose neuron types.

Response:

Thank you for raising this critical question. We fully concur that demonstrating the expression of representative genes (such as Piezo2, etc.) is important for understanding the neuronal subtypes within the vagal ganglia. In this section, we employed the rabies virus (RV) vector tracing method to identify upstream neurons of TH+ NTS neurons, primarily the vagal ganglia. RV-helper AAVs was injected into the NTS of *TH*-ires-Cre mice, followed by RV-DsRed was injected two weeks later. We harvested RV-traced vagal ganglion neurons and performed SMART-seq2 sequencing. We used our previously established vagal ganglion neuronal single-cell database (PMID: 38288368). Sequencing data was deposited in the Genome Sequence Archive (GSA) database (GSA accession number: CRA007790). For clustering analysis, we performed clustering based on 9,000 highly variable feature genes with the FindVariableFeatures() function in Seurat. We then calculated the signature score between all the 31 clusters of vagus sensory neurons and RV-traced neurons according to the signature gene expression similarity. The RV-traced neurons were similar to those in clusters 2, 4, 14, 18, 23 and 31, which contain receptors mediating swallowing induced by chemical,

mechanical, and thermal stimulation.

Following your suggestion, we analyzed the expression of genes including Piezo2, Trpa1, Trpv4, Trpm3 in the vagal ganglia and presented their tSNE plots, a dimensionality reduction visualization tool similar to UMAP (Response Document Figure 3, and also see new Supplementary Figure 15f–g). The TRPV1 you mentioned is the receptor for capsaicin, which was not involved in our research.

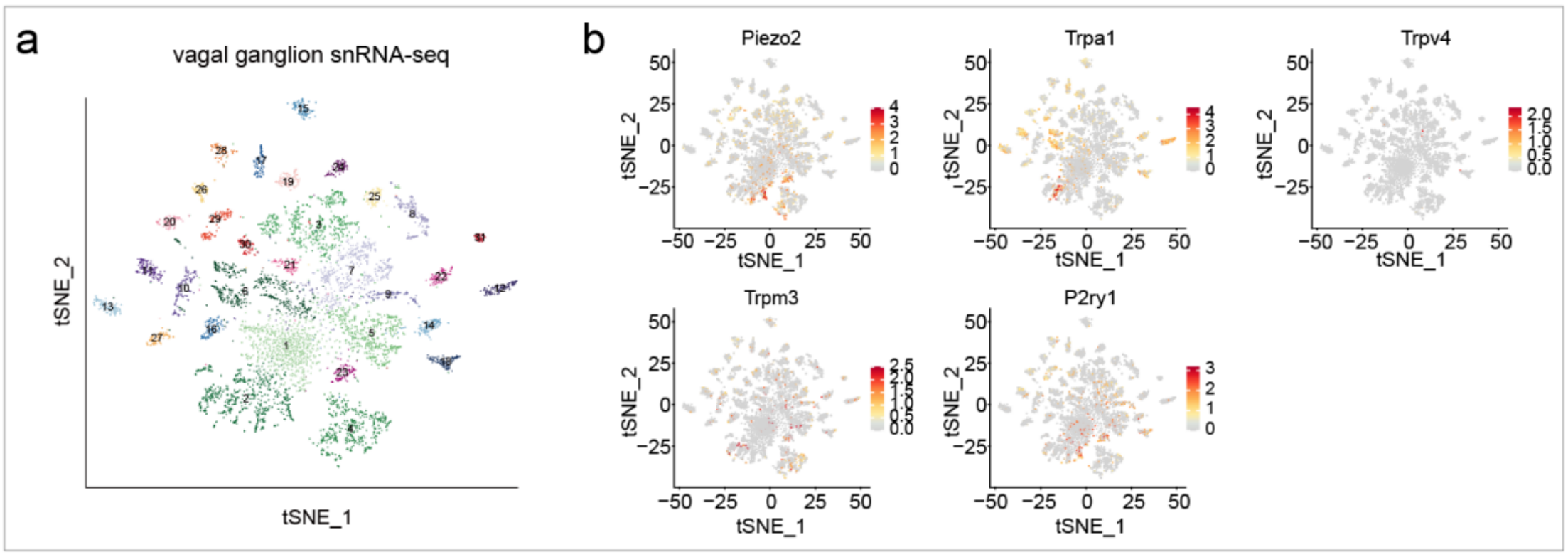

Response Document Figure 3 | tSNE plot of the feature gene expression of vagal ganglion neurons tracked by RV. a t-SNE visualization of the vagal ganglion neuronal clusters. **b** t-SNE plot of characteristic gene expression in the vagus ganglion.

We have supplemented the analytical method in the Method Section (Lines 467-473).

Once again, thank you for your professional advice and careful review, which has significantly improved the understandability and persuasiveness of the data.

Response to Reviews

Manuscript ID: NCOMMS-25-80988-T

Title: A Pharynx-to-brain Sensory Pathway Mediates Swallowing-like Behavior Evoked by Mechanical Stimulation

We sincerely appreciate the positive and helpful evaluations provided by the reviewers. Guided by these detailed recommendations, we have completed our analysis to address specific concerns. Additionally, we have incorporated content addressing the feedback from the third reviewer. The detailed changes and our point-by-point responses to each of the Reviewers' questions and comments are presented below.

Contents:

Response to Reviewer #1: Page 2

Response to Reviewer #3: Page 2-7

Reviewer #1:

I have no further comments to address.

Response:

We would like to once again extend our heartfelt gratitude for the valuable time you have devoted to this manuscript and for your professional guidance. Every constructive suggestion you have provided has significantly enhanced the scientific rigor and the quality of the arguments presented in this study; we are deeply grateful for these contributions. We sincerely thank you once again.

Reviewer #3:

The authors have additional experiments and have satisfactorily addressed most of my previous concerns. Although I continue to believe that the conceptual novelty is primarily in the integration of previously established findings rather than the discovery of an entirely new principle, the study now provides a comprehensive and technically rigorous characterization of a neural circuit mediating mechanically evoked swallowing, which will be a valuable contribution to the field.

We thank you for your recognition of our supplementary experiments and revisions, and we sincerely appreciate your candid assessment of the value and limitations of this study.

We fully acknowledge your observation—that this study's contribution primarily focuses on the systematic functional validation and mechanistic integration of established neural anatomical pathways, rather than discovering a novel biological principle. We concur with this assessment. Therefore, our conclusion focuses on "conducting a systematic analysis of the pharyngeal-brain mechanical sensory conduction pathway across the molecular to circuit level, and providing functional validation and deeper mechanistic insights based on the existing anatomical framework" rather than emphasizing "a principled breakthrough".

I have one remaining comment mainly concern the interpretation of the single-cell analysis results. In the TSNE plots shown in Supplemental Figure 15, there are numerous very small

clusters. Very likely, many of them are not neuronal populations. However, it is difficult to know which clusters really represent nodose neurons or jugular neurons. The authors should show Phox2b expression (nodose), Prdm12 (jugular marker), Slc17a6 (pan), Snap25, Tubb3, Trpv1, Cckar, Gpr65, Uts2b, and other markers used in Kupari, Prescott, Bai, Zhao, and other papers to guide the readers about vagal sensory neuron subtypes. I strongly recommend to replace TSNE plot with UMAP plot, which is the current standard way for single-cell data visualization. I also suggest to subset only neuronal populations for better visualization. Currently, it is difficult to identify cluster 2, 4, 14, 18, and 23, which are enriched clusters from labeled cells, from panel f. The whole idea of this figure is to identify the molecular signatures of retrogradely labeled vagal neurons and current figures cannot serve the role.

Response:

We are very grateful for your professional feedback on the single-cell data analysis section. We fully agree with your views and have made systematic improvements to the relevant analyses in Supplementary Figure 15 based on your suggestions.

1. Regarding an alternative to t-SNE plots

Your suggestion to use UMAP as an alternative to t-SNE is very insightful. Compared to t-SNE, UMAP better preserves global structure and is generally more stable when processing large-scale single-cell datasets. However, t-SNE is also a widely used dimensionality reduction technique for clustering and can similarly be employed to perform clear cell clustering and subpopulation identification (reference: *Genetic identification of vagal sensory neurons that control feeding, Cell; An atlas of vagal sensory neurons and their molecular specialization, Cell Reports; A spatially-resolved transcriptional atlas of the murine dorsal pons at single-cell resolution, Nature Communications; Decoding molecular and cellular heterogeneity of mouse nucleus accumbens, Nature Neuroscience*). Here, to present the single-cell analysis results more clearly and comprehensively, we have combined t-SNE and UMAP to mutually validate the results.

2. Regarding the partitioning that displays only a subset of neuronal groups

Your suggestion for subsampling the neuronal clusters is highly reasonable. When submitting

the manuscript, the clusters shown in the t-SNE plot (see Response Document Figure 1a) were delineated based on Phox2b+ sensory neurons as markers. To further clarify the neuronal identity of these cell populations, we added the following representative marker genes—Snap25, Tubb3, Phox2b, Slc17a6, Trpv1, Cckar, Gpr65, Uts2b, Prdm12—to the existing t-SNE plot (see Response Document Figure 1), to enhance biological interpretability. Therefore, in the original t-SNE plot, we confirm that only neuronal populations are retained. Additionally, we have presented a UMAP visualization of the subsampling of neuronal clusters (see Response Document Figure 2).

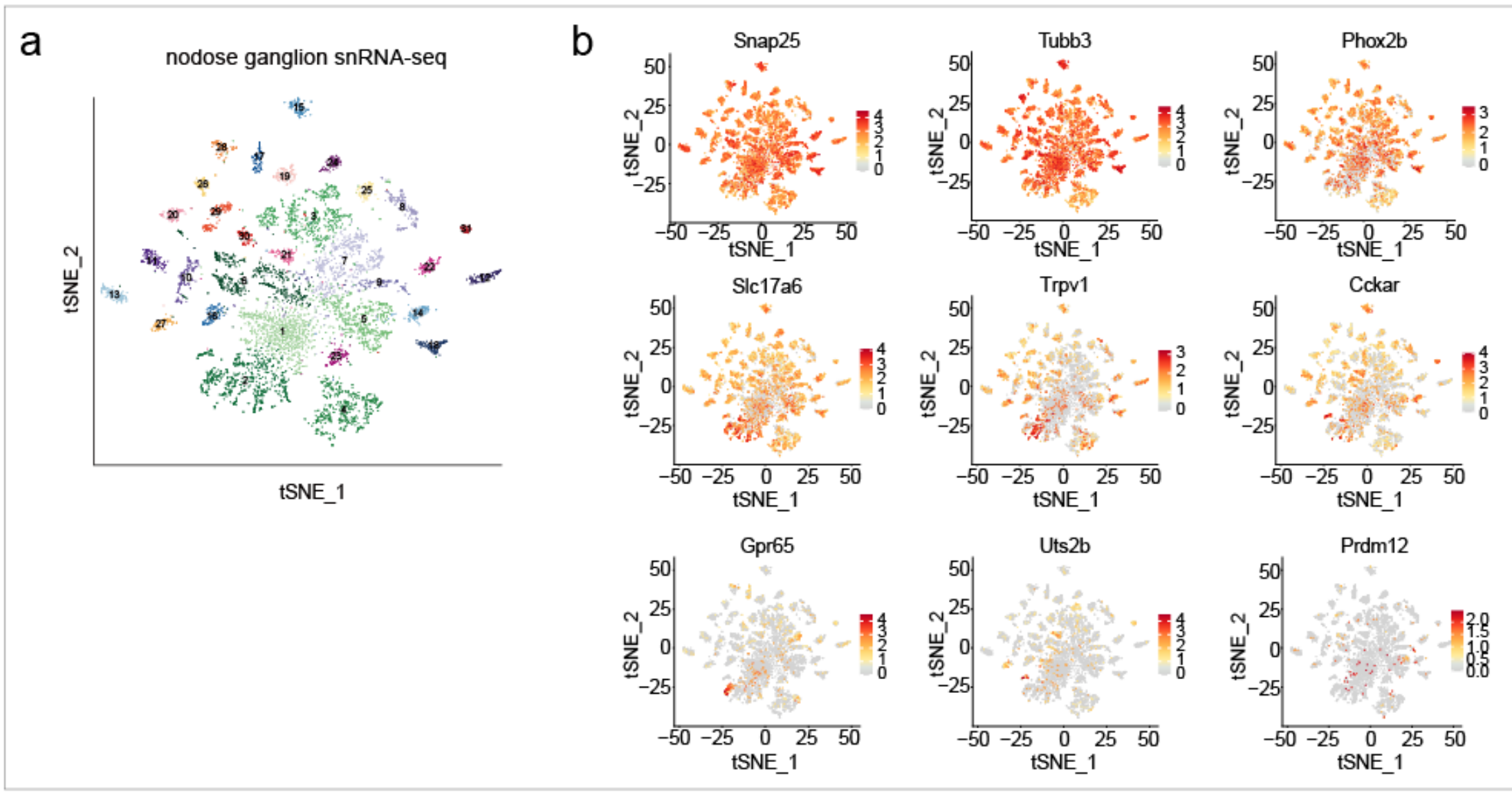

Response Document Figure 1 | t-SNE plot of the feature gene expression of nodose ganglion neurons. a t-SNE visualization showing the clustering pattern of nodose ganglion neurons. b t-SNE plot showing the expression of characteristic genes in nodose ganglion neurons.

3. Regarding the annotation of key marker genes

During our re-analysis of the data, we referred to key biomarkers used in classical literature within this field. Following your recommendation, we specifically annotated the following representative marker genes: Phox2b, Prdm12, Slc17a6, Snap25, Tubb3, Trpv1, Cckar, Gpr65,

and Uts2b, and presented their expression profiles as a UMAP plot (see Response Document Figure 2 and Supplementary Figure15 e–f). The results also show that all clusters identified through our analysis correspond to neuronal populations.

Studies have reported that the vagal ganglion complex consists of approximately 85% nodose neurons and 15% carotid jugular neurons. The nodose ganglion serves as the primary conduit for the physiological sensory signals involved in swallowing, with numerous swallowing-related mechanoreceptors and chemoreceptors being highly expressed within its neurons. In contrast, the jugular ganglion is primarily involved in nociceptive sensation (reference: *An atlas of vagal sensory neurons and their molecular specialization; Brainstem viscerotopic organization of afferents and efferents involved in the control of swallowing; Anatomical and functional mapping of vagal nociceptive sensory nerve subsets innervating the mouse lower airways by intersectional genetics*). Therefore, in single-cell analysis, we focus specifically on the nodose ganglion; this is also the reason why Prdm12 expression levels are relatively low in the UMAP plot.

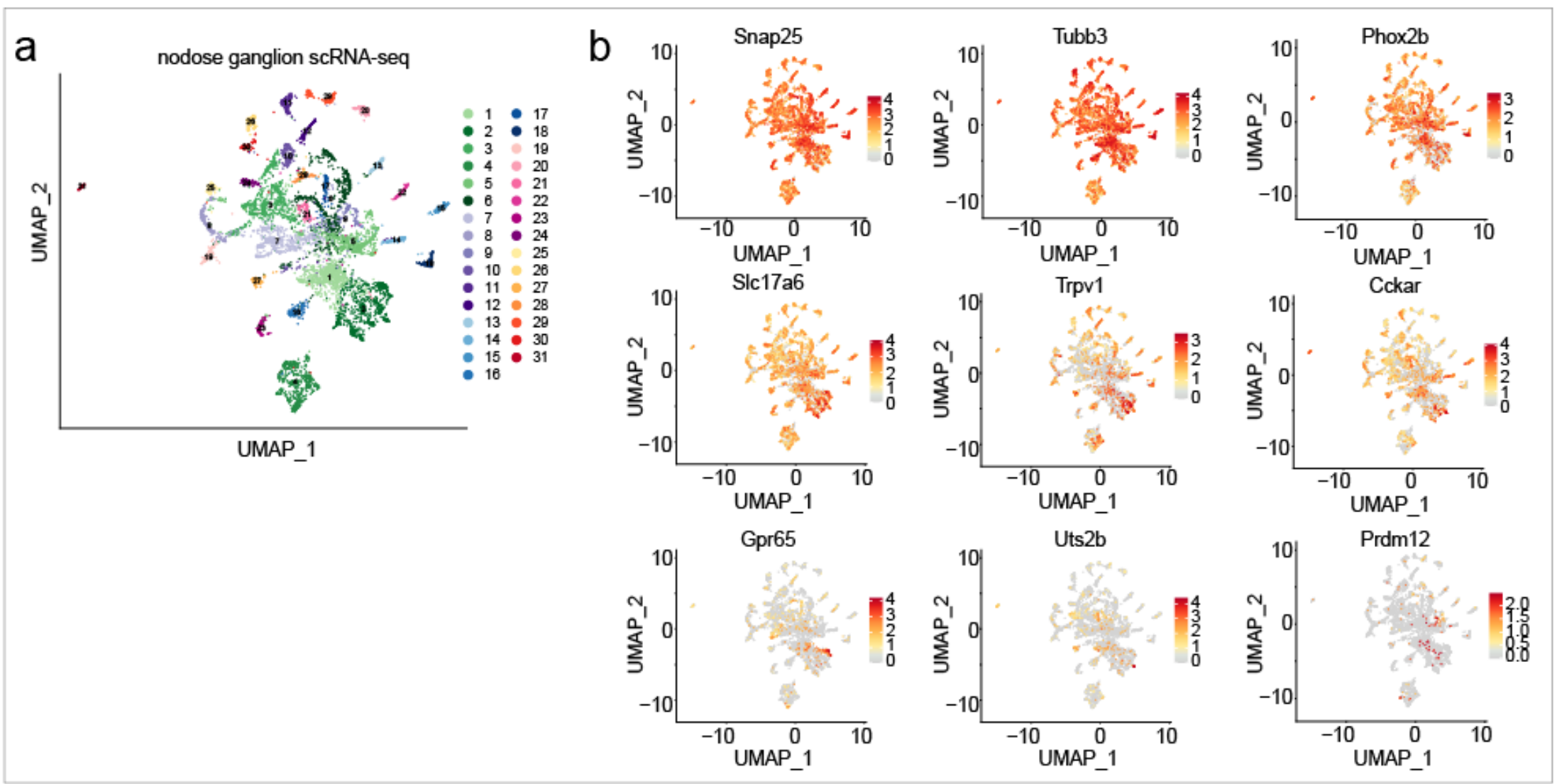

Response Document Figure 2 | UMAP plot of the feature gene expression of nodose ganglion neurons. a UMAP plot showing the clustering pattern of nodose ganglion neurons. b UMAP plot displaying the expression profiles of characteristic genes from nodose ganglion neurons.

Since the nodose and jugular ganglia in mice are located very close to each other, the nodose tissue we collected may inevitably contain neurons from the jugular structure; therefore, we have adopted the term “vagal ganglion”. To avoid any misunderstanding, we explicitly state this in the figure caption; This figure illustrates the clustering results of nodose ganglion neurons and a visualization of their characteristic gene expression.

4. Identification of clusters 2, 4, 14,18, and 23

To identify clusters enriched in RV retrograde-labeled cells, we performed a transcriptomic similarity analysis comparing DsRed-labeled nodose ganglion neurons with nodose neuron clusters, as well as a dot plot of these feature scores, obtained via t-SNE analysis (see Response Document Figure 3a and Supplementary Figure 15 g top). As shown in the figure, RV retrograde labeling cells are enriched in clusters 2, 4, 14, 18, 23 and 31. Additionally, we analyzed the expression levels of Piezo2, P2ry1, Trpm3, Trpv4, and Trpa1 across the various clusters (see Response Document Figure 3b and Supplementary Figure 15 g bottom). Our results demonstrate that Piezo2 and P2ry1 are highly expressed in Cluster 23, suggesting that the receptors mediating mechanical and chemical stimuli that induce swallowing may be distributed within Cluster 23. In contrast, the expression levels of the remaining sensory-related receptors were lower in clusters 2, 4, 14,18,23, and 31, which is consistent with our behavioral findings. In the future, we will further investigate the molecular mechanisms underlying these clusters in relation to swallowing.

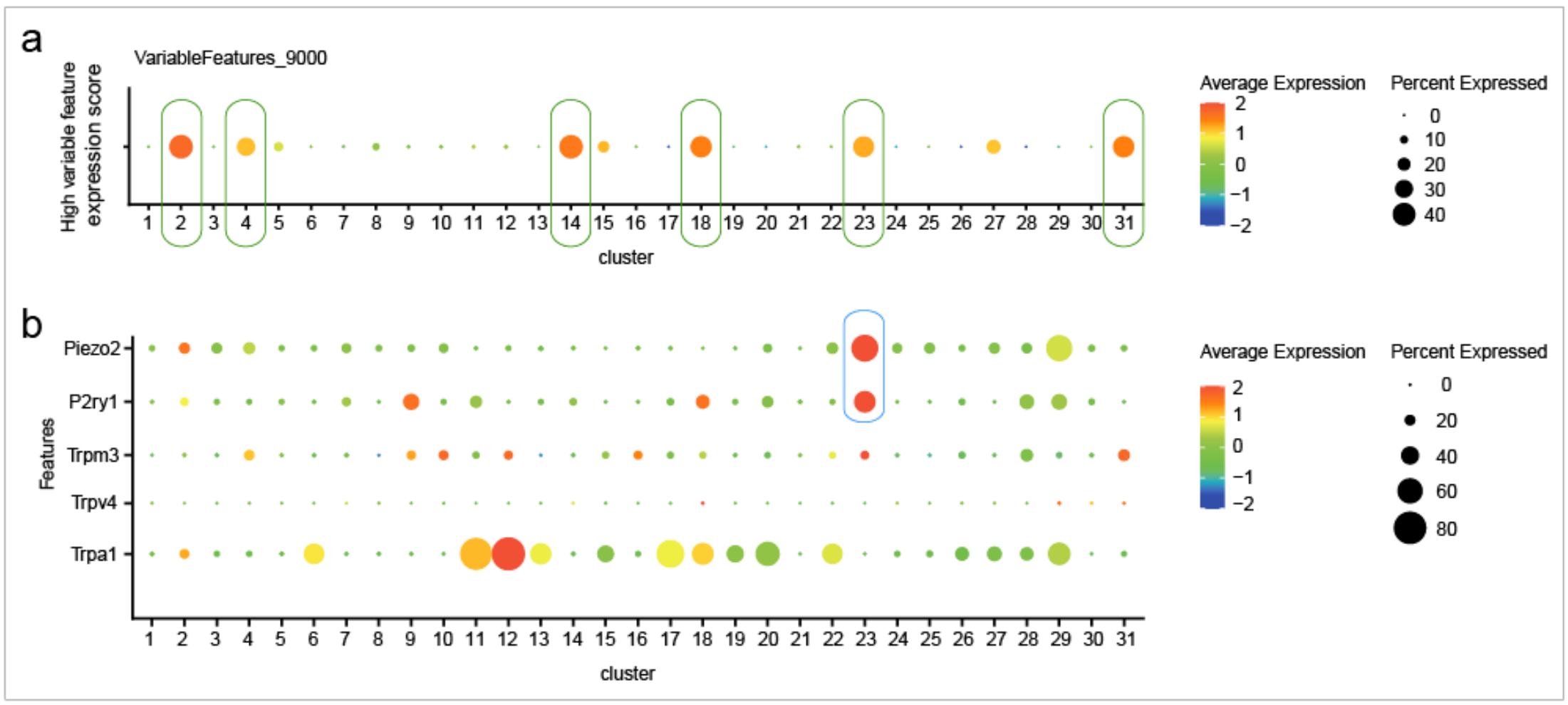

Response Document Figure 3 | Transcriptome similarity analysis of DsRed labeled nodose ganglion neurons from TH+ NTS neurons with nodose ganglion neural clusters, and dotplot of these signature scores.

We believe these data will help you gain a deeper understanding of our single-cell analysis results. Once again, we would like to express our sincere gratitude for your valuable feedback; these revisions will significantly enhance the scientific rigor and readability of our single-cell data presentation.
